# Late‐Stage Modification of Halotryptophan‐Containing Peptides Via Negishi Cross‐Coupling

**DOI:** 10.1002/chem.71177

**Published:** 2026-05-25

**Authors:** Laura Cerveson, Marcel‐Jannik Heldt, Norbert Sewald

**Affiliations:** ^1^ Department of Chemistry, Organic and Bioorganic Chemistry Bielefeld University Bielefeld Germany

**Keywords:** amino acids, bromotryptophan, late‐stage modification, medicinal chemistry, negishi cross‐coupling, non‐proteinogenic amino acids, palladium‐catalyzed coupling reactions, peptide, solubility

## Abstract

Late‐stage functionalization of peptides provides powerful opportunities for the rapid diversification of bioactive sequences. Among the available strategies, Negishi cross‐coupling represents a versatile and orthogonal method for the installation of alkyl substituents under mild conditions. We describe the applicability of Negishi cross‐coupling to increasingly complex bromotryptophan‐containing peptides. While conditions effective for bromo‐l‐tryptophan proved unsuitable for peptide substrates, leading to incomplete conversion and pronounced debromination, systematic optimization enabled efficient cross‐coupling of the model tripeptide Ac‐Ala‐Trp(6‐Br)‐Gly‐NH_2_. The optimized protocol afforded good to excellent conversions and isolated yields while maintaining operational simplicity. A key limitation arising from peptide solubility was addressed through the yet underexplored use of DMSO and DMSO/DMF solvent systems in Negishi cross‐couplings, which enabled efficient reaction of peptides bearing either 6‐ or 7‐bromo‐l‐tryptophan as well as unprotected residues such as aspartic acid and serine. Application to more complex pentapeptides revealed solvent‐dependent reactivity: poor solubility in DMF resulted in reduced conversion and increased debromination, whereas DMSO‐containing solvent systems significantly improved both solubility and catalytic performance. These results demonstrate that Negishi cross‐coupling can be adapted for the late‐stage functionalization of bromotryptophan‐containing peptides with functional group tolerance in structurally complex peptide substrates.

## Graphical abstract

Negishi cross‐coupling provides access to the late‐stage functionalization of bromotryptophan‐containing peptides under comparatively mild conditions and is applicable to a range of peptide substrates in the investigated model systems. The examined alkyl iodides and variation of N‐terminal residues demonstrate the feasibility of the transformation in a peptide context and highlight its potential utility for peptide diversification, while also indicating the current limitations in substrate scope and functional‐group tolerance.

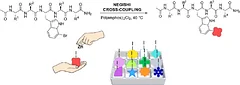

## 1 Introduction

Site‐specific peptide and protein modifications have produced a wide spectrum of derivatives with modified properties or biological effects [1] spanning therapeutic and non‐therapeutic applications [2, 3, 4]. Consequently, the demand for these modified biomolecules continues to grow especially in fields such as chemical biology and peptide therapeutics [5].

A highly versatile approach towards the selective modification of halogenated amino acids, peptides, and proteins uses cross‐coupling reactions [6, 7]. For many of them their compatibility with functional groups present in peptides and proteins has been well demonstrated [1]. However, due to the complex and chiral nature of such biomolecules, mild reaction conditions are generally required, and, therefore, not all cross‐coupling reactions are considered suitable for their modification.

Among the various amino acids, tryptophan is a particularly attractive substrate. This essential amino acid is a key component of many bioactive natural products due to its spectrophotometric properties [8] and plays a central role in folding and stabilization of higher‐order structures in peptides and proteins through intra‐ and intermolecular interactions [9, 10]. Moreover, although it accounts for only ∼1% of amino acid residues in proteins, it is disproportionately represented in protein‐protein interactions [1, 11, 12]. By modulating its steric and electronic properties through functionalization, it becomes possible to tune the fluorescence, conformation, and activity of biomolecules [13, 14, 15].

The derivatization of halotryptophans has long been neglected [16], largely due to the particular challenges posed by the indole scaffold in metal‐mediated cross‐coupling chemistry [17]. However, in recent years, biocatalytic strategies [18, 19, 20] have provided a more straightforward and robust route to halotryptophans, enabling access to electronically less favored positions and thereby stimulating their use as substrates in a range of Pd‐catalyzed cross‐coupling reactions, such as Suzuki–Miyaura coupling [21, 22, 23, 24, 25, 26], Sonogashira coupling [9], Mizoroki–Heck coupling [14, 27], as well as C─O and C─N bond‐forming reactions [13, 28].

**SCHEME 1:**
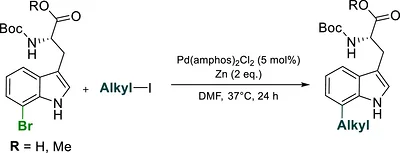
Negishi cross‐coupling of 7‐bromo‐l‐tryptophan with various alkyl iodides under mild conditions without pre‐generation of organozinc reagents [29].

In 2021, Dachwitz et al. described the first mild, direct alkylation of 7‐bromo‐l‐tryptophan derivatives via Negishi cross‐coupling using alkyl iodides [29], notably without the need to pre‐generate organozinc reagents or pre‐activate the zinc (Scheme 1). A variety of primary alkyl iodides, as well as secondary alkyl iodides, were successfully used as coupling partners to both fully protected 7‐bromotryptophan derivatives and N^α^‐Boc‐protected variants bearing a free carboxyl group.

## 2 Results and Discussion

Given the mild conditions, operational simplicity, and the strategic value of modifying a key residue such as tryptophan, the method reported by Dachwitz et al. offers substantial potential for adaptation to more complex systems. We, therefore, sought to extend this approach to the late‐stage diversification of bromotryptophan‐containing peptides.

An exploratory study was first conducted on the model tripeptide Ac‐Ala‐Trp(6‐Br)‐Gly‐NH_2_ to evaluate the applicability of the protocol developed by Dachwitz et al. to increasingly complex substrates. This substrate was designed to minimize potential side reactions: apart from tryptophan, the other amino acid side chains are non‐interfering under the reaction conditions. In addition, both the N‐ and C‐termini were protected to avoid undesired coordination or reactivity. Moreover, 6‐bromo‐l‐tryptophan was selected over 7‐bromo‐l‐tryptophan because of its higher reactivity in cross‐coupling reactions.

Starting from the original procedure reported by Dachwitz et al., some small modifications were also implemented during preliminary tests. First, due to the sensitivity of the Negishi cross‐coupling towards water, anhydrous N,N‐dimethylformamide (DMF) was employed, and the catalyst was stored in a desiccator to minimize exposure to moisture. Furthermore, zinc dust was subjected to an activation procedure prior to use. Prolonged exposure of zinc to air can result in surface oxidation, forming a passivating oxide layer that diminishes reactivity and hampers the formation of the organozinc species. The equivalents of both zinc and alkyl iodide were increased to 4 equivalents. Literature precedents indicate that an excess (3‐4 equivalents) of alkylzinc reagents is often required to achieve satisfactory yields in cross‐coupling reactions [30]. Finally, vigorous stirring ensured reproducibility of the reaction, and particular care was also taken to maintain an inert atmosphere without compromising the operational simplicity of the protocol.

**SCHEME 2:**
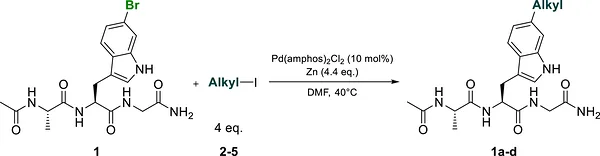
Optimized reaction conditions for Negishi cross‐coupling of Ac‐Ala‐Trp(6‐Br)‐Gly‐NH_2_ with alkyl iodides (Table 1).

Under these conditions, the key reaction (Scheme 2) was subsequently evaluated with various alkyl iodides (**2‐5**, Table 1).

**TABLE 1:** Scope of alkyl iodides in the Negishi cross‐coupling of Ac‐Ala‐Trp(6‐Br)‐Gly‐NH_2_ (Scheme 2, see also Supporting Information).

Entry	Alkyl iodide	#	Conversion (%)^a^, ^b^	Yield (%)
1	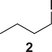	**1a**	79 (61,18)	59
2	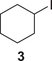	**1b**	89 (65, 24)	55
3	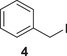	**1c**	>99 (99, 0)	97
4	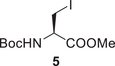	**1d**	92 (87, 5)	86

^a^ Determined by UHPLC‐MS at 220 nm.

^b^ Values in brackets indicate the percentage of product and debrominated peptide (relative to conversion).

A stock solution of the peptide in anhydrous DMF was prepared and purged with argon prior to addition to a reaction vial containing Pd(amphos)_2_Cl_2_, activated zinc dust, and the alkyl iodide dissolved in DMF and likewise purged with argon. The suspension was vigorously stirred at 40°C, and reaction progress was monitored by UHPLC–MS at 220 nm. After purification by reversed‐phase high‐performance liquid chromatography (RP–HPLC), conversions and isolated yields ranged from good to excellent (Table 1, entries 1–4).

The best results were obtained with benzyl iodide (entry 3) and N^α^‐Boc‐l‐3‐iodoalanine methyl ester (entry 4), respectively. Notably, no debrominated peptide was detected in the reaction with benzyl iodide. In contrast, reactions with 1‐iodobutane and iodocyclohexane proceeded less efficiently, displaying lower conversions and higher levels of dehalogenation, although the overall outcomes remained satisfactory.

Interestingly, the reaction with 1‐iodobutane afforded the lowest conversion, which was unexpected. Negishi cross‐coupling reactions involving primary alkyl iodides are generally more favorable than those with secondary or tertiary iodides, owing to the greater stability and lower propensity for *β*‐hydride elimination of their corresponding zinc organyls.

**SCHEME 3:**
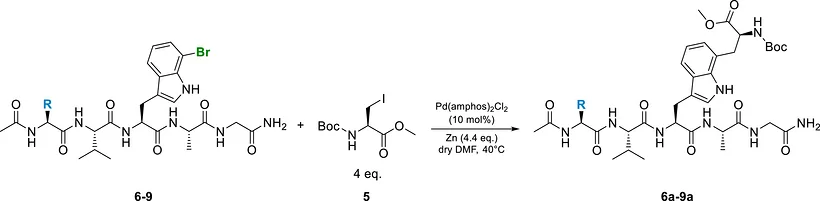
Negishi cross‐coupling of 7‐bromo‐l‐tryptophan containing pentapeptides with N^α^‐Boc‐l‐3‐iodoalanine methyl ester in DMF (Table 2).

Encouraged by the promising results obtained with the tripeptide, the methodology was extended to more challenging substrates. A series of bromotryptophan‐containing pentapeptides was designed to investigate compatibility with more complex peptide structures. All pentapeptides shared the same backbone sequence, predominantly composed of aliphatic amino acids, with variation at the N‐terminal residue. Initially, a Leu‐containing pentapeptide (**6**) was selected to assess the effect of increased peptide length while retaining non‐interfering aliphatic side chains. Subsequently, leucine was replaced with aspartic acid (carboxylic acid in the side chain, **7**), serine (hydroxyl in the side chain, **8**), and methionine (thioether in the side chain, **9**) to evaluate the influence of different functional groups. In all cases, the N‐ and C‐termini were protected. Moreover, 6‐bromo‐l‐tryptophan was replaced with the more challenging 7‐bromo‐l‐tryptophan. For the first set of reactions, N^α^‐Boc‐l‐3‐iodoalanine methyl ester was selected as the coupling partner, given its favorable performance with the tripeptide and its synthetic relevance compared to benzyl iodide. Along this strategy, two peptide chains may be linked across an indole moiety (Scheme 3). Significant solubility issues were encountered for all pentapeptides in DMF, resulting in poor conversions and substantial formation of debrominated peptide (Table 2).

**TABLE 2:** Negishi cross‐coupling of 7‐bromo‐l‐tryptophan containing pentapeptides with N^α^‐Boc‐l‐3‐iodoalanine methyl ester in DMF to investigate the influence of different amino acids' side chains (Scheme 3, see also Supporting Information).

Entry	Peptide	#	Solvent	Conversion (%)^a^, ^b^	Yield
5	Ac‐**Leu**‐Val‐Trp(7‐Br)‐Ala‐Gly‐NH_2_	**6a**	DMF	18 (9,9)	22^c^
6	Ac‐**Asp**‐Val‐Trp(7‐Br)‐Ala‐Gly‐NH_2_	**7a**	DMF	13 (0,13)	n.d.^d^
7	Ac‐**Ser**‐Val‐Trp(7‐Br)‐Ala‐Gly‐NH_2_	**8a**	DMF	58 (32, 26)	21
8	Ac‐**Met**‐Val‐Trp(7‐Br)‐Ala‐Gly‐NH_2_	**9a**	DMF	82 (25, 57)	29^c^

^a^ Determined by UHPLC‐MS at 220 nm.

^b^ Values in brackets indicate the percentage of product and debrominated peptide (relative to conversion).

^c^ Contamination with side‐product Boc‐Ala‐OMe.

^d^ Not determined.

The Leu‐ (entry 5) and Met‐ (entry 8) pentapeptides were isolated in modest yields (20%–30%). However, ^1^H‐NMR analysis revealed the presence of Boc‐Ala‐OMe, likely arising from protodehalogenation of N^α^‐Boc‐l‐3‐iodoalanine methyl ester. This byproduct was not detected by UHPLC–MS and likely co‐eluted with the desired product during RP‐HPLC purification. The Asp‐containing pentapeptide (entry 6) exhibited the lowest conversion, with complete formation of the debrominated product. The Ser‐containing pentapeptide (entry 7) was the only one which was successfully isolated in 21% yield. Due to the pronounced solubility limitations, no definitive conclusions could be drawn regarding the intrinsic influence of the different side chains.

**SCHEME 4:**
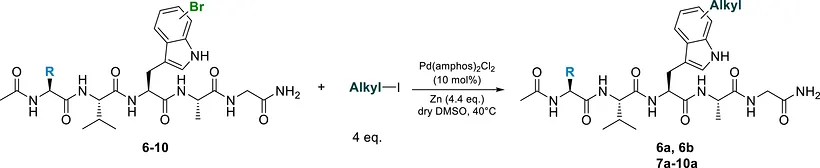
Negishi cross‐coupling of bromo‐l‐tryptophan containing pentapeptides with various alkyl iodides in DMSO (Table 3).

Given that the poor solubility in DMF was considered the primary cause of poor conversions, alternative solvents were evaluated. Tetrahydrofuran (THF) and DMF are two of the commonly employed polar aprotic solvents in Negishi cross‐coupling reactions [30]. Dachwitz et al. reported poor outcomes with THF and excellent results with DMF in the alkylation of 6‐bromoindole. DMF has been shown to suppress β‐elimination, particularly for amino acid‐derived alkylzinc reagents, by inhibiting intramolecular coordination of the carbamate carbonyl group to zinc, which otherwise accelerates β‐hydride elimination [31, 32]. Considering this and the limited solubility of the peptides in DMF, THF was not pursued further. Instead, dimethyl sulfoxide (DMSO) was evaluated due to its superior solubilizing properties. No precedent for Negishi cross‐coupling in DMSO was found in the literature, although it is known that DMSO is a polar aprotic solvent with strong coordinating ability, which could potentially negatively affect catalyst activity and organozinc stability. In anhydrous DMSO, all pentapeptides dissolved completely, and the reaction showed improved conversions (70%–80%), albeit without full conversion and with approximately 25% debromination (Scheme 4, Table 3).

**TABLE 3:** Negishi cross‐coupling of bromo‐l‐tryptophan containing pentapeptides in DMSO: Scope of alkyl iodides, and influence of different amino acids side chains, and of the bromide position (Scheme 4, see also Supporting Information).

Entry	Peptide	Alkyl iodide	Solvent	#	Conversion (%)^a^, ^b^	Yield (%)
9	Ac‐**Leu**‐Val‐**Trp(7‐Br)**‐Ala‐Gly‐NH_2_	Boc‐3‐iodo‐l‐ala‐OMe	DMSO	**6a**	68 (45, 23)	41
10	Ac‐**Leu**‐Val‐**Trp(7‐Br)**‐Ala‐Gly‐NH_2_	Benzyl iodide	DMSO	**6b**	71 (53, 18)	46
11	Ac‐**Asp**‐Val‐**Trp(7‐Br)**‐Ala‐Gly‐NH_2_	Boc‐3‐iodo‐l‐ala‐OMe	DMSO	**7a**	70 (47, 23)	25
12	Ac‐**Ser**‐Val‐**Trp(7‐Br)**‐Ala‐Gly‐NH_2_	Boc‐3‐iodo‐l‐ala‐OMe	DMSO	**8a**	70 (44, 26)	45
13	Ac‐**Met**‐Val‐**Trp(7‐Br)**‐Ala‐Gly‐NH_2_	Boc‐3‐iodo‐l‐ala‐OMe	DMSO	**9a**	81 (14, 67)	15^c^
14	Ac‐**Leu**‐Val**‐Trp(6‐Br)**‐Ala‐Gly‐NH_2_	1‐iodobutane	DMSO	**10a**	79 (69,10)	53

^a^ Determined by UHPLC‐MS at 220 nm.

^b^ Values in brackets indicate the percentage of product and debrominated peptide (relative to conversion).

^c^ Contamination with side‐product Boc‐Ala‐OMe.

The Met‐containing pentapeptide (entry 13) showed the highest overall conversion but predominantly formed the debrominated product, with an isolated yield of only 15%. ^1^H‐NMR analysis again revealed the presence of Boc‐Ala‐OMe. These results indicate that the palladium catalyst is not poisoned by the thioether functionality of Met during the reaction, like previously observed [23]. In our previous studies, we observed that the presence of a methionine residue within a peptide containing bromotryptophan can significantly accelerate Pd‐catalyzed Suzuki–Miyaura cross‐coupling reactions, whereas addition of Boc‐protected methionine to the Suzuki–Miyaura reaction did not lead to an acceleration but effected inhibition of the catalysis [23]. We attributed this difference to intramolecular coordination of the thioether moiety to the Pd center within the peptide framework, while free thioether‐containing additives act as classical catalyst poisons. In the Negishi case, the transformation does not proceed along the desired pathway, and dehalogenation is instead favored. The remaining pentapeptides displayed similar performances with conversions around 70% and debromination ranging from 18% to 26%. Based on these preliminary results, substitution of Leu with Asp or Ser in the peptide sequence does not appear to significantly affect the reaction outcome.

For comparison, the Leu‐containing pentapeptide was reacted with benzyl iodide in DMSO (entry 10), affording 71% conversion and 46% isolated yield, comparable to entry 9.

Finally, a pentapeptide containing 6‐bromo‐l‐tryptophan instead of 7‐bromo‐l‐tryptophan, Ac‐Leu‐Val‐Trp(6‐Br)‐Ala‐Gly‐NH_2_ (**10**), was reacted with 1‐iodobutane in DMSO (entry 14). This reaction provided the best result in DMSO, achieving 79% conversion with only 10% debromination and an isolated yield of 53%. This outcome aligns with the higher reactivity of 6‐bromo‐l‐tryptophan, for which oxidative addition is facilitated due to its lower electron density compared to the 7‐bromo isomer.

Neither DMF nor DMSO alone proved optimal solvents for the reaction. Although DMF is more suitable for the cross‐coupling step, peptide solubility is limited, whereas DMSO ensures full dissolution but may interfere with the catalytic cycle. Therefore, mixtures of DMF and DMSO were evaluated, maintaining a higher proportion of DMF.

**SCHEME 5:**
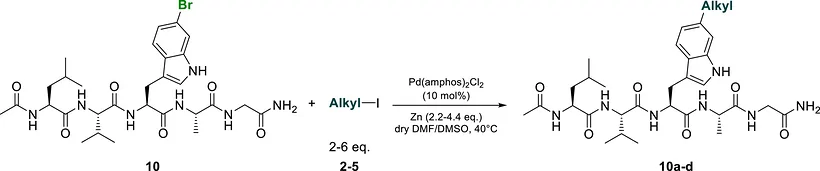
Negishi cross‐coupling of Ac‐Leu‐Val‐Trp(6‐Br)‐Ala‐Gly‐NH_2_ with various alkyl iodides, mixtures of DMF/DMSO (Table 4).

As an initial experiment, the cross‐coupling of Ac‐Leu‐Val‐Trp(6‐Br)‐Ala‐Gly‐NH_2_ (Scheme 5, **10**) with 1‐iodobutane was performed in DMF/DMSO (3:1, Table 4, entry 15). Despite incomplete solubility, the reaction afforded 95% conversion and only 5% debromination, outperforming either solvent alone.

**TABLE 4:** Negishi cross‐coupling of Ac‐Leu‐Val‐Trp(6‐Br)‐Ala‐Gly‐NH_2_ with various alkyl iodides in mixtures of DMF/DMSO (Scheme 5, see also Supporting Information).

Entry	Alkyl iodide	Eq.	Solvent	#	Conversion (%)^a^, ^b^	Yield (%)
15	1‐iodobutane	5.1	DMF/DMSO 3:1	10a	95 (90, 5)	75
16	iodocyclohexane	4.0	DMF/DMSO 3:2	10b	90 (81, 9)	68
17	Benzyl iodide	5.9	DMF/DMSO 3:2	10c	98 (92, 6)	83
18	Boc‐3‐iodo‐l‐ala‐OMe	4.0	DMF/DMSO 3:2	10d	85 (80, 5)	90^c^
19	Boc‐3‐iodo‐l‐ala‐OMe	2.0	DMF/DMSO 3:2	10d	82 (74, 8)	55

^a^ Determined by UHPLC‐MS at 220 nm.

^b^ Values in brackets indicate the percentage of product and debrominated peptide (relative to conversion).

^c^ Contamination with side‐product Boc‐Ala‐OMe.

Increasing the DMSO content to a 3:2 DMF/DMSO mixture resulted in complete peptide dissolution. Under these conditions, good conversions were achieved for all alkyl iodides, with debromination below 10% (Table 4). The observed reactivity trend, benzyl iodide > 1‐iodobutane > iodocyclohexane > N^α^‐Boc‐3‐iodo‐l‐ala‐OMe was consistent with expectations based on alkyl iodide structure.

However, in the reaction involving N^α^‐Boc‐3‐iodo‐l‐ala‐OMe (Table 4, entry 18), ^1^H‐NMR analysis of the isolated product again revealed the presence of Boc‐Ala‐OMe, likely due to similar retention times and challenging chromatographic separation. To mitigate this issue, the reaction was repeated using a lower amount of alkyl iodide (2 eq., entry 19). Although conversion decreased slightly (82%) and peptide debromination also slightly increased, careful purification by RP‐HPLC enabled isolation of the desired product with minor impurities in 55% yield.

## 3 Conclusion

In this study, the applicability of the Negishi cross‐coupling to increasingly complex peptide substrates was evaluated and optimized. While the original conditions proved effective for bromo‐l‐tryptophan alone, their direct translation to bromotryptophan‐containing peptides resulted in incomplete conversion and significant debromination. Through screening reaction and small, targeted modifications, efficient cross‐coupling could be achieved on the model tripeptide Ac‐Ala‐Trp(6‐Br)‐Gly‐NH_2_. Good conversions and isolated yields were obtained, demonstrating that the methodology can be successfully adapted to short peptide substrates while retaining operational simplicity.

Extension of the protocol to more complex pentapeptides revealed solvent‐dependent limitations. Poor solubility in DMF significantly hampered reactivity and promoted debromination. Switching to DMSO improved peptide solubility and increased conversion, but the best overall performance was achieved using mixed DMF/DMSO systems, which balanced peptide solubility and catalytic efficiency. Under these conditions, high conversions and low levels of dehalogenation were obtained.

With a suitable solvent system now established, a more systematic investigation of the intrinsic influence of amino acid side chains on the cross‐coupling outcome can be undertaken. Such studies will enable a clearer understanding of the functional group tolerance of this methodology and further define its scope for the diversification of structurally complex and functionally rich peptide substrates.

## 4 Experimental Section

The chemicals used were commercially available, purchased from BLDpharm, Carbolution Chemicals, Fisher Scientific, Roth, Sigma‐Aldrich, Thermo Fisher Scientific, Fluorochem, or VWR, and were used as received without further purification.

Upon receipt, alkyl iodides were transferred to a Schlenk flask and stored under an inert atmosphere in the absence of light. The catalyst Pd(amphos)_2_Cl_2_ was stored in a desiccator under reduced pressure.

Dichloromethane (DCM) and ethyl acetate were purchased as technical grade and distilled before usage. All other solvents were used as purchased (analytical grade). For further drying, DCM was freshly distilled over CaH_2_, while N,N‐dimethylformamide (DMF) was dried over activated 4 Å molecular sieves and stored under an inert atmosphere. Deionized water was used in every case, and MPW was purified by PURELAB flex 2.

### 4.1 Activation of Zinc Dust [33]

Zinc powder (16 g) was added to a 300 mL, round‐bottomed flask containing 2% (v/v) HCl_aq_ (100 mL) under vigorous stirring. Stirring was continued for approximately 4 min, until the surface of the zinc became visibly bright. The acidic solution was then decanted, and the zinc powder was washed by decantation with deionized water (4 × 200 mL). The zinc was subsequently transferred to a Büchner funnel using deionized water (200 mL) and washed successively with ethanol (50 mL), acetone (100 mL), and dry diethyl ether (50 mL). Filtration and washing steps were carried out as rapidly as possible, preferably under an argon atmosphere, to minimize exposure of the activated zinc to air. The activated zinc was dried under reduced pressure at 100°C for 1 h, then allowed to cool and stored under inert atmosphere. Prior to use, the zinc was heated under vacuum at 100°C for approximately 30 min.

### 4.2 General Procedure for Pd‐Catalyzed Negishi Cross‐Coupling on Peptides (GP1)

A stock solution of the peptide was prepared in anhydrous DMF, DMSO, or a mixture of them and degassed under argon atmosphere for 30 min. Separately, activated zinc dust (4.4 eq.), Pd(amphos)_2_Cl_2_ (10 mol%), and the alkyl iodide (if solid) (4.0 eq.) were charged into a glass vial, which was subsequently placed under argon atmosphere. After 10 min anhydrous DMF, DMSO, or a mixture of them, the alkyl iodide (if liquid) (4.0 eq.), and the peptide stock solution (1.0 eq.) were added sequentially to the vial. In some experiments, the reaction mixture was purged with argon for 5 min after each addition, whereas in others it was purged once for 5 min upon completion of all additions. The vial was sealed, and the suspension was vigorously stirred at 40°C for 24–48 h. When the reaction time elapsed, the mixture was allowed to cool to room temperature and centrifuged. The crude product was purified by preparative RP‐HPLC.

### 4.3 General Procedure for Solid‐Phase Peptide Synthesis (GP2)

The peptides were synthesized employing the Fmoc/*^t^* Bu‐strategy. The resin loading and the following coupling steps were performed in a syringe, and using an automatic shaker, or an ultrasonic bath alternated with the automatic shaker.

#### 4.3.1 Resin Loading

Rink‐amide resin was swollen in DMF (10 mL/g resin) for 10 min at room temperature. The solvent was removed, and the Fmoc‐group attached to the resin was cleaved with a solution of 20% piperidine and 100 mm HOBt in DMF (10 mL/g resin). After removal of the cleavage solution, the resin was washed with DMF (3 × 10 mL/g resin), DCM (3 × 10 mL/g resin), and DMF (3 × 10 mL/g resin) again, and the syringe filled with the solution of the loading amino acid (4.0 eq.), Oxyma (4.0 eq.) and DIC (4.0 eq.) in DMF (10 mL/g resin, 1 h on shaker at r.t. or 5 min ultrasonic bath 40°C followed by 5 min on shaker at r.t.). The resin was washed with DMF (3 × 10 mL/g resin), DCM (3 × 10 mL/g resin), and DMF (3 × 10 mL/g resin) again and dried with DCM (3×) and Et_2_O (5×) under vacuum. Resin loading was determined by UV‐analysis of the piperidine dibenzofulvene adduct formed upon cleavage of the Fmoc protecting group with 20% piperidine in DMF.

#### 4.3.2 Determination of Resin Loading

A small amount (≈1 mg) of resin (*m*) was incubated with 3 mL of 20% piperidine in DMF 3 h. The absorption (A) at λ = 290 nm was measured, and the resin loading was calculated with the following formula:

$$loading\;\;\left[\frac{mmol}{g}\right]=\frac{A\left(290\;\mathrm{nm}\right)}{m\;\left[\mathrm{mg}\right]\cdot 1.65}$$

#### 4.3.3 Fmoc‐Deprotection and Coupling of Amino Acids

When necessary, the resin was first swollen in DMF (10 mL/g resin) for 10 min at room temperature. Fmoc‐cleavage was performed by addition of 20% piperidine in DMF (10 mL/g resin) and using either the shaker (2 × 20 min at r.t.) or the ultrasonic bath at 40°C (30 sec the first time, 2 min the second) followed by washing with DMF (3 × 10 mL/g resin), DCM (3 × 10 mL/g resin) and DMF (3 × 10 mL/g resin).

For the coupling step, Fmoc‐protected amino acids were coupled to the *N* ^α^‐deprotected peptide by addition of a solution of amino acid (4.0 eq.), Oxyma (4.0 eq.), and DIC (4 eq.) in DMF (10 mL/g resin) to the resin, and incubation of the reaction mixture for 1 h on the shaker at r.t. or sonication (5 min at 40°C) followed by 5 min on the shaker. Afterwards, the resin was washed again with DMF (3 × 10 mL/g resin), DCM (3×10 mL/g resin), and DMF (3×10 mL/g resin). Full conversion was verified by analysis via LC‐MS after test cleavage. For the measurement, the resin was dried with DCM (3×) and Et_2_O (5×) under vacuum, then approx 10 beads were transferred into an Eppendorf tube and treated with 100 µL of TFA/TIS/MPW (95:2.5:2.5). The mixture was incubated for 10 min and then diluted with 500–1000 µL of ACN/MPW (1:1) and analyzed by LC‐MS.

For the coupling of Fmoc‐7‐bromo‐l‐tryptophan the following coupling procedure was used: Fmoc‐7‐bromo‐l‐tryptophan (1.1 eq.), Oxyma (1.1 eq.), and DIC (1.1 eq.) in DMF (10 mL/g resin) were added to the resin. The reaction mixture was sonicated at 40°C for 5 min and then incubated for 5 min on the shaker. The coupling was repeated twice, then the resin was subjected to a capping step in which the remaining unreacted sites were capped. For this, a solution of acetic anhydride (10 eq.) and pyridine (10 eq.) in DMF (10 mL/g resin) was added to the resin, and the reaction mixture was incubated 20 min on the shaker at room temperature (2×).

#### 4.3.4 *N*‐Acetylation

After coupling the last amino acid, the peptide was *N*‐acetylated by first cleaving the Fmoc‐group and then adding a solution of acetic anhydride (10 eq.) and pyridine (10 eq.) in DMF (10 mL/g resin) to the resin and incubating of the reaction mixture on the shaker (2 × 20 min at r.t.).

#### 4.3.5 Cleavage of the Peptide

The resin was incubated for 2 h with a mixture of TFA/TIS/MPW (95:2.5:2.5). The solution with the cleaved peptide was transferred into a flask containing isopropanol. The resin was briefly washed with 2 mL of the same cleavage mixture. The solvent was evaporated, and the residue was dissolved in water and lyophilized.

Alternatively, after rinsing the resin in the syringe, the two peptide fractions were combined in a flask containing cold diethyl ether and stored in the freezer overnight to promote peptide precipitation. The mixture was then centrifuged (2000 g, 4°C, 10 min), the supernatant discarded, and the residue dissolved in water and lyophilized.

The peptide was purified by RP‐HPLC.

## Conflicts of Interest

The authors declare no conflicts of interest.

## Supporting information




**Supporting File**: Full characterization details of the synthesized compounds is provided in the Supporting Information. The authors have cited additional references within the Supporting Information [34].

## Data Availability

The data that supports the findings of this study are available in the supplementary material of this article.
