# Supplementary material for: Late‐Stage Modification of Halotryptophan‐Containing Peptides Via Negishi Cross‐Coupling
Source: Chemistry. 2026 May 25;32(34):e71177. doi: 10.1002/chem.71177 (PMC13569947; doi:10.1002/chem.71177)
Supplement: Supplementary file 1 [file CHEM-32-e71177-s001.pdf]

Table of contents

Instruments ... 2
General Procedures ... 5
Compounds ... 8
6-Bromo-L-tryptophan ... 8
7-Bromo-L-tryptophan ... 10
N^{α}-Fmoc-6-bromo-L-tryptophan ... 12
N^{α}-Fmoc-7-bromo-L-tryptophan ... 14
Benzyl iodide (4) ... 16
Ac-Ala-Trp(6-Br)-Gly-NH2 (1) ... 18
Ac-Leu-Val-Trp(7-Br)-Ala-Gly-NH_2 (6) ... 20
Ac-Asp-Val-Trp(7-Br)-Ala-Gly-NH_2 (7) ... 22
Ac-Ser-Val-Trp(7-Br)-Ala-Gly-NH_2 (8) ... 24
Ac-Met-Val-Trp(7-Br)-Ala-Gly-NH_2 (9) ... 26
Ac-Leu-Val-Trp(6-Br)-Ala-Gly-NH_2 (10) ... 28
Ac-Ala-Trp(6-butyl)-Gly-NH_2 (1a) ... 30
Ac-Ala-Trp(6-cyclohexyl)-Gly-NH_2 (1b) ... 33
Ac-Ala-Trp(6-benzyl)-Gly-NH_2 (1c) ... 36
Peptide 1d ... 39
Peptide 6a ... 42
Ac-Leu-Val-Trp(7-benzyl)-Ala-Gly-NH_2 (6b) ... 45
Peptide 7a ... 48
Peptide 8a ... 51
Peptide 9a ... 54
Ac-Leu-Val-Trp(6-butyl)-Ala-Gly-NH_2 (10a) ... 57
Ac-Leu-Val-Trp(6-cyclohexyl)-Ala-Gly-NH_2 (10b) ... 60
Ac-Leu-Val-Trp(6-benzyl)-Ala-Gly-NH_2 (10c) ... 63
Peptide 10d ... 66
Supplementary figures ... 69
References ... 74

Instruments

Nuclear Magnetic Resonance Spectroscopy

NMR spectroscopy was done on a Bruker Avance III 500 HD (500 MHz for ^{1}H, 126 MHz for $^{13}C\{^{1}H\}$, 471 MHz for $^{19}F\{^{13}C\}$) or Bruker Avance NEO 600 (600 MHz for ^{1}H, 150 MHz for $^{13}C\{^{1}H\}$, 565 MHz for $^{19}F\{^{13}C\}$). The chemical shift δ is reported in ppm relative to the residual proton signal or the ^{13}C signal, resp. of the solvent: DMSO-d_6: δ = 2.50 ppm (^{1}H), δ = 39.52 ppm ($^{13}C\{^{1}H\}$); Acetone-d_6: δ = 2.05 ppm (^{1}H); δ = 29.84 ppm ($^{13}C\{^{1}H\}$). 2D methods (^{1}H,^{1}H-COSY, ^{1}H,^{13}C-HMQC, ^{1}H,^{13}C-HMBC) were used to support and confirm the assignment.

Liquid Chromatography Mass Spectrometry

LC-MS analysis was performed on an Agilent 6220 time-of-flight mass spectrometer (Agilent Technologies) in extended dynamic range mode equipped with a Dual-ESI source, operating with a spray voltage of 2.5 kV. Nitrogen served both as nebulizer gas and dry gas. Nitrogen was generated by a nitrogen generator NGM 11. Samples were dissolved in ACN/water 1:1 and introduced with a 1200 HPLC system consisting of an autosampler, degasser, binary pump, column oven (40 °C) and diode array detector (Agilent Technologies, at 220 nm and 254 nm) using a C18 Hypersil Gold column (length: 50 mm, diameter: 2.1 mm, particle size: 1.9 µm).
The mass axis was externally calibrated with ESI-L Low Concentration Tuning Mix (Agilent Technologies) as calibration standard. The mass spectra are recorded in both profile and centroid mode with the MassHunter Workstation Acquisition B.04.00 software (Agilent Technologies). MassHunter Qualitative Analysis B.07.00 software (Agilent Technologies) was used for processing and averaging of several single spectra.

Analytical run protocol:

Time / min	Eluent A[a] / %	Eluent B[b] / %	Flow rate / mL·min^{-1}
0.0	98	2	0.3
10.0	2	98	0.3
11.0	2	98	0.3
11.5	98	2	0.3
15.0	98	2	0.3

[a] Eluent A: water/acetonitrile/formic acid, 94.9:5:0.1, v/v/v
[b] Eluent B: acetonitrile/water/formic acid, 94.9:5:0.1, v/v/v

High Resolution Mass Spectrometry

Accurate Mass nano-ESI measurements are performed using a Q-IMS-TOF mass spectrometer Synapt G2Si (Waters GmbH) in resolution mode, interfaced to a nano-ESI ion source. Nitrogen serves both as the nebulizer gas and the dry gas for nano-ESI. Nitrogen is generated by a nitrogen

generator NGM 11. Samples were dissolved in ACN or ACN/water mixtures (if necessary acidified using formic acid) and introduced by static nano-ESI using in-house pulled glass emitters.
The mass axis was externally calibrated with ESI-L Low Concentration Tuning Mix (Agilent Technologies) as calibration standard and internally calibrated with the protonated LeuEnk ion as internal calibration standard. Scan accumulation and data processing was performed with MassLynx 4.1 (Waters GmbH) on a PC Workstation. The spectra shown here were generated by the accumulation and averaging of several single spectra. Determination of exact masses were performed using centroided data.

Ultra-High Performance Liquid Chromatography Mass Spectrometry

UHPLC-MS analysis was performed on an AmaZonX ESI ion trap mass spectrometer (Bruker) in extended dynamic range mode equipped with an ESI source, operating with a spray voltage of 4.5 kV. Nitrogen served both as nebulizer gas and dry gas. Nitrogen was generated by a nitrogen generator G6-0-1-0-E (Parker-Hannifin, model G4600E). Samples were dissolved in ACN/water 1:1 and introduced with an Ultimate 3000RS UHPLC system (Thermo Fischer Scientific) consisting of an autosampler, degasser, binary pump, column oven (60 °C) and diode array detector using a C_{18} Kinetex column (Phenomenex, length: 100 mm, diameter: 2.1 mm, particle size: 1.7 µm).
The mass axis was externally calibrated with ESI-T Tuning Mix (Agilent technologies) as calibration standard. The mass spectra were recorded in profile mode. Compass Data Analysis 4.2 software (Bruker) was used for processing and averaging of several single spectra.

Analytical run protocol:

Time / min	Eluent A[a] / %	Eluent B[b] / %	Flow rate / $mL \cdot min^{-1}$
0.0	100	0	0.75
3.5	0	100	0.75
4.0	0	100	0.75
4.5	100	0	0.75
5.0	100	20	0.75

[a] Eluent A: water/acetonitrile/formic acid, 94.9:5:0.1, v/v/v
[b] Eluent B: acetonitrile/water/formic acid, 94.9:5:0.1, v/v/v

Reverse-Phase High-Pressure Liquid Chromatography

Purification by preparative RP-HPLC was done on a Shimadzu Nexera series consisting of a system controller (SCL-40), UV-VIS detector (SPD-40V, at 200 nm, 220 nm 254 nm and 280 nm) and a degassing unit (DGU-405). As column a Shimadzu Phenomenex Luna C18 (length: 250.0 mm, diameter: 21.2 mm, particle size: 5.0 µm) was used.

Preparative run protocol 1:

Time / min	Eluent A[a] / %	Eluent B[b] / %	Flow rate / mL·min^{-1}
0.0	90	10	10.0
30.0	0	100	10.0
40.0	0	100	10.0

[a] Eluent A: water/acetonitrile/TFA, 94.9:5:0.1, v/v/v
[b] Eluent B: acetonitrile/water/TFA, 94.9:5:0.1, v/v/v

Preparative run protocol 2:

Time / min	Eluent A[a] / %	Eluent B[b] / %	Flow rate / mL·min^{-1}
0.0	90	10	10.0
5.0	90	10	10.0
65.0	30	70	10.0
70.0	0	100	10.0
80.0	0	100	10.0

[a] Eluent A: water/acetonitrile/TFA, 94.9:5:0.1, v/v/v
[b] Eluent B: acetonitrile/water/TFA, 94.9:5:0.1, v/v/v

Preparative run protocol 3:

Time / min	Eluent A[a] / %	Eluent B[b] / %	Flow rate / mL·min^{-1}
0.0	95	5	10.0
5.0	95	5	10.0
75.0	25	75	10.0
80.0	0	100	10.0
90.0	0	100	10.0

[a] Eluent A: water/acetonitrile/TFA, 94.9:5:0.1, v/v/v
[b] Eluent B: acetonitrile/water/TFA, 94.9:5:0.1, v/v/v

Preparative run protocol 4:

Time / min	Eluent A[a] / %	Eluent B[b] / %	Flow rate / mL·min^{-1}
0.0	97	3	10.0
5.0	97	3	10.0
32.0	70	30	10.0
52.0	60	40	10.0
72.0	40	60	10.0
75.0	0	100	10.0
85.0	0	100	10.0

[a] Eluent A: water/acetonitrile/TFA, 94.9:5:0.1, v/v/v
[b] Eluent B: acetonitrile/water/TFA, 94.9:5:0.1, v/v/v

Fully automatic flash chromatography system

An automatic flash chromatography system Pure C-815 Flash BÜCHI (Flawil, Switzerland) was used. UV vis detector: silicon photodiode; ELSD detector light source: laser (0.3 mW). Columns:

Flash Pure Silica, pore size 40 µm, Flash Pure Select Silica, pore size 25 µm; Flash Pure Select C_{18}, pore size 30 µm.

MPLC run protocol 1:

Time / min	Eluent A[a] / %	Eluent B[b] / %	Flow rate / mL·min^{-1}
0.0	100	0	30.0
5.0	100	0	30.0
35.0	0	100	30.0
40.0	0	100	30.0

[a] Eluent A: water
[b] Eluent B: acetonitrile

MPLC run protocol 2:

Time / min	Eluent A[a] / %	Eluent B[b] / %	Flow rate / mL·min^{-1}
0.0	100	0	30.0
5.0	100	0	30.0
35.0	95	5	30.0
45.0	90	10	30.0
50.0	90	10	30.0

[a] Eluent A: dichloromethane
[b] Eluent B: methanol

General Procedures

Activation of zinc dust[1]

Zinc powder (16 g) was added with to a 300 mL, round-bottomed flask containing 2% (v/v) HCl_{aq} (100 mL) under vigorous stirring. Stirring was continued for approximately 4 min, until the surface of the zinc became visibly bright. The acidic solution was then decanted, and the zinc powder was washed by decantation with deionized water (4×200 mL). The zinc was subsequently transferred to a Büchner funnel using distilled water (200 mL) and washed successively with ethanol (50 mL), acetone (100 mL), and dry diethyl ether (50 mL). Filtration and washing steps were carried out as rapidly as possible, preferably under an argon atmosphere, to minimize exposure of the activated zinc to air. The activated zinc was dried under reduced pressure at 100 °C for 1 h, then allowed to cool and stored under inert atmosphere. Prior to use, the zinc was heated under vacuum at 100 °C for approximately 30 min.

GP1: *General procedure for Pd-catalysed Negishi cross-coupling on peptides*

A stock solution of the peptide was prepared in anhydrous DMF, DMSO or a mixture of them and degassed under argon atmosphere for 30 min. Separately, activated zinc dust (4.4 eq.), $Pd(amphos)_2Cl_2$ (10 mol%) and the alkyl iodide (if solid) (4.0 eq.) were charged into a glass vial,

which was subsequently placed under argon atmosphere. After 10 min anhydrous DMF, DMSO or a mixture of them, the alkyl iodide (if liquid) (4.0 eq.), and the peptide stock solution (1.0 eq.) were added sequentially to the vial. In some experiments, the reaction mixture was purged with argon for 5 min after each addition, whereas in others it was purged once for 5 min upon completion of all additions. The vial was sealed and the suspension was vigorously stirred at 40 °C for 24-48 h. When the reaction time elapsed, the mixture was allowed to cool to room temperature and centrifuged. The crude product was purified by preparative RP-HPLC.

GP2: *General procedure for Solid-Phase Peptide Synthesis*

The peptides were synthesized employing the Fmoc/^{t}Bu-strategy. The resin loading and the following coupling steps were performed in a syringe and using an automatic shaker, or an ultrasonic bath alternated with the automatic shaker.

Resin loading: Rink-amide resin was swollen in DMF (10 mL/g resin) for 10 min at room temperature. The solvent was removed, and the Fmoc-group attached to the resin was cleaved with a solution of 20% piperidine and 100 mm HOBt in DMF (10mL/g resin). After removal of the cleavage solution, the resin was washed with DMF (3×10 mL/g resin), DCM (3×10 mL/g resin) and DMF (3×10 mL/g resin) again and the syringe filled with the solution of the loading amino acid (4.0 eq.), Oxyma (4.0 eq.) and DIC (4.0 eq.) in DMF (10 mL/g resin, 1 h on shaker at r.t. or 5 min ultrasonic bath 40 °C followed by 5 min on shaker at r.t.). The resin was washed with DMF (3×10 mL/g resin), DCM (3×10 mL/g resin) and DMF (3×10 mL/g resin) again and dried with DCM (3×) and Et_2O (5×) under vacuum. Resin loading was determined by UV-analysis of the piperidine dibenzofulvene adduct formed upon cleavage of the Fmoc protecting group with 20% piperidine in DMF.

Determination of resin loading: a small amount (≈1 mg) of resin (*m*) was incubated with 3 mL of 20% piperidine in DMF 3 h. The absorption (A) at λ=290 nm was measured, and the resin loading (L) calculated with the following formula:

$$loading\ \left[\frac{mmol}{g}\right] = \frac{\mathrm{A(290\ nm)}}{m\ [\mathrm{mg}] \cdot 1.65}$$

Fmoc-deprotection and coupling of amino acids: when necessary, the resin was first swollen in DMF (10 mL/g resin) for 10 min at room temperature. Fmoc-cleavage was performed by addition of 20% piperidine in DMF (10 mL/g resin) and using either the shaker (2×20 min at r.t) or the ultrasonic bath at 40 °C (30 sec the first time, 2 min the second) followed by washing with DMF (3×10 mL/g resin), DCM (3×10 mL/g resin) and DMF (3×10 mL/g resin).

For the coupling step, Fmoc-protected amino acids were coupled to the N^{α}-deprotected peptide by addition of a solution of amino acid (4.0 eq.), Oxyma (4.0 eq.) and DIC (4 eq.) in DMF (10 mL/g resin) to the resin, and incubation of the reaction mixture for 1 h on the shaker at r.t. or sonication (5 min at 40 °C) followed by 5 min on the shaker. Afterwards the resin was washed again with DMF

(3×10 mL/g resin), DCM (3×10 mL/g resin) and DMF (3×10 mL/g resin). Full conversion was verified by analysis via LC-MS after test cleavage. For the measurement the resin was dried with DCM (3×) and Et_2O (5×) under vacuum, then approx. 10 beads were transferred into an Eppendorf tube and treated with 100 µL of TFA/TIS/MPW (95:2.5:2.5). the mixture was incubated for 10 min and then diluted with 500-1000 µL of ACN/MPW (1:1) and analysed by LC-MS.

For the coupling of Fmoc-7-bromo-L-tryptophan the following coupling procedure was used: Fmoc-7-bromo-L-tryptophan (1.1 eq.), Oxyma (1.1 eq.) and DIC (1.1 eq.) in DMF (10 mL/g resin) were added to the resin. The reaction mixture was sonicated at 40 °C for 5 min and then incubated for 5 min on the shaker. The coupling was repeated twice, then the resin was subjected to a capping step in which the remaining unreacted sites were capped. For this, a solution of acetic anhydride (10 eq.) and pyridine (10 eq.) in DMF (10 mL/g resin) were added to the resin and the reaction mixture was incubated 20 min on the shaker at room temperature (x2).

***N*-Acetylation:** after coupling the last amino acid, the peptide was *N*-acetylated by first cleaving the Fmoc-group and then adding a solution of acetic anhydride (10 eq.) and pyridine (10 eq.) in DMF (10 mL/g resin) to the resin and incubating of the reaction mixture on the shaker (2×20 min at r.t).

Cleavage of the peptide: the resin was incubated for 2 h with a mixture of TFA/TIS/MPW (95:2.5:2.5). The solution with the cleaved peptide was transferred into a flask containing isopropanol. The resin was briefly washed with 2 mL of the same cleavage mixture. The solvent was evaporated, and the residue dissolved in water and lyophilized.

Alternatively, after rinsing the resin in the syringe, the two peptide fractions were combined in a flask containing cold diethyl ether and stored in the freezer overnight to promote peptide precipitation. The mixture was then centrifuged (2000 g, 4 °C, 10 min), the supernatant discarded, and the residue dissolved in water and lyophilized.

The peptide was purified by RP-HPLC.

Compounds

6-Bromo-L-tryptophan

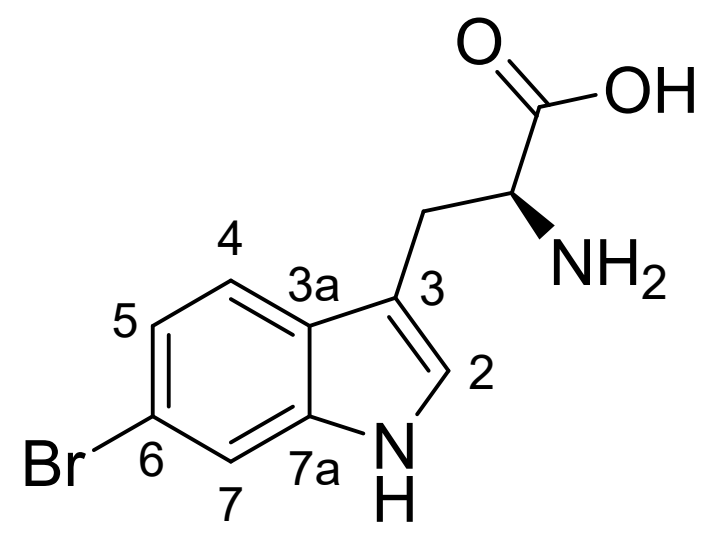

$C_{11}H_{11}BrN_2O_2$
MW = 283,13

6-Bromo-L-tryptophan was synthesized using combiCLEAs. The biocatalyst was generated from *E. coli* BL21 (DE3) pGro7 pETDuet-*thal-ptdh-borf* according to the procedure reported from *Montua et al.*[2] The reaction buffer consisted of 1 mM L-tryptophan, 15 mM Na_2HPO_4, 50 mM $Na_2HPO_3 \cdot 5H_2O$, 30 mM NaBr, 0.1 mM NAD, and 1 µM FAD at pH = 7.4 in a total reaction volume of 1 L. The mixture was shaken at 25°C for at least 2 days, while monitoring the reaction progress by RP-HPLC. To stop the reaction, the CLEAs were removed by centrifugation (4000 × *g*, 30 min) and the supernatant concentrated to a volume of about 50 mL. The crude residue was centrifuged again, and the cleared supernatant loaded on an RP-C_{18}-column for desalting. The loaded column was washed with 200 mL H_2O and then the product was eluted with 300 mL of MeOH. Volatiles were removed using a rotary evaporator. Lyophilization gave 6-bromo-L-tryptophan as a colourless to yellow solid.

^{1}H NMR (500 MHz, [D$_6$]DMSO): δ [ppm] = 11.18 (d, $^3J_{H,H}$ = 1.8 Hz, 1H; N**H**-indole), 7.56 (d, $^3J_{H,H}$ = 1.7 Hz, 1H; C7-**H**), 7.52 (d, $^3J_{H,H}$ = 8.5 Hz, 1H; C4-**H**), 7.25 (d, $^3J_{H,H}$ = 1.8 Hz, 1H; C2-**H**), 7.13 (dd, $^3J_{H,H}$ = 8.5 Hz, $^3J_{H,H}$ = 1.7 Hz, 1H; C5-**H**), 4.02 (dd, $^3J_{H,H}$ = 6.6 Hz $^3J_{H,H}$ = 5.7 Hz, 1H; C_α-**H**), 3.25 (dd, $^2J_{H,H}$ = 15.1 Hz, $^3J_{H,H}$ = 5.7 Hz, 1H; C_β-**H**), 3.18 (dd, $^2J_{H,H}$ = 15.1 Hz, $^3J_{H,H}$ = 6.6 Hz, 1H; C_β-**H**).

LC-MS: *m/z* calcd: 265.98 [M(^{79}Br)-NH$_2$]$^+$, 267.98 [M(^{81}Br)-NH$_2$]$^+$, 283.01 [M(^{79}Br)+H]$^+$, 285.01 [M(^{81}Br)+H]$^+$; found: 265.98 [M(^{79}Br)-NH$_2$]$^+$, 267.98 [M(^{81}Br)-NH$_2$]$^+$, 283.02 [M(^{79}Br)+H]$^+$, 285.01 [M(^{81}Br)+H]$^+$.

LC-MS chromatogram at λ=220 nm

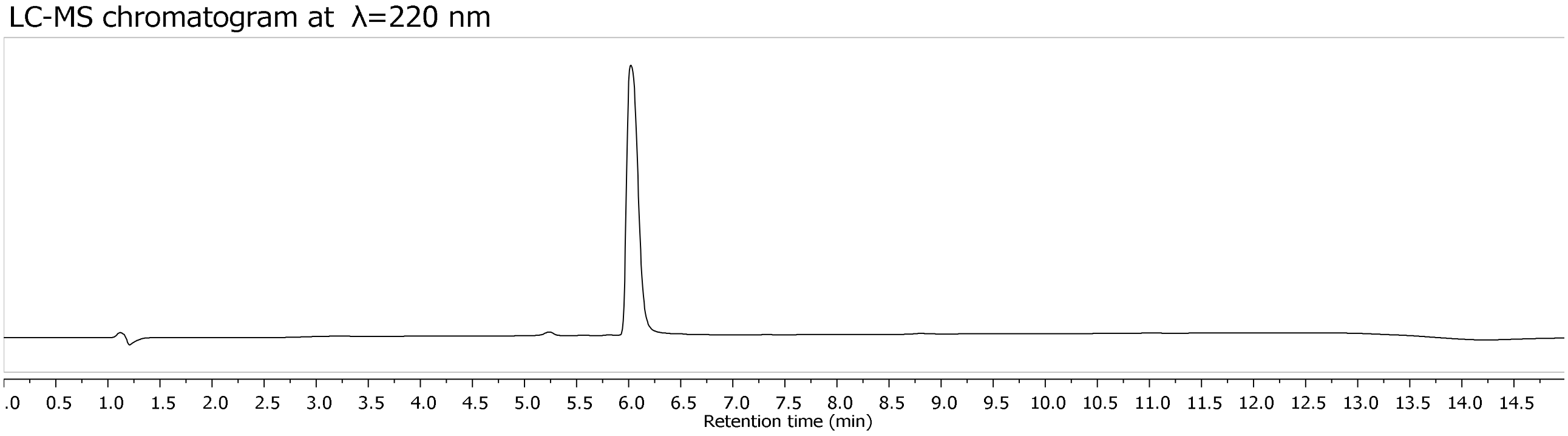

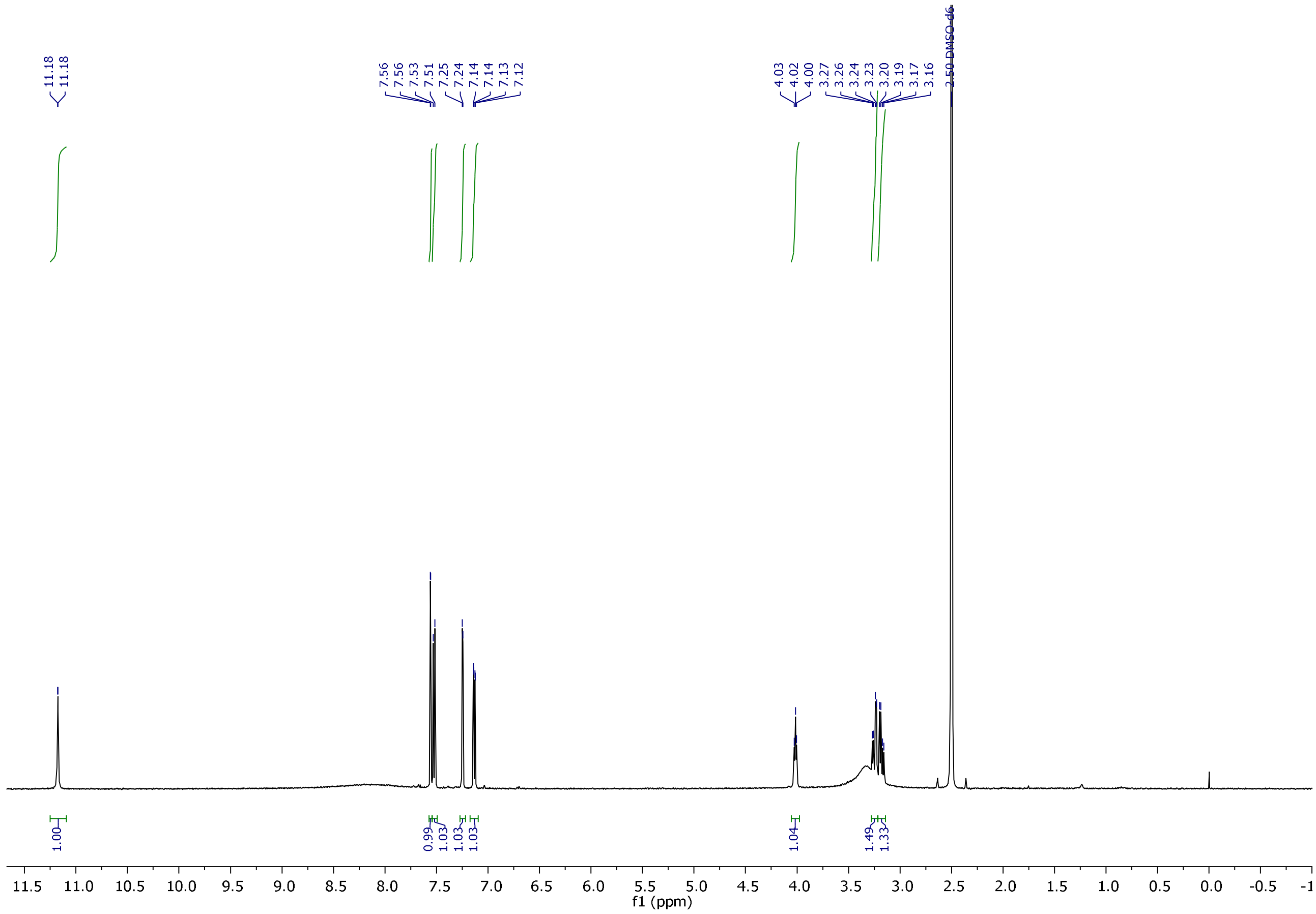
11.18
11.18
7.56
7.56
7.53
7.51
7.25
7.24
7.14
7.14
7.13
7.12
4.03
4.02
4.00
3.27
3.26
3.24
3.23
3.20
3.19
3.17
3.16
2.50 DMSO-d6
1.00
0.99
1.03
1.03
1.03
1.04
1.49
1.33
11.5
11.0
10.5
10.0
9.5
9.0
8.5
8.0
7.5
7.0
6.5
6.0
5.5
5.0
4.5
4.0
3.5
3.0
2.5
2.0
1.5
1.0
0.5
0.0
-0.5
-1
f1 (ppm)

7-Bromo-L-tryptophan

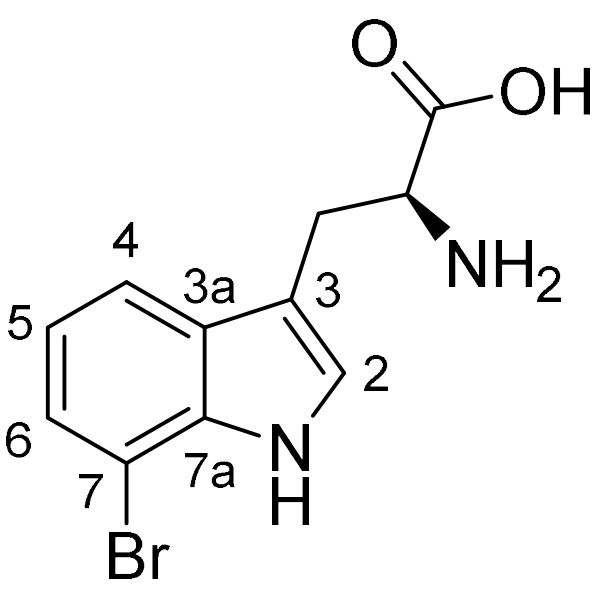

$C_{11}H_{11}BrN_2O_2$
MW = 283,13

7-Bromo-L-tryptophan was synthesized using combiCLEAs. The biocatalyst was generated from *E. coli* BL21 (DE3) pGro7 pETDuet-*rebH*-*ptdh*-*borf* according to the procedure reported from Montua et al.[3] The reaction buffer consisted of 1 mM L-tryptophan, 15 mM Na_2HPO_4, 50 mM $Na_2HPO_3 \cdot 5H_2O$, 30 mM NaBr, 0.1 mM NAD, and 1 µM FAD at pH = 7.4 in a total reaction volume of 1 L. The mixture was shaken at 25°C for at least 2 days while monitoring the reaction progress by RP-HPLC. To stop the reaction, the CLEAs were removed by centrifugation (4000 × *g*, 30 min) and the supernatant concentrated to a volume of about 50 mL. The crude residue was centrifuged again, and the cleared supernatant loaded on a 24 g RP-C_{18}-column and purified via MPLC (MPLC run protocol 1). Lyophilization gave 7-bromo-L-tryptophan as a colourless to yellow solid.

^{1}H NMR (500 MHz, [D_6]DMSO): δ [ppm] = 11.26 (s, 1H; N**H**-indole), 8.10 (s br, 2**H**; NH_2), 7.58 (d, $^3J_{H,H}$ = 7.7 Hz, 1H; C4-**H**), 7.32 (d, $^3J_{H,H}$ = 7.7 Hz, 1H; C6-**H**), 7.29 (d, $^3J_{H,H}$ = 2.3 Hz, 1H; C2-**H**), 6.97 (t, $^3J_{H,H}$ = 7.7 Hz, 1H; C5-**H**), 4.07 (dd, $^3J_{H,H}$ = 6.1 Hz, $^3J_{H,H}$ = 5.4 Hz; 1H, C_α-**H**), 3.27 (dd, $^2J_{H,H}$ = 15.2 Hz, $^3J_{H,H}$ = 5.4 Hz, 1H; C_β-**H**), 3.20 (dd, $^2J_{H,H}$ = 15.2 Hz, $^3J_{H,H}$ = 6.1 Hz, 1H; C_β-**H**).

LC-MS: *m/z* calcd: 265.98 [M(^{79}Br)-NH_2]$^+$, 267.98 [M(^{81}Br)-NH_2]$^+$, 283.01 [M(^{79}Br)+H]$^+$, 285.01 [M(^{81}Br)+H]$^+$; found: 265.98 [M(^{79}Br)-NH_2]$^+$, 267.98 [M(^{81}Br)-NH_2]$^+$, 283.02 [M(^{79}Br)+H]$^+$, 285.01 [M(^{81}Br)+H]$^+$.

LC-MS chromatogram at λ=220 nm

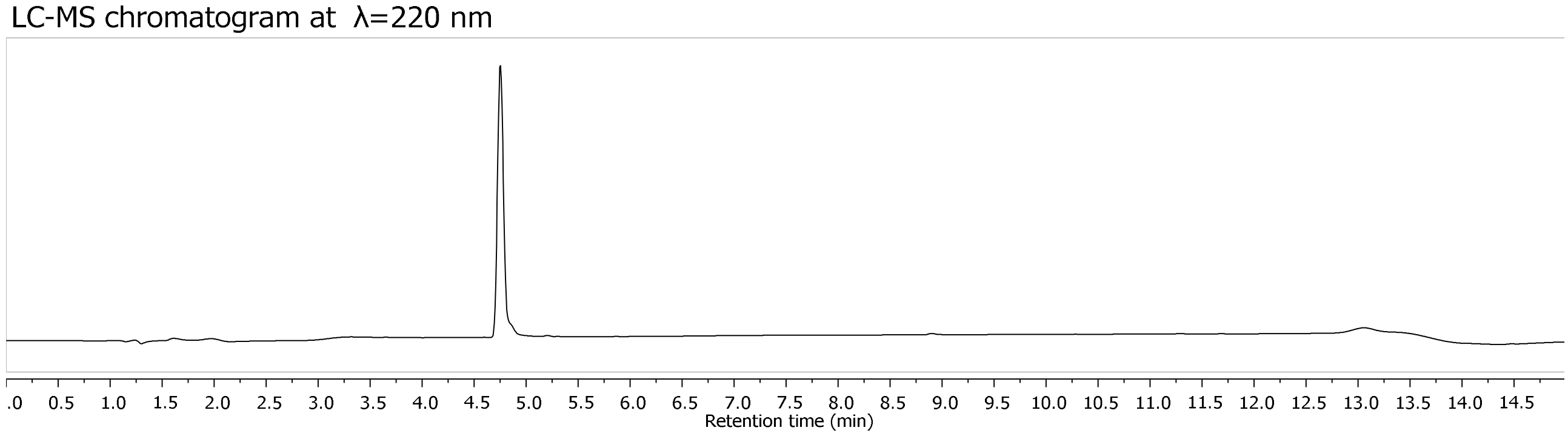

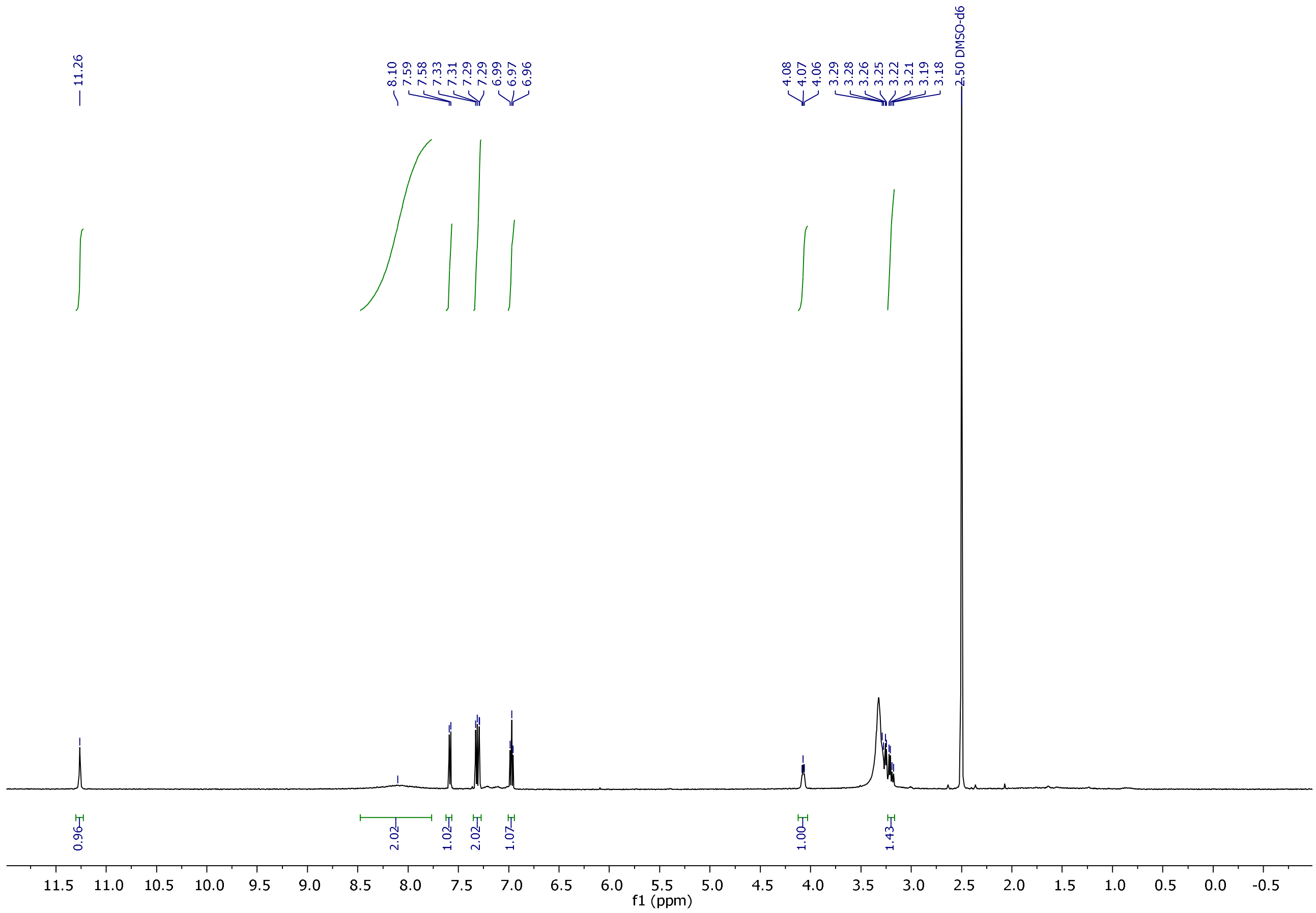

11.26
8.10
7.59
7.58
7.33
7.31
7.29
7.29
6.99
6.97
6.96
4.08
4.07
4.06
3.29
3.28
3.26
3.25
3.22
3.21
3.19
3.18
2.50 DMSO-d6
0.96
2.02
1.02
2.02
1.07
1.00
1.43
11.5 11.0 10.5 10.0 9.5 9.0 8.5 8.0 7.5 7.0 6.5 6.0 5.5 5.0 4.5 4.0 3.5 3.0 2.5 2.0 1.5 1.0 0.5 0.0 -0.5
f1 (ppm)

N$^{\alpha}$-Fmoc-6-bromo-L-tryptophan

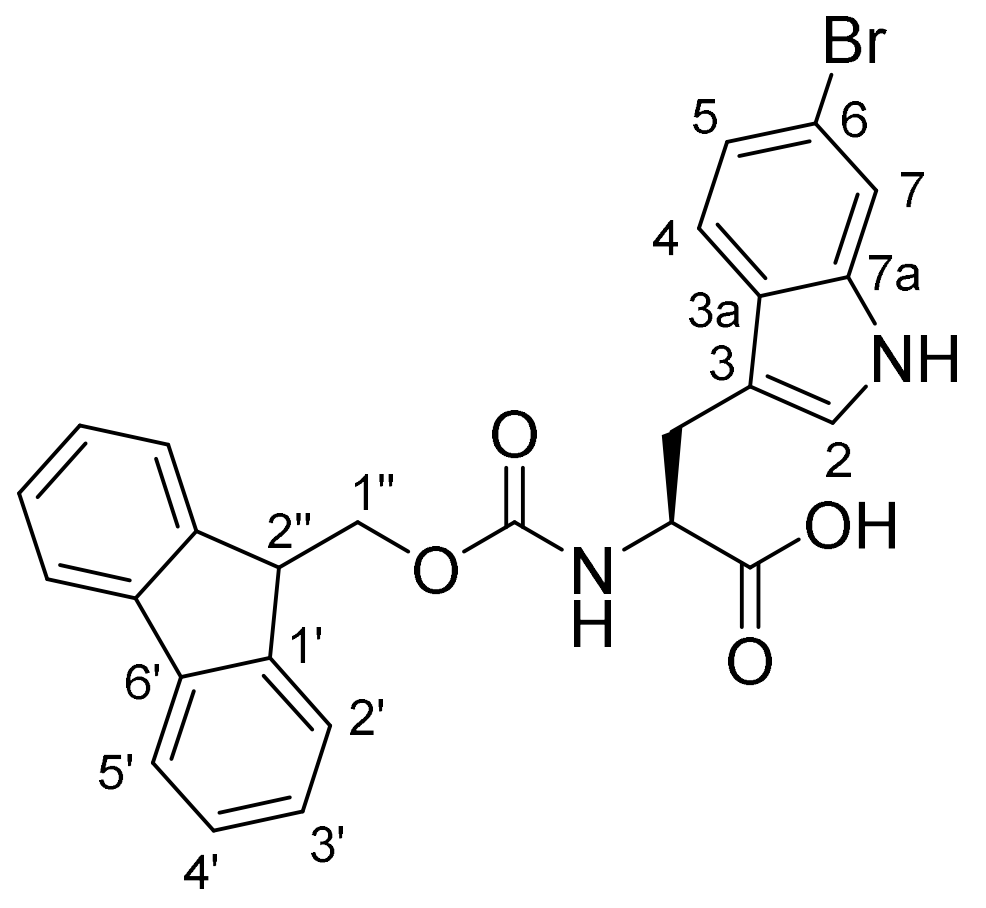

$C_{26}H_{21}BrN_2O_4$
MW = 505,37

For the synthesis of N$^{\alpha}$-Fmoc-6-bromo-L-tryptophan, 6-bromo-L-tryptophan (212.5 mg, 0.75 mmol, 1.0 eq.) was dissolved in H_2O/ACN 1:1 (10 mL). The pH was adjusted to a value of 9-10 by dropwise addition of KOH 1 M and then Fmoc-Osu was added (317.2 mg, 0.94 mmol, 1.3 eq.). The reaction mixture was stirred overnight. After 1 h, the pH was checked and readjusted to 9-10 with KOH 1 M. When full conversion was reached, EtOAc was added (20 mL), and the reaction mixture acidified to pH 3 with HCl 1 M. The crude was extracted with EtOAc (3×15 mL). The combined organic layers were washed with $KHSO_4$ 5% (3×50 mL) and saturated aqueous NaCl (2×50 mL), dried over $MgSO_4$, filtered, and concentrated under reduced pressure. The crude product was purified via MPLC (MPLC run protocol 2) to afford a colourless to yellow solid (281.5 mg, 557.0 µmol, 74%).

^{1}H NMR (500 MHz, [D$_6$]DMSO): δ [ppm] = 12.69 (s br, 1H; COO**H**), 11.00 (d, $^3J_{H,H}$ = 1.7 Hz, 1H; N**H**-indole), 7.88 (d, $^3J_{H,H}$ = 7.3 Hz, 2H; C5'-**H**), 7.70 (d, $^3J_{H,H}$ = 8.3 Hz, 1H; N**H**), 7.65 (d, $^3J_{H,H}$ = 8.2 Hz, 1H; C2'-**H**), 7.63 (d, $^3J_{H,H}$ = 8.2 Hz, 1H; C2'-**H**), 7.53 (d, $^3J_{H,H}$ = 6.9 Hz, 1H; C4-**H**), 7.52 (s, 1H; C7-**H**), 7.41 (dd, $^3J_{H,H}$ = 13.2 Hz, $^3J_{H,H}$ = 7.3 Hz, 2H; C4'-**H**), 7.33-7.24 (m, 2H; C3'-**H**), 7.20 (d, $^3J_{H,H}$ = 1.7 Hz, 1H; C2-**H**), 7.10 (dd, $^3J_{H,H}$ = 6.9 Hz, $^3J_{H,H}$ = 1.4 Hz, 1H; C5-**H**), 4.24-4.13 (m, 4H, C$_{\alpha}$-**H**, C2''-**H**; C**H$_2$**), 3.17 (dd, $^2J_{H,H}$ = 14.6 Hz, $^3J_{H,H}$ = 4.4 Hz, 1H; C$_{\beta}$-**H**), 3.01 (dd, $^2J_{H,H}$ = 14.6 Hz, $^3J_{H,H}$ = 9.8 Hz, 1H; C$_{\beta}$-**H**).

LC-MS: *m/z* calcd: 505.08 [M(^{79}Br)+H]$^+$, 507.07 [M(^{81}Br)+H]$^+$; found: 505.02 [M(^{79}Br)+H]$^+$, 507.01 [M(^{81}Br)+H]$^+$.

LC-MS chromatogram at λ=220 nm

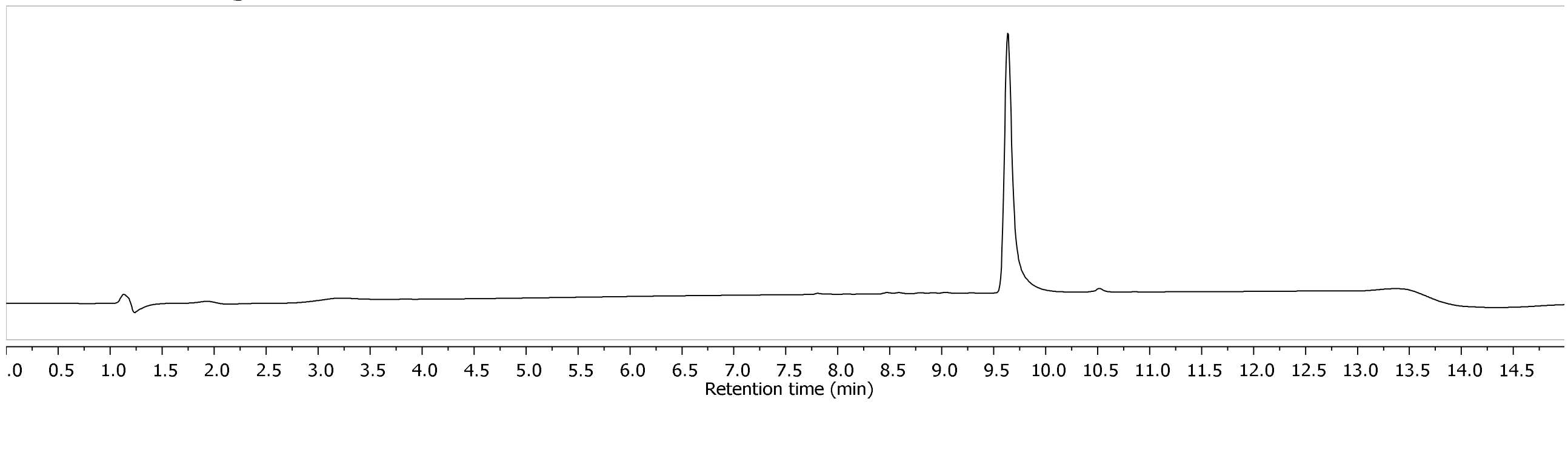

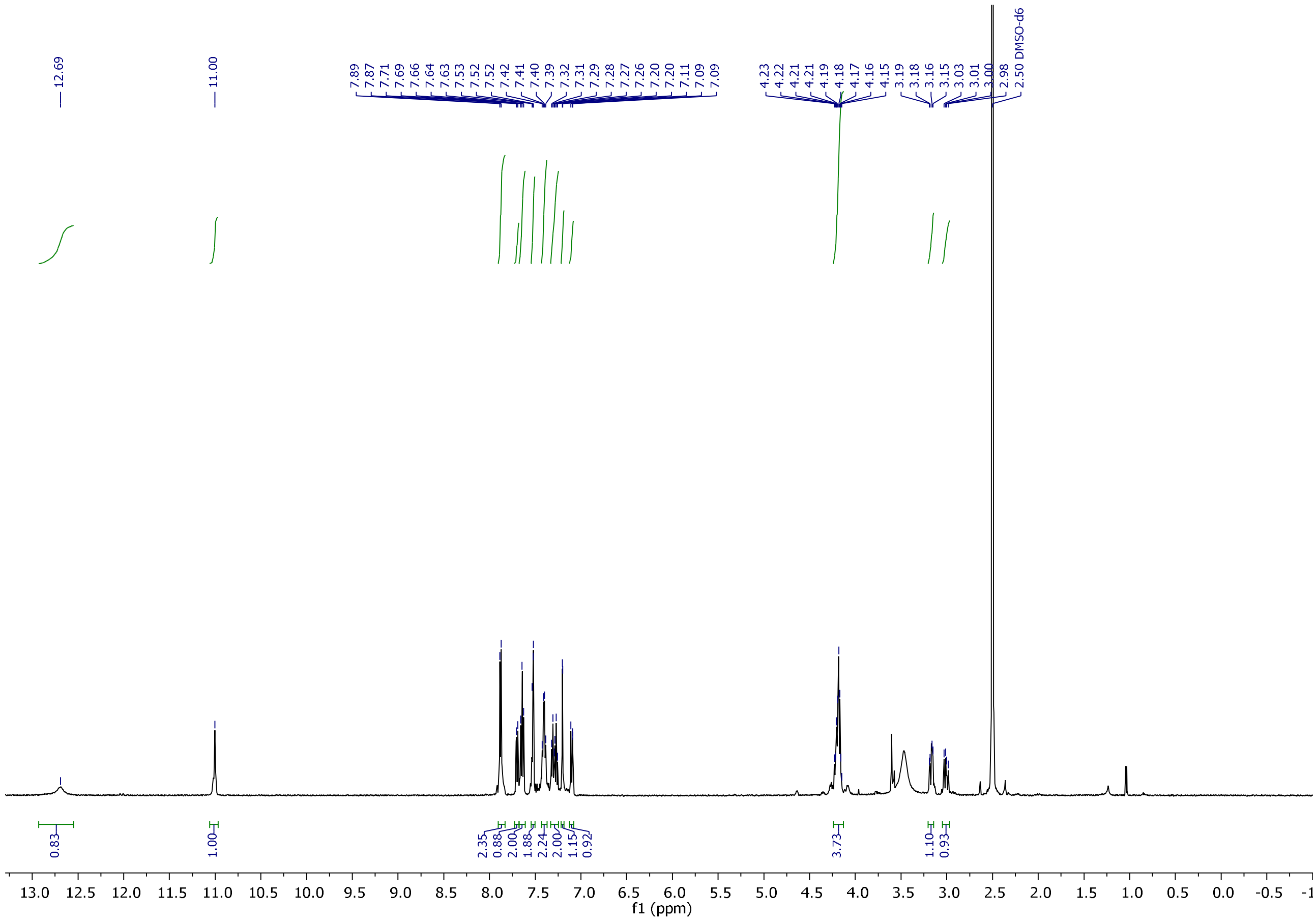
12.69
11.00
7.89
7.87
7.71
7.69
7.66
7.64
7.63
7.53
7.52
7.52
7.42
7.41
7.40
7.39
7.32
7.31
7.29
7.28
7.27
7.26
7.20
7.20
7.11
7.09
7.09
4.23
4.22
4.21
4.21
4.19
4.18
4.17
4.16
4.15
3.19
3.18
3.16
3.15
3.03
3.01
3.00
2.98
2.50 DMSO-d6
0.83
1.00
2.35
0.88
2.00
1.88
2.24
2.00
1.15
0.92
3.73
1.10
0.93
13.0 12.5 12.0 11.5 11.0 10.5 10.0 9.5 9.0 8.5 8.0 7.5 7.0 6.5 6.0 5.5 5.0 4.5 4.0 3.5 3.0 2.5 2.0 1.5 1.0 0.5 0.0 -0.5 -1
f1 (ppm)

N$^{\alpha}$-Fmoc-7-bromo-L-tryptophan

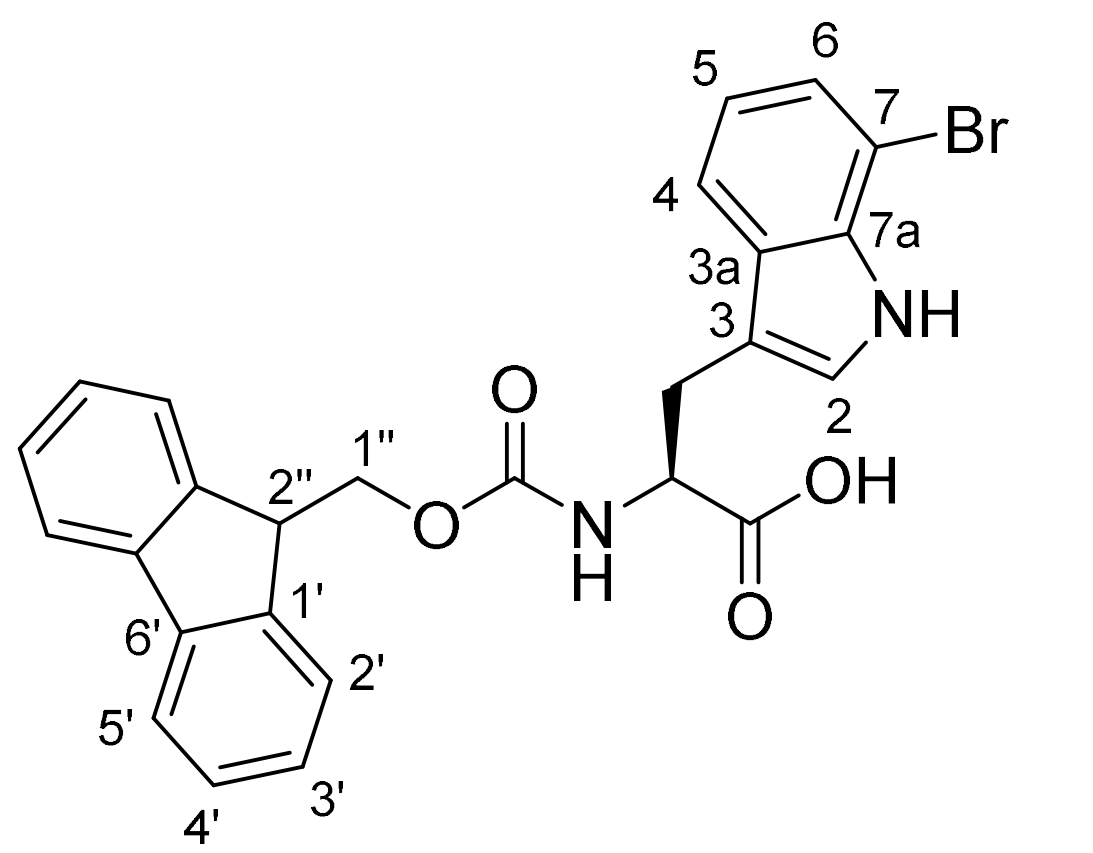

$C_{26}H_{21}BrN_2O_4$
MW = 505,37

For the synthesis of N$^{\alpha}$-Fmoc-7-bromo-L-tryptophan, 7-bromo-L-tryptophan (823.9 mg, 2.91 mmol, 1.0 eq.) was dissolved in H_2O/ACN 4:6 (50 mL). $NaHCO_3$ (730.7 mg, 8.70 mmol, 3 eq.) was added to the flask and the pH was adjusted to 8-9. After addition of Fmoc-Osu (1.470 g, 4.36 mmol, 1.5 eq.) the reaction mixture was stirred overnight. When full conversion was reached, EtOAc was added (50 mL), and the reaction mixture acidified to pH 3 with HCl 1.5 M. The crude was extracted with EtOAc (3×30 mL). The combined organic layers were washed with $KHSO_4$ 5% (3×100 mL) and saturated aqueous NaCl (2×100 mL), dried over $MgSO_4$, filtered, and concentrated under reduced pressure. The crude product was purified via MPLC (MPLC run protocol 2) to afford a colourless to yellow solid (945.0 mg, 1.87 mmol, 64%).

^{1}H NMR (500 MHz, [D$_6$]DMSO): δ [ppm] = 12.72 (s br, 1H; COO**H**), 11.11 (s, 1H; N**H**-indole), 7.88 (d, $^{3}J_{H,H}$ = 7.2 Hz, 2H; C5'-**H**), 7.73 (d, $^{3}J_{H,H}$ = 8.2 Hz, 1H; N**H**), 7.65 (d, $^{3}J_{H,H}$ = 7.5 Hz, 1H; C2'-**H**), 7.62 (d, $^{3}J_{H,H}$ = 7.7 Hz, 1H; C2'-**H**), 7.61 (d, $^{3}J_{H,H}$ = 7.8 Hz, 1H; C4-**H**), 7.40 (dd, $^{3}J_{H,H}$ = 13.9 Hz, $^{3}J_{H,H}$ = 7.2 Hz, 2H; C4'-**H**), 7.33-7.24 (m, 4H, C6-**H**, C3'-**H**; C2-**H**), 6.94 (t, $^{3}J_{H,H}$ = 7.8 Hz, 1H; C5-**H**), 4.26-4.14 (m, 4H, C_{α}-**H**, C2''-**H**; C**H$_2$**), 3.19 (dd, $^{2}J_{H,H}$ = 14.7 Hz, $^{3}J_{H,H}$ = 4.6 Hz, 1H; C_{β}-**H**), 3.02 (dd, $^{2}J_{H,H}$ = 14.7 Hz, $^{3}J_{H,H}$ = 9.8 Hz, 1H; C_{β}-**H**).

LC-MS: *m/z* calcd: 505.08 [M(^{79}Br)+H]$^{+}$, 507.07 [M(^{81}Br)+H]$^{+}$; found: 505.50 [M(^{79}Br)+H]$^{+}$, 507.60 [M(^{81}Br)+H]$^{+}$.

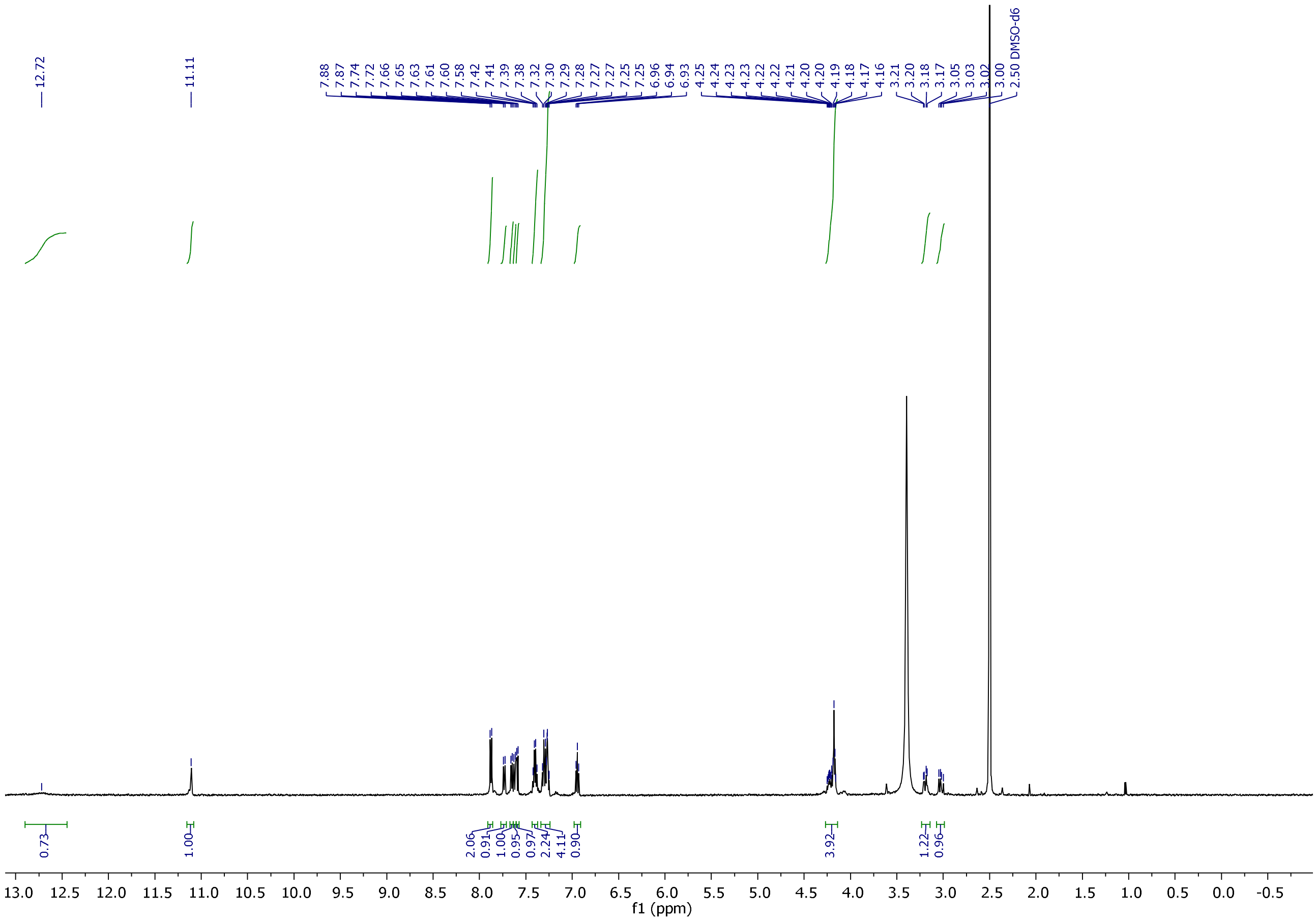

12.72
11.11
7.88
7.87
7.74
7.72
7.66
7.65
7.63
7.61
7.60
7.58
7.42
7.41
7.39
7.38
7.32
7.30
7.29
7.28
7.27
7.27
7.25
6.96
6.94
6.93
4.25
4.24
4.23
4.22
4.22
4.21
4.20
4.20
4.19
4.18
4.17
4.16
3.21
3.20
3.18
3.17
3.05
3.03
3.02
3.00
2.50 DMSO-d6
0.73
1.00
2.06
0.91
1.00
0.95
0.97
2.24
4.11
0.90
3.92
1.22
0.96
13.0 12.5 12.0 11.5 11.0 10.5 10.0 9.5 9.0 8.5 8.0 7.5 7.0 6.5 6.0 5.5 5.0 4.5 4.0 3.5 3.0 2.5 2.0 1.5 1.0 0.5 0.0 -0.5
f1 (ppm)

Benzyl iodide (4)

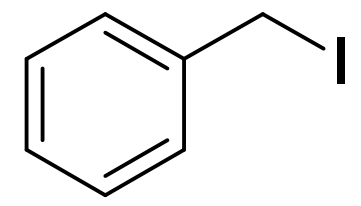

C_7H_7I
MW = 218,04

Benzyl iodide was prepared according to the protocol of Castle et. al[3]. Sodium iodide, NaI (9.11 g, 60.78 mmol, 1.4 eq.) was dissolved in acetone (60 mL) in a 100 mL round-bottom flask equipped with a magnetic stir bar. Benzyl chloride (5.00 mL, 43.45 mmol, 1.0 eq.) was added under stirring at room temperature. Ater 24 h, the solvent was removed under reduced pressure. The residue was dissolved in water (50 mL) and extracted with dichloromethane (5×30 mL). The combined organic layers were washed with 5% aqueous $KHSO_4$ solution (3×30 mL), then with brine (30 mL), dried over anhydrous $MgSO_4$, filtered, and concentrated under reduced pressure.

The crude product was purified by vacuum distillation to afford benzyl iodide as a pale brown crystalline solid (7.20 g, 33.02 mmol, 76%).

^{1}H NMR (500 MHz, [D$_6$]CD_3COCD_3): δ [ppm] = 7.45 (d, $^3J_{H,H}$ = 7.4 Hz, 2H; 2C**H**-*ortho*), 7.31 (dd, $^3J_{H,H}$ = 7.4 Hz, $^3J_{H,H}$ = 7.3 Hz, 2H; 2C**H**-*meta*), 7.25 (m, 1H; C**H**-*para*), 4.62 (s, 1H; C**H**$_2$).

UHPLC-MS chromatogram at λ = 220 nm

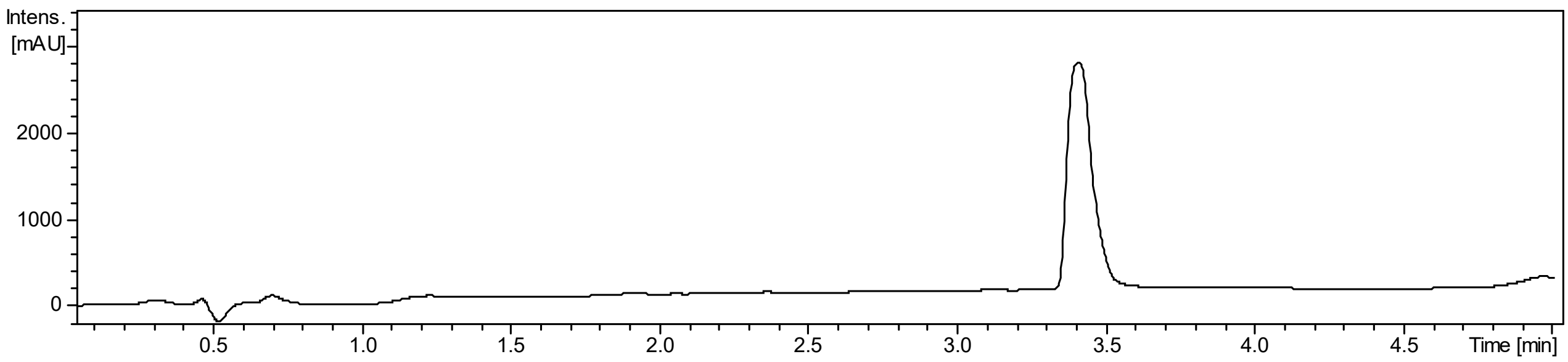

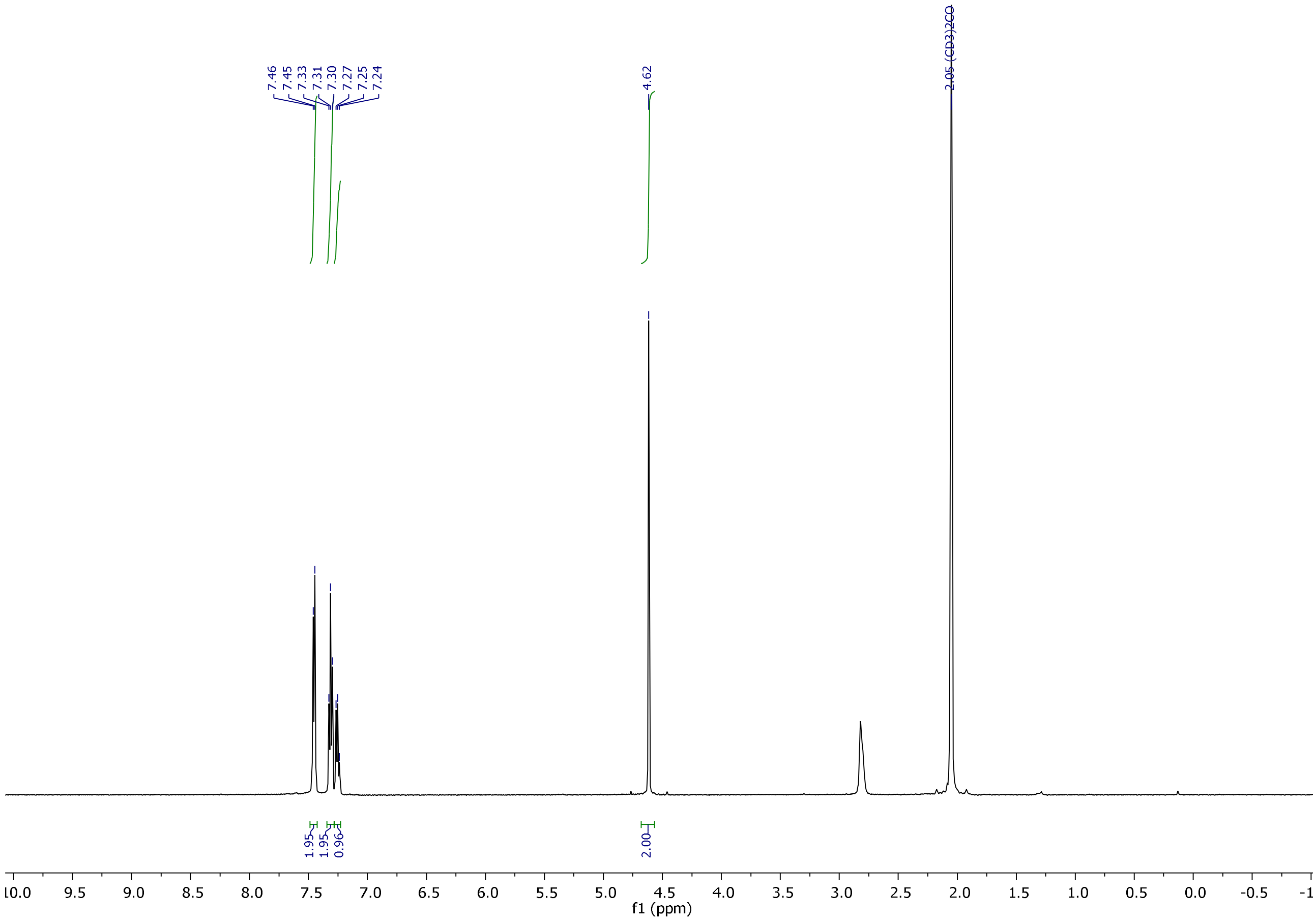

7.46
7.45
7.33
7.31
7.30
7.27
7.25
7.24
4.62
2.05 (CD3)2CO
1.95
1.95
0.96
2.00
10.0
9.5
9.0
8.5
8.0
7.5
7.0
6.5
6.0
5.5
5.0
4.5
4.0
3.5
3.0
2.5
2.0
1.5
1.0
0.5
0.0
-0.5
-1
f1 (ppm)

Ac-Ala-Trp(6-Br)-Gly-NH2 (1)

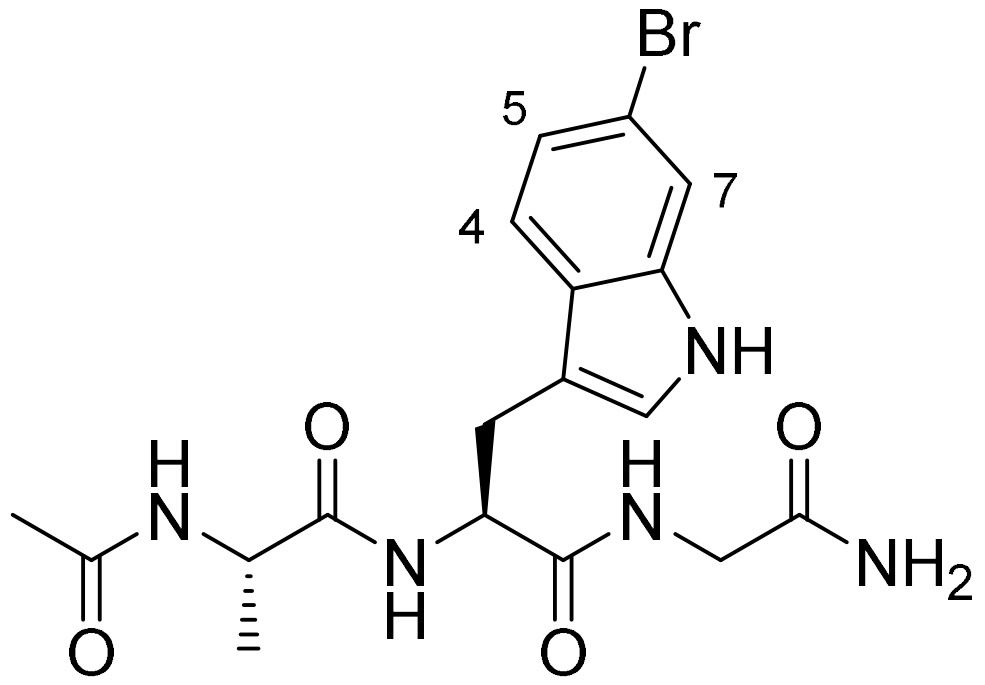

$C_{18}H_{22}BrN_5O_4$
MW = 452,31

Ac-Ala-Trp(6-Br)-Gly-NH_2 was synthesized *via* solid phase peptide synthesis (SPPS) according to GP2. The crude was dissolved in DMF/MeOH 1:1 and purified via preparative RP-HPLC (preparative run protocol 1). Lyophilization provided the product as a colourless solid (71.9 mg, 0.16 mmol, 59%).

^{1}H NMR (600 MHz, [D$_6$]DMSO): δ [ppm] = 10.98 (d, $^3J_{H,H}$ = 1.8 Hz 1H; N**H**-indole), 8.12 (t, $^3J_{H,H}$ = 5.8 Hz, 1H; N**H**^{Gly}), 8.04 (d, $^3J_{H,H}$ = 7.0 Hz, 1H; N**H**^{Ala}), 7.97 (d, $^3J_{H,H}$ = 7.8 Hz, 1H; N**H**^{Trp}), 7.50 (d, $^3J_{H,H}$ = 8.4 Hz, 1H; C4-**H**^{Trp}), 7.49 (d, $^3J_{H,H}$ = 1.7 Hz, 1H; C7-**H**^{Trp}), 7.17 (d, $^3J_{H,H}$ = 1.8 Hz, 1H; C2-**H**^{Trp}), 7.14 (s, 1H; N**H**$_2$), 7.09 (s, 1H; N**H**$_2$), 7.08 (dd, $^3J_{H,H}$ = 8.4 Hz, $^3J_{H,H}$ = 1.7 Hz, 1H; C5-**H**^{Trp}), 4.44 (ddd, $^3J_{H,H}$ = 8.3 Hz, $^3J_{H,H}$ = 7.8 Hz, $^3J_{H,H}$ = 5.0 Hz, 1H; C$_\alpha$-**H**^{Trp}), 4.20 (m, 1H; C$_\alpha$-**H**^{Ala}), 3.66 (dd, $^2J_{H,H}$ = 16.8 Hz, $^3J_{H,H}$ = 5.8 Hz, 1H; C$_\alpha$-**H**^{Gly}), 3.53 (dd, $^2J_{H,H}$ = 16.8 Hz, $^3J_{H,H}$ = 5.8 Hz, 1H; C$_\alpha$-**H**^{Gly}), 3.15 (dd, $^2J_{H,H}$ = 14.8 Hz, $^3J_{H,H}$ = 5.0 Hz, 1H; C$_\beta$-**H**^{Trp}), 2.97 (dd, $^2J_{H,H}$ = 14.8 Hz, $^3J_{H,H}$ = 8.3 Hz, 1H; C$_\beta$-**H**^{Trp}), 1.79 (s, 3H, C**H**$_3$-Ac), 1.11 (d, $^3J_{H,H}$ = 7.1 Hz, 3H; C**H**$_3$^{Ala}).

^{13}C NMR (151 MHz, DMSO): δ [ppm] = 172.6 (**C**=O^{Ala}), 171.3 (**C**=O^{Trp}), 170.8 (**C**=O^{Gly}), 169.4 (CH$_3$**C**=O), 136.9 (**C**7a), 126.4 (**C**3a), 124.8 (**C**2), 121.1 (**C**5), 120.2 (**C**4), 113.8 (**C**6), 113.6 (**C**7), 110.3 (**C**3), 53.5 (**C**$_\alpha$^{Trp}), 48.4 (**C**$_\alpha$^{Ala}), 42.0 (**C**$_\alpha$^{Gly}), 27.0 (**C**$_\beta$^{Trp}), 22.4 (**C**H$_3$C=O), 17.7 (**C**H$_3$^{Ala}).

HRMS (ESI): *m/z* = calcd: 474.0747 [M+Na]$^+$; found: 474.0742 [M+Na]$^+$.

LC-MS chromatogram at λ=220 nm

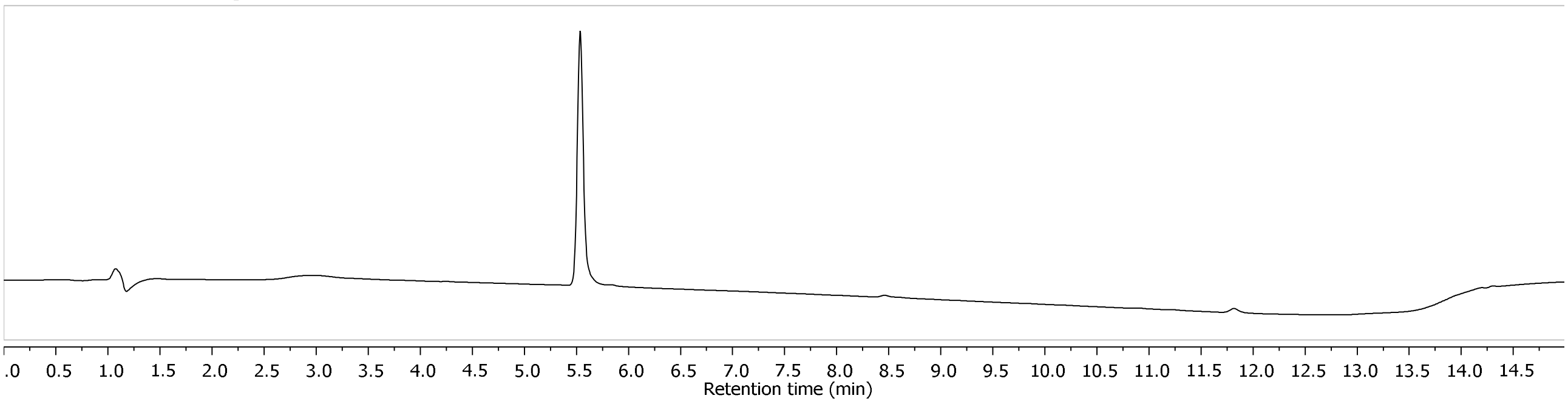

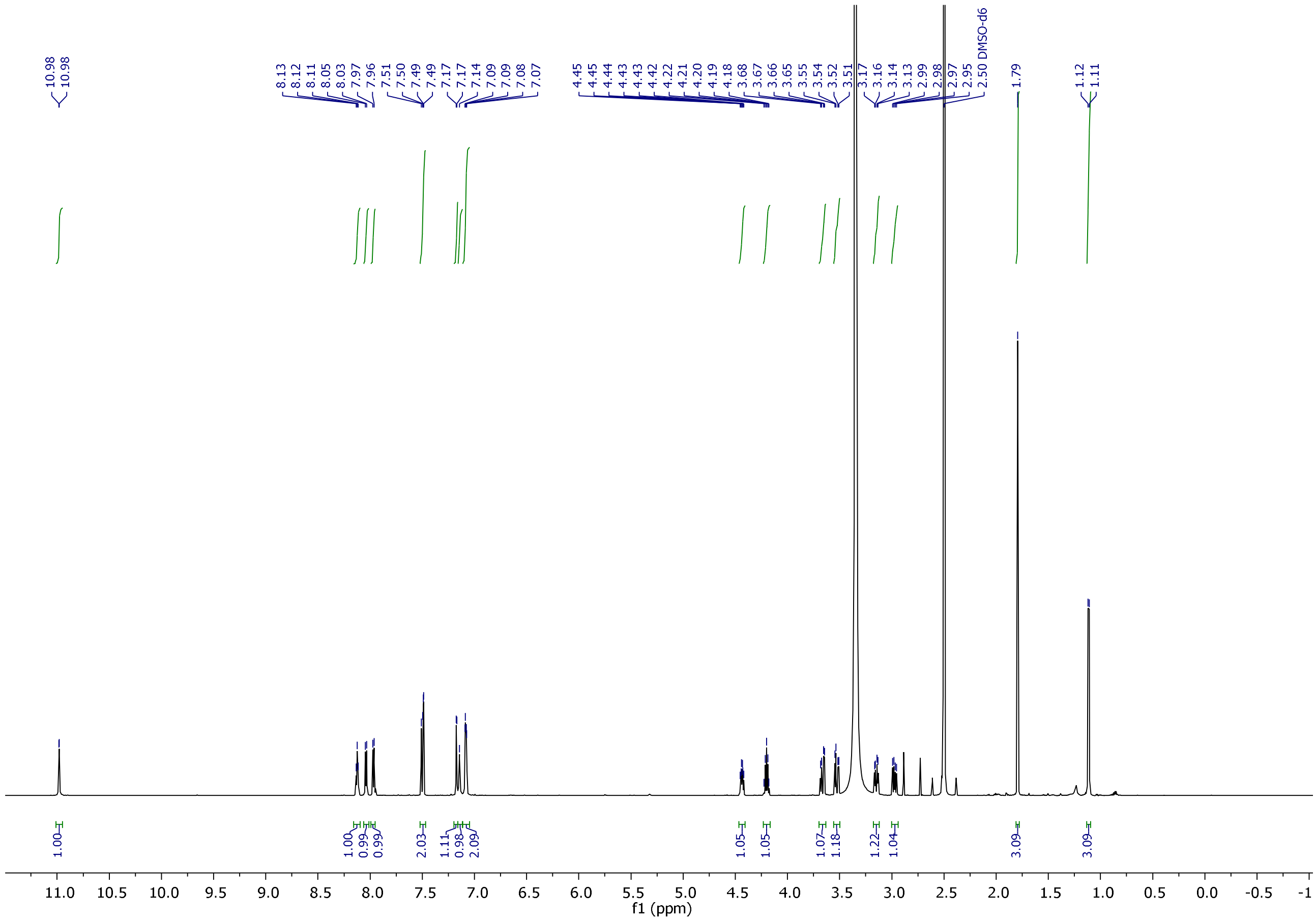

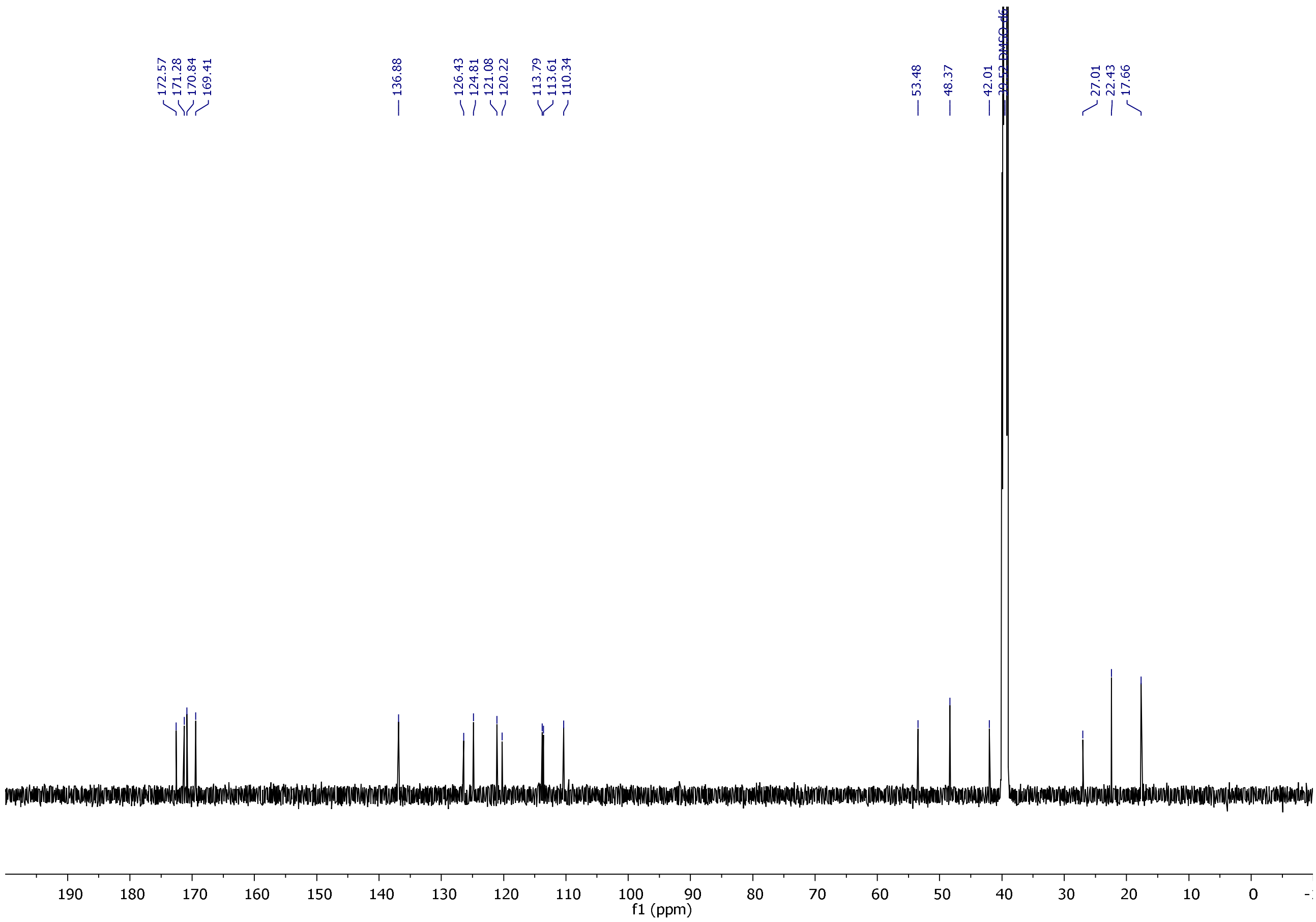

Ac-Leu-Val-Trp(7-Br)-Ala-Gly-NH$_2$ (6)

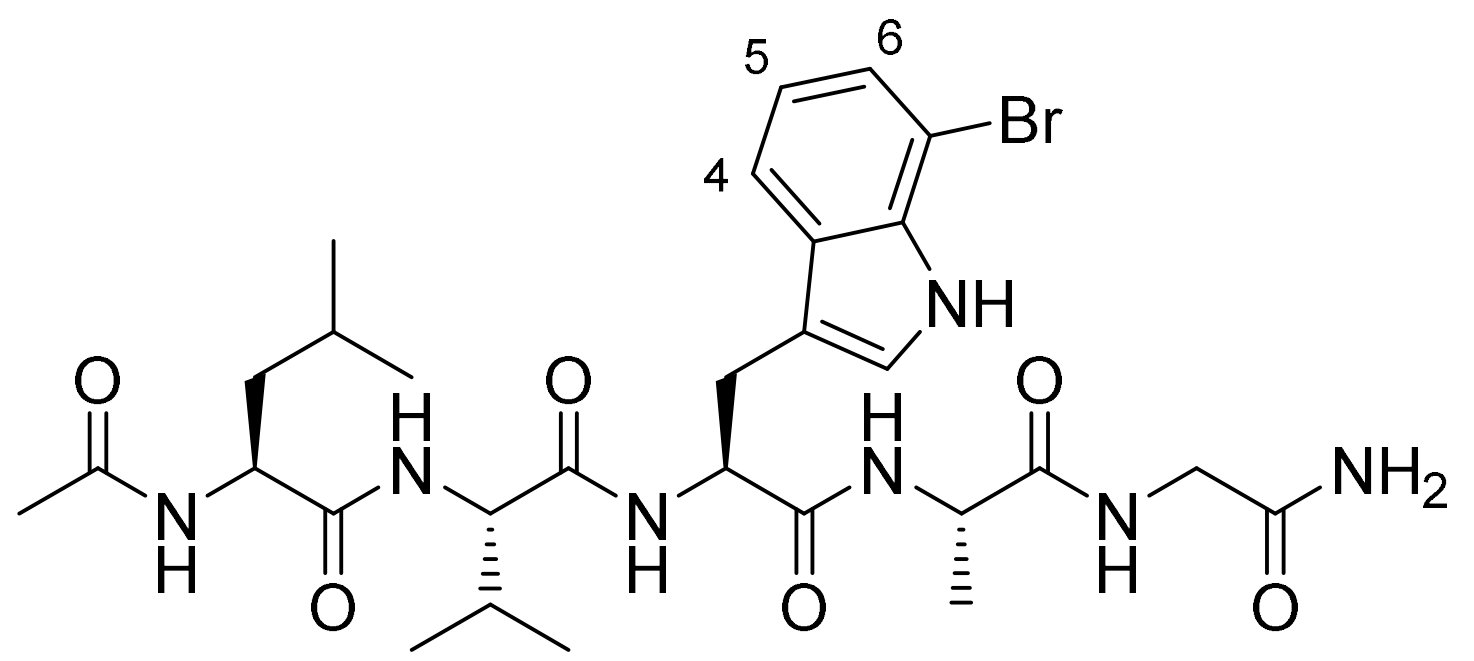

$C_{29}H_{42}BrN_7O_6$
MW = 664,60

Ac-Leu-Val-Trp(7-Br)-Ala-Gly-NH$_2$ was synthesized *via* solid phase peptide synthesis (SPPS) according to GP2. The crude was purified via preparative RP-HPLC (preparative run protocol 2). Lyophilization provided the product as a colourless solid (79.1 mg, 0.12 mmol, 51%).

<u>^{1}H NMR</u> (600 MHz, [D$_6$]DMSO) δ [ppm] = 11.01 (s, 1H; N**H**-indole), 8.12 (d, $^3J_{H,H}$ = 6.9 Hz, 1H; N**H**^{Ala}), 8.02 (m, 2H; N**H**^{Trp}, N**H**^{Leu}), 7.94 (t, $^3J_{H,H}$ = 5.7 Hz, 1H; N**H**^{Gly}), 7.62 (m, 2H; N**H**^{Val}, C4-**H**^{Trp}), 7.27 (d, $^3J_{H,H}$ = 7.7 Hz, 1H; C6-**H**^{Trp}), 7.29 (d, $^3J_{H,H}$ = 2.0 Hz, 1H; C2-**H**^{Trp}), 7.18 (s, 1H; N**H**$_2$), 7.08 (s, 1H; N**H**$_2$), 6.92 (t, $^3J_{H,H}$= 7.7 Hz, 1H; C5-**H**^{Trp}), 4.55 (m, 1H; C$_\alpha$-**H**^{Trp}), 4.28 (m, 1H; C$_\alpha$-**H**^{Leu}), 4.21 (m, 1H; C$_\alpha$-**H**^{Ala}), 4.11 (dd, $^3J_{H,H}$ = 8.2 Hz, $^3J_{H,H}$ = 6.9 Hz, 1H; C$_\alpha$-**H**^{Val}), 3.63 (dd, $^2J_{H,H}$ = 16.8 Hz, $^3J_{H,H}$ = 5.7 Hz, 1H; C$_\alpha$-**H**^{Gly}), 3.57 (dd, $^2J_{H,H}$ = 16.8 Hz, $^3J_{H,H}$ = 5.7 Hz, 1H; C$_\alpha$-**H**^{Gly}), 3.11 (dd, $^2J_{H,H}$ = 14.8 Hz, $^3J_{H,H}$ = 5.2 Hz, 1H; C$_\beta$-**H**^{Trp}), 2.94 (dd, $^2J_{H,H}$ = 14.8 Hz, $^3J_{H,H}$ = 8.5 Hz, 1H; C$_\beta$-**H**^{Trp}), 1.91 (m, 1H; C**H**^{Val}), 1.79 (s, 3H; C**H**$_3$-Ac), 1.56 (m, 1H; C**H**^{Leu}), 1.39 (m, 2H; C-**H**$_2$^{Leu}), 1.18 (d, $^3J_{H,H}$ = 7.1 Hz, 3H; C**H**$_3$^{Ala}), 0.85 (d, $^3J_{H,H}$ = 6.6 Hz, 3H; C**H**$_3$^{Leu}), 0.80 (d, $^3J_{H,H}$ = 6.6 Hz, 3H; C**H**$_3$^{Leu}) 0.75 (d, $^3J_{H,H}$ = 6.8 Hz, 6H; 2C**H**$_3$^{Val}).

<u>^{13}C NMR</u> (151 MHz, DMSO): δ [ppm] = 172.1 (**C**=O^{Ala}), 172.0 (**C**=O^{Leu}), 171.1 (**C**=O^{Trp}), 171.0 (**C**=O^{Val}, **C**=O^{Gly}), 169.3 (CH$_3$**C**=O), 134.3 (**C**7a), 129.1 (**C**3a), 125.1 (**C**2), 123.4 (**C**6), 119.7 (**C**5), 118.1 (**C**4), 111.4 (**C**3), 104.1 (**C**7), 57.5 (**C**$_\alpha$^{Val}), 53.1 (**C**$_\alpha$^{Trp}), 51.1 (**C**$_\alpha$^{Leu}), 48.6 (**C**$_\alpha$^{Ala}), 41.9 (**C**$_\alpha$^{Gly}), 40.3 (**C**$_\beta$^{Leu}), 30.7 (**C**$_\beta$^{Val}), 27.4 (**C**$_\beta$^{Trp}), 24.2 (**C**$_\gamma$^{Leu}), 23.1 (**C**H$_3$^{Leu}), 22.5 (**C**H$_3$C=O), 21.6 (**C**H$_3$^{Leu}), 19.1 (**C**H$_3$^{Val}), 18.0 (**C**H$_3$^{Val}), 17.8 (**C**H$_3$^{Ala}).

<u>HRMS</u> (ESI): *m/z* calcd: 686.2272 [M+Na]$^+$; found: 686.2276 [M+Na]$^+$.

LC-MS chromatogram at λ=220 nm

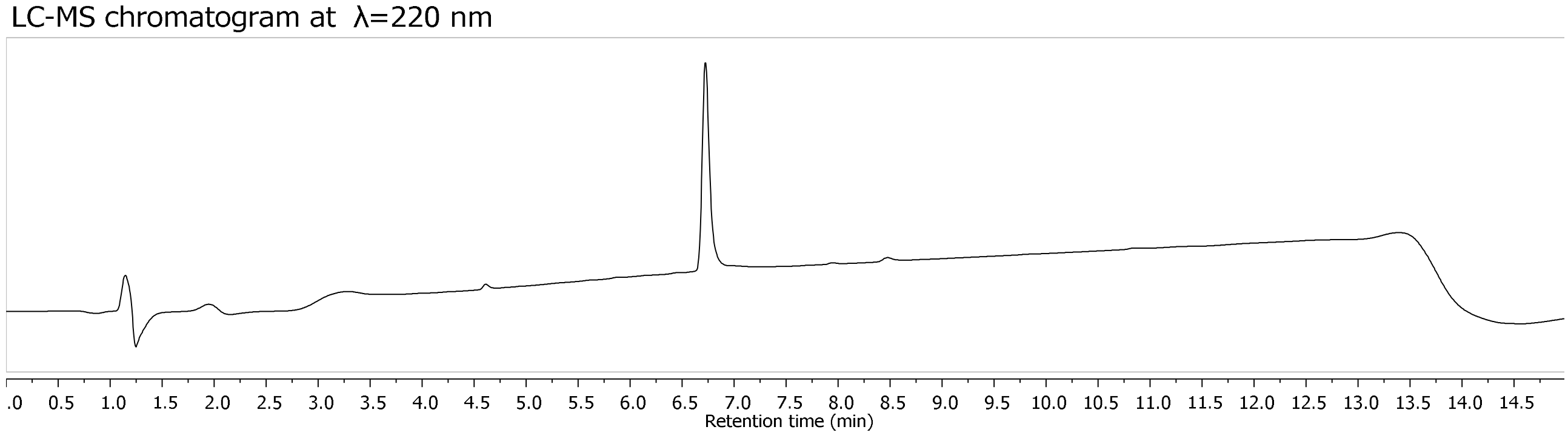

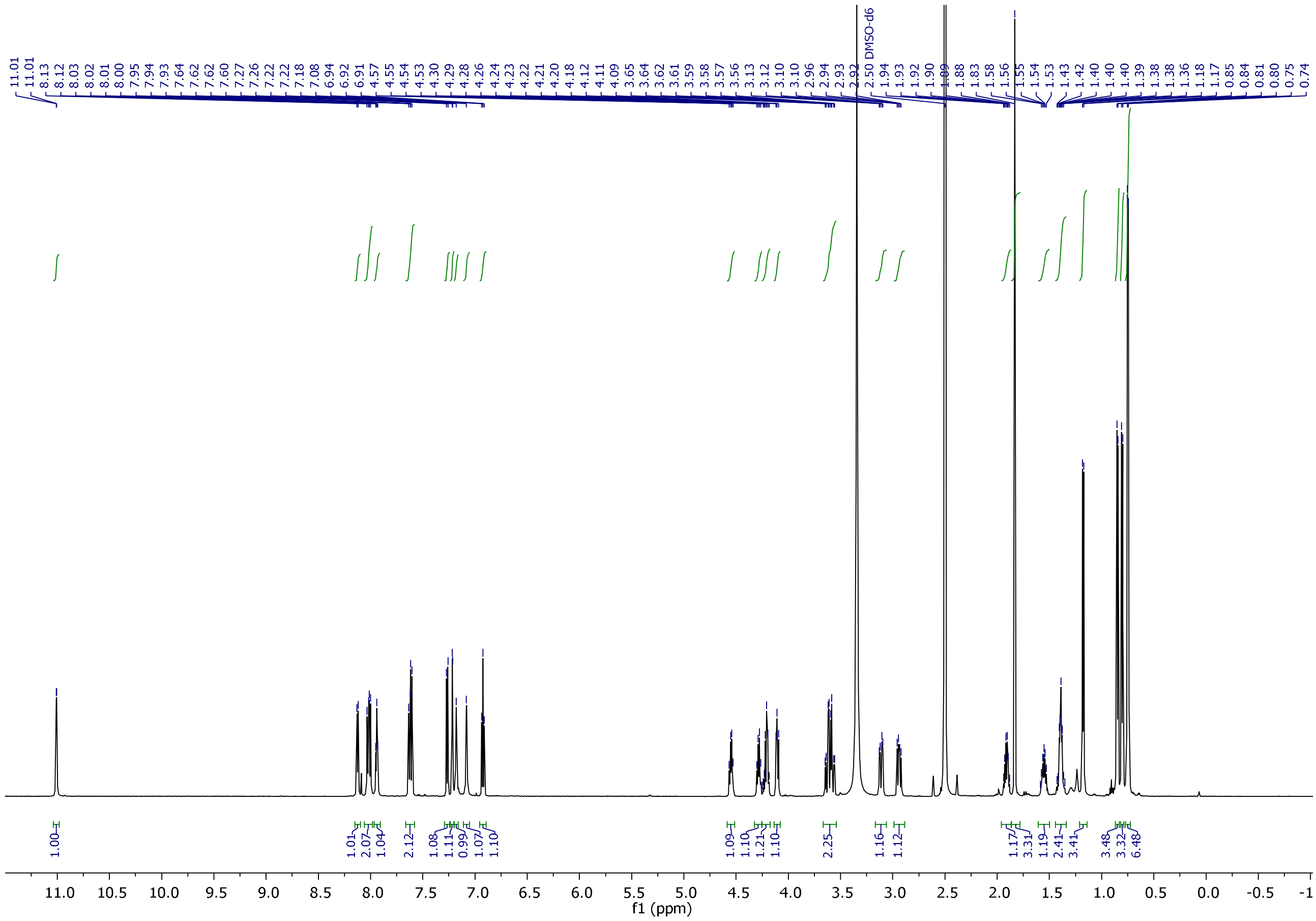

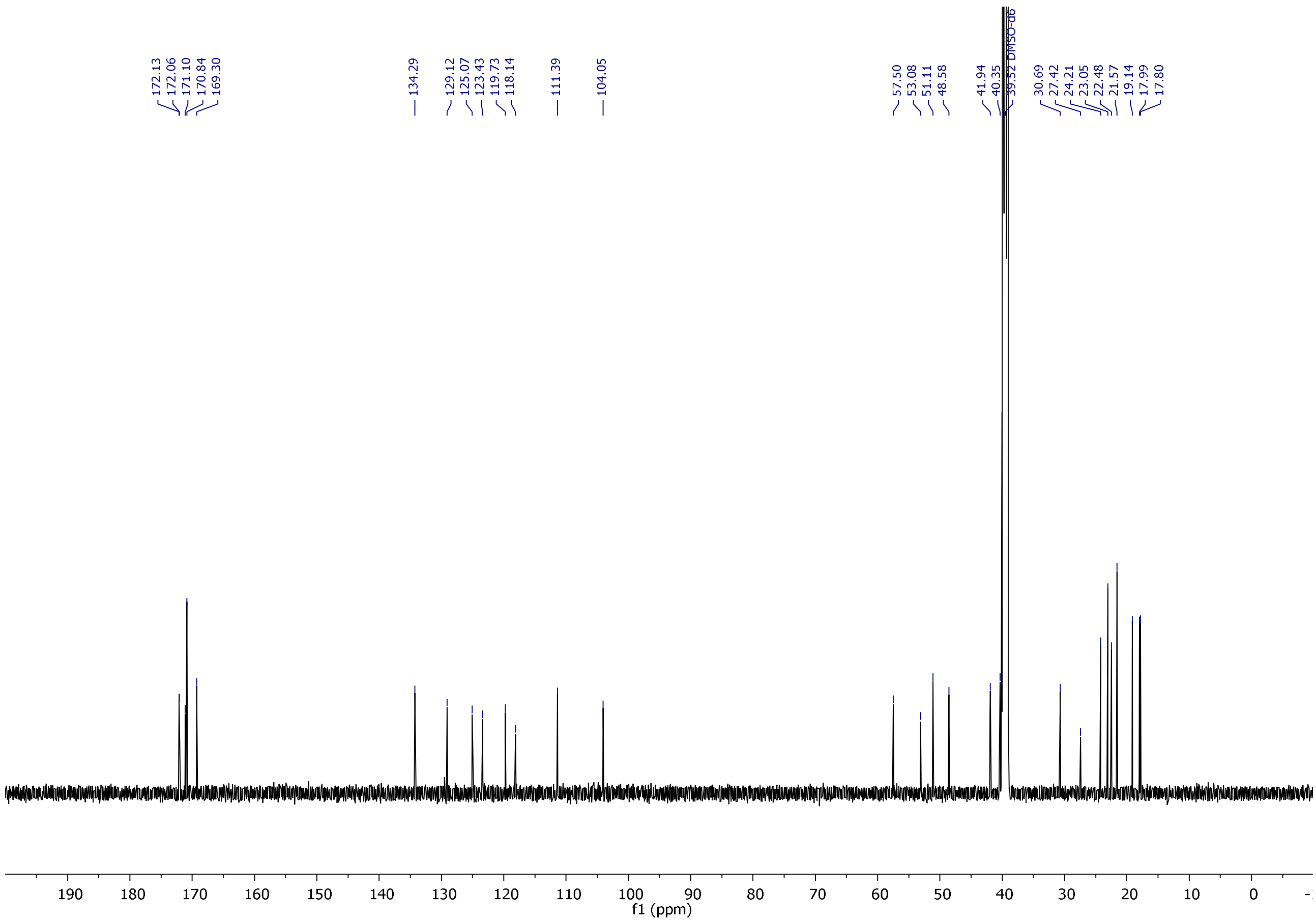

Ac-Asp-Val-Trp(7-Br)-Ala-Gly-NH₂ (7)

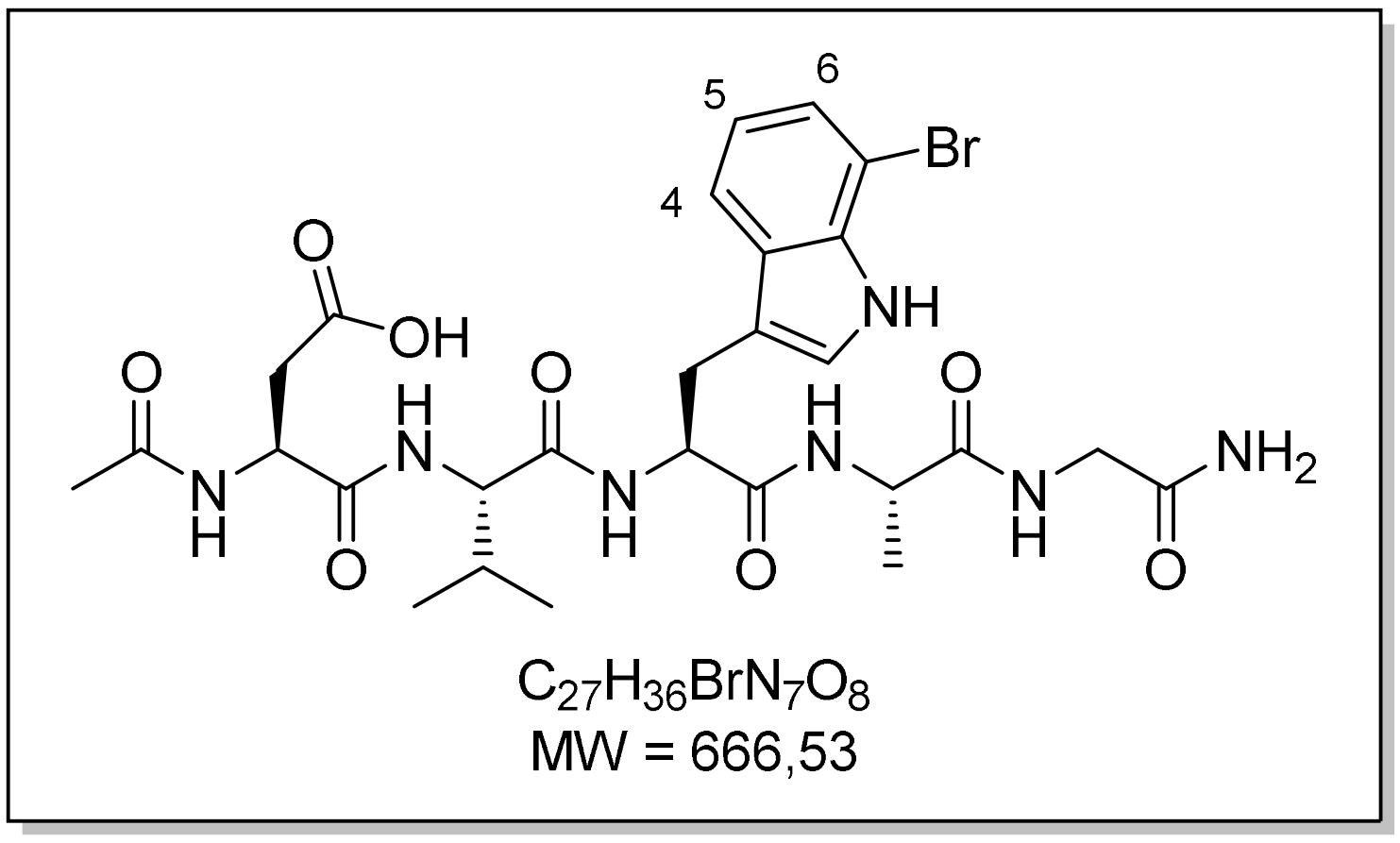

Ac-Asp-Val-Trp(7-Br)-Ala-Gly-NH_2 was synthesized *via* solid phase peptide synthesis (SPPS) according to GP2. The crude was purified via preparative RP-HPLC (preparative run protocol 2). Lyophilization provided the product as a colourless solid (69.9 mg, 0.10 mmol, 72%).

^{1}H NMR (600 MHz, [D_6]DMSO): δ [ppm] = 12.31 (s br, 1H; COO**H**^{Asp}), 11.01 (s, 1H; N**H**-indole), 8.26 (d, $^3J_{H-H}$ = 7.9 Hz, 1H; N**H**^{Asp}), 8.09 (d, $^3J_{H-H}$ = 6.9 Hz, 1H; N**H**^{Ala}), 8.03 (d, $^3J_{H-H}$ = 7.9 Hz, 1H; N**H**^{Trp}), 7.93 (t, $^3J_{H-H}$ = 5.8 Hz, 1H; N**H**^{Gly}), 7.62 (d, $^3J_{H-H}$ = 7.7 Hz, 1H; C4-**H**^{Trp}), 7.58 (d, $^3J_{H-H}$ = 8.6 Hz, 1H; N**H**^{Val}), 7.27 (d, $^3J_{H-H}$ = 7.7 Hz, 1H; C6-**H**^{Trp}), 7.21 (d, $^3J_{H-H}$ = 2.0 Hz, 1H; C2-**H**^{Trp}), 7.17 (s, 1H; N**H**$_2$), 7.08 (s, 1H; N**H**$_2$), 6.93 (t, $^3J_{H-H}$ = 7.7 Hz, 1H; C5-**H**^{Trp}), 4.59 (m, 1H; C_α-**H**^{Asp}), 4.54 (m, 1H; C_α-**H**^{Trp}), 4.21 (m, 1H; C_α-**H**^{Ala}), 4.10 (dd, $^3J_{H-H}$ = 8.6 Hz, $^3J_{H-H}$ = 6.1 Hz, 1H; C_α-**H**^{Val}), 3.63 (dd, $^2J_{H-H}$ = 16.8 Hz, $^3J_{H-H}$ = 5.8 Hz, 1H; C_α-**H**^{Gly}), 3.58 (dd, $^2J_{H-H}$ = 16.8 Hz, $^3J_{H-H}$ = 5.8 Hz, 1H; C_α-**H**^{Gly}), 3.14 (dd, $^2J_{H-H}$ = 14.8 Hz, $^3J_{H-H}$ = 5.0 Hz, 1H; C_β-**H**^{Trp}), 2.94 (dd, $^2J_{H-H}$ = 14.8 Hz, $^3J_{H-H}$ = 9.0 Hz, 1H; C_β-**H**^{Trp}), 2.67 (dd, $^2J_{H-H}$ = 16.6 Hz, $^3J_{H-H}$ = 5.8 Hz, 1H; C_β-**H**^{Asp}), 2.47 (m, 1H; C_β-**H**^{Asp}), 1.91 (m, 1H; C**H**^{Val}), 1.83 (s, 3H; C**H**$_3$-Ac), 1.19 (d, $^3J_{H-H}$ = 7.1 Hz, 3H; C**H3**^{Ala}), 0.72 (d, $^3J_{H-H}$ = 6.8 Hz, 3H; C**H3**^{Val}), 0.69 (d, $^3J_{H-H}$ = 6.8 Hz, 3H; C**H3**^{Val}).

^{13}C NMR (151 MHz, DMSO): δ [ppm] = 172.2 (**C**=O^{Ala}), 172.0 (**C**OOH^{Asp}) 171.2 (**C**=O^{Trp}), 170.9 (**C**=O^{Gly}), 170.8 (**C**=O^{Asp}), 170.6 (**C**=O^{Val}), 169.6 (CH_3**C**=O), 134.3 (**C**7a), 129.1 (**C**3a), 125.1 (**C**2), 123.5 (**C**6), 119.8 (**C**5), 118.2 (**C**4), 111.5 (**C**3), 104.1 (**C**7), 57.6 (**C$_\alpha$**^{Val}), 53.1 (**C$_\alpha$**^{Trp}), 49.5 (**C$_\alpha$**^{Asp}), 48.7 (**C$_\alpha$**^{Ala}), 42.0 (**C$_\alpha$**^{Gly}), 35.6 (**C$_\beta$**^{Asp}), 30.7 (**C$_\beta$**^{Val}), 27.3 (**C$_\beta$**^{Trp}), 22.5 (**C**H_3C=O), 19.1 (**C**H_3^{Val}), 17.8 (**C**H_3^{Ala}), 17.6 (**C**H_3^{Val}).

HRMS (ESI): *m/z* calcd 666.1882 [M+H]$^+$, 688.1701 [M+Na]$^+$; found 666.1885 [M+H]$^+$, 688.1708 [M+Na]$^+$.

LC-MS chromatogram at λ=220 nm

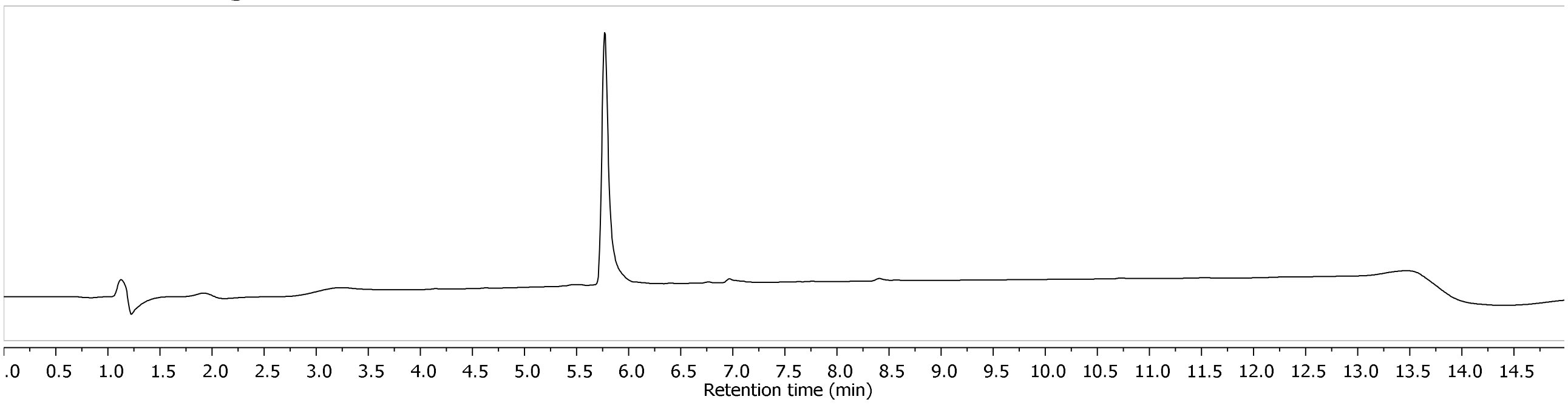

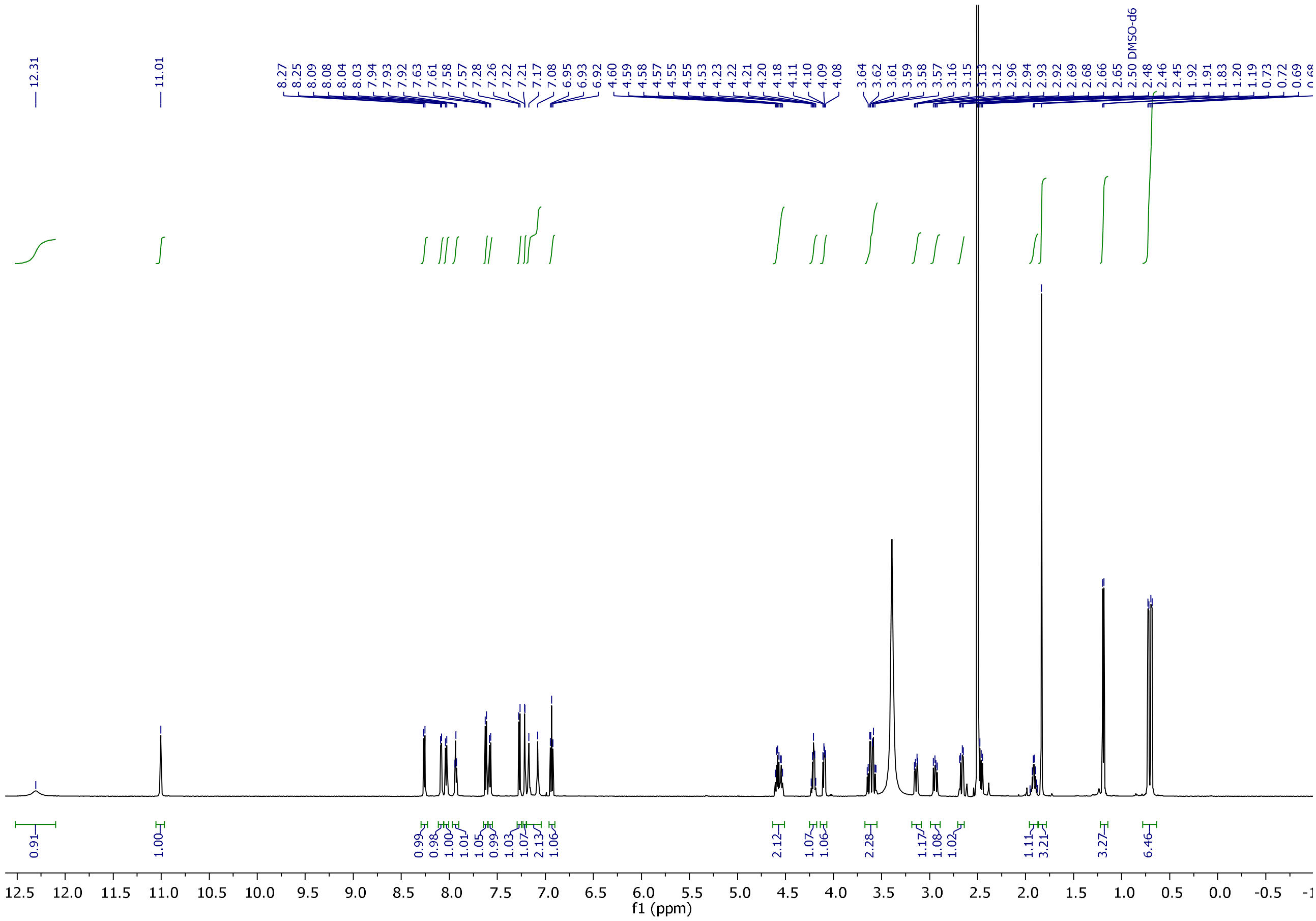
12.31
11.01
8.27 8.25 8.09 8.08 8.04 8.03 7.94 7.93 7.92 7.63 7.61 7.58 7.57 7.28 7.26 7.22 7.21 7.17 7.08 6.95 6.93 6.92
4.60 4.59 4.58 4.57 4.55 4.55 4.53 4.23 4.22 4.21 4.20 4.18 4.11 4.10 4.09 4.08
3.64 3.62 3.61 3.59 3.58 3.57 3.16 3.15 3.13 3.12 2.96 2.94 2.93 2.92 2.69 2.68 2.66 2.65 2.50 DMSO-d6 2.48 2.46 2.45 1.92 1.91 1.83 1.20 1.19 0.73 0.72 0.69
0.91
1.00
0.99 0.98 1.00 1.01 1.05 0.99 1.03 1.07 2.13 1.06
2.12 1.07 1.06 2.28 1.17 1.08 1.02 1.11 3.21 3.27 6.46
f1 (ppm)

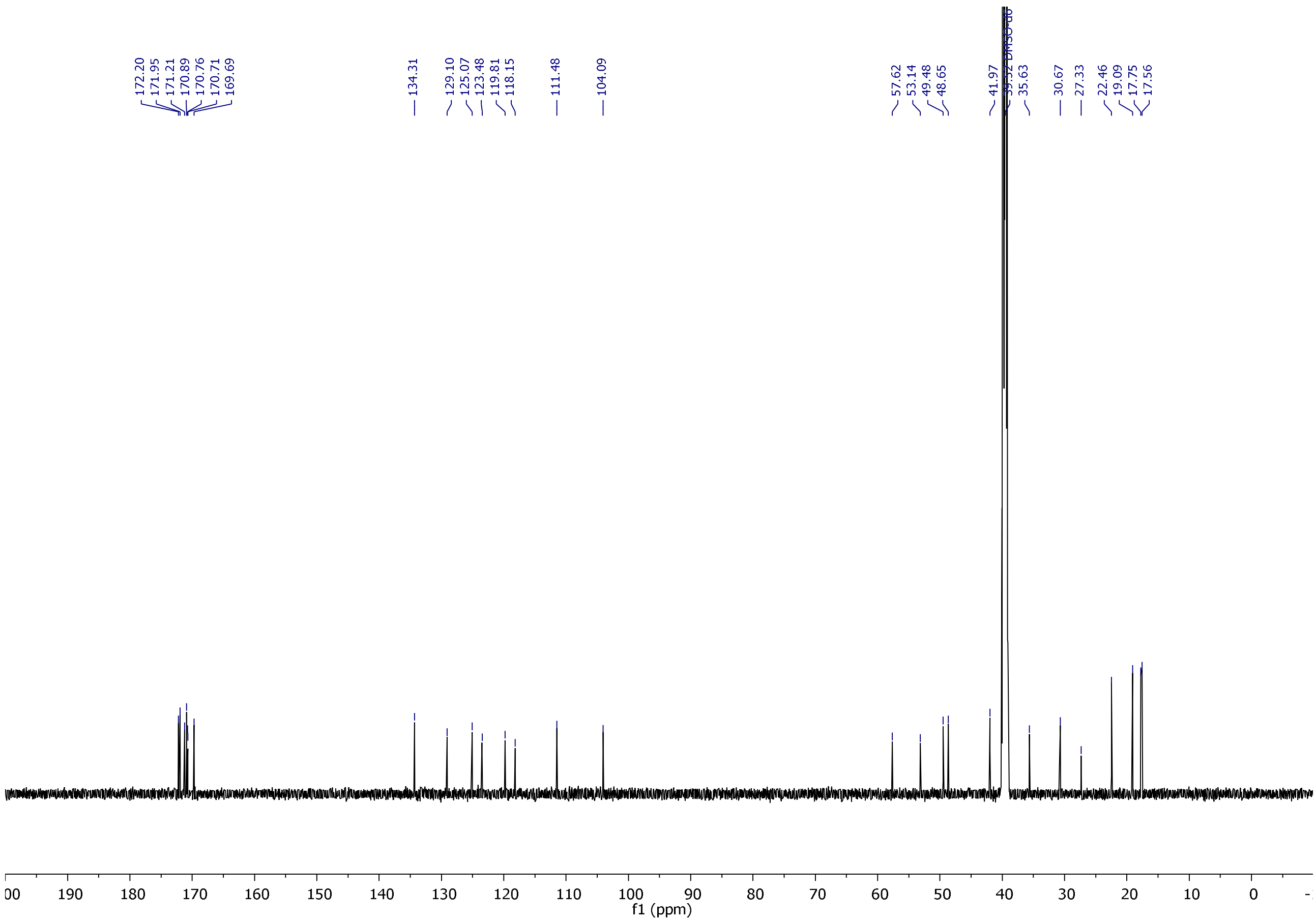
172.20 171.95 171.21 170.89 170.76 170.71 169.69
134.31
129.10 125.07 123.48 119.81 118.15
111.48
104.09
57.62 53.14 49.48 48.65
41.97 39.52 DMSO-d6 35.63
30.67 27.33 22.46 19.09 17.75 17.56
f1 (ppm)

Ac-Ser-Val-Trp(7-Br)-Ala-Gly-NH_2 (8)

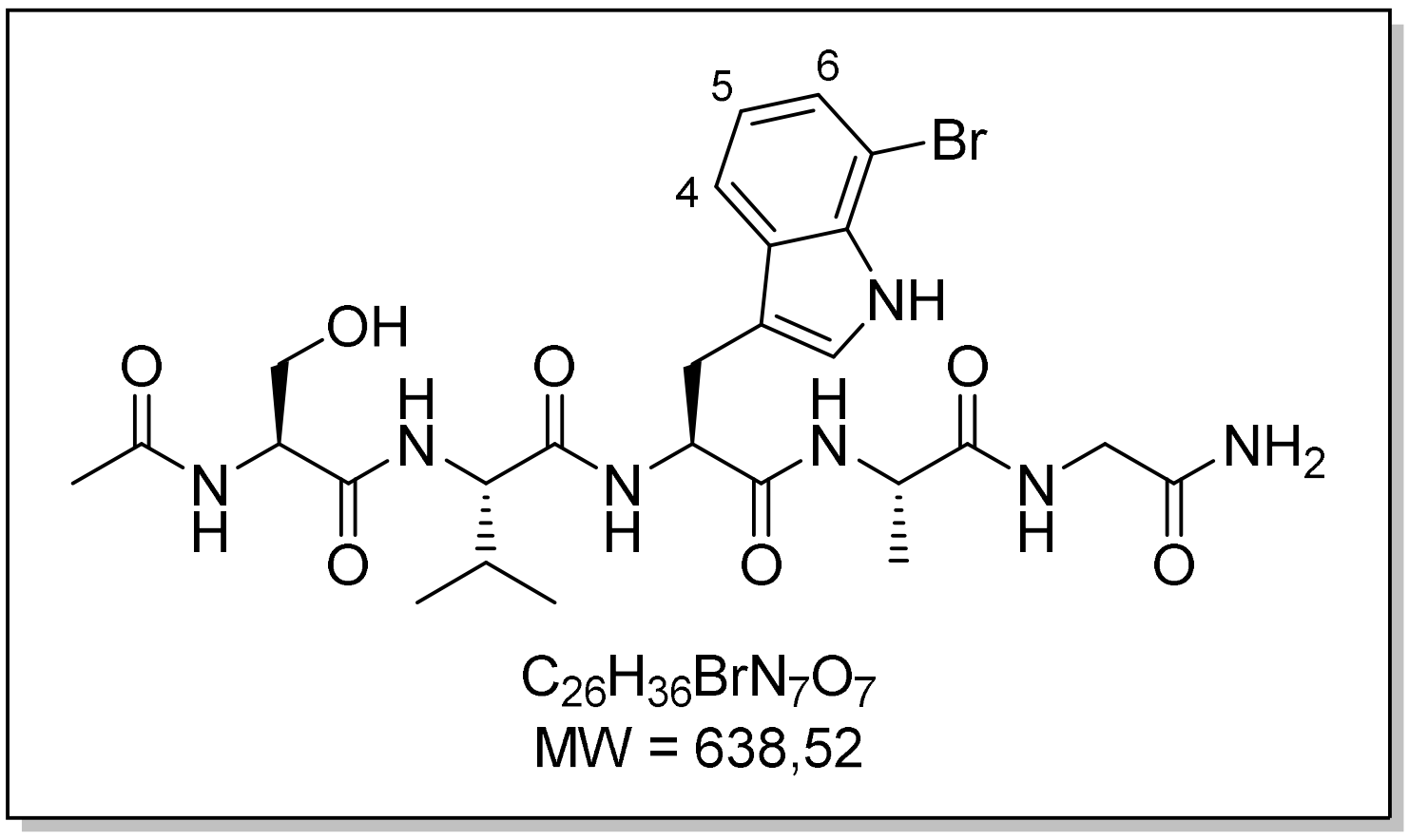

Ac-Ser-Val-Trp(7-Br)-Ala-Gly-NH_2 was synthesized *via* solid phase peptide synthesis (SPPS) according to GP2. The crude was purified via preparative RP-HPLC (preparative run protocol 2). Lyophilization provided the product as a colourless solid (59.3 mg, 92.9 µmol, 50%).

^{1}H NMR (600 MHz, [D_6]DMSO) δ [ppm] = 11.01 (s, 1H; N**H**-indole), 8.07 (d, $^3J_{H\text{-}H}$ = 6.8 Hz, 1H; N**H**^{Ala}), 8.01 (d, $^3J_{H\text{-}H}$ = 8.1 Hz, 1H; N**H**^{Trp}), 7.97 (d, $^3J_{H\text{-}H}$ = 7.8 Hz, 1H; N**H**^{Ser}), 7.93 (t, $^3J_{H\text{-}H}$ = 5.8 Hz, 1H; N**H**^{Gly}), 7.73 (d, $^3J_{H\text{-}H}$ = 8.3 Hz, 1H; N**H**^{Val}), 7.61 (d, $^3J_{H\text{-}H}$ = 7.7 Hz, 1H; C4-**H**^{Trp}), 7.27 (d, $^3J_{H\text{-}H}$ = 7.7 Hz, 1H; C6-**H**^{Trp}), 7.21 (d, $^3J_{H\text{-}H}$ = 2.0 Hz, 1H; C2-**H**^{Trp}), 7.18 (s, 1H; N**H**$_2$), 7.08 (s, 1H; N**H**$_2$), 6.94 (t, $^3J_{H\text{-}H}$ = 7.7 Hz, 1H; C5-**H**^{Trp}), 5.06 (s br, 1H; O**H**^{Ser}), 4.54 (ddd, $^3J_{H\text{-}H}$ = 8.8 Hz, $^3J_{H\text{-}H}$ = 8.1 Hz, $^3J_{H\text{-}H}$ = 5.1 Hz, 1H; C_α-**H**^{Trp}), 4.38 (m, 1H, C_α-**H**^{Ser}), 4.21 (m, 1H; C_α-**H**^{Ala}), 4.11 (dd, $^3J_{H\text{-}H}$ = 8.3 Hz, $^3J_{H\text{-}H}$ = 6.0 Hz, 1H; C_α-**H**^{Val}), 3.64 (dd, $^2J_{H\text{-}H}$ = 16.8 Hz, $^3J_{H\text{-}H}$ = 5.8 Hz, 1H; C_α-**H**^{Gly}), 3.61-3.51 (m, 3H; C_α-**H**^{Gly}, C_β-**H**$_2^{Ser}$), 3.14 (dd, $^2J_{H\text{-}H}$ = 14.9 Hz, $^3J_{H\text{-}H}$ = 5.1 Hz, 1H; C_β-**H**^{Trp}), 2.91 (dd, $^2J_{H\text{-}H}$ = 14.9 Hz, $^3J_{H\text{-}H}$ = 8.8 Hz, 1H; C_β-**H**^{Trp}), 1.93 (m, 1H; C**H**^{Val}), 1.86 (s, 3H; C**H**$_3$-Ac), 1.20 (d, $^3J_{H\text{-}H}$ = 7.1 Hz, 3H; C**H**$_3^{Ala}$), 0.74 (d, $^3J_{H\text{-}H}$ = 6.8 Hz, 3H; C**H**$_3^{Val}$), 0.69 (d, $^3J_{H\text{-}H}$ = 6.8 Hz, 3H; C**H**$_3^{Val}$).

^{13}C NMR (151 MHz, DMSO): δ [ppm] = 172.2 (**C**=O^{Ala}), 171.2 (**C**=O^{Trp}), 170.9 (**C**=O^{Val}), 170.8 (**C**=O^{Gly}), 170.6 (**C**=O^{Ser}), 169.6 (CH_3**C**=O), 134.3 (**C**7a), 129.1 (**C**3a), 125.0 (**C**2), 123.5 (**C**6), 119.8 (**C**5), 118.1 (**C**4), 111.4 (**C**3), 104.1 (**C**7), 61.7 (**C**$_\beta^{Ser}$), 57.9 (**C**$_\alpha^{Val}$), 54.8 (**C**$_\alpha^{Ser}$), 53.1 (**C**$_\alpha^{Trp}$), 48.7 (**C**$_\alpha^{Ala}$), 42.0 (**C**$_\alpha^{Gly}$), 30.3 (**C**$_\beta^{Val}$), 27.4 (**C**$_\beta^{Trp}$), 22.6 (**C**H_3C=O), 19.1 (**C**H_3^{Val}), 17.7 (**C**H_3^{Ala}), 17.6 (**C**H_3^{Val}).

HRMS (ESI): *m/z* calcd: 660.1752 $[M+Na]^+$; found: 660.1752 $[M+Na]^+$.

LC-MS chromatogram at λ=220 nm

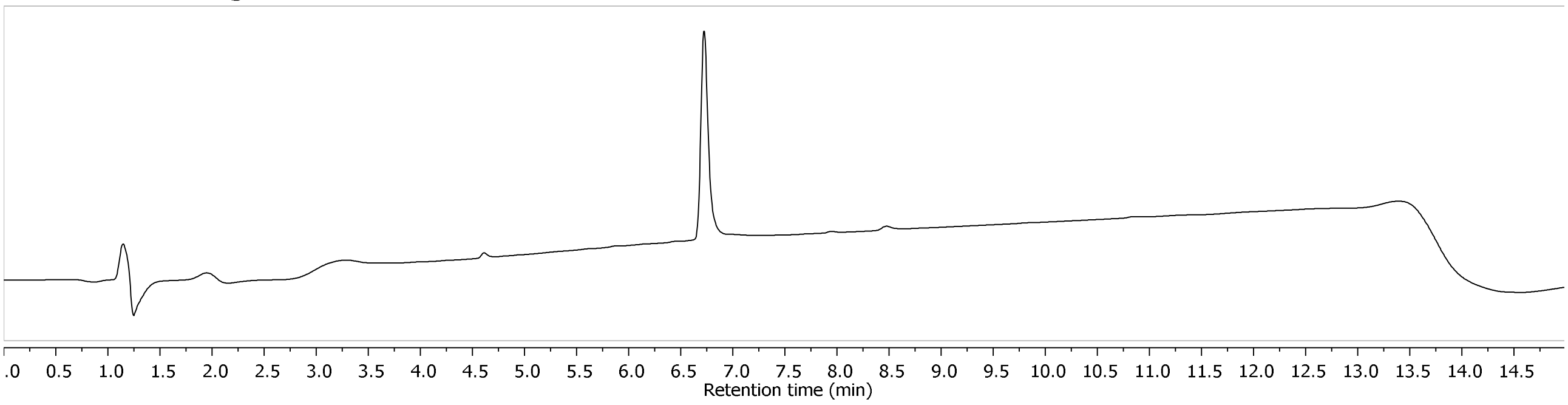

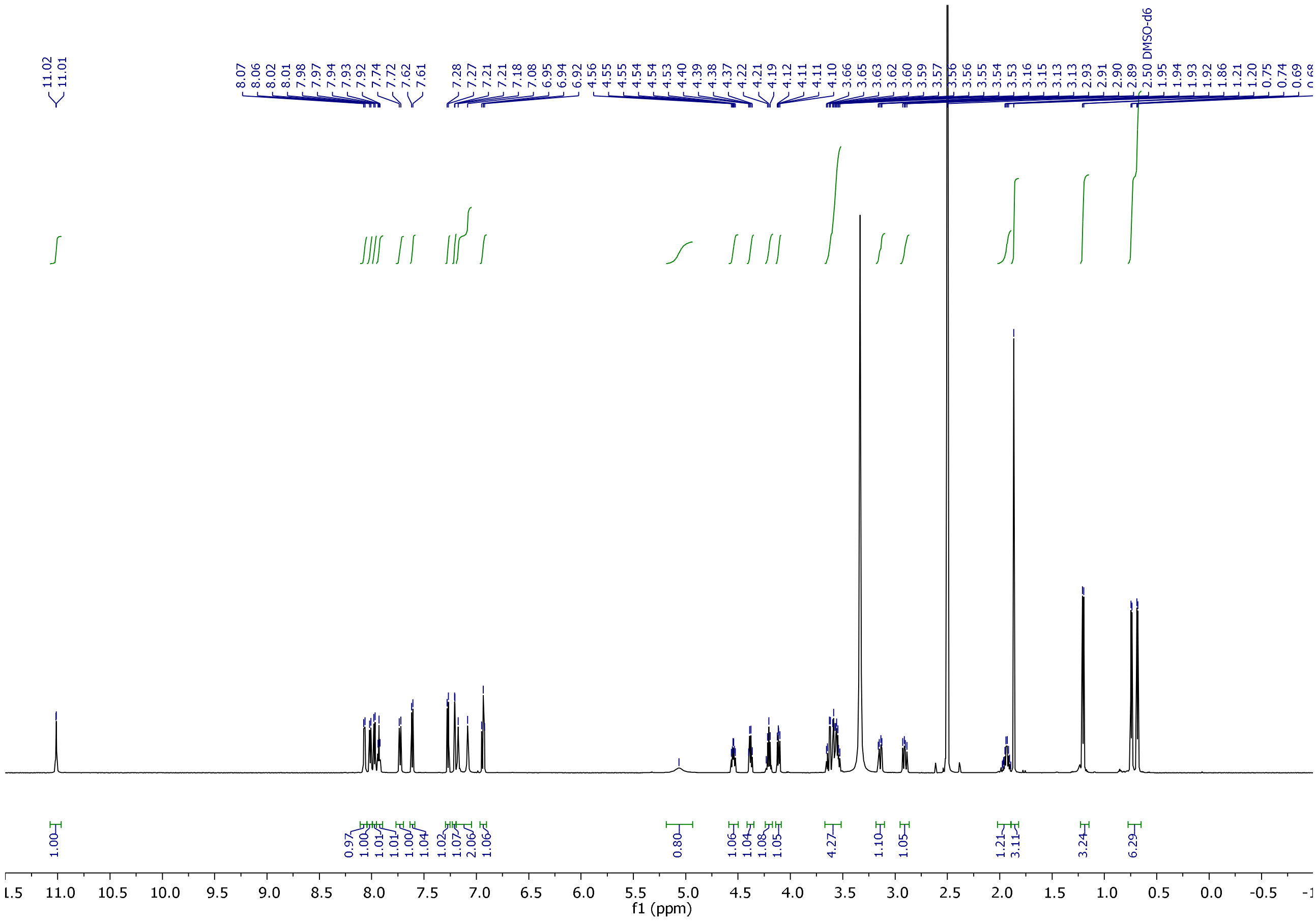
11.02
11.01
f1 (ppm)
2.50 DMSO-d6

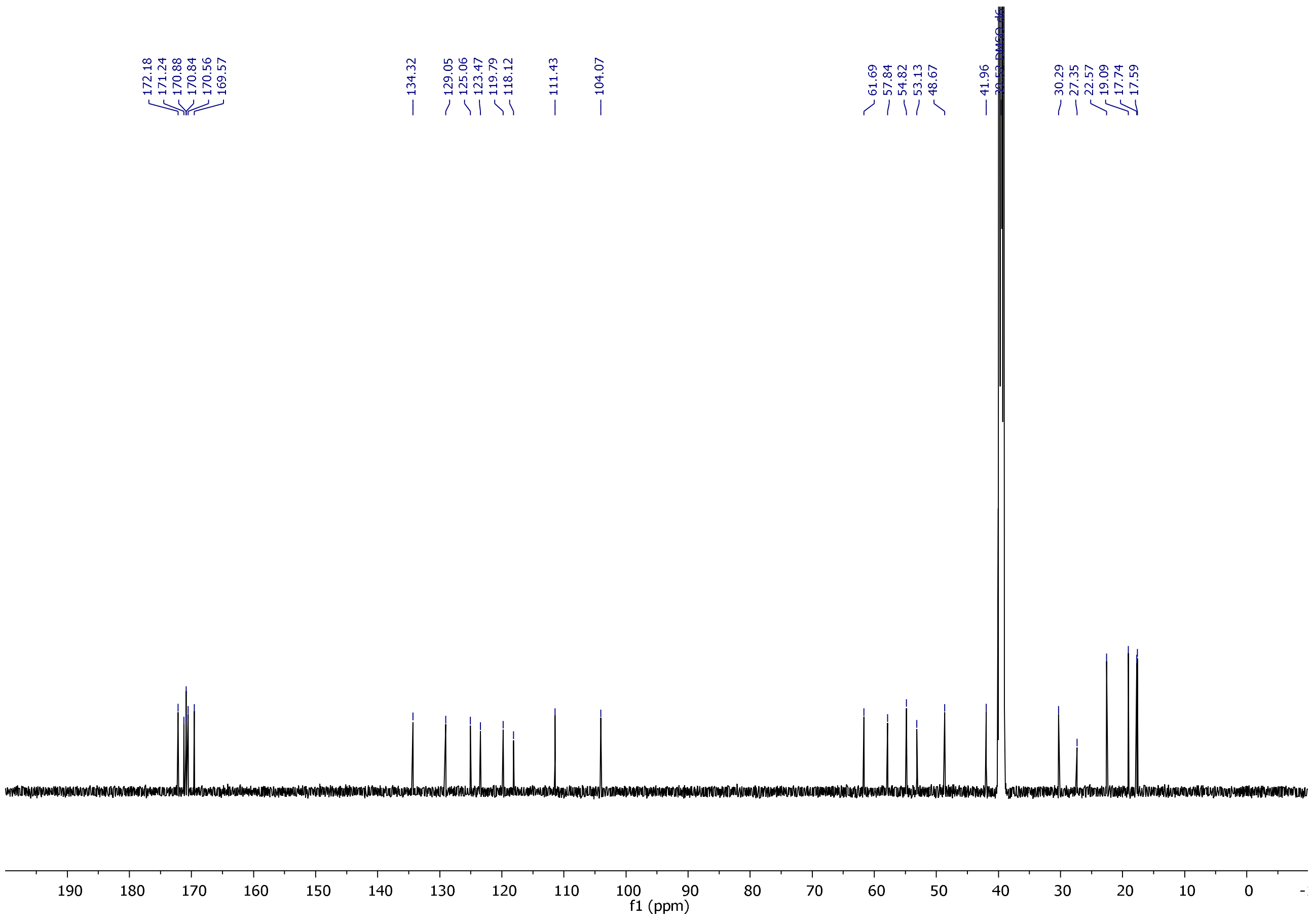
172.18
171.24
170.88
170.84
170.56
169.57
134.32
129.05
125.06
123.47
119.79
118.12
111.43
104.07
61.69
57.84
54.82
53.13
48.67
41.96
30.29
27.35
22.57
19.09
17.74
17.59
f1 (ppm)

Ac-Met-Val-Trp(7-Br)-Ala-Gly-NH2 (9)

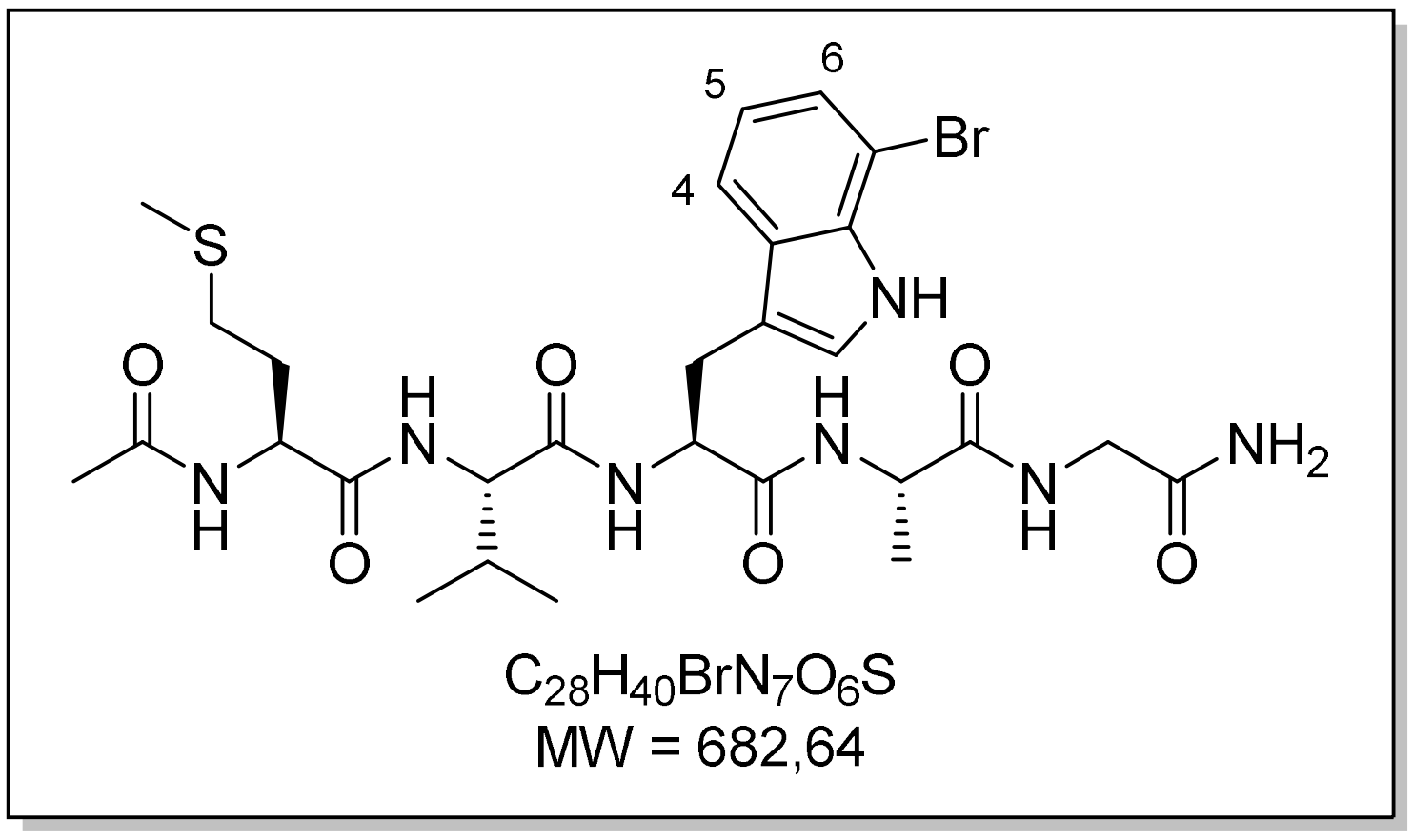

Ac-Met-Val-Trp(7-Br)-Ala-Gly-NH_2 was synthesized *via* solid phase peptide synthesis (SPPS) according to GP2. The crude was purified via preparative RP-HPLC (preparative run protocol 2). Lyophilization provided the product as a colourless solid (48.6 mg, 71.2 µmol, 56%).

^{1}H NMR (600 MHz, [D$_6$]DMSO) δ [ppm] = 11.01 (s, 1H; N**H**-indole), 8.14 (d, $^3J_{H\text{-}H}$ = 6.9 Hz, 1H; N**H**^{Ala}), 8.06 (m, 2H; N**H**^{Trp}, N**H**^{Met}), 7.93 (t, $^3J_{H\text{-}H}$ = 5.8 Hz, 1H; N**H**^{Gly}), 7.68 (d, $^3J_{H\text{-}H}$ = 8.6 Hz, 1H; N**H**^{Val}), 7.61 (d, $^3J_{H\text{-}H}$ = 7.7 Hz, 1H; C4-**H**^{Trp}), 7.27 (d, $^3J_{H\text{-}H}$ = 7.7 Hz, 1H; C6-**H**^{Trp}), 7.22 (d, $^3J_{H\text{-}H}$ = 1.9 Hz, 1H; C2-**H**^{Trp}), 7.18 (s, 1H; N**H**$_2$), 7.08 (s, 1H; N**H**$_2$), 6.93 (t, $^3J_{H\text{-}H}$ = 7.7 Hz, 1H; C5-**H**^{Trp}), 4.55 (m, 1H; C$_\alpha$-**H**^{Trp}), 4.34 (ddd, $^3J_{H\text{-}H}$ = 8.3 Hz, $^3J_{H\text{-}H}$ = 8.3 Hz, ^3J = 5.4 Hz, 1H; C$_\alpha$-**H**^{Met}), 4.21 (m, 1H; C$_\alpha$-**H**^{Ala}), 4.12 (dd, $^3J_{H\text{-}H}$ = 8.6 Hz, $^3J_{H\text{-}H}$ = 6.8 Hz, 1H; C$_\alpha$-**H**^{Val}), 3.63 (dd, $^2J_{H\text{-}H}$ = 16.8 Hz, $^3J_{H\text{-}H}$ = 5.8 Hz, 1H; C$_\alpha$-**H**^{Gly}), 3.58 (dd, $^2J_{H\text{-}H}$ = 16.8 Hz, $^3J_{H\text{-}H}$ = 5.8 Hz, 1H; C$_\alpha$-**H**^{Gly}), 3.12 (dd, $^2J_{H\text{-}H}$ = 14.8 Hz, $^3J_{H\text{-}H}$ = 5.3 Hz, 1H; C$_\beta$-**H**^{Trp}), 2.94 (dd, $^2J_{H\text{-}H}$ = 14.8 Hz, $^3J_{H\text{-}H}$ = 8.4 Hz, 1H; C$_\beta$-**H**^{Trp}), 2.40 (m, 2H; C$_\gamma$-**H**$_2^{Met}$), 1.99 (s, 3H; C**H**$_3^{Met}$), 1.92 (m, 1H; C**H**^{Val}), 1.84 (m, 4H; C**H**$_3$-Ac, C$_\beta$-**H**^{Met}), 1.74 (m, 1H; C$_\beta$-**H**^{Met}), 1.18 (d, $^3J_{H\text{-}H}$ = 7.1 Hz, 3H; C**H**$_3^{Ala}$), 0.76 (d, $^3J_{H\text{-}H}$ = 5.5 Hz, 3H; C**H**$_3^{Val}$), 0.74 (d, $^3J_{H\text{-}H}$ = 5.6 Hz, 3H; C**H**$_3^{Val}$).

^{13}C NMR (151 MHz, DMSO) δ [ppm] = 172.6 (**C**=O^{Ala}), 171.7 (**C**=O^{Trp}), 171.6 (**C**=O^{Met}), 171.3 (**C**=O^{Val}, **C**=O^{Gly}), 169.9 (CH$_3$**C**=O), 134.7 (**C**7a), 129.6 (**C**3a), 125.5 (**C**2), 123.9 (**C**6), 120.2 (**C**5), 118.6 (**C**4), 111.8 (**C**3), 104.5 (**C**7), 58.0 (**C**$_\alpha^{Val}$), 53.5 (**C**$_\alpha^{Trp}$), 52.4 (**C**$_\alpha^{Met}$), 49.0 (**C**$_\alpha^{Ala}$), 42.4 (**C**$_\alpha^{Gly}$), 32.0 (**C**$_\beta^{Met}$), 31.1 (**C**$_\beta^{Val}$), 30.1 (**C**$_\gamma^{Met}$), 27.9 (**C**$_\beta^{Trp}$), 22.9 (**C**H$_3$C=O), 19.6 (**C**H$_3^{Val}$), 18.4 (**C**H$_3^{Val}$), 18.2 (**C**H$_3^{Ala}$), 15.1 (**C**H$_3^{Met}$).

HRMS (ESI): *m/z* calcd: 682.2017 [M+H]$^+$, 704.1836 [M+Na]$^+$; found: 682.2017 [M+H]$^+$, 704.1838 [M+Na]$^+$.

LC-MS chromatogram at λ=220 nm

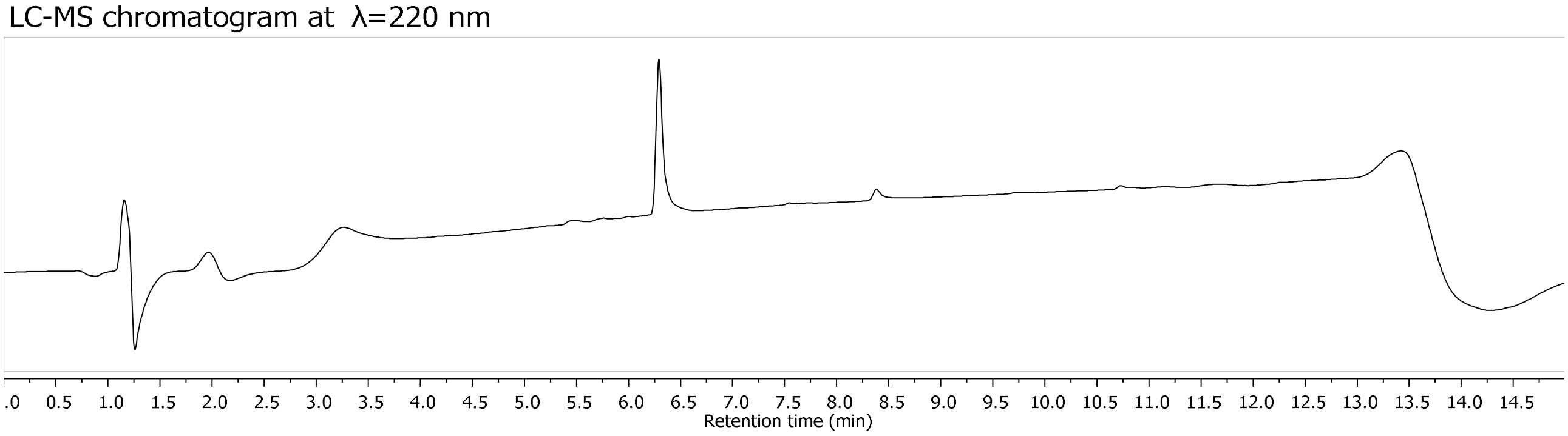

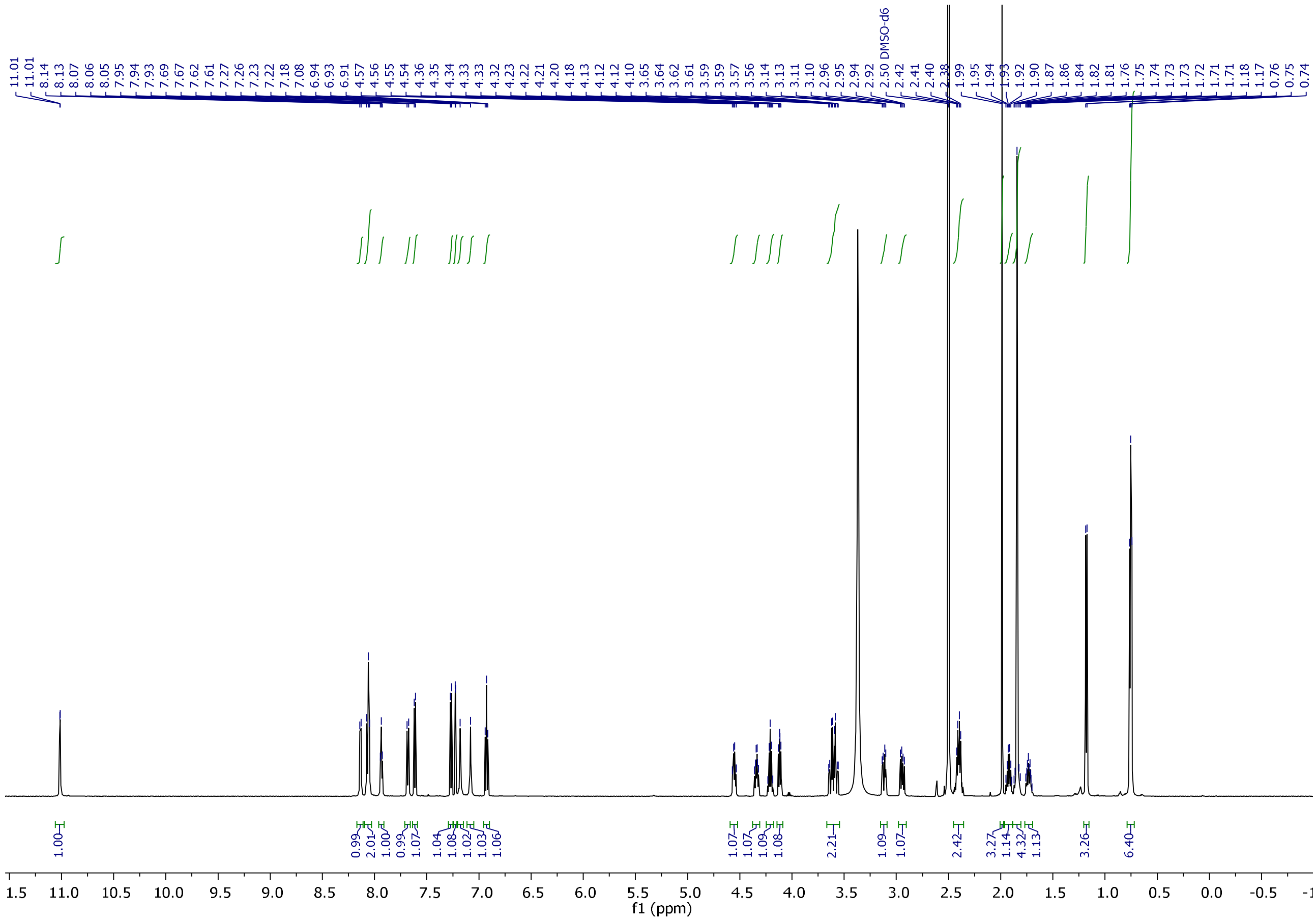

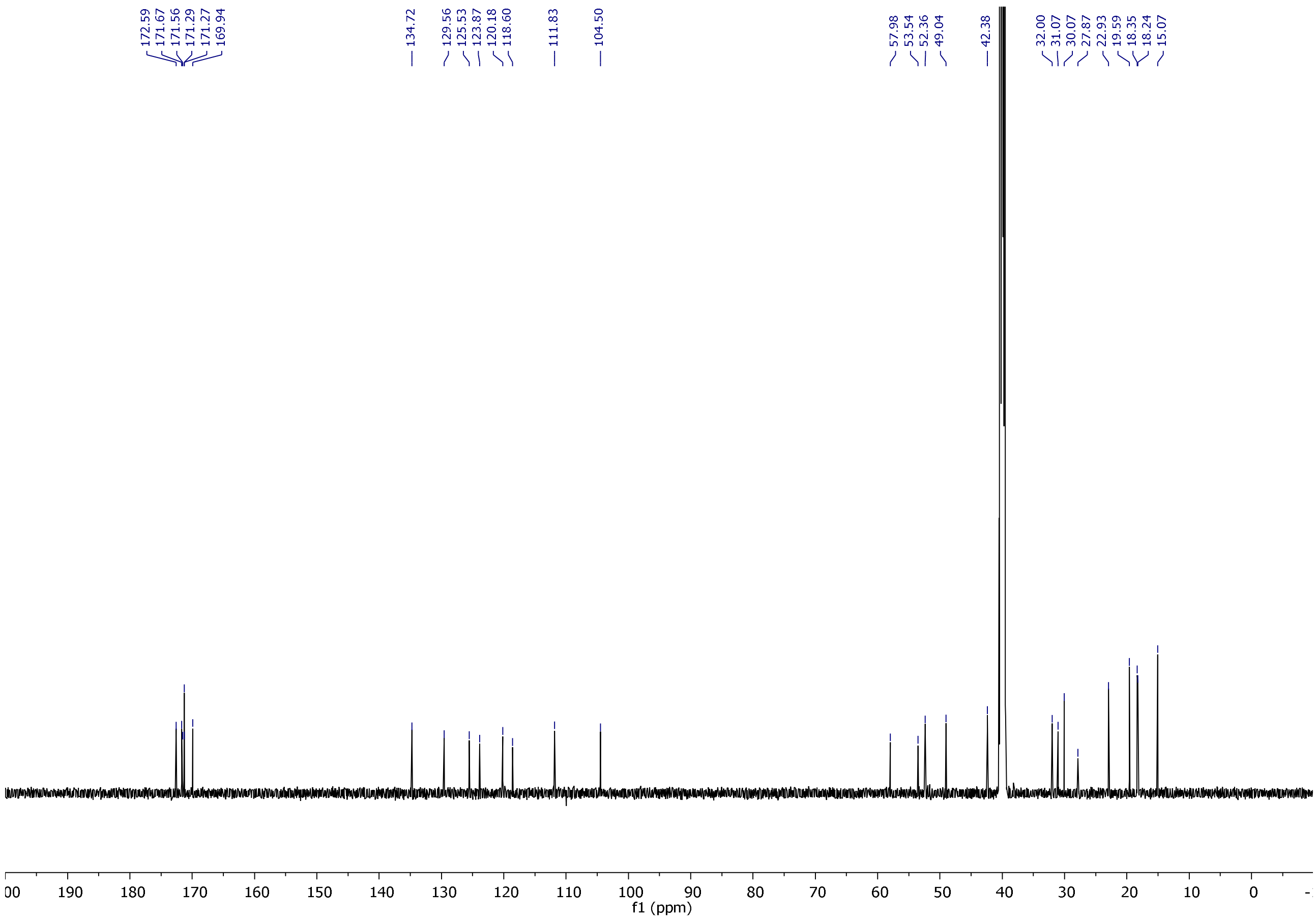

Ac-Leu-Val-Trp(6-Br)-Ala-Gly-NH2 (10)

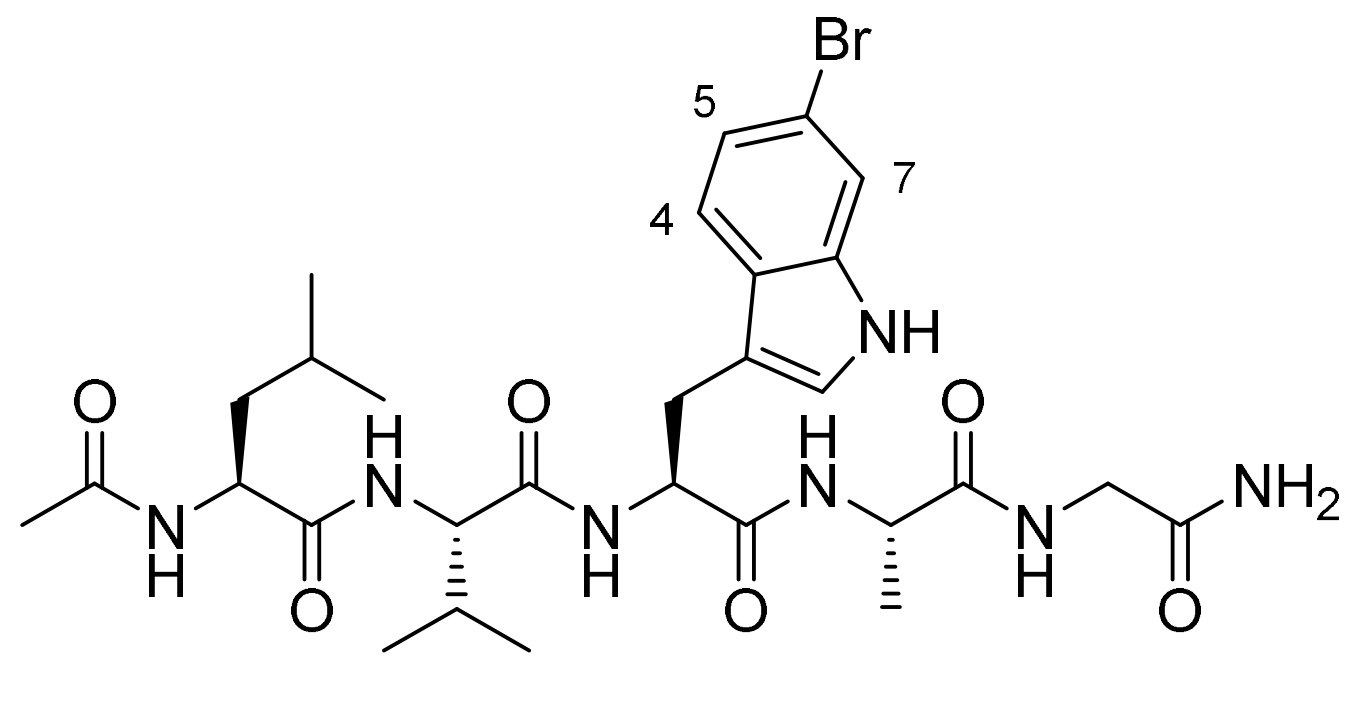

$C_{29}H_{42}BrN_7O_6$
MW = 664,60

Ac-Leu-Val-Trp(6-Br)-Ala-Gly-NH2 was synthesized *via* solid phase peptide synthesis (SPPS) according to GP2. The crude was purified via preparative RP-HPLC (preparative run protocol 2). Lyophilization provided the product as a colourless solid (79.1 mg, 0.12 mmol, 53%).

^{1}H NMR (600 MHz, [D$_6$]DMSO) δ [ppm] = 10.93 (s, 1H; N**H**-indole), 8.12 (d, $^3J_{H-H}$ = 6.6 Hz, 1H; N**H**^{Ala}), 8.01 (m, 2H; N**H**^{Trp}, N**H**^{Leu}), 7.93 (t, $^3J_{H-H}$ = 5.8 Hz, 1H; N**H**^{Gly}), 7.63 (d, $^3J_{H-H}$ = 8.7 Hz, 1H; N**H**^{Val}), 7.53 (d, $^3J_{H-H}$ = 8.4 Hz, 1H; C4-**H**^{Trp}), 7.48 (d, $^3J_{H-H}$ = 1.7 Hz, 1H; C7-**H**^{Trp}), 7.18 (s, 1H; N**H**$_2$), 7.16 (d, $^3J_{H-H}$ = 2.0 Hz, 1H; C2-**H**^{Trp}), 7.08 (dd, $^3J_{H-H}$ = 8.4 Hz, $^3J_{H-H}$ = 1.7 Hz, 1H; C5-**H**^{Trp}), 7.08 (s, 1H; N**H**$_2$), 4.53 (ddd, $^3J_{H-H}$ = 8.2 Hz, $^3J_{H-H}$ = 7.9 Hz, $^3J_{H-H}$ = 5.5 Hz, 1H; C$_\alpha$-**H**^{Trp}), 4.28 (m, 1H; C$_\alpha$-**H**^{Leu}), 4.20 (m, 1H; C$_\alpha$-**H**^{Ala}), 4.10 (dd, $^3J_{H-H}$ = 8.7 Hz, $^3J_{H-H}$ = 6.9 Hz, 1H; C$_\alpha$-**H**^{Val}), 3.63 (dd, $^2J_{H-H}$ = 16.8 Hz, $^3J_{H-H}$ = 5.8 Hz, 1H; C$_\alpha$-**H**^{Gly}), 3.57 (dd, $^2J_{H-H}$ = 16.8 Hz, $^3J_{H-H}$ = 5.8 Hz, 1H; C$_\alpha$-**H**^{Gly}), 3.09 (dd, $^2J_{H-H}$ = 14.8 Hz, $^3J_{H-H}$ = 5.5 Hz, 1H; C$_\beta$-**H**^{Trp}), 2.92 (dd, $^2J_{H-H}$ = 14.8 Hz, $^3J_{H-H}$ = 8.2 Hz, 1H; C$_\beta$-**H**^{Trp}), 1.91 (m, 1H; C**H**^{Val}), 1.83 (s, 3H; C**H**$_3$-Ac), 1.55 (m, 1H; C**H**^{Leu}), 1.39 (m, 2H; C-**H**$_2$^{Leu}), 1.17 (d, $^3J_{H-H}$ = 7.1 Hz, 3H; C**H**$_3$^{Ala}), 0.85 (d, $^3J_{H-H}$ = 6.6 Hz, 3H; C**H**$_3$^{Leu}), 0.81 (d, $^3J_{H-H}$ = 6.6 Hz, 3H; C**H**$_3$^{Leu}) 0.75 (d, $^3J_{H-H}$ = 6.7 Hz, 3H; C**H**$_3$^{Val}), 0.75 (d, $^3J_{H-H}$ = 6.7 Hz, 3H; C**H**$_3$^{Val}).

^{13}C NMR (151 MHz, DMSO): δ [ppm] = 172.6 (**C**=O^{Ala}), 172.5 (**C**=O^{Leu}), 171.5 (**C**=O^{Trp}), 171.3 (**C**=O^{Val}, **C**=O^{Gly}), 169.7 (CH$_3$**C**=O), 137.4 (**C**7a), 126.8 (**C**3a), 125.2 (**C**2), 121.5 (**C**5), 120.8 (**C**4), 114.2 (**C**6), 114.1 (**C**7), 110.7 (**C**3), 57.9 (**C**$_\alpha^{Val}$), 53.5 (**C**$_\alpha^{Trp}$), 51.6 (**C**$_\alpha^{Leu}$), 49.0 (**C**$_\alpha^{Ala}$), 42.4 (**C**$_\alpha^{Gly}$), 40.8 (**C**$_\beta^{Leu}$), 31.1 (**C**$_\beta^{Val}$), 27.7 (**C**$_\beta^{Trp}$), 24.7 (**C**$_\gamma^{Leu}$), 23.5 (**C**H$_3$^{Leu}), 22.9 (**C**H$_3$C=O), 22.0 (**C**H$_3$^{Leu}), 19.6 (**C**H$_3$^{Val}), 18.4 (**C**H$_3$^{Val}), 18.3 (**C**H$_3$^{Ala}).

HRMS (ESI): *m/z* calcd: 664.2453 [M+H]$^+$, 686.2272 [M+Na]$^+$;found: 664.2454 [M+H]$^+$, 686.2278 [M+Na]$^+$.

UHPLC-MS chromatogram at λ = 220 nm

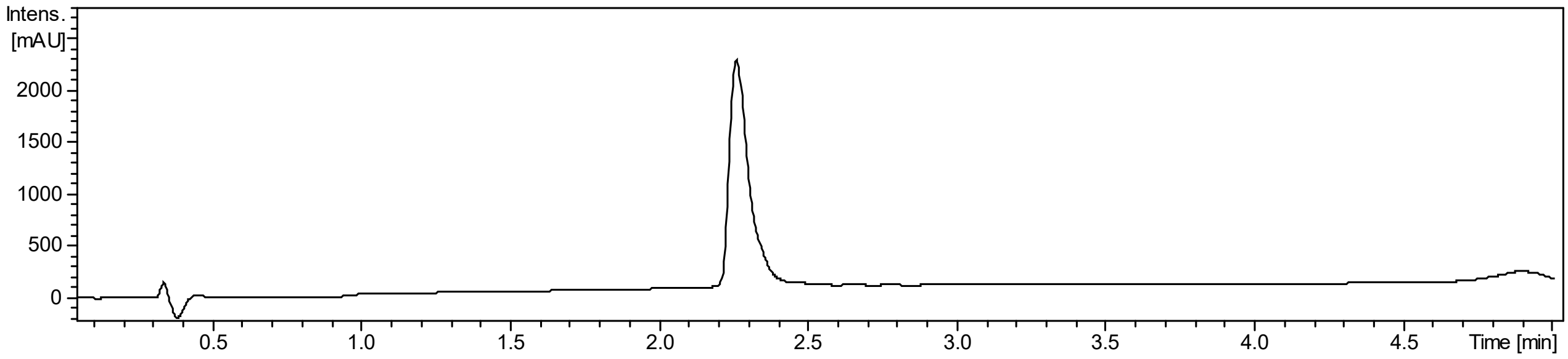

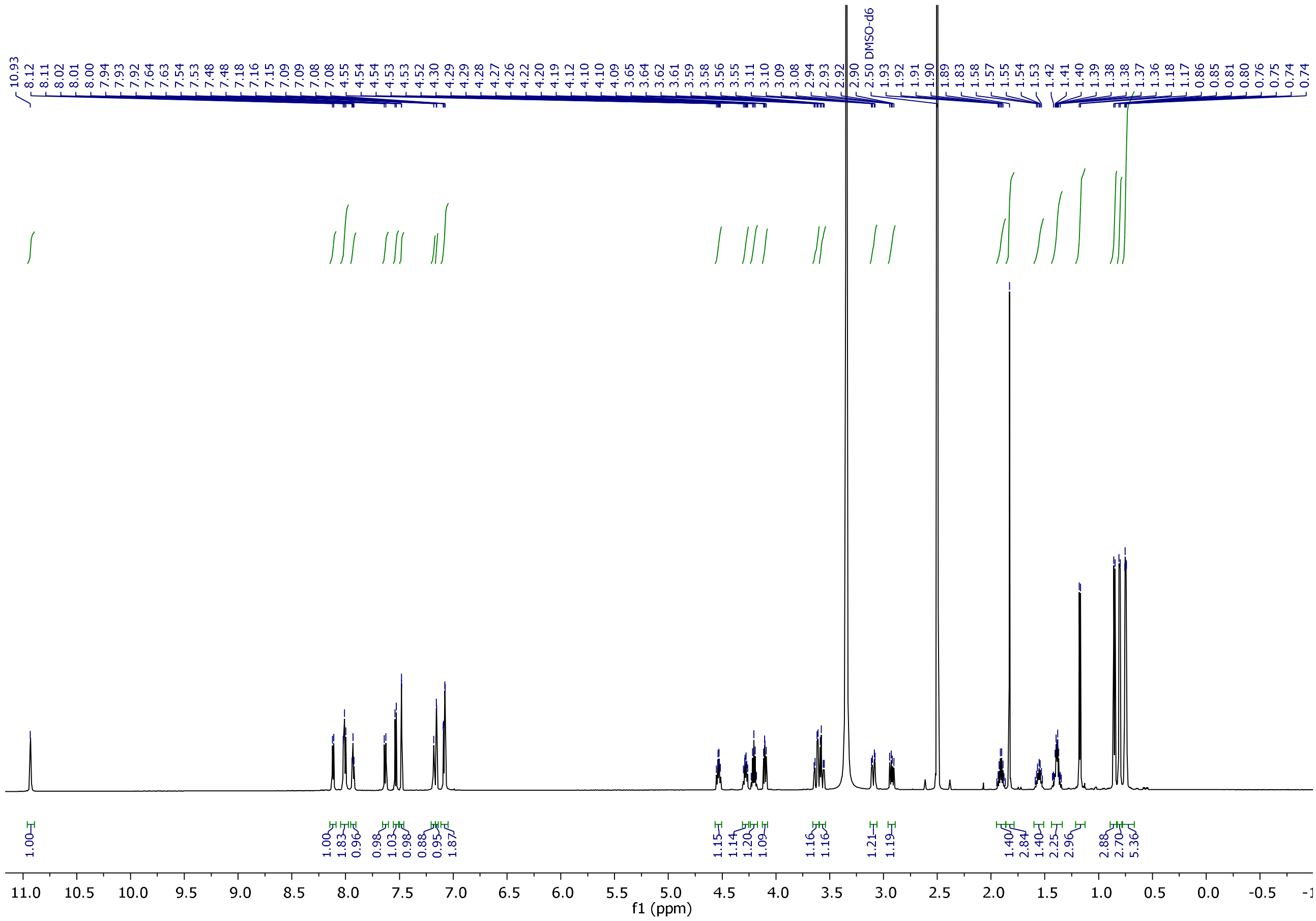

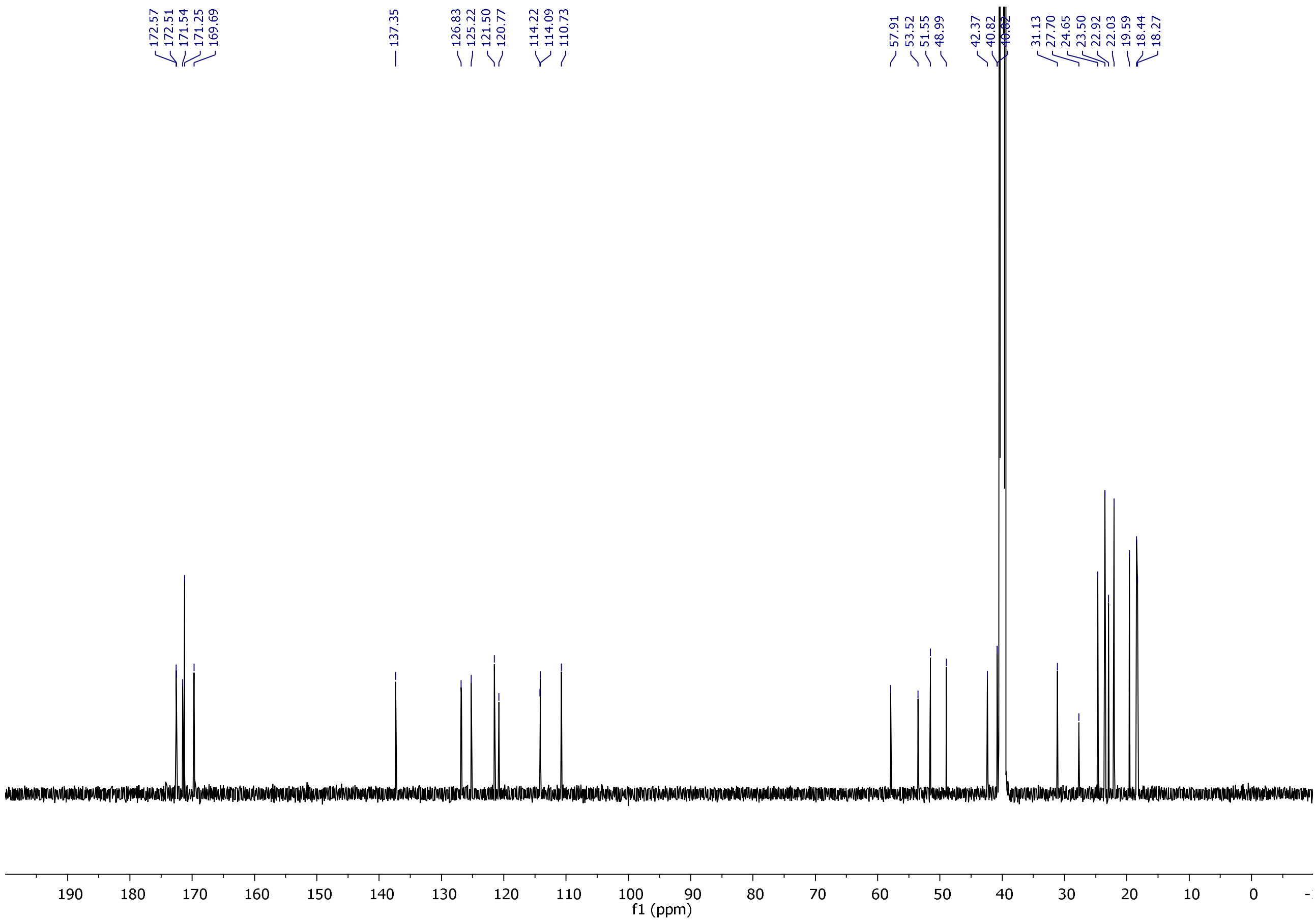

Ac-Ala-Trp(6-butyl)-Gly-NH2 (1a)

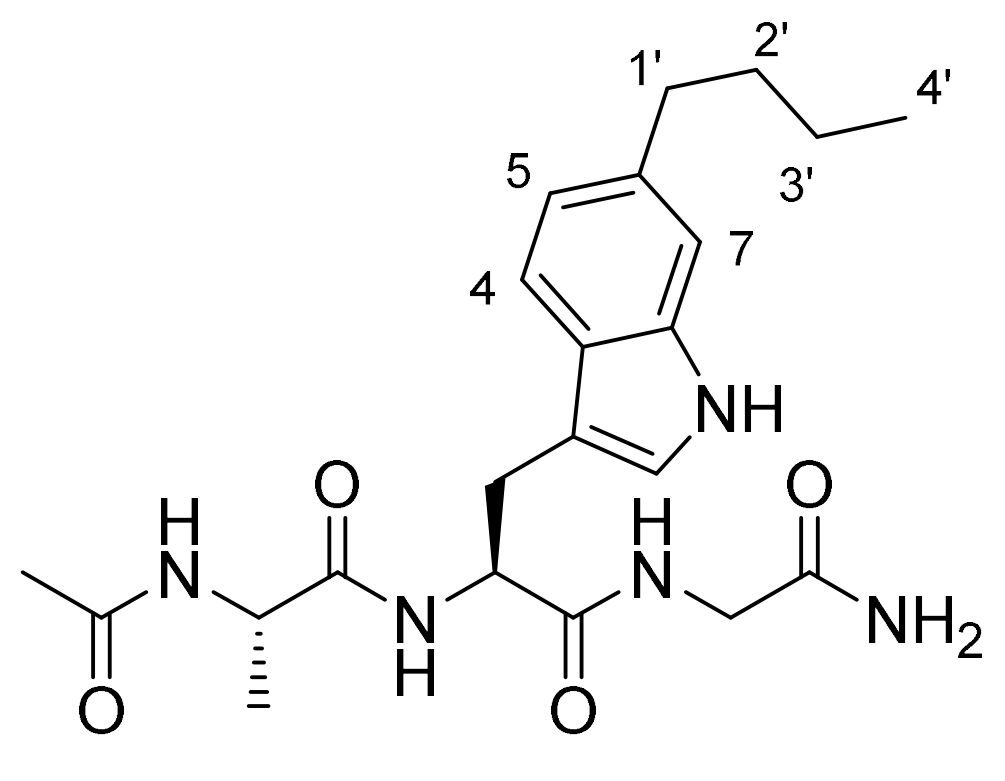

Ac-Ala-Trp(6-butyl)-Gly-NH_2 was synthesized according to GP1. Therefore, a stock solution of Ac-Ala-Trp(6-Br)-Gly-NH_2 (0.167 M) was prepared in anhydrous DMF and degassed under argon atmosphere for 30 min. Separately, activated zinc dust (14.18 mg, 217 µmol, 4.3 eq.) and Pd(amphos)$_2$Cl$_2$ (3.86 mg, 5.45 µmol, 11 mol%) were charged into a glass vial, which was subsequently placed under argon atmosphere. After 10 min anhydrous DMF (200 µL), 1-iodobutane (22.6 µL, 199 µmol, 4.0 eq.), and the peptide stock solution (0.167 M, 300 µL, 1.0 eq.) were added sequentially to the vial. After every addition the reaction mixture purged with argon for 5 min. The vial was sealed and the suspension was vigorously stirred at 40 °C for 24 h. When the reaction time elapsed, the mixture was allowed to cool to room temperature, and centrifuged. The crude product was purified by preparative RP-HPLC (preparative run protocol 3).

Lyophilization provided the product as a colourless solid (12.8 mg, 29.8 µmol, 59%).

^{1}H NMR (600 MHz, [D$_6$]DMSO): δ [ppm] = 10.65 (d, $^3J_{H\text{-}H}$ = 1.8 Hz 1H; N**H**-indole), 8.09 (t, $^3J_{H\text{-}H}$ = 5.8 Hz, 1H; N**H**^{Gly}), 8.05 (d, $^3J_{H\text{-}H}$ = 7.0 Hz, 1H; N**H**^{Ala}), 7.97 (d, $^3J_{H\text{-}H}$ = 7.5 Hz, 1H; N**H**^{Trp}), 7.42 (d, $^3J_{H\text{-}H}$ = 8.1 Hz, 1H; C4-**H**^{Trp}), 7.11 (s, 1H; N**H**$_2$), , 7.09 (s, 2H, N**H**$_2$; C7-**H**^{Trp}), 7.05 (d, $^3J_{H\text{-}H}$ = 1.8 Hz, 1H; C2-**H**^{Trp}), 6.81 (dd, $^3J_{H\text{-}H}$ = 8.1 Hz, $^3J_{H\text{-}H}$ = 1.2 Hz, 1H; C5-**H**^{Trp}), 4.41 (ddd, $^3J_{H\text{-}H}$ = 8.5 Hz, $^3J_{H\text{-}H}$ = 7.5 Hz, $^3J_{H\text{-}H}$ = 4.9 Hz, 1H; C$_\alpha$-**H**^{Trp}), 4.21 (m, 1H; C$_\alpha$-**H**^{Ala}), 3.67 (dd, $^2J_{H\text{-}H}$ = 16.8 Hz, $^3J_{H\text{-}H}$ = 5.8 Hz, 1H; C$_\alpha$-**H**^{Gly}), 3.52 (dd, $^2J_{H\text{-}H}$ = 16.8 Hz, $^3J_{H\text{-}H}$ = 5.8 Hz, 1H; C$_\alpha$-**H**^{Gly}), 3.14 (dd, $^2J_{H\text{-}H}$ = 14.7 Hz, $^3J_{H\text{-}H}$ = 4.9 Hz, 1H; C$_\beta$-**H**^{Trp}), 2.96 (dd, $^2J_{H\text{-}H}$ = 14.7 Hz, $^3J_{H\text{-}H}$ = 8.5 Hz, 1H; C$_\beta$-**H**^{Trp}), 2.63 (t, $^3J_{H\text{-}H}$ = 7.6 Hz, 2H; C1'-**H**$_2$), 1.79 (s, 3H; C**H**$_3$-Ac), 1.57 (m, 2H; C2'-**H**$_2$), 1.31 (m, 2H; C3'-**H**$_2$), 1.12 (d, $^3J_{H\text{-}H}$ = 7.2 Hz, 3H; C**H**$_3$^{Ala}), 0.90 (t, $^3J_{H\text{-}H}$ = 7.4 Hz, 3H; C4'-**H**$_3$).

^{13}C NMR (151 MHz, DMSO-d_6): δ [ppm] = 172.6 (**C**=O^{Ala}), 171.4 (**C**=O^{Trp}), 170.9 (**C**=O^{Gly}), 169.4 (CH$_3$**C**=O), 136.4 (**C**7a), 135.0 (**C**6), 125.5 (**C**3a), 122.9 (**C**2), 119.4 (**C**5), 118.0 (**C**4), 110.5 (**C**7), 109.7 (**C**3), 53.7 (**C**$_\alpha$^{Trp}), 48.3 (**C**$_\alpha$^{Ala}), 42.0 (**C**$_\alpha$^{Gly}), 35.2 (**C**1'), 33.9 (**C**2'), 26.7 (**C**$_\beta$^{Trp}), 22.4 (**C**H$_3$C=O), 21.8 (**C**3'), 17.6 (**C**H$_3$^{Ala}), 13.9 (**C**4').

HRMS (ESI): *m/z* calcd: 430.2448 [M+H]$^+$, 452.2268 [M+Na]$^+$; found: 430.2437 [M+H]$^+$, 452.2264 [M+Na]$^+$.

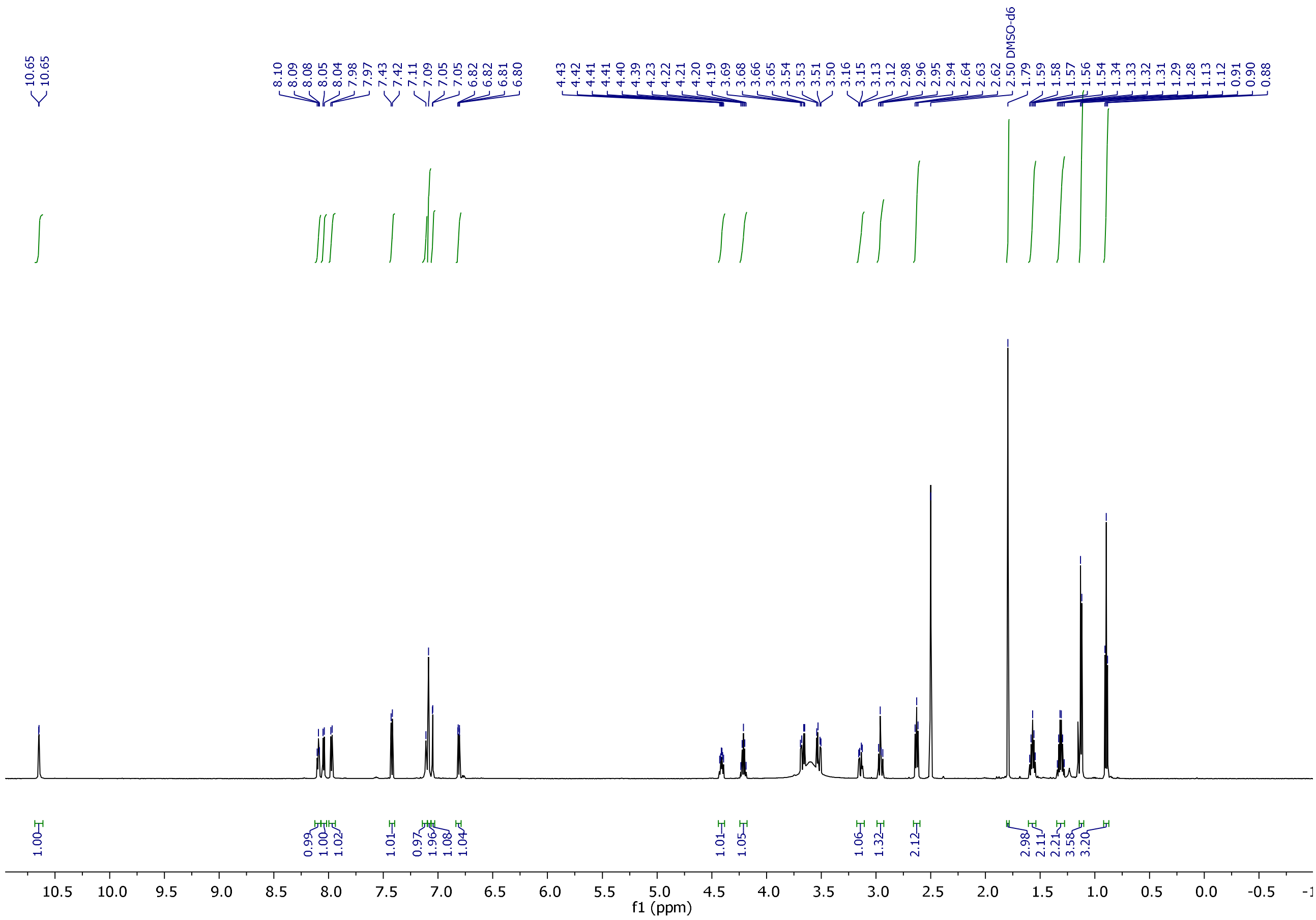

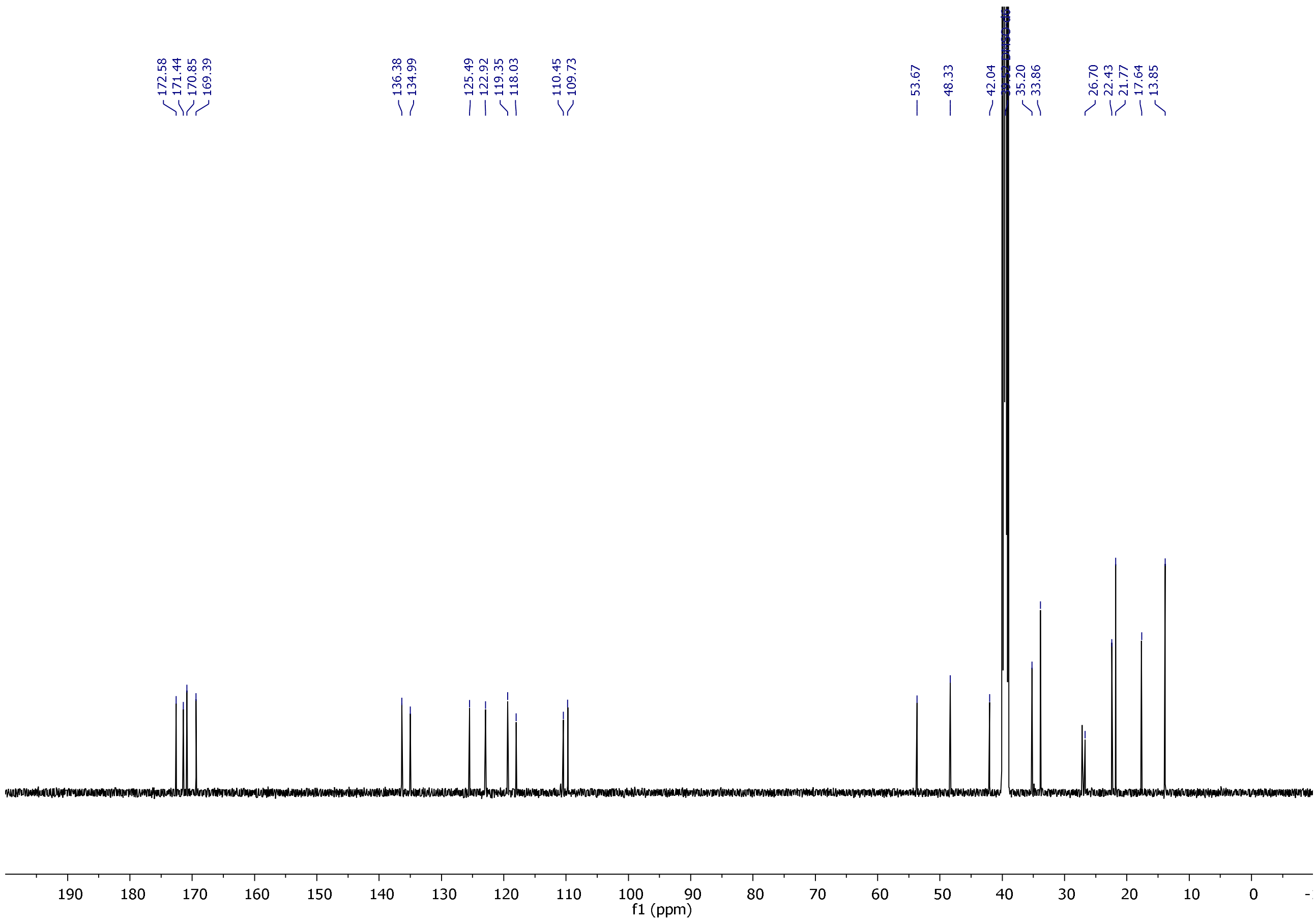

UHPLC-MS chromatogram at λ = 220 nm

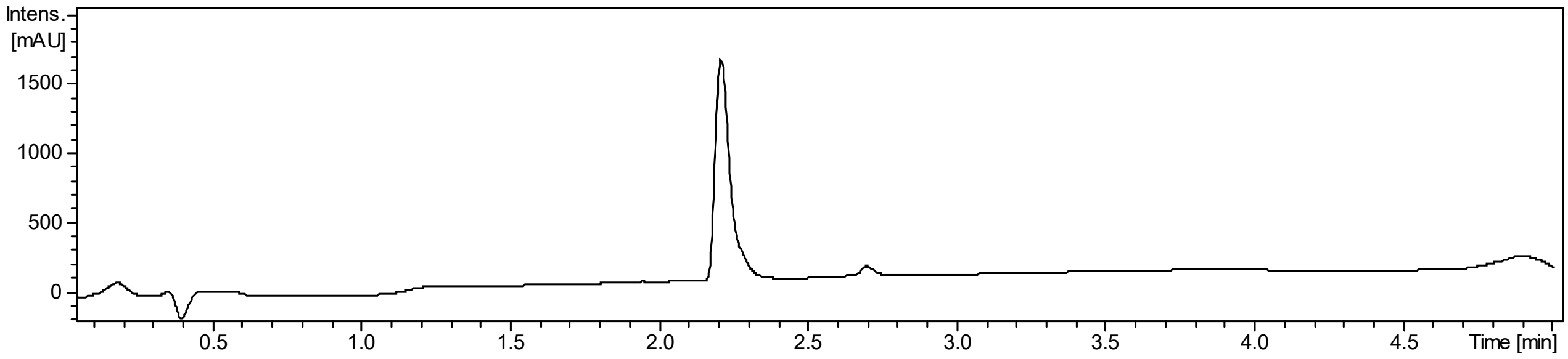

Ac-Ala-Trp(6-cyclohexyl)-Gly-NH2 (1b)

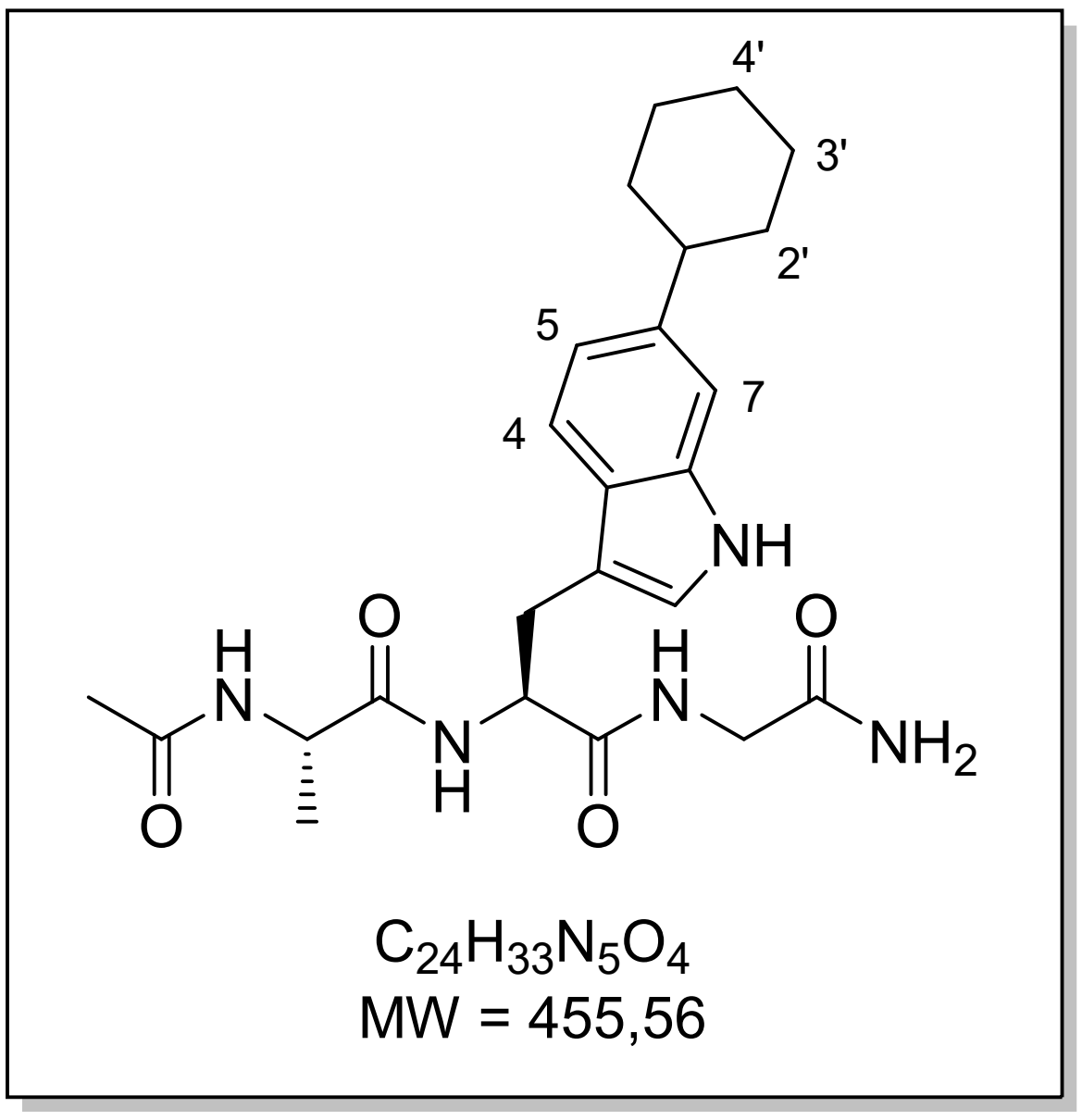

Ac-Ala-Trp(6-cyclohexyl)-Gly-NH_2 was synthesized according to GP1. Therefore, a stock solution of Ac-Ala-Trp(6-Br)-Gly-NH_2 (0.167 M) was prepared in anhydrous DMF and degassed under argon atmosphere for 30 min. Separately, activated zinc dust (14.06 mg, 215 µmol, 4.3 eq.) and Pd(amphos)$_2$Cl$_2$ (3.80 mg, 5.37 µmol, 11 mol%) were charged into a glass vial, which was subsequently placed under argon atmosphere. After 10 min anhydrous DMF (200 µL), iodocyclohexane (26.0 µL, 201 µmol, 4.0 eq.), and the peptide stock solution (0.167 M, 300 µL, 1.0 eq.) were added sequentially to the vial. After every addition the reaction mixture purged with argon for 5 min. The vial was sealed and the suspension was vigorously stirred at 40 °C for 48 h. When the reaction time elapsed, the mixture was allowed to cool to room temperature, and centrifuged. The crude product was purified by preparative RP-HPLC. (preparative run protocol 3).
Lyophilization provided the product as a colourless solid (12.5 mg, 27.4 µmol, 55%).

^{1}H NMR (600 MHz, [D$_6$]DMSO): δ [ppm] = 10.65 (s, 1H; N**H**-indole), 8.10 (t, $^3J_{H\text{-}H}$ = 5.8 Hz, 1H; N**H**^{Gly}), 8.04 (d, $^3J_{H\text{-}H}$ = 7.1 Hz, 1H; N**H**^{Ala}), 7.96 (d, $^3J_{H\text{-}H}$ = 7.6 Hz, 1H; N**H**^{Trp}), 7.43 (d, $^3J_{H\text{-}H}$ = 8.3 Hz, 1H; C4-**H**^{Trp}), 7.11 (s, 2H, N**H**$_2$; C7-**H**^{Trp}), 7.09 (s, 1H; N**H**$_2$), 7.05 (d, $^3J_{H\text{-}H}$ = 2.0 Hz, 1H; C2-**H**^{Trp}), 6.85 (dd; $^3J_{H\text{-}H}$ = 8.3 Hz, $^3J_{H\text{-}H}$ = 1.1 Hz, 1H; C5-**H**^{Trp}), 4.40 (ddd, $^3J_{H\text{-}H}$ = 8.4 Hz, $^3J_{H\text{-}H}$ = 7.6 Hz, $^3J_{H\text{-}H}$ = 5.0 Hz, 1H; C$_\alpha$-**H**^{Trp}), 4.21 (m, 1H; C$_\alpha$-**H**^{Ala}), 3.67 (dd, $^2J_{H\text{-}H}$ = 16.8 Hz, $^3J_{H\text{-}H}$ = 5.8 Hz, 1H; C$_\alpha$-**H**^{Gly}), 3.52 (dd, $^2J_{H\text{-}H}$ = 16.8 Hz, $^3J_{H\text{-}H}$ = 5.8 Hz, 1H; C$_\alpha$-**H**^{Gly}), 3.14 (dd, $^2J_{H\text{-}H}$ = 14.7 Hz, $^3J_{H\text{-}H}$ = 5.0 Hz, 1H; C$_\beta$-**H**^{Trp}), 2.95 (dd, $^2J_{H\text{-}H}$ = 14.7 Hz, $^3J_{H\text{-}H}$ = 8.4 Hz, 1H; C$_\beta$-**H**^{Trp}), 2.53 (m, 1H; C1'-**H**), 1.81 (m, 4H; 2C**H**$_2$-cyclohexy), 1.79 (s, 3H; C**H**$_3$-Ac), 1.71 (m, 1H; C4'-**H**), 1.41 (m, 4H; 2C**H**$_2$-cyclohexy), 1.26 (m, 1H; C4'-**H**), 1.13 (d, $^3J_{H\text{-}H}$ = 7.1 Hz, 3H; C**H**$_3$^{Ala}).

^{13}C NMR (151 MHz, DMSO): δ [ppm] = 172.6 (**C**=O^{Ala}), 171.4 (**C**=O^{Trp}), 170.9 (**C**=O^{Gly}), 169.4 (CH$_3$**C**=O), 140.6 (**C**6), 136.4 (**C**7a), 125.6 (**C**3a), 123.1 (**C**2), 118.1 (**C**4), 118.0 (**C**5), 109.6 (**C**3), 108.6 (**C**7), 53.7 (**C**$_\alpha$^{Trp}), 48.3 (**C**$_\alpha$^{Ala}), 44.1 (**C**1'), 42.0 (**C**$_\alpha$^{Gly}), 34.7 (2**C**H$_2$-cyclohexy), 27.2 (**C**$_\beta$^{Trp}), 26.4 (2**C**H$_2$-cyclohexy), 25.8 (**C**4'), 22.4 (**C**H$_3$C=O), 17.6 (**C**H$_3$^{Ala}).

HRMS (ESI): *m/z* calcd: 456.2605 [M+H]$^+$, 478.2425 [M+Na]$^+$, 494.2164 [M+K]$^+$; found: 456.2595 [M+H]$^+$, 478.2419 [M+Na]$^+$, 494.2156 [M+K]$^+$.

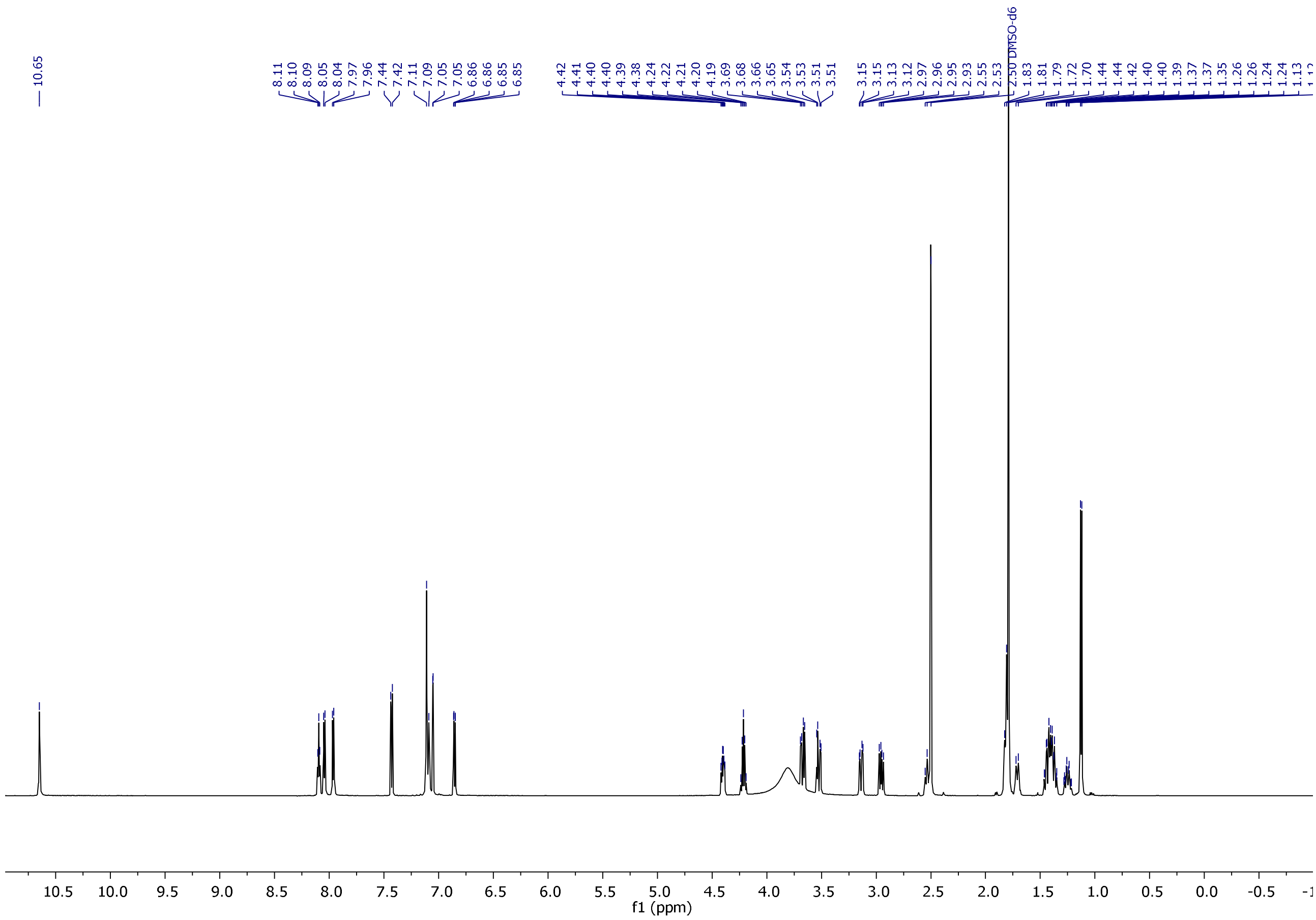
10.65
8.11
8.10
8.09
8.05
8.04
7.97
7.96
7.44
7.42
7.11
7.09
7.05
7.05
6.86
6.86
6.85
6.85
4.42
4.41
4.40
4.40
4.39
4.38
4.24
4.22
4.21
4.20
4.19
3.69
3.68
3.66
3.65
3.54
3.53
3.51
3.51
3.15
3.15
3.13
3.12
2.97
2.96
2.95
2.93
2.55
2.53
2.50 DMSO-d6
1.83
1.81
1.79
1.72
1.70
1.44
1.42
1.40
1.40
1.39
1.37
1.37
1.35
1.26
1.24
1.13
10.5
10.0
9.5
9.0
8.5
8.0
7.5
7.0
6.5
6.0
5.5
5.0
4.5
4.0
3.5
3.0
2.5
2.0
1.5
1.0
0.5
0.0
-0.5
f1 (ppm)

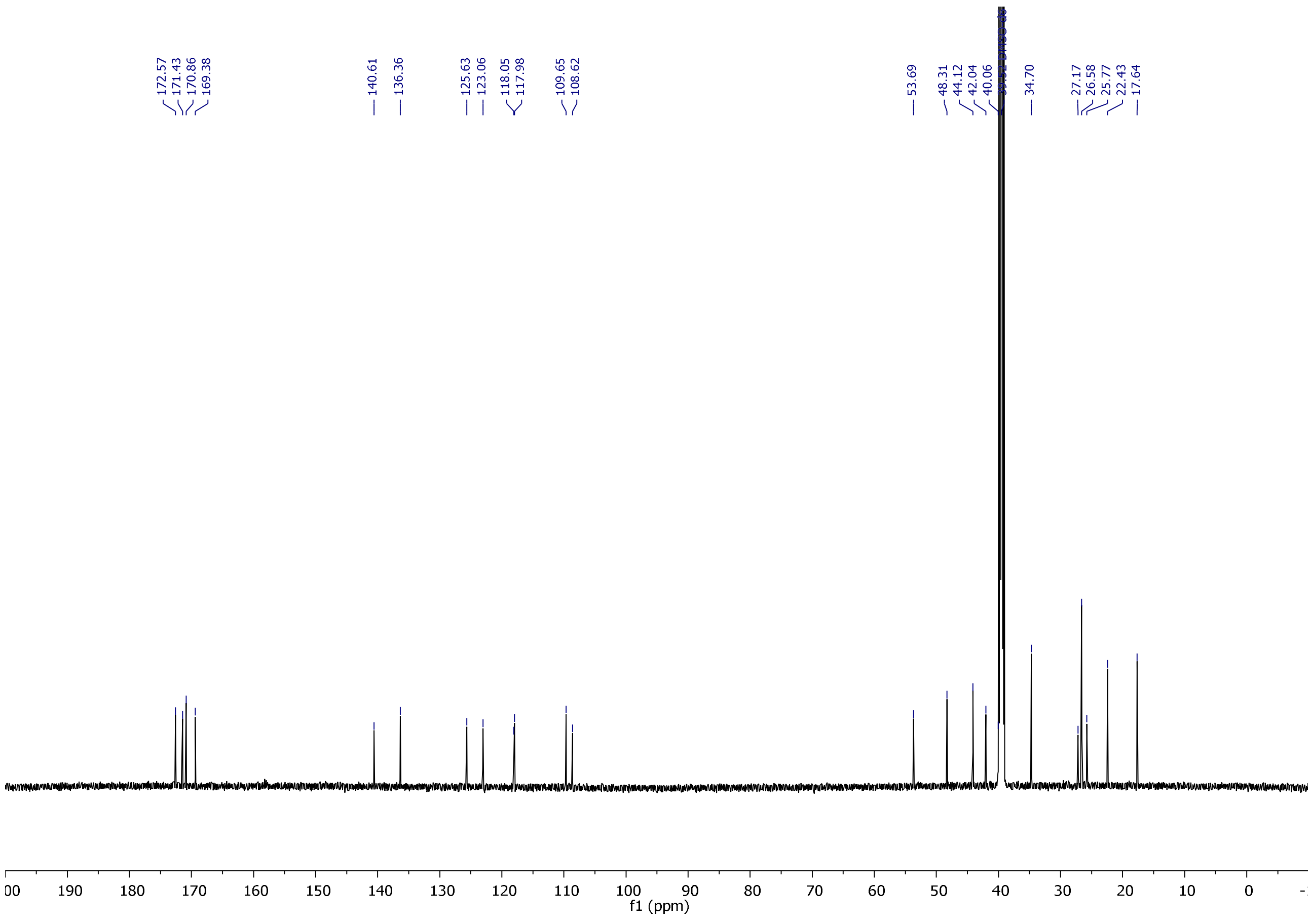
172.57
171.43
170.86
169.38
140.61
136.36
125.63
123.06
118.05
117.98
109.65
108.62
53.69
48.31
44.12
42.04
40.06
34.70
27.17
26.58
25.77
22.43
17.64
190
180
170
160
150
140
130
120
110
100
90
80
70
60
50
40
30
20
10
0
f1 (ppm)

UHPLC-MS chromatogram at λ = 220 nm

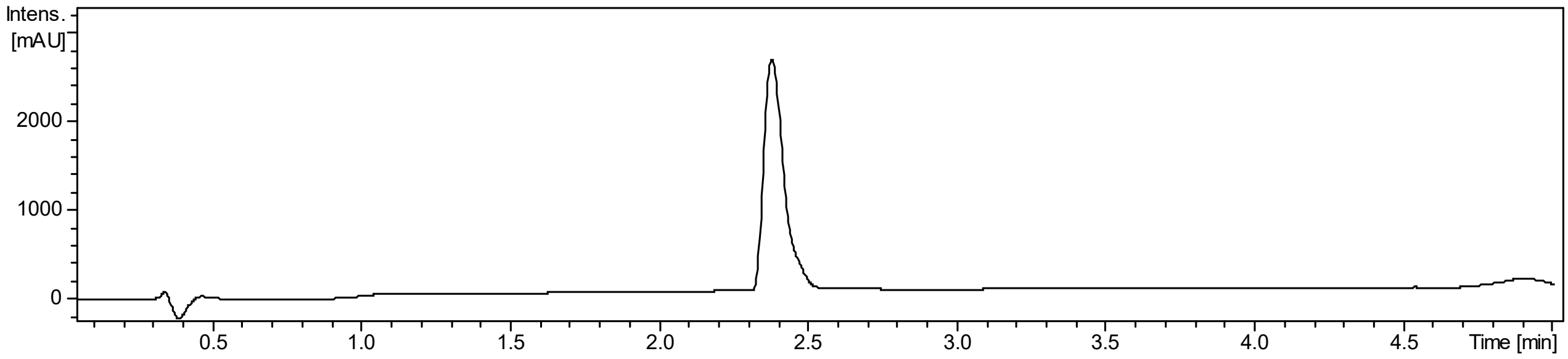

Ac-Ala-Trp(6-benzyl)-Gly-NH2 (1c)

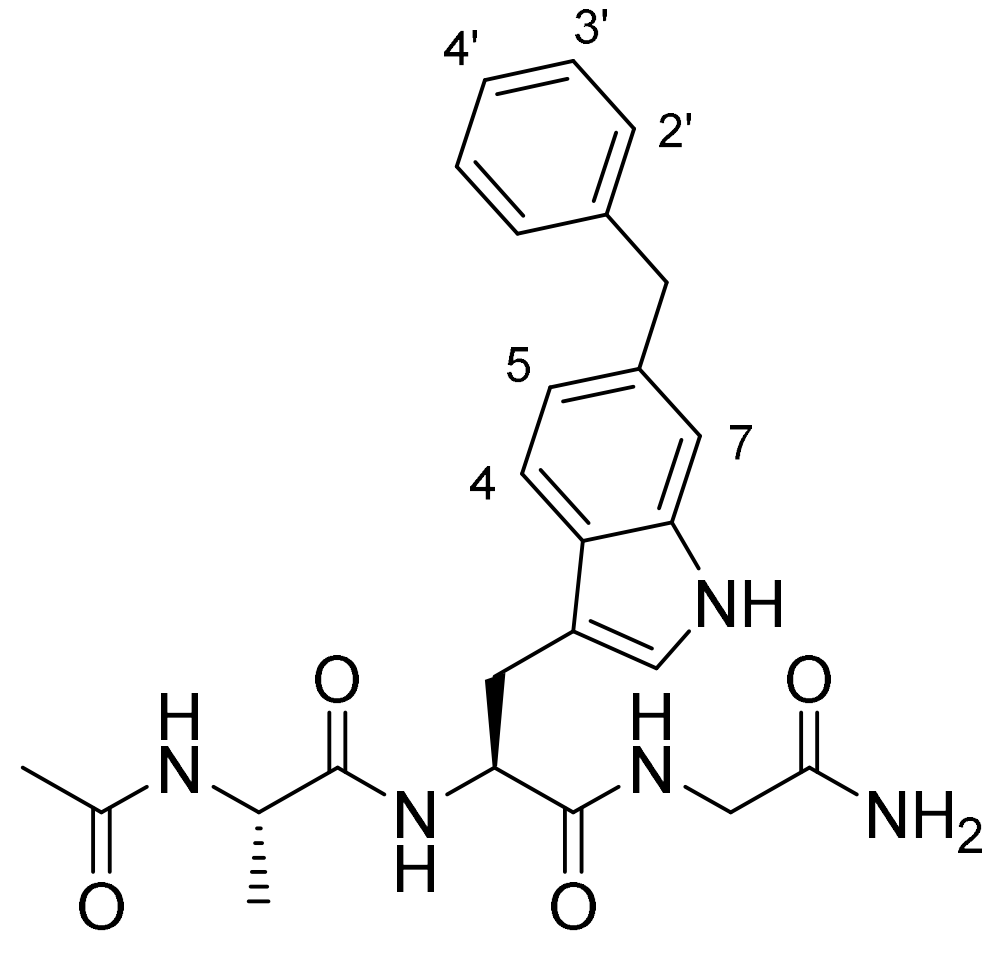

Ac-Ala-Trp(6-benzyl)-Gly-NH_2 was synthesized according to GP1. Therefore, a stock solution of Ac-Ala-Trp(6-Br)-Gly-NH_2 (0.167 M) was prepared in anhydrous DMF and degassed under argon atmosphere for 30 min. Separately, activated zinc dust (14.65 mg, 224 µmol, 4.5 eq.), Pd(amphos)$_2$$Cl_2$ (3.62 mg, 5.11 µmol, 10 mol%) and benzyl iodide (70.07 mg, 321 µmol, 6.4 eq.) were charged into a glass vial, which was subsequently placed under argon atmosphere. After 10 min anhydrous DMF (200 µL), and the peptide stock solution (0.167 M, 300 µL, 1.0 eq.) were added sequentially to the vial. After every addition the reaction mixture purged with argon for 5 min. The vial was sealed and the suspension was vigorously stirred at 40 °C for 48 h. When the reaction time elapsed, the mixture was allowed to cool to room temperature, and centrifuged. The crude product was purified by preparative RP-HPLC (preparative run protocol 4). Lyophilization provided the product as a colourless solid (22.6 mg, 48.8 µmol, 97%).

^{1}H NMR (600 MHz, [D$_6$]DMSO) δ [ppm] = 10.69 (s, 1H; N**H**-indole), 8.09 (t, $^3J_{H\text{-}H}$ = 5.8 Hz, 1H; N**H**^{Gly}), 8.04 (d, $^3J_{H\text{-}H}$ = 7.0 Hz, 1H; N**H**^{Ala}), 7.97 (d, $^3J_{H\text{-}H}$ = 7.6 Hz, 1H; N**H**^{Trp}), 7.44 (d, $^3J_{H\text{-}H}$ = 8.1 Hz, 1H; C4-**H**^{Trp}), 7.26 (m, 2H; C2'-**H**/C6'-**H**), 7.23 (m, 2H; C3'-**H**/C5'-**H**), 7.16 (m, 1H; C4'-**H**), 7.11 (s, 2H, N**H**$_2$; C7-**H**^{Trp}), 7.08 (s, 1H; N**H**$_2$), 7.06 (d, $^3J_{H\text{-}H}$ = 2.1 Hz, 1H; C2-**H**^{Trp}), 6.86 (dd, $^3J_{H\text{-}H}$ = 8.1 Hz, $^3J_{H\text{-}H}$ = 1.2 Hz, 1H; C5-**H**^{Trp}), 4.40 (ddd, $^3J_{H\text{-}H}$ = 8.4 Hz, $^3J_{H\text{-}H}$ = 7.6 Hz, $^3J_{H\text{-}H}$ = 4.9 Hz, 1H; C$_\alpha$-**H**^{Trp}), 4.21 (m, 1H; C$_\alpha$-**H**^{Ala}), 3.99 (s, 2H; C**H**$_2$), 3.67 (dd, $^2J_{H\text{-}H}$ = 16.8 Hz, $^3J_{H\text{-}H}$ = 5.8 Hz, 1H; C$_\alpha$-**H**^{Gly}), 3.51 (dd, $^2J_{H\text{-}H}$ = 16.8 Hz, $^3J_{H\text{-}H}$ = 5.8 Hz, 1H; C$_\alpha$-**H**^{Gly}), 3.13 (dd, $^2J_{H\text{-}H}$ = 14.8 Hz, $^3J_{H\text{-}H}$ = 4.9 Hz, 1H; C$_\beta$-**H**^{Trp}), 2.95 (dd, $^2J_{H\text{-}H}$ = 14.8 Hz, $^3J_{H\text{-}H}$ = 8.4 Hz, 1H; C$_\beta$-**H**^{Trp}), 1.78 (s, 3H; C**H**$_3$-Ac), 1.11 (d, $^3J_{H\text{-}H}$ = 7.1 Hz, 3H; C**H**$_3$^{Ala}).

^{13}C NMR (151 MHz, DMSO) δ [ppm] = 172.6 (**C**=O^{Ala}), 171.4 (**C**=O^{Trp}), 170.8 (**C**=O^{Gly}), 169.4 (CH$_3$**C**=O), 142.20 (**C**1'), 136.4 (**C**7a), 134.0 (**C**6), 128.7 (**C**2'/**C**6'), 128.3 (**C**3'/**C**5'), 125.7 (**C**3a), 125.6 (**C**4'), 123.3 (**C**2), 119.7 (**C**5), 118.2 (**C**4), 111.1 (**C**7), 109.8 (**C**3), 53.6 (**C**$_\alpha$^{Trp}), 48.3 (**C**$_\alpha$^{Ala}), 42.0 (**C**$_\alpha$^{Gly}), 41.5 (**C**H$_2$), 27.1 (**C**$_\beta$^{Trp}), 22.4 (**C**H$_3$C=O), 17.6 (**C**H$_3$^{Ala}).

HRMS (ESI): *m/z* calcd: 464.2292 [M+H]$^+$, 486.2112 [M+Na]$^+$, 502.1851 [M+K]$^+$;found 464.2283 [M+H]$^+$, 486.2106 [M+Na]$^+$, 502.1859 [M+K]$^+$.

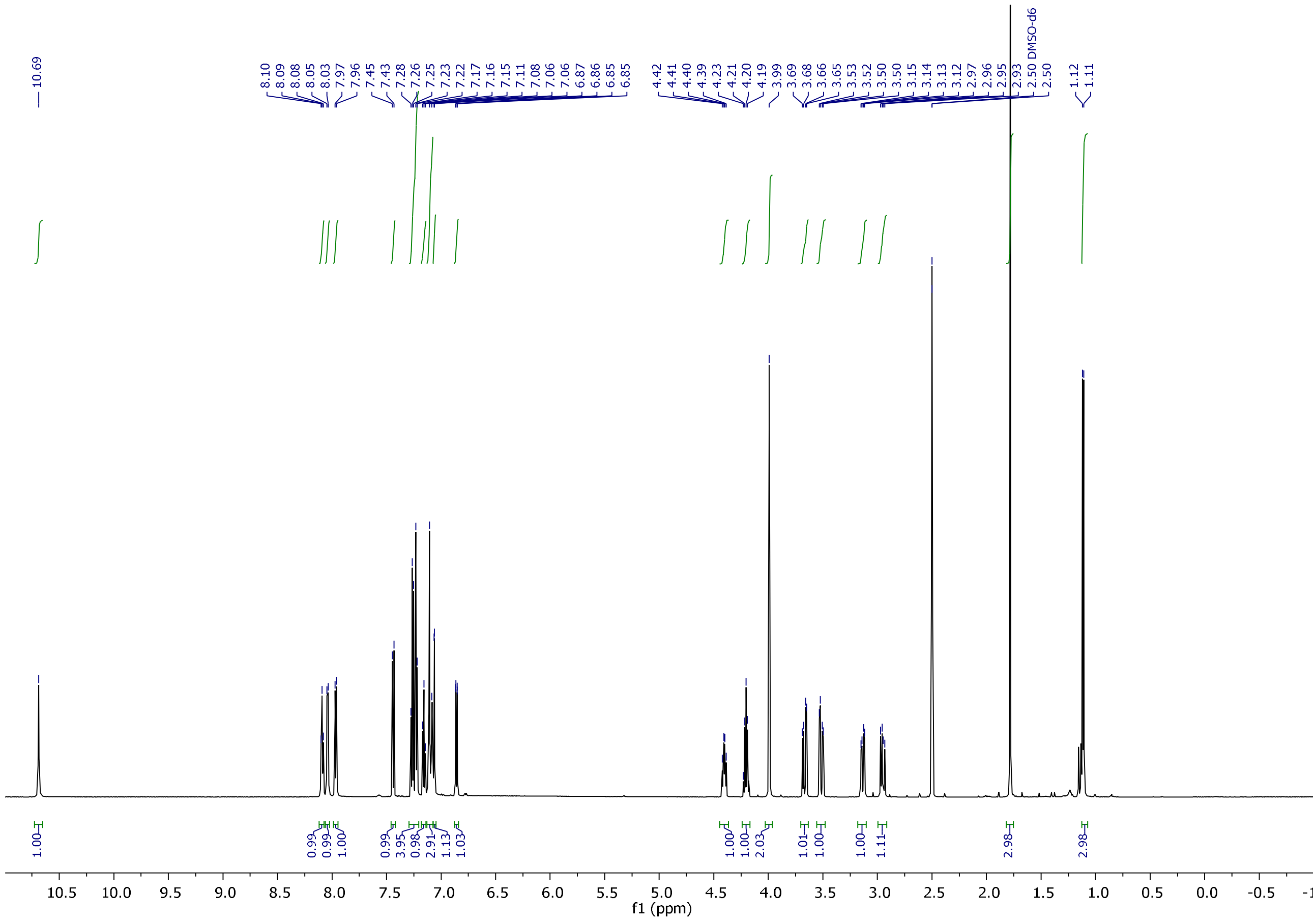
10.69
8.10 8.09 8.08 8.05 8.03 7.97 7.96 7.45 7.43 7.28 7.26 7.25 7.23 7.22 7.17 7.16 7.15 7.11 7.08 7.06 7.06 6.87 6.86 6.85 6.85
4.42 4.41 4.40 4.39 4.23 4.21 4.20 4.19 3.99 3.69 3.68 3.66 3.65 3.53 3.52 3.50 3.50 3.15 3.14 3.13 3.12 2.97 2.96 2.95 2.93 2.50 DMSO-d6 2.50
1.12 1.11
1.00 0.99 0.99 1.00 0.99 3.95 0.98 2.91 1.13 1.03 1.00 1.00 2.03 1.01 1.00 1.00 1.11 2.98 2.98
f1 (ppm)

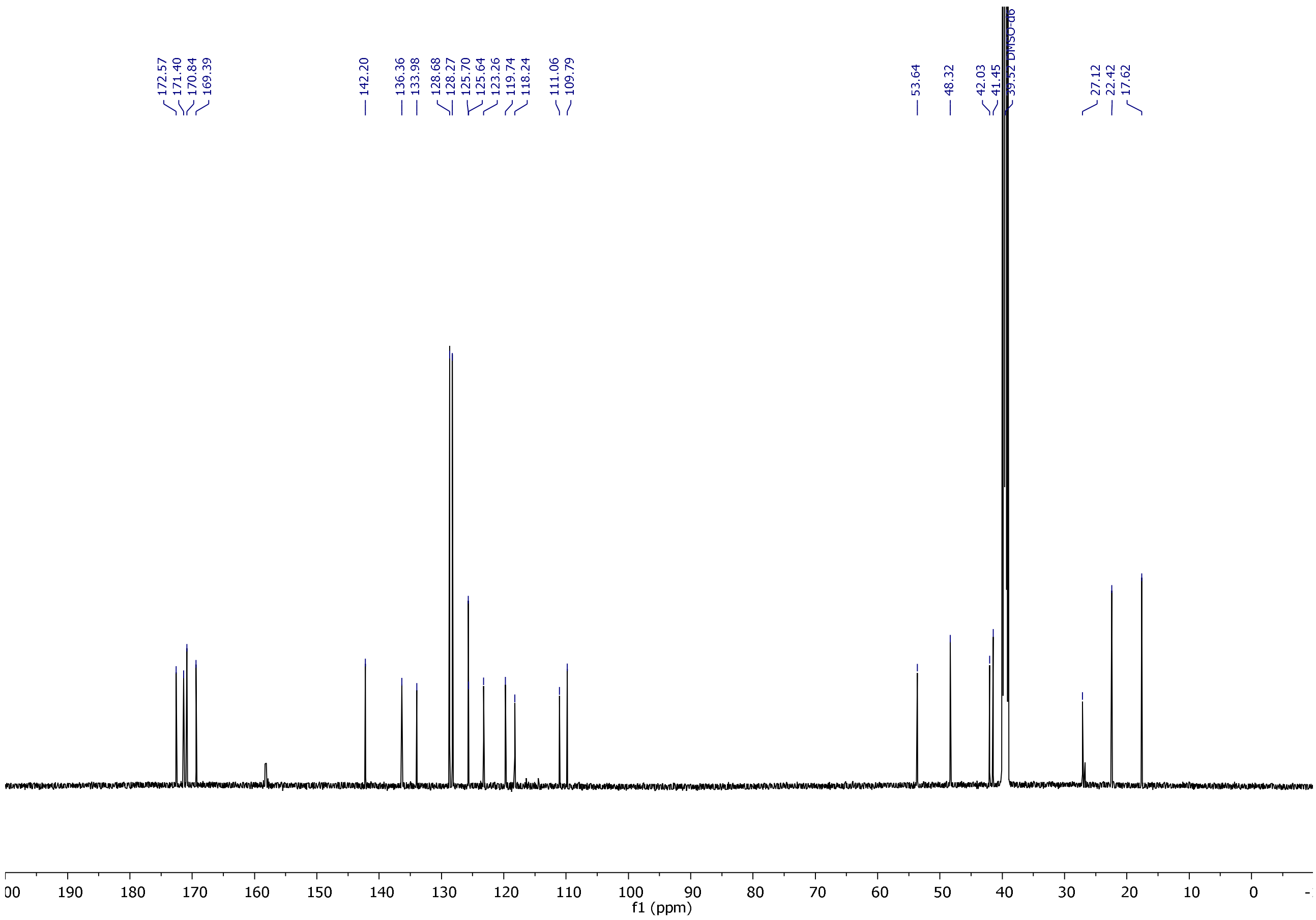
172.57 171.40 170.84 169.39
142.20
136.36 133.98 128.68 128.27 125.70 125.64 123.26 119.74 118.24 111.06 109.79
53.64 48.32 42.03 41.45 39.52 DMSO-d6
27.12 22.42 17.62
f1 (ppm)

UHPLC-MS chromatogram at λ = 220 nm

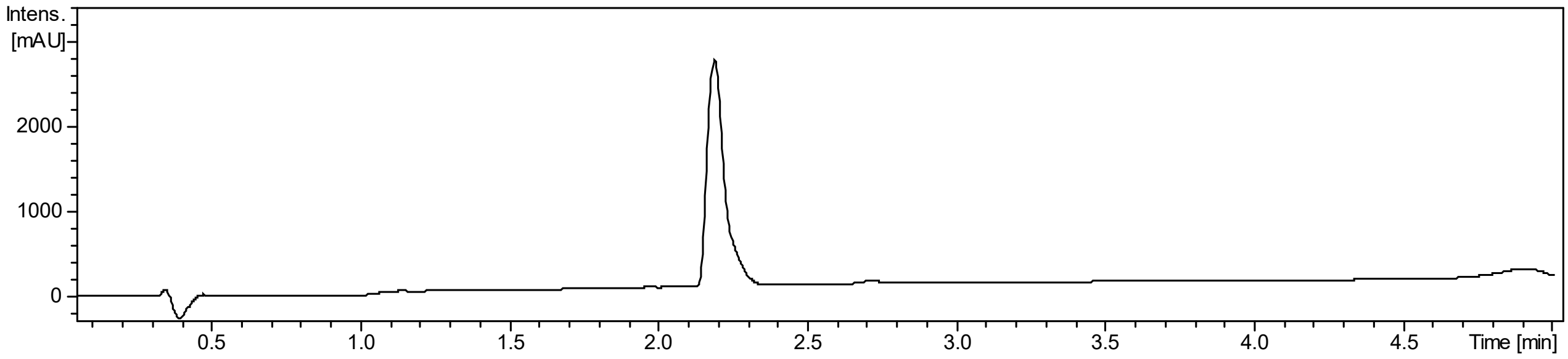

Peptide 1d

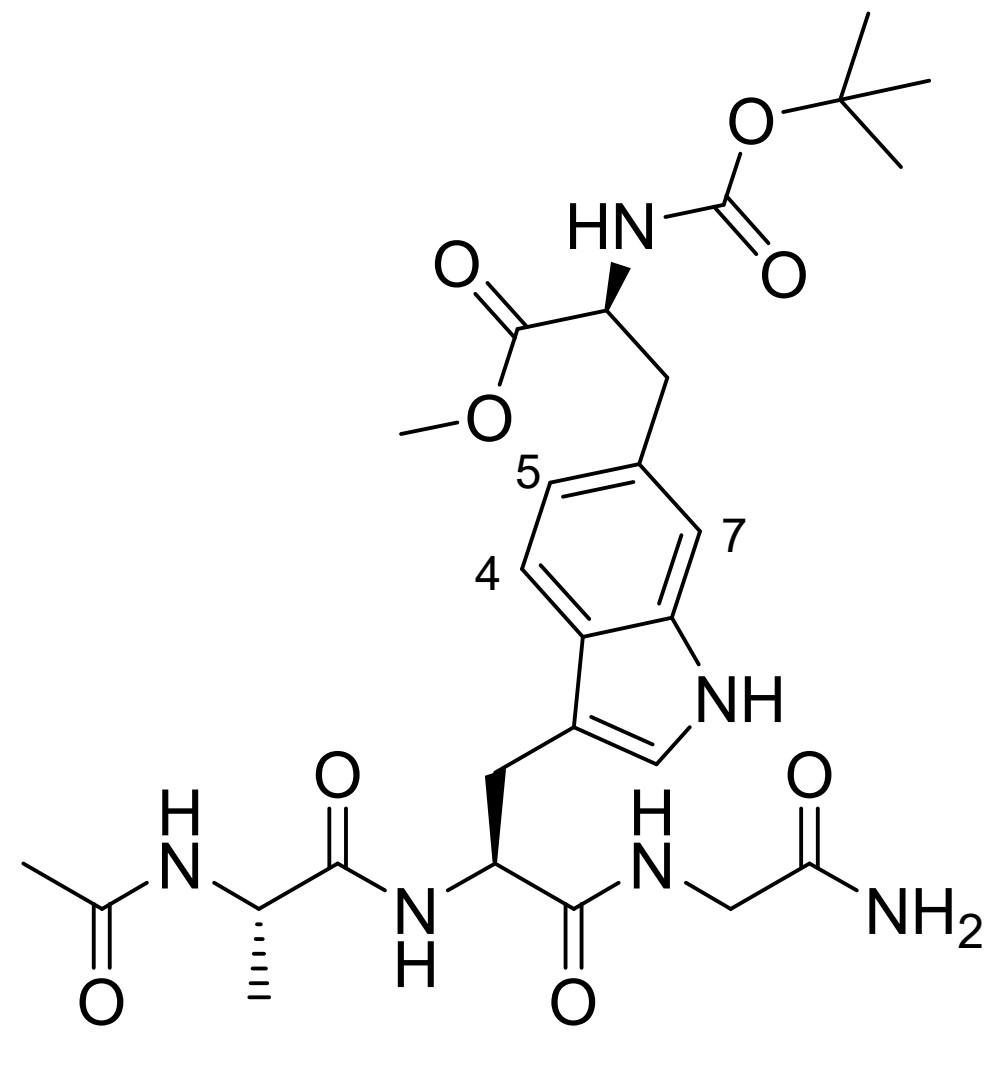

Peptide **1d** was synthesized according to GP1. Therefore, a stock solution of Ac-Ala-Trp(6-Br)-Gly-NH_2 (0.167 M) was prepared in anhydrous DMF and degassed under argon atmosphere for 30 min. Separately, activated zinc dust (14.24 mg, 218 µmol, 4.4 eq.), Pd(amphos)$_2$Cl$_2$ (3.68 mg, 5.20 µmol, 10 mol%) and N$^{\alpha}$-Boc-L-3-iodoalanine methyl ester (66.75 mg, 203 µmol, 4.0 eq.) were charged into a glass vial, which was subsequently placed under argon atmosphere. After 10 min anhydrous DMF (200 µL), and the peptide stock solution (0.167 M, 300 µL, 1.0 eq.) were added sequentially to the vial. The reaction mixture was purged with argon for 5 min. The vial was sealed and the suspension was vigorously stirred at 40 °C for 24 h. When the reaction time elapsed, the mixture was allowed to cool to room temperature and centrifuged. The crude product was purified by preparative RP-HPLC (preparative run protocol 4).

Lyophilization provided the product as a colourless solid (24.6 mg, 42.8 µmol, 86%).

^{1}H NMR (600 MHz, [D$_6$]DMSO) δ [ppm] = 10.76 (d, $^3J_{H\text{-}H}$ = 1.8 Hz, 1H; N**H**-indole), 8.09 (t, $^3J_{H\text{-}H}$ = 5.8 Hz, 1H; N**H**^{Gly}), 8.04 (d, $^3J_{H\text{-}H}$ = 7.0 Hz, 1H; N**H**^{Ala}), 7.98 (d, $^3J_{H\text{-}H}$ = 7.5 Hz, 1H; N**H**^{Trp}), 7.45 (d, $^3J_{H\text{-}H}$ = 8.1 Hz, 1H; C4-**H**^{Trp}), 7.23 (d, $^3J_{H\text{-}H}$ = 8.1 Hz, 1H; N**H**^{Ala2}), 7.17 (s, 1H; C7-**H**^{Trp}), 7.11 (s, 1H; N**H**$_2$), 7.08 (s, 2H, N**H**$_2$; C2-**H**^{Trp}), 6.84 (d, $^3J_{H\text{-}H}$ = 8.1 Hz, 1H; C5-**H**^{Trp}), 4.41 (ddd, $^3J_{H\text{-}H}$ = 8.0 Hz, $^3J_{H\text{-}H}$ = 7.5 Hz, $^3J_{H\text{-}H}$ = 4.9 Hz, 1H; C$_{\alpha}$-**H**^{Trp}), 4.21 (m, 1H; C$_{\alpha}$-**H**^{Ala}), 4.14 (ddd, $^3J_{H\text{-}H}$ = 8.7 Hz, $^3J_{H\text{-}H}$ = 8.1 Hz, $^3J_{H\text{-}H}$ = 5.2 Hz, 1H; C$_{\alpha}$-**H**^{Ala2}), 3.67 (dd, $^2J_{H\text{-}H}$ = 16.8 Hz, $^3J_{H\text{-}H}$ = 5.8 Hz, 1H; C$_{\alpha}$-**H**^{Gly}), 3.61 (s, 3H; C**H**$_3$-OMe), 3.52 (dd, $^2J_{H\text{-}H}$ = 16.8 Hz, $^3J_{H\text{-}H}$ = 5.8 Hz, 1H; C$_{\alpha}$-**H**^{Gly}), 3.14 (dd, $^2J_{H\text{-}H}$ = 14.8 Hz, $^3J_{H\text{-}H}$ = 4.9 Hz, 1H; C$_{\beta}$-**H**^{Trp}), 3.03 (dd, $^2J_{H\text{-}H}$ = 13.8 Hz, $^3J_{H\text{-}H}$ = 5.2 Hz, 1H; C$_{\beta}$-**H**^{Ala2}), 2.99-2.87 (m, 2H; C$_{\beta}$-**H**^{Trp}, C$_{\beta}$-**H**^{Ala2}), 1.79 (s, 3H; C**H**$_3$-Ac), 1.37-1.26 (m, 9H; 3C**H**$_3$-Boc), 1.12 (d, $^3J_{H\text{-}H}$ = 7.1 Hz, 3H; C**H**$_3$^{Ala}).

^{13}C NMR (151 MHz, DMSO): δ [ppm] = 172.9 (**C**=O^{Ala2}), 172.6 (**C**=O^{Ala}), 171.4 (**C**=O^{Trp}), 170.9 (**C**=O^{Gly}), 169.4 (CH$_3$**C**=O), 155.4 (NH**C**=O), 136.2 (**C**7a), 130.1 (**C**6), 126.1 (**C**3a), 123.4 (**C**2), 119.7 (**C**5), 118.1 (**C**4), 111.6 (**C**7), 109.8 (**C**3), 78.3 (**C**-Boc), 56.0 (**C**$_{\alpha}$^{Ala2}), 53.6 (**C**$_{\alpha}$^{Trp}), 51.7 (**C**H$_3$-OMe), 48.3 (**C**$_{\alpha}$^{Ala}), 42.0 (**C**$_{\alpha}$^{Gly}), 36.8 (**C**$_{\beta}$^{Ala2}), 28.1 (3**C**H$_3$-Boc), 27.1 (**C**$_{\beta}$^{Trp}), 22.4 (**C**H$_3$C=O), 17.6 (**C**H$_3$^{Ala}).

HRMS (ESI): *m/z* calcd: 597.2643 [M+Na]$^+$; found: 597.2648 [M+Na]$^+$.

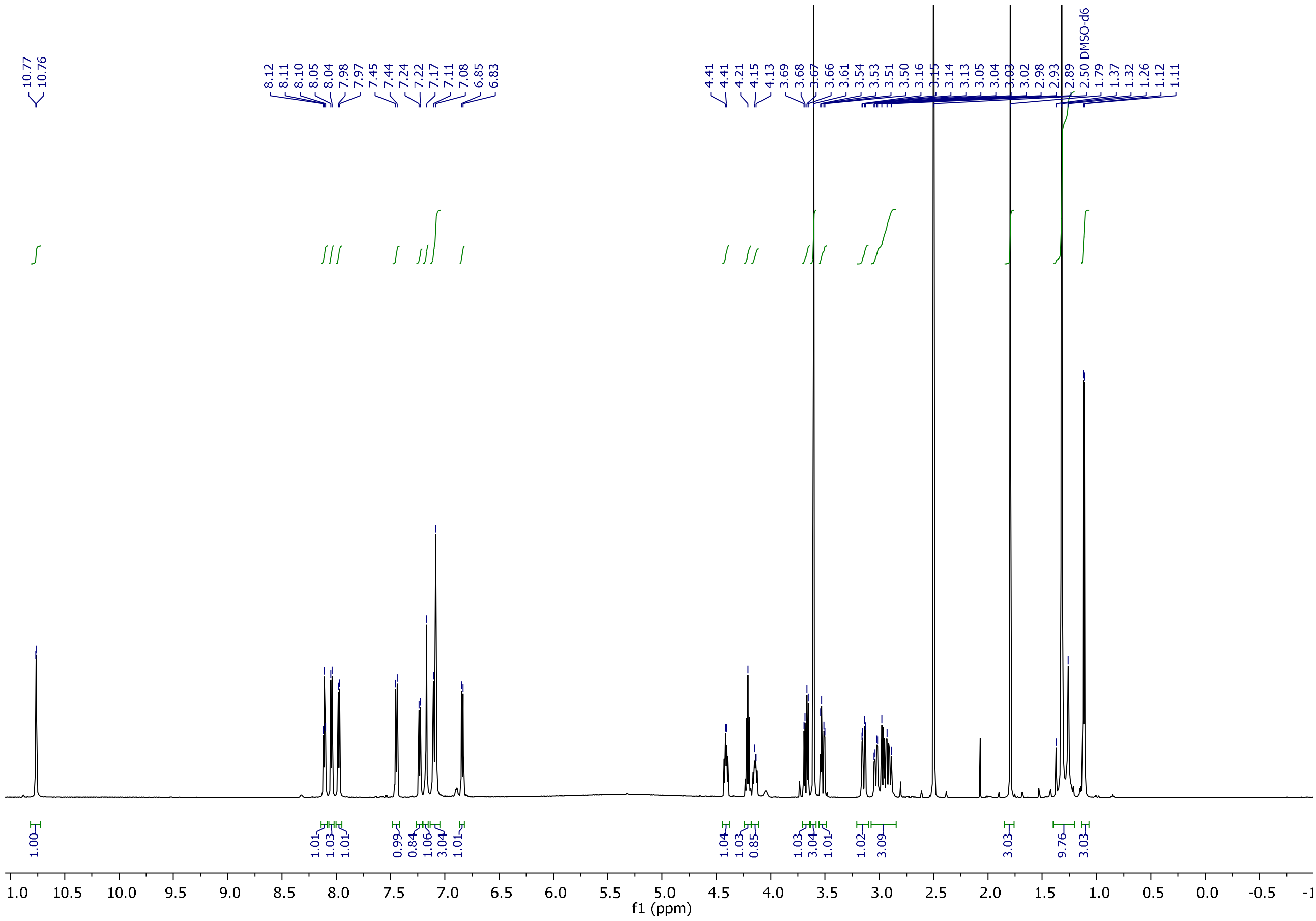
10.77
10.76
8.12
8.11
8.10
8.05
8.04
7.98
7.97
7.45
7.44
7.24
7.22
7.17
7.11
7.08
6.85
6.83
4.41
4.41
4.21
4.15
4.13
3.69
3.68
3.67
3.66
3.61
3.54
3.53
3.51
3.50
3.16
3.15
3.14
3.13
3.05
3.04
3.03
3.02
2.98
2.93
2.89
2.50 DMSO-d6
1.79
1.37
1.32
1.26
1.12
1.11
1.00
1.01
1.03
1.01
0.99
0.84
1.06
3.04
1.01
1.04
1.03
0.85
1.03
3.04
1.01
1.02
3.09
3.03
9.76
3.03
f1 (ppm)

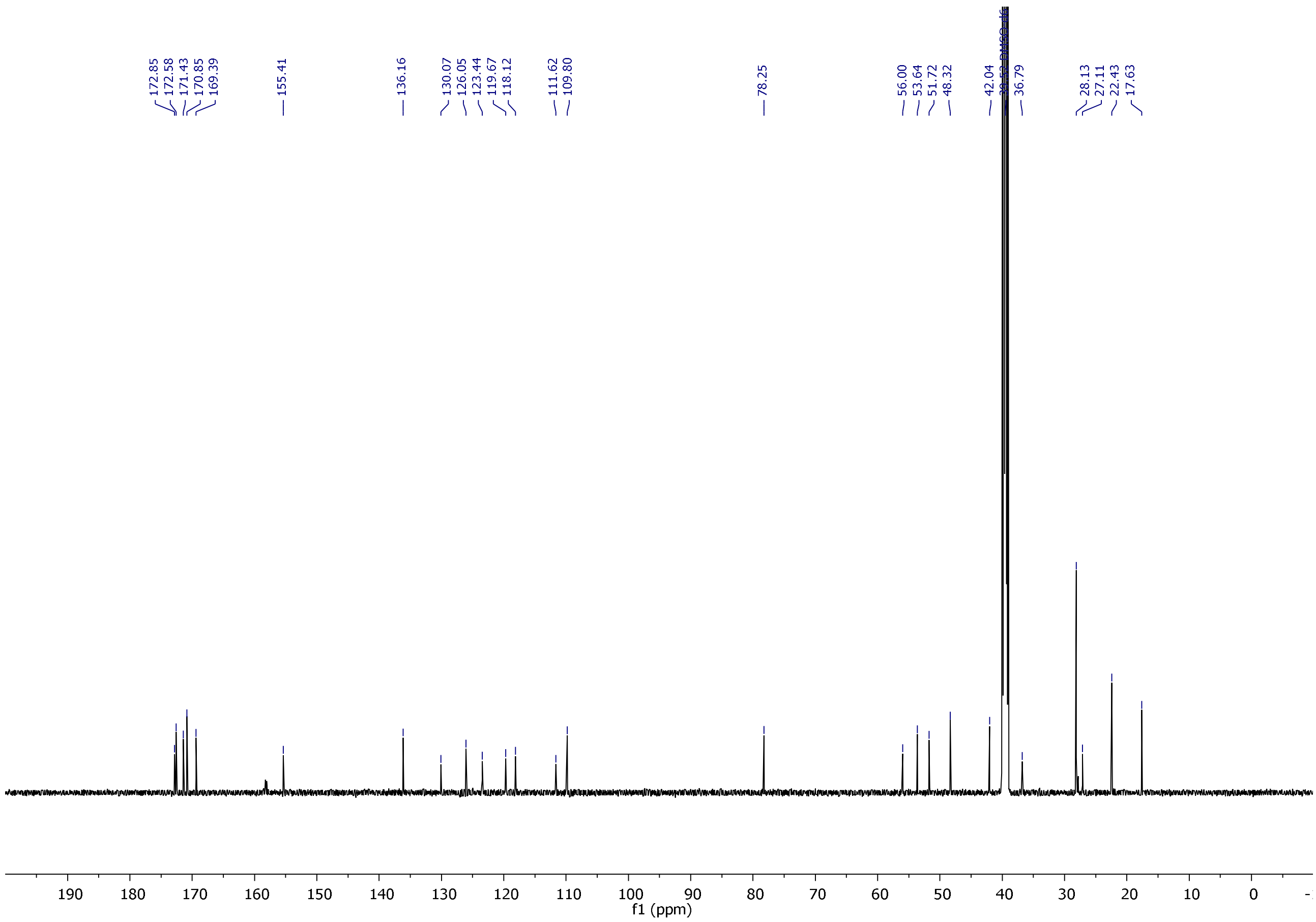
172.85
172.58
171.43
170.85
169.39
155.41
136.16
130.07
126.05
123.44
119.67
118.12
111.62
109.80
78.25
56.00
53.64
51.72
48.32
42.04
36.79
28.13
27.11
22.43
17.63
f1 (ppm)

UHPLC-MS chromatogram at λ = 220 nm

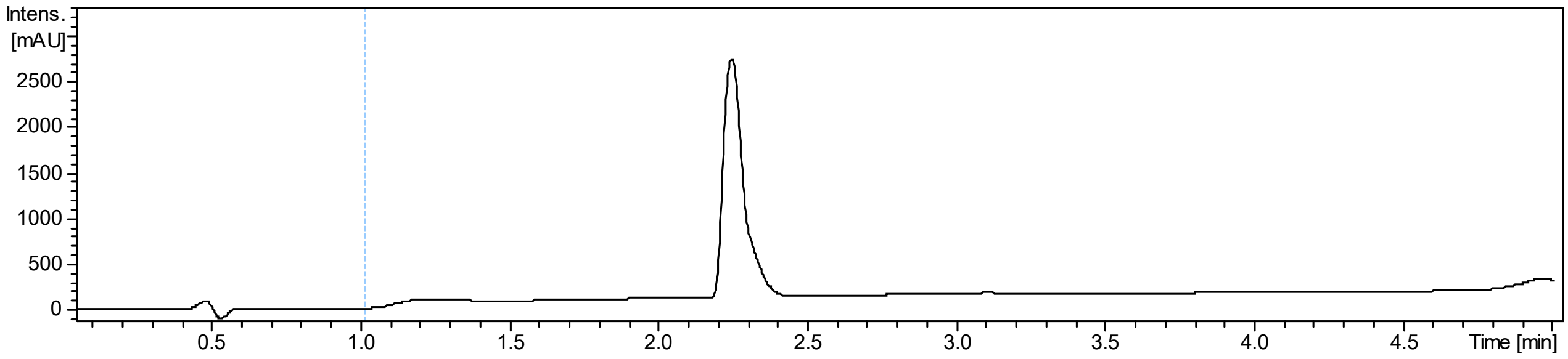

Peptide 6a

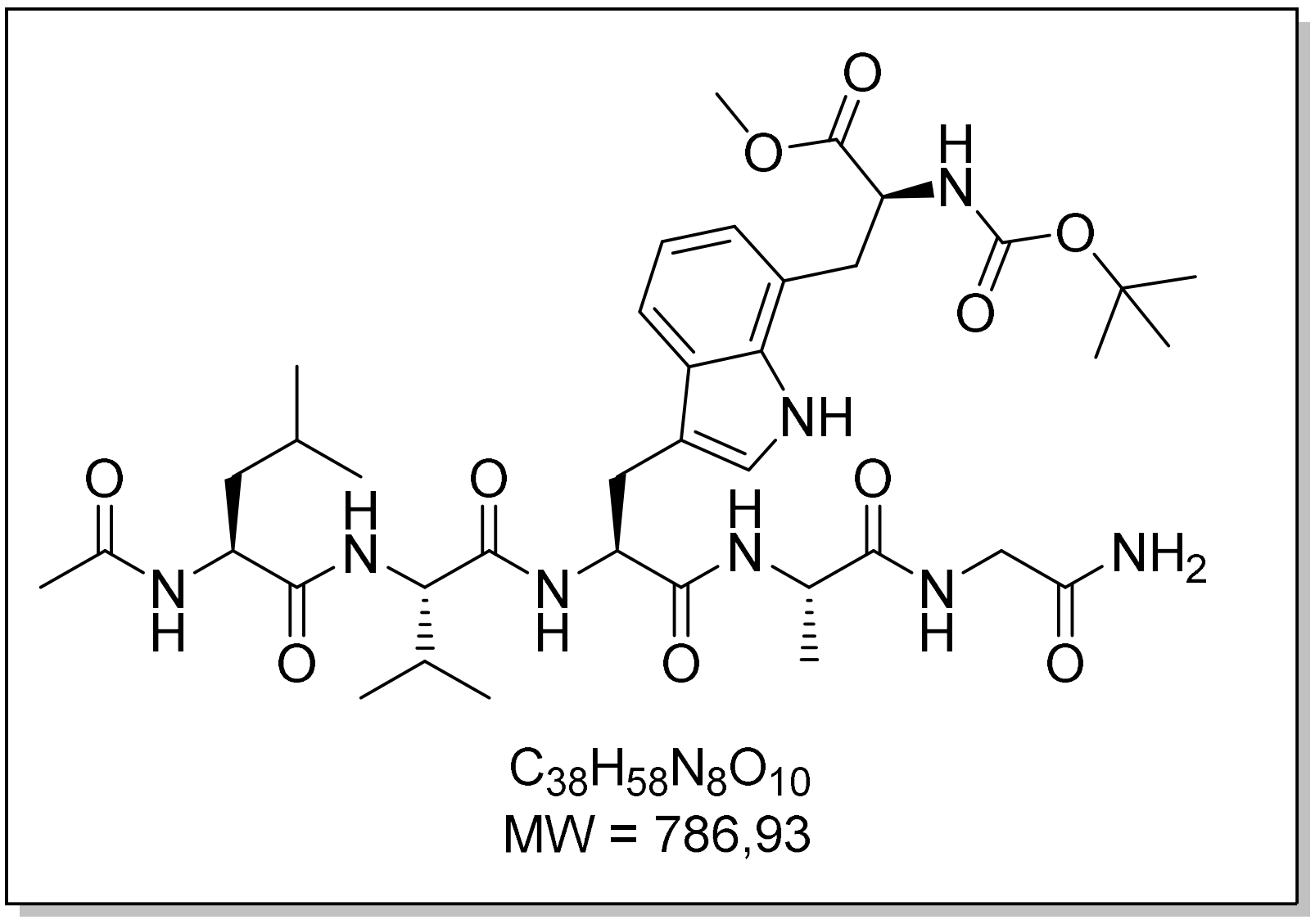

Peptide **6a** was synthesized according to GP1. Therefore, a stock solution of Ac-Leu-Val-Trp(7-Br)-Ala-Gly-NH_2 (0.200 M) was prepared in anhydrous DMSO and degassed under argon atmosphere for 30 min. Separately, activated zinc dust (8.89 mg, 134 µmol, 4.5 eq.), Pd(amphos)$_2$Cl$_2$ (2.38 mg, 3.36 µmol, 11 mol%) and N$^{\alpha}$-Boc-L-3-iodoalanine methyl ester (40.04 mg, 122 µmol, 4.1 eq.) were charged into a glass vial, which was subsequently placed under argon atmosphere. After 10 min anhydrous DMSO (150 µL), and the peptide stock solution (0.200 M, 150 µL, 1.0 eq.) were added sequentially to the vial. After every addition the reaction mixture purged with argon for 5 min. The vial was sealed and the suspension was vigorously stirred at 40 °C for 48 h. When the reaction time elapsed, the mixture was allowed to cool to room temperature, and centrifuged. The crude product was purified by preparative RP-HPLC (preparative run protocol 3). Lyophilization provided the product as a colourless solid (9.63 mg, 12.2 µmol, 41%).

^{1}H NMR (600 MHz, [D$_6$]DMSO): δ [ppm] = 10.78 (s, 1H; N**H**-indole), 8.12 (d, $^3J_{H\text{-}H}$ = 6.2 Hz, 1H; N**H**^{Ala}), 8.03 (m, 2H; N**H**^{Trp}, N**H**^{Leu}), 7.90 (m, 1H; N**H**^{Gly}), 7.62 (d, $^3J_{H\text{-}H}$ = 7.8 Hz, 1H; N**H**^{Val}), 7.45 (d, $^3J_{H\text{-}H}$ = 7.2 Hz, 1H; C4-**H**^{Trp}), 7.26 (d, $^3J_{H\text{-}H}$ = 7.5 Hz, 1H; N**H**^{Ala2}), 7.16 (m, 2H, N**H**$_2$; C2-**H**^{Trp}), 7.08 (s, 1H; N**H**$_2$), 6.90 (t, $^3J_{H\text{-}H}$ = 7.2 Hz, 1H; C5-**H**^{Trp}), 6.85 (d, $^3J_{H\text{-}H}$ = 7.2 Hz, 1H; C6-**H**^{Trp}), 4.53 (m, 1H; C$_{\alpha}$-**H**^{Trp}), 4.29 (m, 2H; C$_{\alpha}$-**H**^{Leu}, C$_{\alpha}$-**H**^{Ala2}), 4.20 (m, 1H; C$_{\alpha}$-**H**^{Ala}), 4.13 (t, $^3J_{H\text{-}H}$ = 7.8 Hz, 1H; C$_{\alpha}$-**H**^{Val}), 3.65-3.51 (m, 4H; C$_{\alpha}$-**H**$_2^{Gly}$, C**H**$_3$-OMe), 3.20-3.00 (m, 3H; C$_{\beta}$-**H**^{Trp}, C$_{\beta}$-**H**$_2^{Ala2}$), 2.93 (dd, $^2J_{H\text{-}H}$ = 14.4 Hz, $^3J_{H\text{-}H}$ = 8.3 Hz, 1H; C$_{\beta}$-**H**^{Trp}), 1.93 (m, 1H; C**H**^{Val}), 1.83 (s, 3H; C**H**$_3$-Ac), 1.57 (m, 1H; C**H**^{Leu}), 1.41 (m, 2H; C-**H**$_2^{Leu}$), 1.31 (s, 9H; 3C**H**$_3$-Boc), 1.18 (d, $^3J_{H\text{-}H}$ = 7.0 Hz, 3H; C**H**$_3^{Ala}$), 0.86 (d, $^3J_{H\text{-}H}$ = 6.2 Hz, 3H; C**H**$_3^{Leu}$), 0.81 (d, $^3J_{H\text{-}H}$ = 6.1 Hz, 3H; C**H**$_3^{Leu}$) 0.76 (d, $^3J_{H\text{-}H}$ = 7.1 Hz, 3H; C**H**$_3^{Val}$), 0.75 (d, $^3J_{H\text{-}H}$ = 7.0 Hz, 3H; C**H**$_3^{Val}$).

^{13}C NMR (151 MHz, DMSO): δ [ppm] = 172.8 (**C**=O^{Ala2}), 172.1 (**C**=O^{Ala}), 172.0 (**C**=O^{Leu}), 171.3 (**C**=O^{Trp}), 170.8 (**C**=O^{Val}, **C**=O^{Gly}), 169.2 (CH$_3$**C**=O), 155.4 (NH**C**=O), 135.2 (**C**7a), 127.4 (**C**3a), 123.5 (**C**2), 121.3 (**C**6), 120.3 (**C**3), 118.3 (**C**5), 117.0 (**C**4), 110.3 (**C**7), 78.3 (**C**-Boc), 57.3 (**C**$_{\alpha}^{Val}$), 53.9 (**C**$_{\alpha}^{Ala2}$), 53.2 (**C**$_{\alpha}^{Trp}$), 51.6 (**C**H$_3$-OMe), 51.1 (**C**$_{\alpha}^{Leu}$), 48.6 (**C**$_{\alpha}^{Ala}$), 41.9 (**C**$_{\alpha}^{Gly}$), 40.4 (**C**$_{\beta}^{Leu}$), 32.2 (**C**$_{\beta}^{Ala2}$),

30.8 (C_{β}^{Val}), 28.1 (3**C**H_3-Boc), 27.3 (C_{β}^{Trp}), 24.2 (C_{γ}^{Leu}), 23.0 (**C**H_3^{Leu}), 22.5 (**C**H_3C=O), 21.6 (**C**H_3^{Leu}), 19.1 (**C**H_3^{Val}), 17.9 (**C**H_3^{Val}), 17.7 (**C**H_3^{Ala}).

HRMS (ESI): *m/z* calcd: 787.4349 $[M+H]^+$, 804.4614 $[M+NH_4]^+$, 809.4168 $[M+Na]^+$; found: 787.4356 $[M+H]^+$, 804.4595 $[M+NH_4]^+$, 809.4172 $[M+Na]^+$.

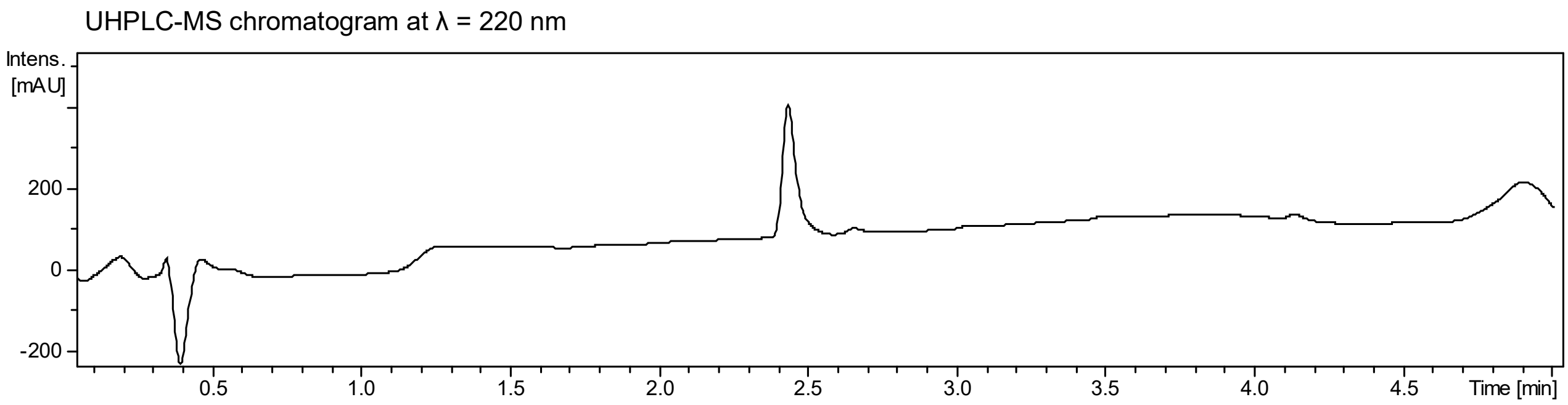

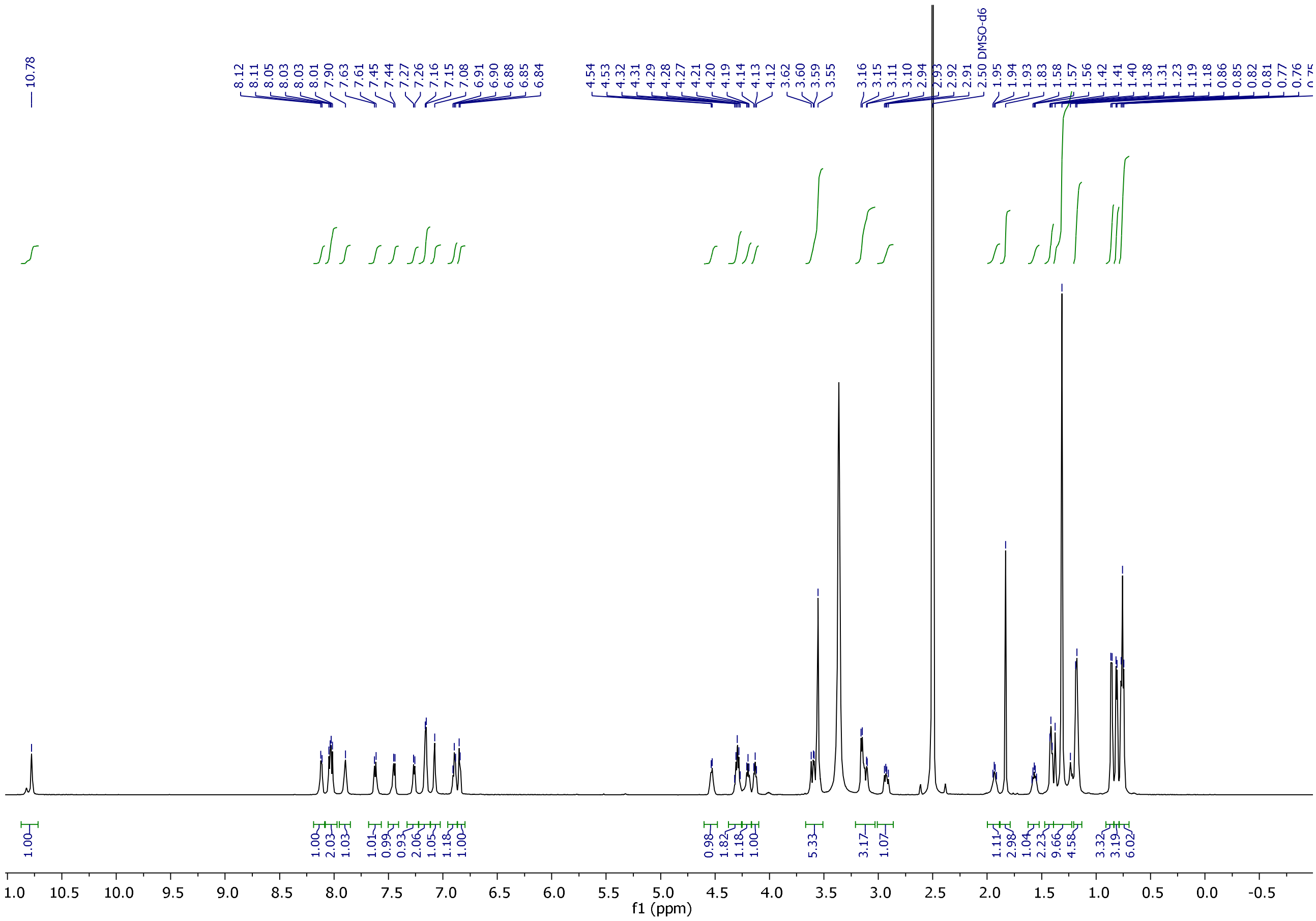
f1 (ppm)
DMSO-d6

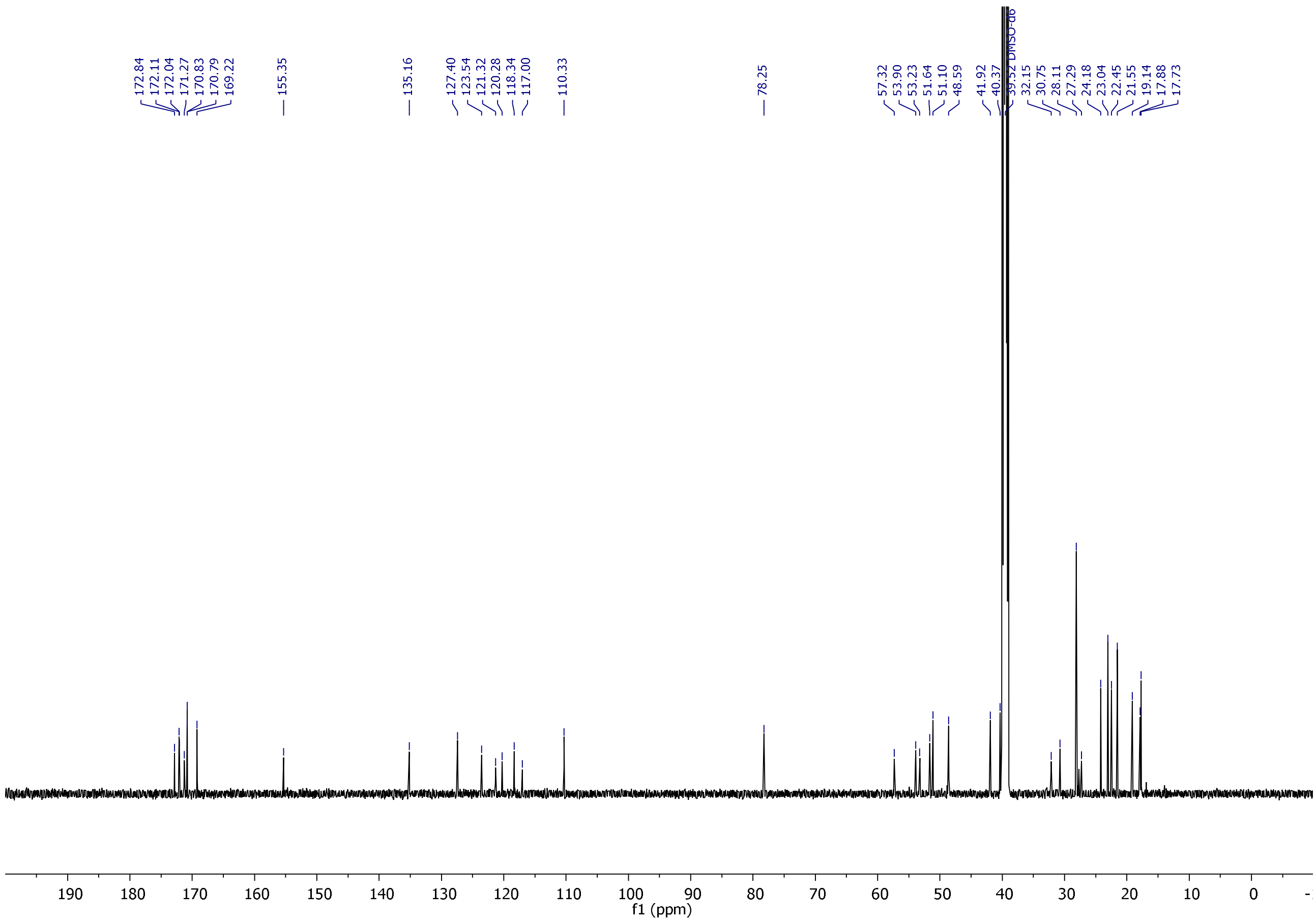
f1 (ppm)
DMSO-d6

Ac-Leu-Val-Trp(7-benzyl)-Ala-Gly-NH2 (6b)

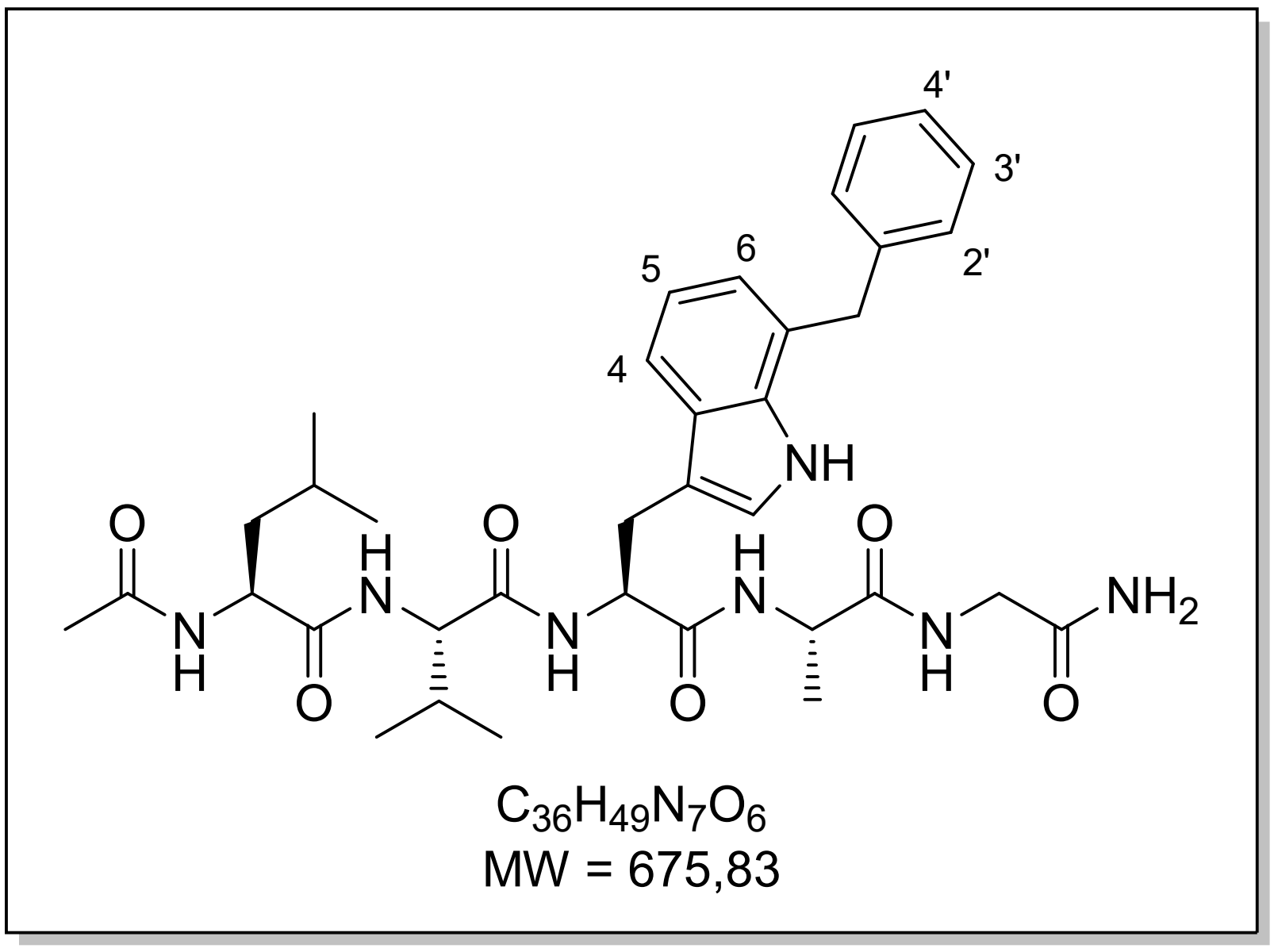

Ac-Leu-Val-Trp(7-benzyl)-Ala-Gly-NH_2 (**6b**) was synthesized according to GP1. Therefore, a stock solution of Ac-Leu-Val-Trp(7-Br)-Ala-Gly-NH_2 (0.200 M) was prepared in anhydrous DMSO and degassed under argon atmosphere for 30 min. Separately, activated zinc dust (8.26 mg, 126 µmol, 4.2 eq.), Pd(amphos)$_2$$Cl_2$ (2.19 mg, 3.09 µmol, 10 mol%) and benzyl iodide (39.67 mg, 182 µmol, 6.0 eq.) were charged into a glass vial, which was subsequently placed under argon atmosphere. After 10 min anhydrous DMSO (150 µL), and the peptide stock solution (0.200 M, 150 µL, 1.0 eq.) was added to the vial. After every addition the reaction mixture purged with argon for 5 min. The vial was sealed and the suspension was vigorously stirred at 40 °C for 48 h. When the reaction time elapsed, the mixture was allowed to cool to room temperature, and centrifuged. The crude product was purified by preparative RP-HPLC (preparative run protocol 3). Lyophilization provided the product as a colourless solid (9.24 mg, 13.7 µmol, 46%).

^{1}H NMR (600 MHz, [D$_6$]DMSO): δ [ppm] = 10.86 (d, 1H, $^3J_{H-H}$ = 2.1 Hz; N**H**-indole), 8.10 (d, $^3J_{H-H}$ = 6.8 Hz, 1H; N**H**^{Ala}), 8.02 (m, 2H; N**H**^{Trp}, N**H**^{Leu}), 7.86 (t, $^3J_{H-H}$ = 5.7 Hz, 1H; N**H**^{Gly}), 7.64 (d, $^3J_{H-H}$ = 8.7 Hz, 1H; N**H**^{Val}), 7.42 (d, $^3J_{H-H}$ = 7.5 Hz, 1H; C4-**H**^{Trp}), 7.29 (m, 2H; C2'-**H**/C6'-**H**), 7.24 (m, 2H; C3'-**H**/C5'-**H**), 7.18-7.13 (m, 3H; C4'-**H**, N**H**$_2$, C2-**H**^{Trp}), 7.08 (s, 1H; N**H**$_2$), 6.90 (t, $^3J_{H-H}$ = 7.5 Hz, 1H; C5-**H**^{Trp}), 6.82 (d, $^3J_{H-H}$ = 7.5 Hz, 1H; C6-**H**^{Trp}), 4.54 (m, 1H; C$_\alpha$-**H**^{Trp}), 4.29 (m, 1H; C$_\alpha$-**H**^{Leu}), 4.19 (m, 1H; C$_\alpha$-**H**^{Ala}), 4.14 (s, 2H; C**H**$_2$), 4.12 (dd, $^3J_{H-H}$ = 8.7 Hz, $^3J_{H-H}$ = 6.8 Hz, 1H; C$_\alpha$-**H**^{Val}), 3.60 (dd, $^2J_{H-H}$ = 16.8 Hz, $^3J_{H-H}$ = 5.7 Hz, 1H; C$_\alpha$-**H**^{Gly}), 3.54 (dd, $^2J_{H-H}$ = 16.8 Hz, $^3J_{H-H}$ = 5.7 Hz, 1H; C$_\alpha$-**H**^{Gly}), 3.11 (dd, $^2J_{H-H}$ = 14.8 Hz, $^3J_{H-H}$ = 6.0 Hz, 1H; C$_\beta$-**H**^{Trp}), 2.93 (dd, $^2J_{H-H}$ = 14.8 Hz, $^3J_{H-H}$ = 8.2 Hz, 1H; C$_\beta$-**H**^{Trp}), 1.92 (m, 1H; C**H**^{Val}), 1.83 (s, 3H; C**H**$_3$-Ac), 1.57 (m, 1H; C**H**^{Leu}), 1.41 (m, 2H, C-**H**$_2$^{Leu}), 1.17 (d, $^3J_{H-H}$ = 7.1 Hz, 3H; C**H**$_3$^{Ala}), 0.86 (d, $^3J_{H-H}$ = 6.6 Hz, 3H; C**H**$_3$^{Leu}), 0.81 (d, $^3J_{H-H}$ = 6.6 Hz, 3H; C**H**$_3$^{Leu}), 0.74 (d, $^3J_{H-H}$ = 6.8 Hz, 6H; C**H**$_3$^{Val}).

^{13}C NMR (151 MHz, [D$_6$]DMSO): δ [ppm] = 172.1 (**C**=O^{Ala}), 172.0 (**C**=O^{Leu}), 171.2 (**C**=O^{Trp}), 170.8 (**C**=O^{Val}, **C**=O^{Gly}), 169.2 (CH$_3$**C**=O), 140.7 (**C**1'), 134.9 (**C**7a), 128.7 (**C**2'/**C**6'), 128.2 (**C**3'/**C**5'), 127.5 (**C**3a), 125.8 (**C**4'), 124.1 (**C**7), 123.5 (**C**2), 121.1 (**C**6), 118.6 (**C**5), 116.5 (**C**4), 110.4 (**C**3), 57.4 (**C**$_\alpha$^{Val}), 53.2 (**C**$_\alpha$^{Trp}), 51.1 (**C**$_\alpha$^{Leu}), 48.6 (**C**$_\alpha$^{Ala}), 41.9 (**C**$_\alpha$^{Gly}), 40.4 (**C**$_\beta$^{Leu}), 36.3 (**C**H$_2$), 30.7 (**C**$_\beta$^{Val}), 27.4

(C_{β}^{Trp}), 24.2 (C_{γ}^{Leu}), 23.0 (**C**H_3^{Leu}), 22.5 (**C**H_3C=O), 21.6 (**C**H_3^{Leu}), 19.1 (**C**H_3^{Val}), 17.9 (**C**H_3^{Val}), 17.7 (**C**H_3^{Ala}).

<u>HRMS</u> (ESI): *m/z* calcd: 676.3817 $[M+H]^+$, 693.4083 $[M+NH_4]^+$, 698.3637 $[M+Na]^+$, 714.3376 $[M+K]^+$; found: 676.3820 $[M+H]^+$, 693.4084 $[M+NH_4]^+$, 698.3634 $[M+Na]^+$, 714.3374 $[M+K]^+$.

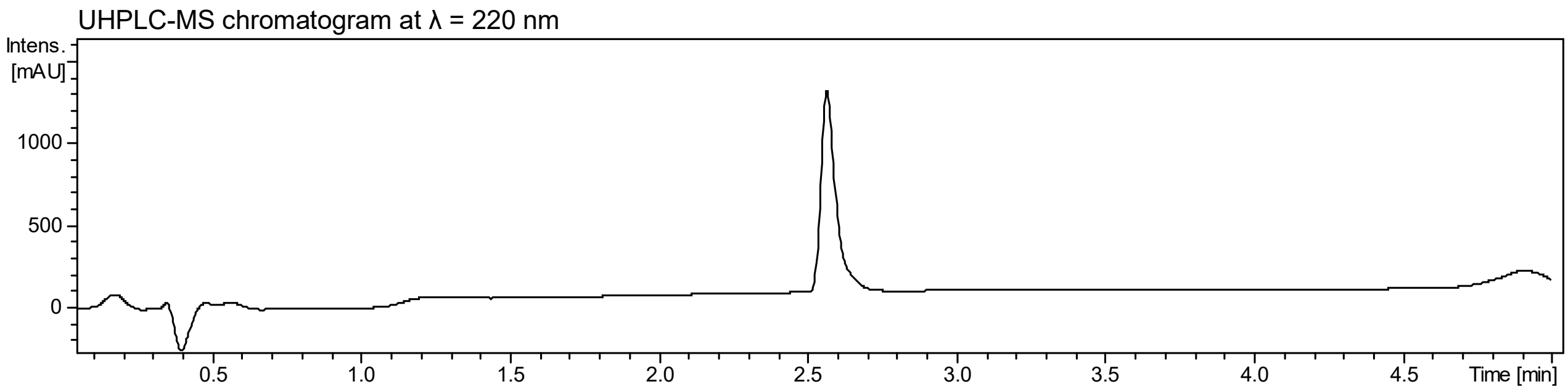

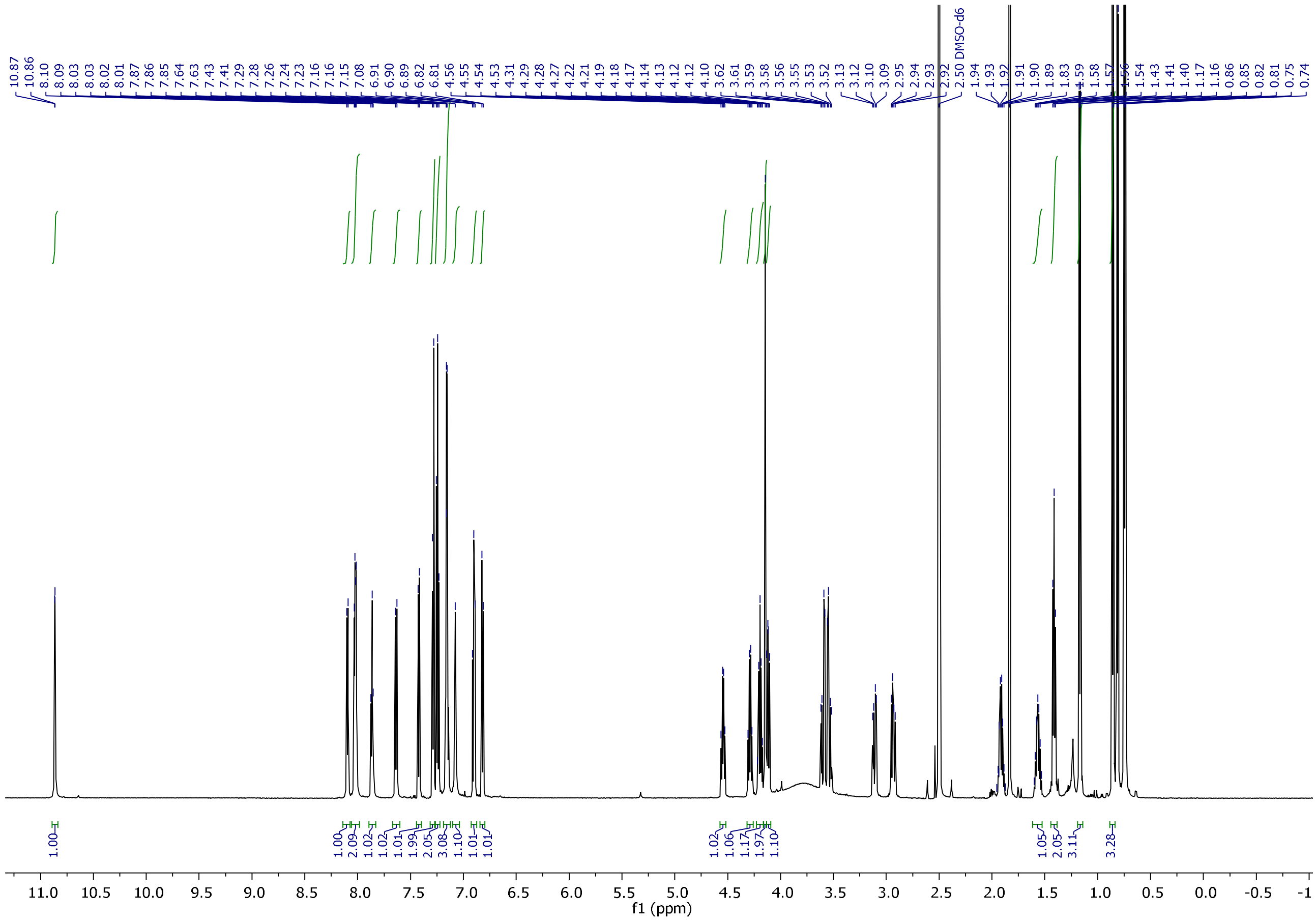

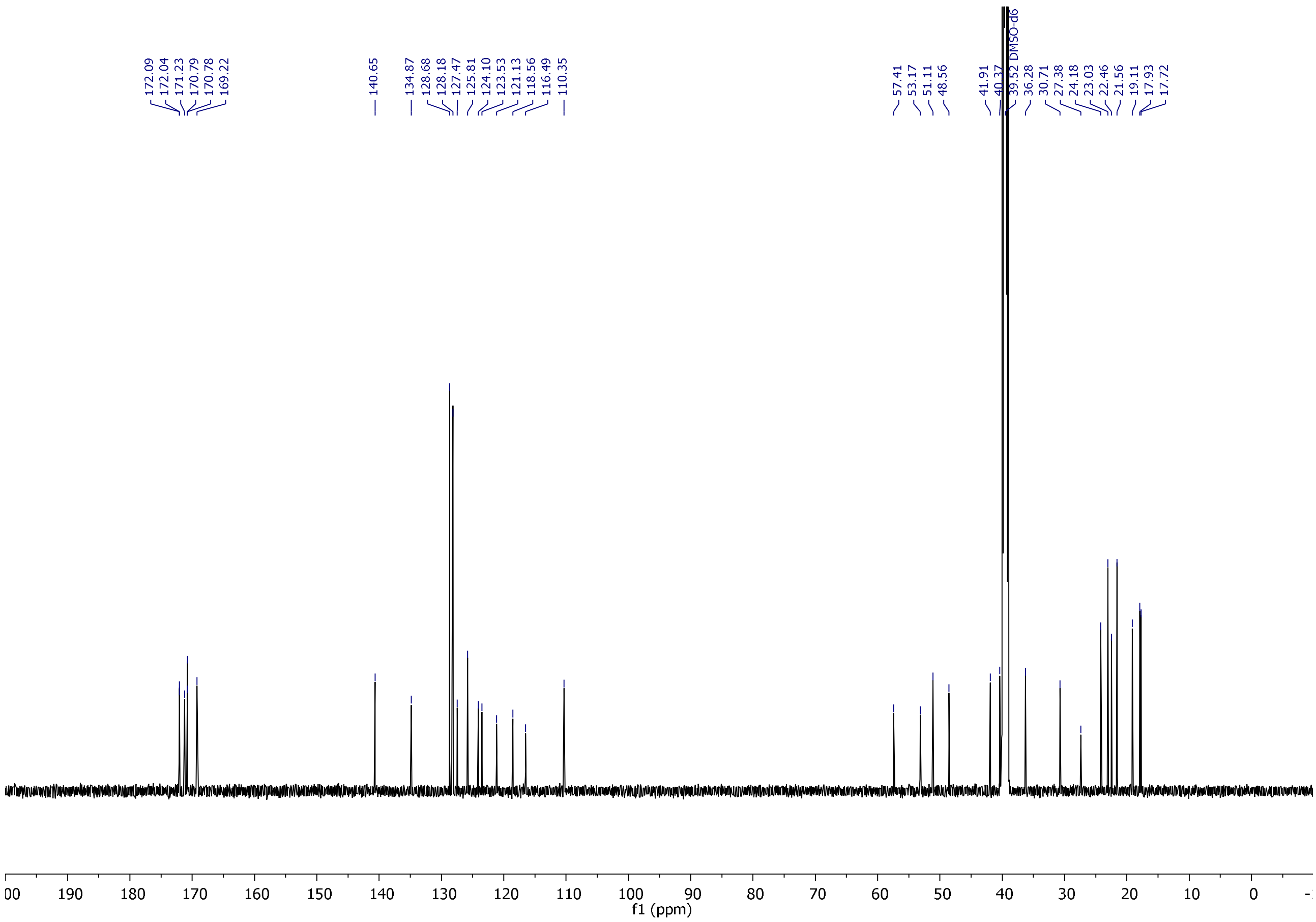

Peptide 7a

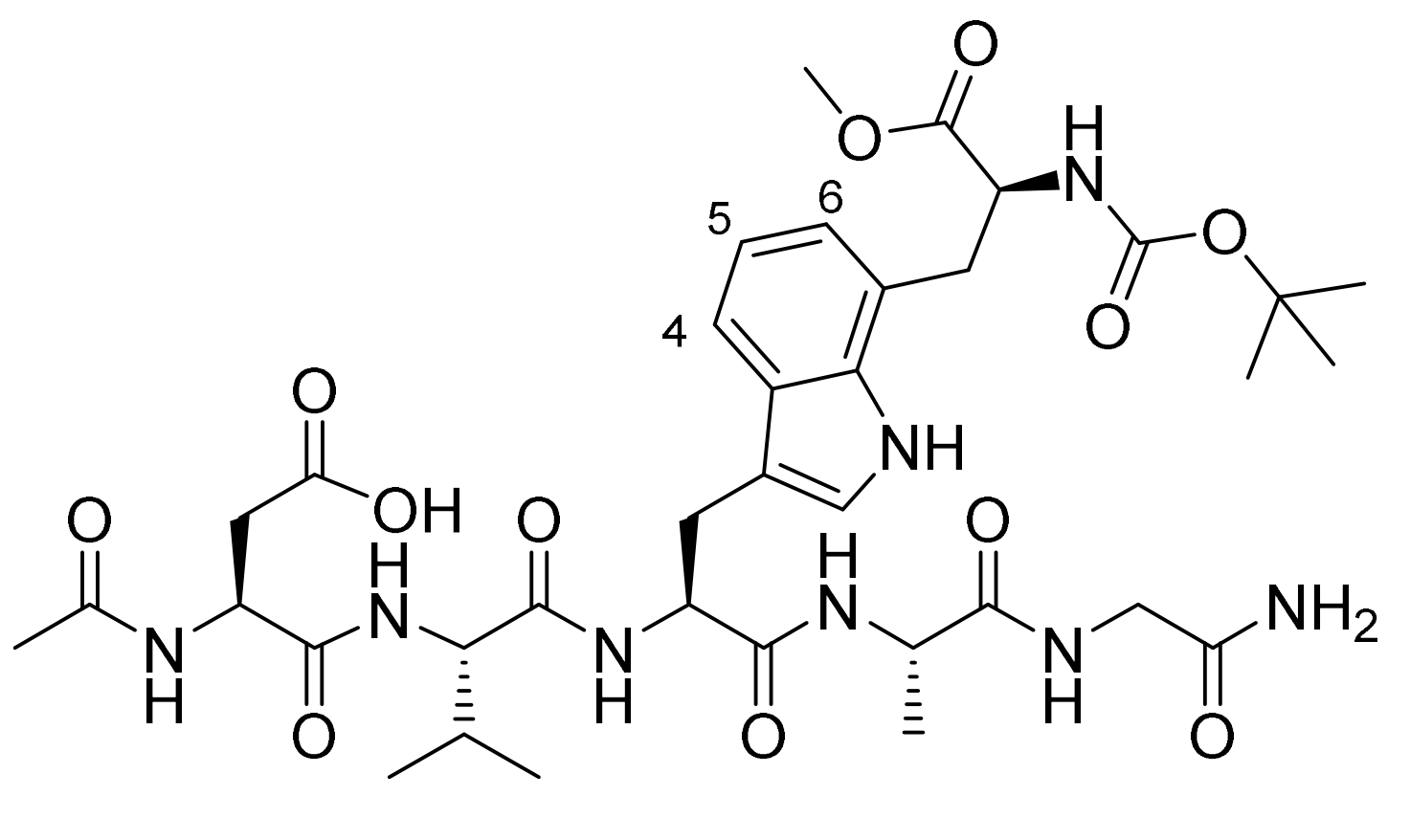

Peptide **7a** was synthesized according to GP1. Therefore, a stock solution of Ac-Asp-Val-Trp(7-Br)-Ala-Gly-NH_2 (0.200 M) was prepared in anhydrous DMSO and degassed under argon atmosphere for 30 min. Separately, activated zinc dust (8.37 mg, 128 µmol, 4.3 eq.), $Pd(amphos)_2Cl_2$ (2.21 mg, 3.12 µmol, 10 mol%) and N^{α}-Boc-L-3-iodoalanine methyl ester (39.95 mg, 121 µmol, 4.0 eq.) were charged into a glass vial, which was subsequently placed under argon atmosphere. After 10 min anhydrous DMSO (150 µL), and the peptide stock solution (0.200 M, 150 µL, 1.0 eq.) were added sequentially to the vial. After every addition the reaction mixture purged with argon for 5 min. The vial was sealed and the suspension was vigorously stirred at 40 °C for 48 h. When the reaction time elapsed, the mixture was allowed to cool to room temperature, and centrifuged. The crude product was purified by preparative RP-HPLC (preparative run protocol 3). Lyophilization provided the product as a colourless solid (5.81 mg, 7.37 µmol, 25%).

^{1}H NMR (600 MHz, [D_8]DMSO): *δ* [ppm] = 12.31 (s br, 1H; COO**H**^{Asp}), 10.78 (s, 1H; N**H**-indole), 8.26 (d, $^3J_{H\text{-}H}$ = 7.8 Hz, 1H; N**H**^{Asp}), 8.07 (d, $^3J_{H\text{-}H}$ = 6.7 Hz, 1H; N**H**^{Ala}), 8.03 (d, $^3J_{H\text{-}H}$ = 7.6 Hz, 1H; N**H**^{Trp}), 7.89 (t, $^3J_{H\text{-}H}$ = 5.8 Hz, 1H; N**H**^{Gly}), 7.58 (d, $^3J_{H\text{-}H}$ = 8.4 Hz, 1H; N**H**^{Val}), 7.46 (d, $^3J_{H\text{-}H}$ = 7.5 Hz, 1H; C4-**H**^{Trp}), 7.26 (d, $^3J_{H\text{-}H}$ = 7.9 Hz, 1H; N**H**^{Ala2}), 7.15 (s, 2H, N**H**$_2$; C2-**H**^{Trp}), 7.07 (s, 1H; N**H**$_2$), 6.90 (t, $^3J_{H\text{-}H}$ = 7.5 Hz, 1H; C5-**H**^{Trp}), 6.85 (d, $^3J_{H\text{-}H}$ = 7.5 Hz, 1H; C6-**H**^{Trp}), 4.59 (m, 1H; C_{α}-**H**^{Asp}), 4.53 (m, 1H; C_{α}-**H**^{Trp}), 4.30 (m, 1H; C_{α}-**H**^{Ala2}), 4.21 (m, 1H; C_{α}-**H**^{Ala}), 4.11 (dd, $^3J_{H\text{-}H}$ = 8.4 Hz, $^3J_{H\text{-}H}$ = 6.0 Hz, 1H; C_{α}-**H**^{Val}), 3.61 (dd, $^2J_{H\text{-}H}$ = 16.8 Hz, $^3J_{H\text{-}H}$ = 5.8 Hz, 1H; C_{α}-**H**^{Gly}), 3.58-3.52 (m, 4H, C_{α}-**H**^{Gly}; C**H**$_3$-OMe), 3.19-3.09 (m, 3H; C_{β}-**H**^{Trp}, C_{β}-**H**$_2^{Ala2}$), 2.93 (dd, $^2J_{H\text{-}H}$ = 14.8 Hz, $^3J_{H\text{-}H}$ = 8.9 Hz, 1H; C_{β}-**H**^{Trp}), 2.67 (dd, $^2J_{H\text{-}H}$ = 16.6 Hz, $^3J_{H\text{-}H}$ = 5.8 Hz, 1H; C_{β}-**H**^{Asp}), 2.47 (m, 1H; C_{β}-**H**^{Asp}), 1.93 (m, 1H; C**H**^{Val}), 1.83 (s, 3H; C**H**$_3$-Ac), 1.31 (s, 9H; 3C**H**$_3$-Boc), 1.20 (d, $^3J_{H\text{-}H}$ = 7.1 Hz, 3H; C**H**$_3^{Ala}$), 0.74 (d, $^3J_{H\text{-}H}$ = 6.8 Hz, 3H; C**H**$_3^{Val}$), 0.69 (d, $^3J_{H\text{-}H}$ = 6.8 Hz, 3H; C**H**$_3^{Val}$).

^{13}C NMR (151 MHz, DMSO): *δ* [ppm] = 172.9 (**C**=O^{Ala2}), 172.1 (**C**=O^{Ala}), 171.9 (**C**OOH^{Asp}) 171.4 (**C**=O^{Trp}), 170.8 (**C**=O^{Gly}), 170.7 (**C**=O^{Asp}), 170.7 (**C**=O^{Val}), 169.6 (CH_3**C**=O), 155.4 (NH**C**=O), 135.2 (**C**7a), 127.4 (**C**3a), 123.5 (**C**2), 121.3 (**C**6), 120.3 (**C**3), 118.4 (**C**5), 117.0 (**C**4), 110.4 (**C**7), 78.3 (**C**-Boc), 57.5 (**C**$_{\alpha}^{Val}$), 53.9 (**C**$_{\alpha}^{Ala2}$), 53.3 (**C**$_{\alpha}^{Trp}$), 51.7 (**C**H_3-OMe), 49.4 (**C**$_{\alpha}^{Asp}$), 48.6 (**C**$_{\alpha}^{Ala}$), 41.9 (**C**$_{\alpha}^{Gly}$),

35.6 (C_{β}^{Asp}), 32.2 (C_{β}^{Ala2}), 30.7 (C_{β}^{Val}), 28.1 (3**C**H_3-Boc), 27.2 (C_{β}^{Trp}), 22.4 (**C**H_3C=O), 19.1 (**C**H_3^{Val}), 17.7 (**C**H_3^{Ala}), 17.5 (**C**H_3^{Val}).

HRMS (ESI): *m/z* calcd: 789.3778 $[M+H]^+$, 811.3597 $[M+Na]^+$; found: 789.3778 $[M+H]^+$, 811.3619 $[M+Na]^+$.

UHPLC-MS chromatogram at λ = 220 nm

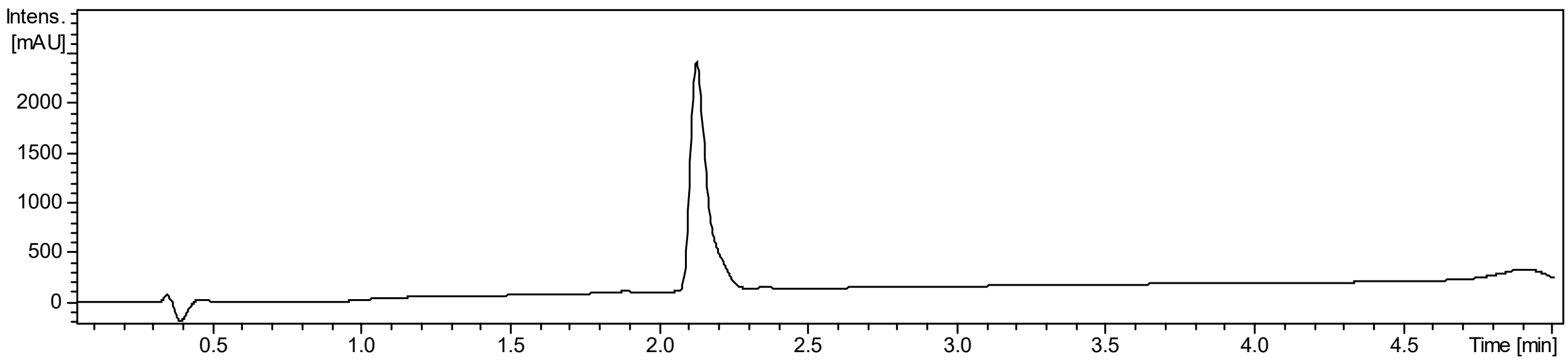

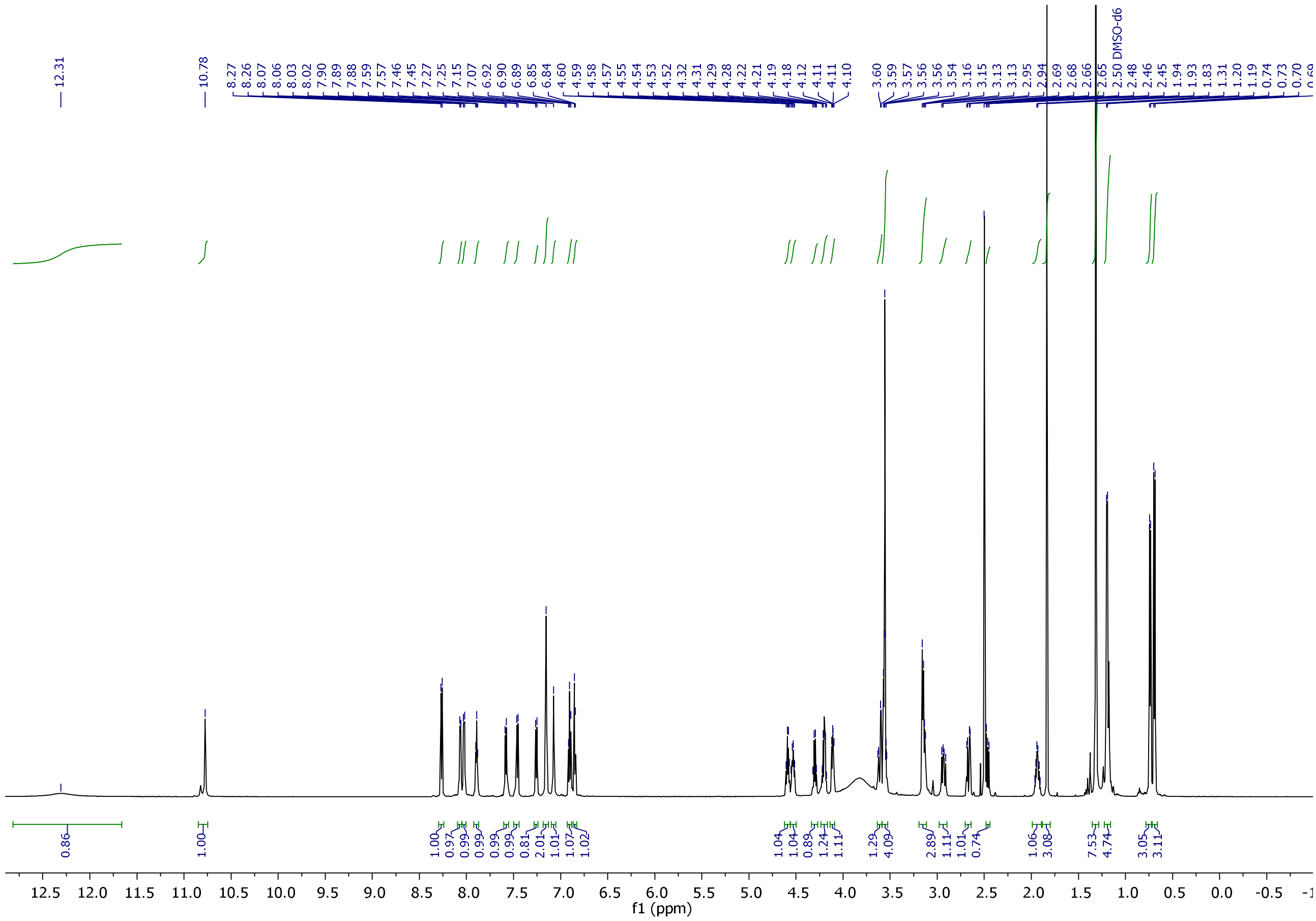

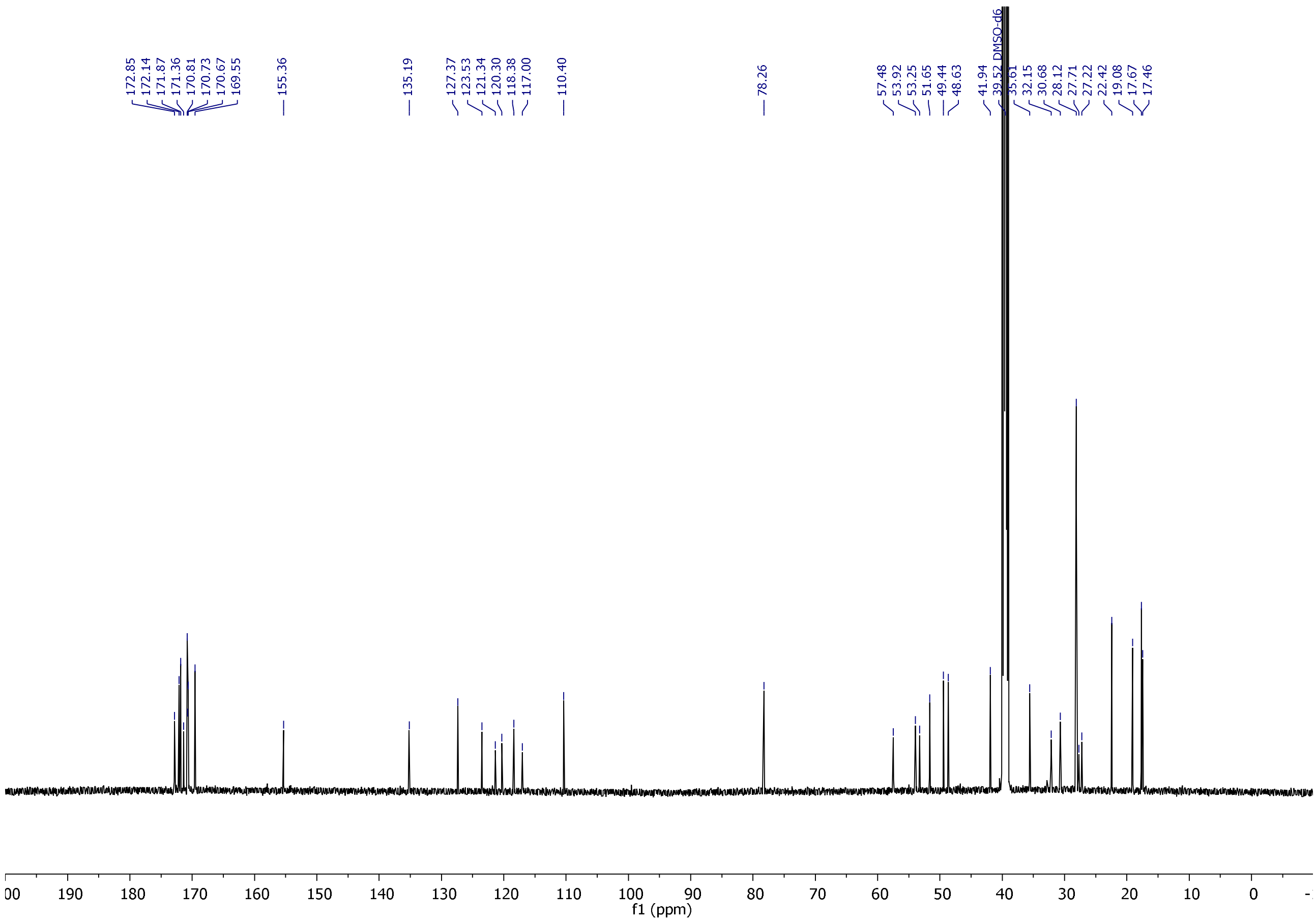

Peptide 8a

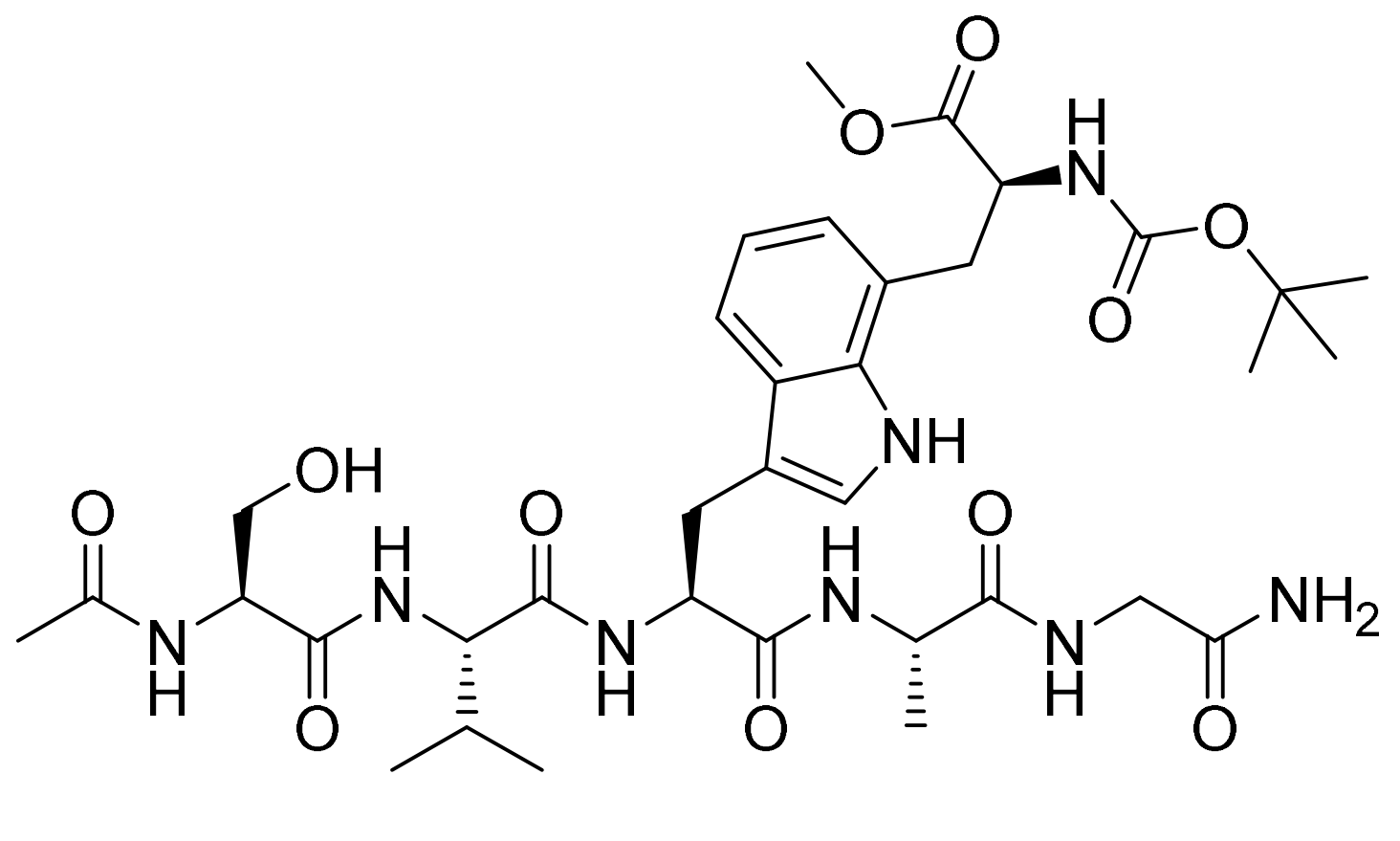

Peptide **8a** was synthesized according to GP1. Therefore, a stock solution of Ac-Ser-Val-Trp(7-Br)-Ala-Gly-NH_2 (0.162 M) was prepared in anhydrous DMSO and degassed under argon atmosphere for 30 min. Separately, activated zinc dust (7.54 mg, 115 µmol, 4.8 eq.), $Pd(amphos)_2Cl_2$ (2.65 mg, 3.74 µmol, 15 mol%) and N^{α}-Boc-L-3-iodoalanine methyl ester (34.31 mg, 104 µmol, 4.3 eq.) were charged into a glass vial, which was subsequently placed under argon atmosphere. After 10 min anhydrous DMSO (150 µL), and the peptide stock solution (0.162 M, 150 µL, 1.0 eq.) were added sequentially to the vial. After every addition the reaction mixture purged with argon for 5 min. The vial was sealed and the suspension was vigorously stirred at 40 °C for 48 h. When the reaction time elapsed, the mixture was allowed to cool to room temperature, and centrifuged. The crude product was purified by preparative RP-HPLC (preparative run protocol 3). Lyophilization provided the product as a colourless solid (8.28 mg, 10.9 µmol, 45%).

^{1}H NMR (600 MHz, [D$_6$]DMSO): *δ* [ppm] = 10.77 (s, 1H; N**H**-indole), 8.05 (d, $^3J_{H-H}$ = 6.7 Hz, 1H; N**H**^{Ala}), 8.01 (d, ^3J = 7.7 Hz, 1H; N**H**^{Trp}), 7.98 (d, ^3J = 7.8 Hz, 1H; N**H**^{Ser}), 7.89 (t, $^3J_{H-H}$ = 7.4 Hz, 1H; N**H**^{Gly}), 7.73 (d, $^3J_{H-H}$ = 8.2 Hz, 1H; N**H**^{Val}), 7.45 (d, $^3J_{H-H}$ = 7.5 Hz, 1H; C4-**H**^{Trp}), 7.23 (d, $^3J_{H-H}$ = 7.9 Hz, 1H; N**H**^{Ala2}), 7.16 (s, 1H; N**H**$_2$), 7.14 (d, $^3J_{H-H}$ = 1.5 Hz, 1H; C2-**H**^{Trp}), 7.08 (s, 1H; N**H**$_2$), 6.90 (t, $^3J_{H-H}$ = 7.5 Hz, 1H; C5-**H**^{Trp}), 6.85 (d, $^3J_{H-H}$ = 7.5 Hz, 1H; C6-**H**^{Trp}), 4.53 (m, 1H; C_α-**H**^{Trp}), 4.38 (m, 1H; C_α-**H**^{Ser}), 4.30 (m, 1H; C_α-**H**^{Ala2}), 4.20 (m, 1H; C_α-**H**^{Ala}), 4.12 (dd, $^3J_{H-H}$ = 8.2 Hz, $^3J_{H-H}$ = 6.0 Hz, 1H; C_α-**H**^{Val}), 3.63 (dd, $^2J_{H-H}$ = 16.9 Hz, $^3J_{H-H}$ = 7.4 Hz, 1H; C_α-**H**^{Gly}), 3.59-3.52 (m, 6H; C_α-**H**^{Gly}, C_β-**H**$_2^{Ser}$, C**H**$_3$-OMe), 3.19-3.09 (m, 3H; C_β-**H**^{Trp}, C_β-**H**$_2^{Ala2}$), 2.89 (dd, $^2J_{H-H}$ = 14.9 Hz, $^3J_{H-H}$ = 9.2 Hz, 1H; C_β-**H**^{Trp}), 1.95 (m, 1H; C**H**^{Val}), 1.86 (s, 3H; C**H**$_3$-Ac), 1.31 (s, 9H; 3C**H**$_3$-Boc), 1.21 (d, $^3J_{H-H}$ = 7.1 Hz, 3H; C**H**$_3^{Ala}$), 0.76 (d, $^3J_{H-H}$ = 6.8 Hz, 3H; C**H**$_3^{Val}$), 0.69 (d, $^3J_{H-H}$ = 6.80 Hz, 3H; C**H**$_3^{Val}$).

^{13}C NMR (151 MHz, DMSO): *δ* [ppm] = 172.9 (**C**=O^{Ala2}), 172.1 (**C**=O^{Ala}), 171.4 (**C**=O^{Trp}), 170.8 (**C**=O^{Val}, **C**=O^{Gly}), 170.6 (**C**=O^{Ser}), 169.4 (CH_3**C**=O), 155.4 (NH**C**=O), 135.2 (**C**7a), 127.3 (**C**3a), 123.5 (**C**2), 121.3 (**C**6), 120.3 (**C**3), 118.4 (**C**5), 117.0 (**C**4), 110.4 (**C**7), 78.3 (**C**-Boc), 61.7 (**C**$_\beta^{Ser}$), 57.7 (**C**$_\alpha^{Val}$), 54.8 (**C**$_\alpha^{Ser}$), 53.9 (**C**$_\alpha^{Ala2}$), 53.3 (**C**$_\alpha^{Trp}$), 51.6 (**C**H_3-OMe), 48.7 (**C**$_\alpha^{Ala}$), 41.9 (**C**$_\alpha^{Gly}$), 32.2 (**C**$_\beta^{Ala2}$), 30.3 (**C**$_\beta^{Val}$), 28.1 (3**C**H_3-Boc), 27.3 (**C**$_\beta^{Trp}$), 22.5 (**C**H_3C=O), 19.1 (**C**H_3^{Val}), 17.7 (**C**H_3^{Ala}), 17.5 (**C**H_3^{Val}).

HRMS (ESI): *m/z* calcd: 783.3648 [M+Na]$^+$; found: 783.3645 [M+Na]$^+$.

UHPLC-MS chromatogram at λ = 220 nm

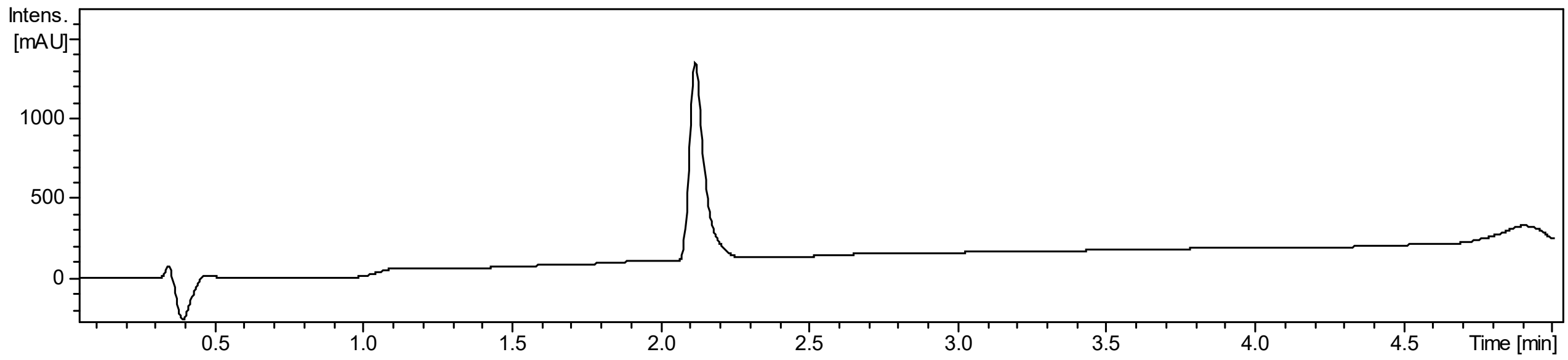

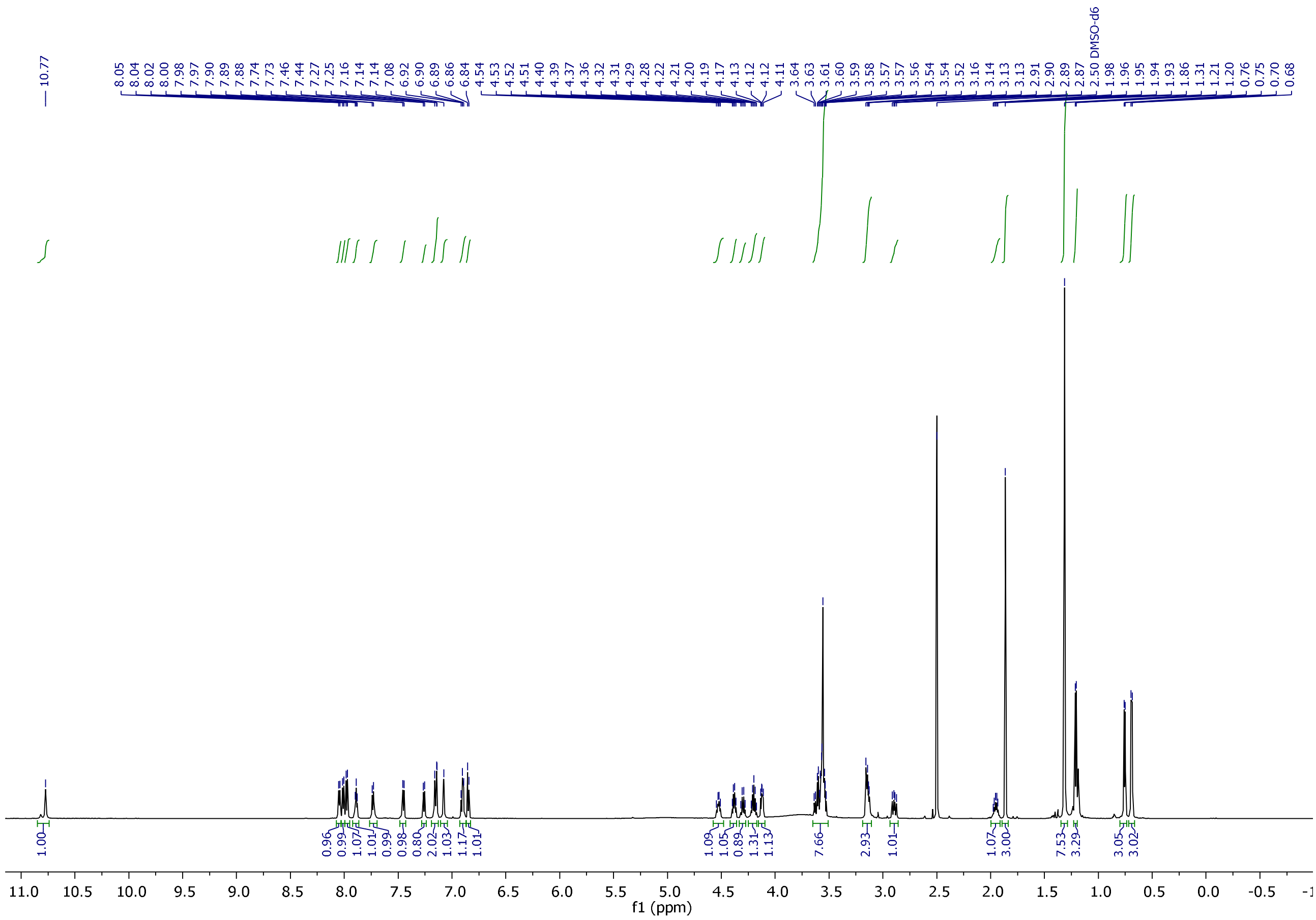

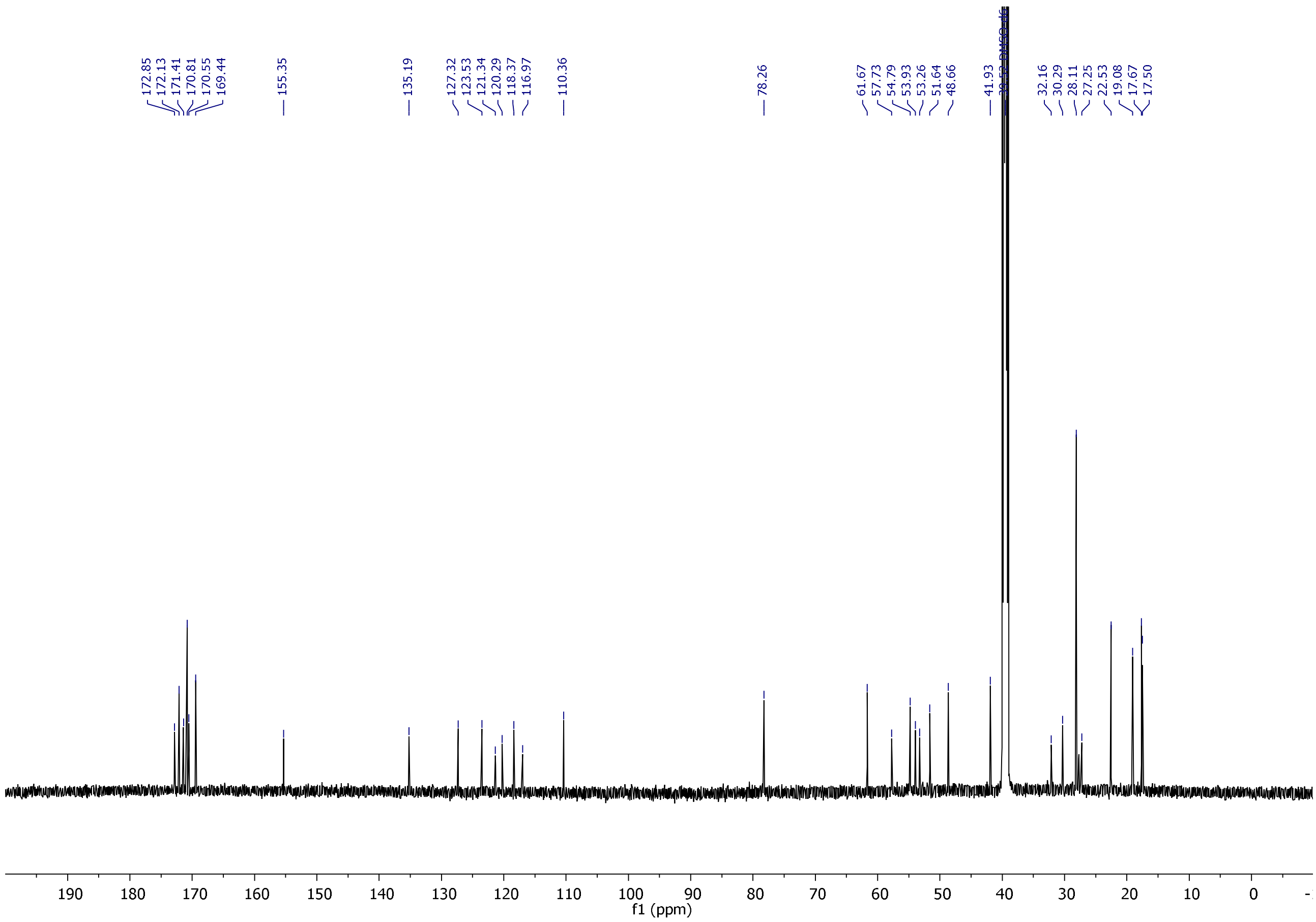

Peptide 9a

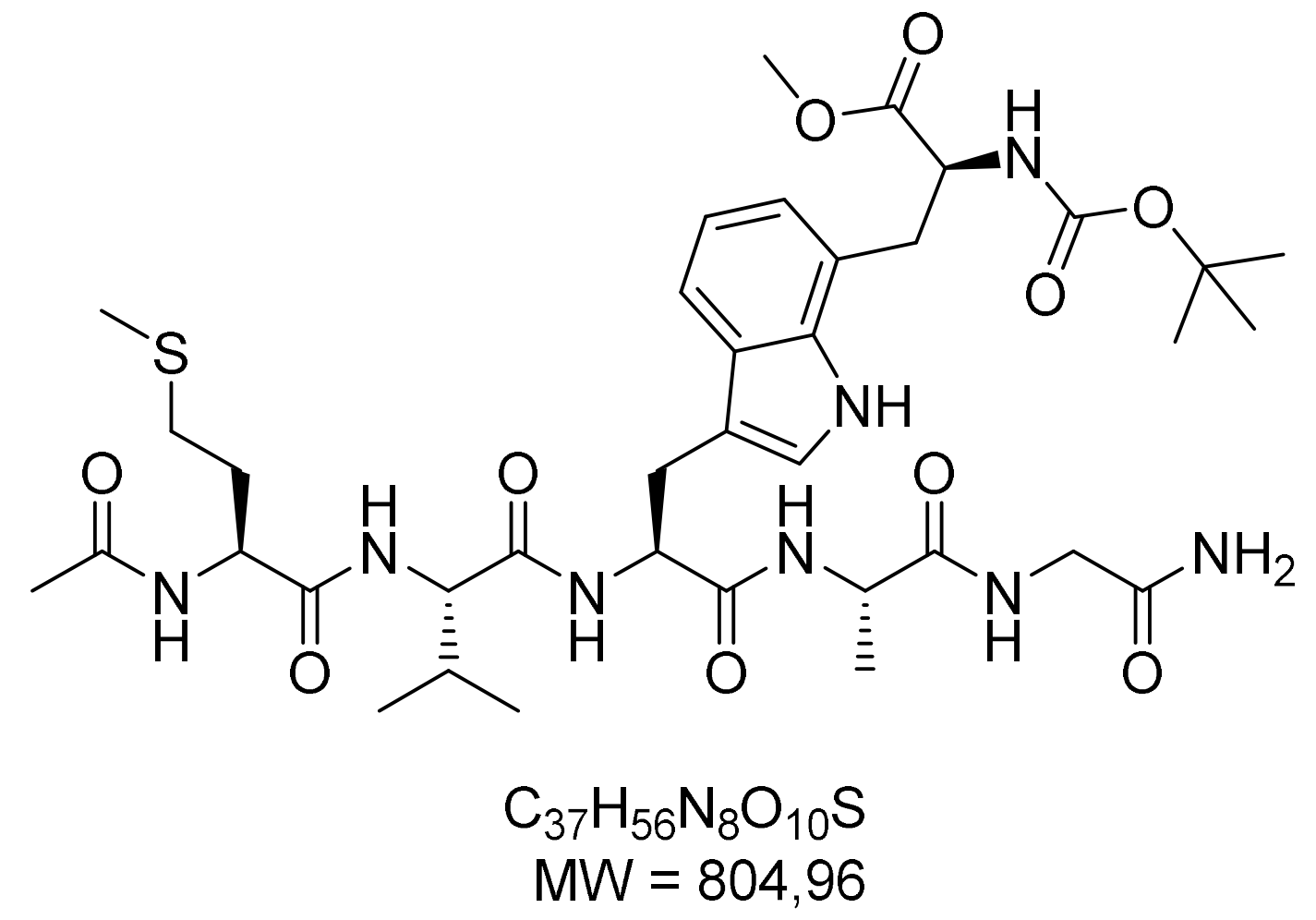

Peptide **9a** was synthesized according to GP1. Therefore, a stock solution of Ac-Met-Val-Trp(7-Br)-Ala-Gly-NH_2 (0.173 M) was prepared in anhydrous DMSO and degassed under argon atmosphere for 30 min. Separately, activated zinc dust (7.03 mg, 108 µmol, 4.2 eq.), $Pd(amphos)_2Cl_2$ (1.95 mg, 2.75 µmol, 11 mol%) and N^{α}-Boc-L-3-iodoalanine methyl ester (33.14 mg, 101 µmol, 3.9 eq.) were charged into a glass vial, which was subsequently placed under argon atmosphere. After 10 min anhydrous DMSO (150 µL), and the peptide stock solution (0.173 M, 150 µL, 1.0 eq.) were added sequentially to the vial. After every addition the reaction mixture purged with argon for 5 min. The vial was sealed and the suspension was vigorously stirred at 40 °C for 48 h. When the reaction time elapsed, the mixture was allowed to cool to room temperature, and centrifuged. The crude product was purified by preparative RP-HPLC (preparative run protocol 3). After lyophilization, a colorless solid was obtained consisting of the desired product together with the side product Boc-Ala-OMe (3.16 mg).

^{1}H NMR (600 MHz, [D_6]DMSO) δ [ppm] = 10.77 (s, 1H; N**H**-indole), 8.12 (d, $^{3}J_{H\text{-}H}$ = 6.8 Hz, 1H; N**H**^{Ala}), 8.07 (m, 2H; N**H**^{Trp}, N**H**^{Met}), 7.89 (t, $^{3}J_{H\text{-}H}$ = 5.8 Hz, 1H; N**H**^{Gly}), 7.68 (d, $^{3}J_{H\text{-}H}$ = 8.6 Hz, 1H; N**H**^{Val}), 7.45 (d, $^{3}J_{H\text{-}H}$ = 7.5 Hz, 1H; C4-**H**^{Trp}), 7.23 (d, $^{3}J_{H\text{-}H}$ = 7.8 Hz, 1H; N**H**^{Ala2}), 7.16 (s, 2H; C2-**H**^{Trp}, N**H**$_2$), 7.07 (s, 1H; N**H**$_2$), 6.90 (t, $^{3}J_{H\text{-}H}$ = 7.5 Hz, 1H; C5-**H**^{Trp}), 6.84 (d, $^{3}J_{H\text{-}H}$ = 7.5 Hz, 1H; C6-**H**^{Trp}), 4.54 (m, 1H; C_α-**H**^{Trp}), 4.38-4.27 (m, 2H; C_α-**H**^{Met}, C_α-**H**^{Ala2}), 4.20 (m, 1H; C_α-**H**^{Ala}), 4.12 (dd, $^{3}J_{H\text{-}H}$ = 8.6 Hz, $^{3}J_{H\text{-}H}$ = 6.6 Hz, 1H; C_α-**H**^{Val}), 3.65-3.52 (m, 5H; C_α-**H**$_2^{Gly}$, C**H**$_3$-OMe), 3.18-3.10 (m, 3H; C_β-**H**^{Trp}, C_β-**H**$_2^{Ala2}$), 2.93 (dd, $^{2}J_{H\text{-}H}$ = 14.8 Hz, $^{3}J_{H\text{-}H}$ = 8.3 Hz, 1H; C_β-**H**^{Trp}), 2.41 (m, 2H; C_γ-**H**$_2^{Met}$), 1.99 (s, 3H; C**H**$_3^{Met}$), 1.94 (m, 1H; C**H**^{Val}), 1.90-1.81 (m, 4H; C**H**$_3$-Ac, C_β-**H**^{Met}), 1.74 (m, 1H; C_β-**H**^{Met}), 1.31 (s, 9H; 3C**H**$_3$-Boc), 1.22 (d, $^{3}J_{H\text{-}H}$ = 7.4 Hz, 3H; C**H**$_3^{Ala}$), 0.76 (d, $^{3}J_{H\text{-}H}$ = 6.8 Hz, 3H; C**H**$_3^{Val}$), 0.75 (d, $^{3}J_{H\text{-}H}$ = 6.8 Hz, 3H; C**H**$_3^{Val}$).

^{13}C NMR (151 MHz, DMSO) δ [ppm] = 172.9 (**C**=O^{Ala2}), 172.1 (**C**=O^{Ala}), 171.3 (**C**=O^{Trp}), 171.2 (**C**=O^{Met}), 170.8 (**C**=O^{Val}, **C**=O^{Gly}), 169.4 (CH_3**C**=O), 155.4 (NH**C**=O), 135.2 (**C**7a), 127.4 (**C**3a), 123.5 (**C**2), 121.3 (**C**6), 120.3 (**C**3), 118.3 (**C**5), 117.0 (**C**4), 110.3 (**C**7), 78.3 (**C**-Boc), 57.4 (**C**$_\alpha^{Val}$), 53.9 (**C**$_\alpha^{Ala2}$), 53.2 (**C**$_\alpha^{Trp}$), 51.9 (**C**$_\alpha^{Met}$), 51.7 (**C**H_3-OMe), 48.6 (**C**$_\alpha^{Ala}$), 41.9 (**C**$_\alpha^{Gly}$), 32.2 (**C**$_\beta^{Ala2}$), 31.6 (**C**$_\beta^{Met}$),

30.7 (C_{β}^{Val}), 29.6 (C_{γ}^{Met}), 28.1 (3$C$$H_3$-Boc), 26.7 ($C_{\beta}^{Trp}$), 22.5 ($CH_3C$=O), 19.2 ($CH_3^{Val}$), 17.7 ($CH_3^{Ala}$), 16.9 ($CH_3^{Val}$), 14.6 ($CH_3^{Met}$).

HRMS (ESI): *m/z* calcd: 805.3913 $[M+H]^+$, 822.4178 $[M+NH_4]^+$, 827.3732 $[M+Na]^+$, 843.3472 $[M+K]^+$; found: 805.3917 $[M+H]^+$, 822.4169 $[M+NH_4]^+$, 827.3735 $[M+Na]^+$, 843.3497 $[M+K]^+$.

UHPLC-MS chromatogram at λ = 220 nm

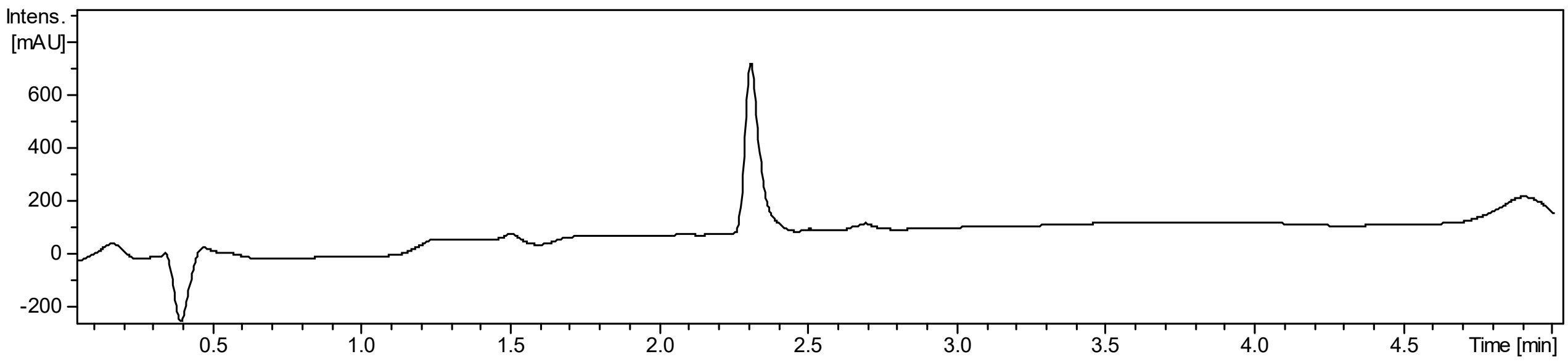

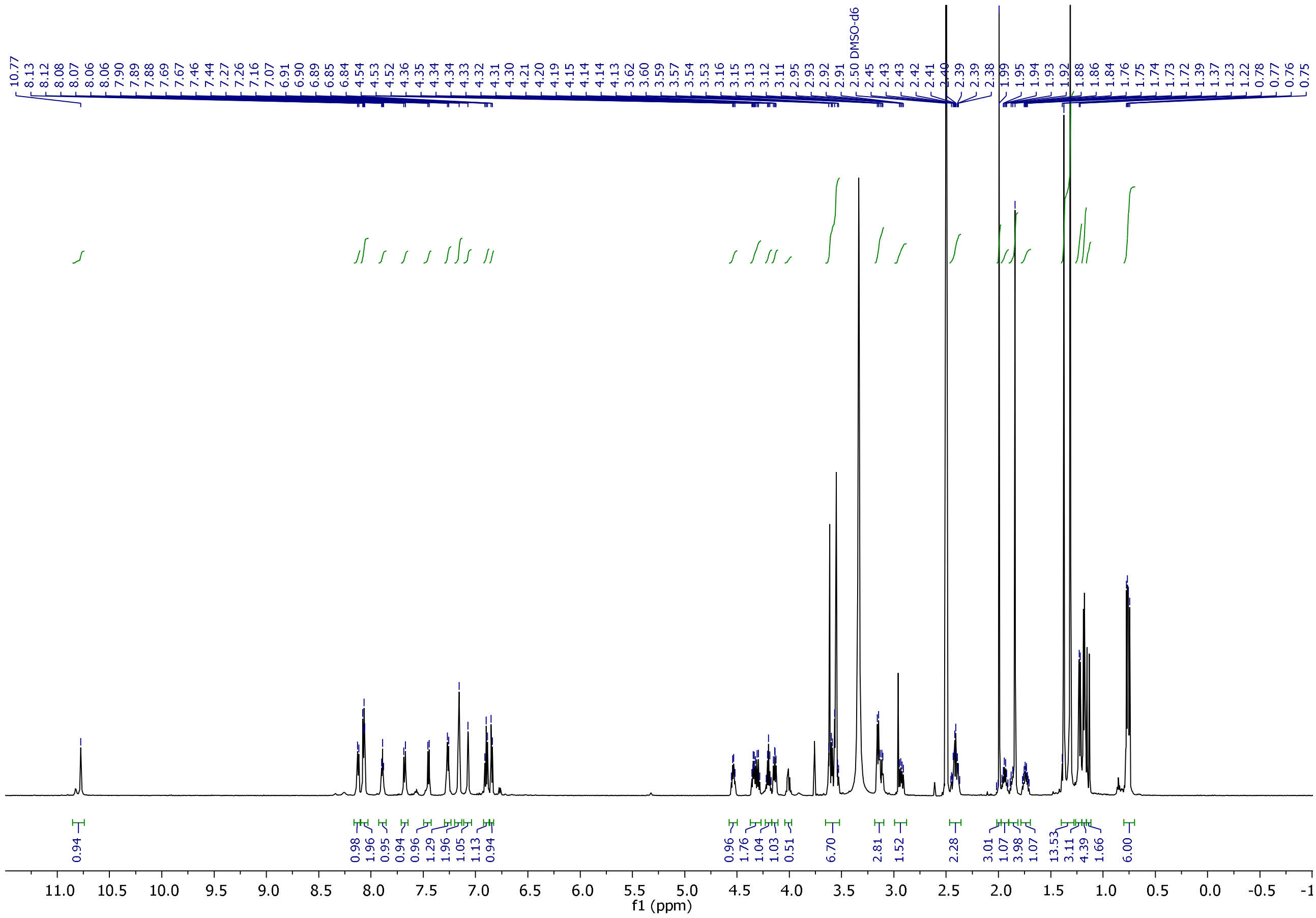

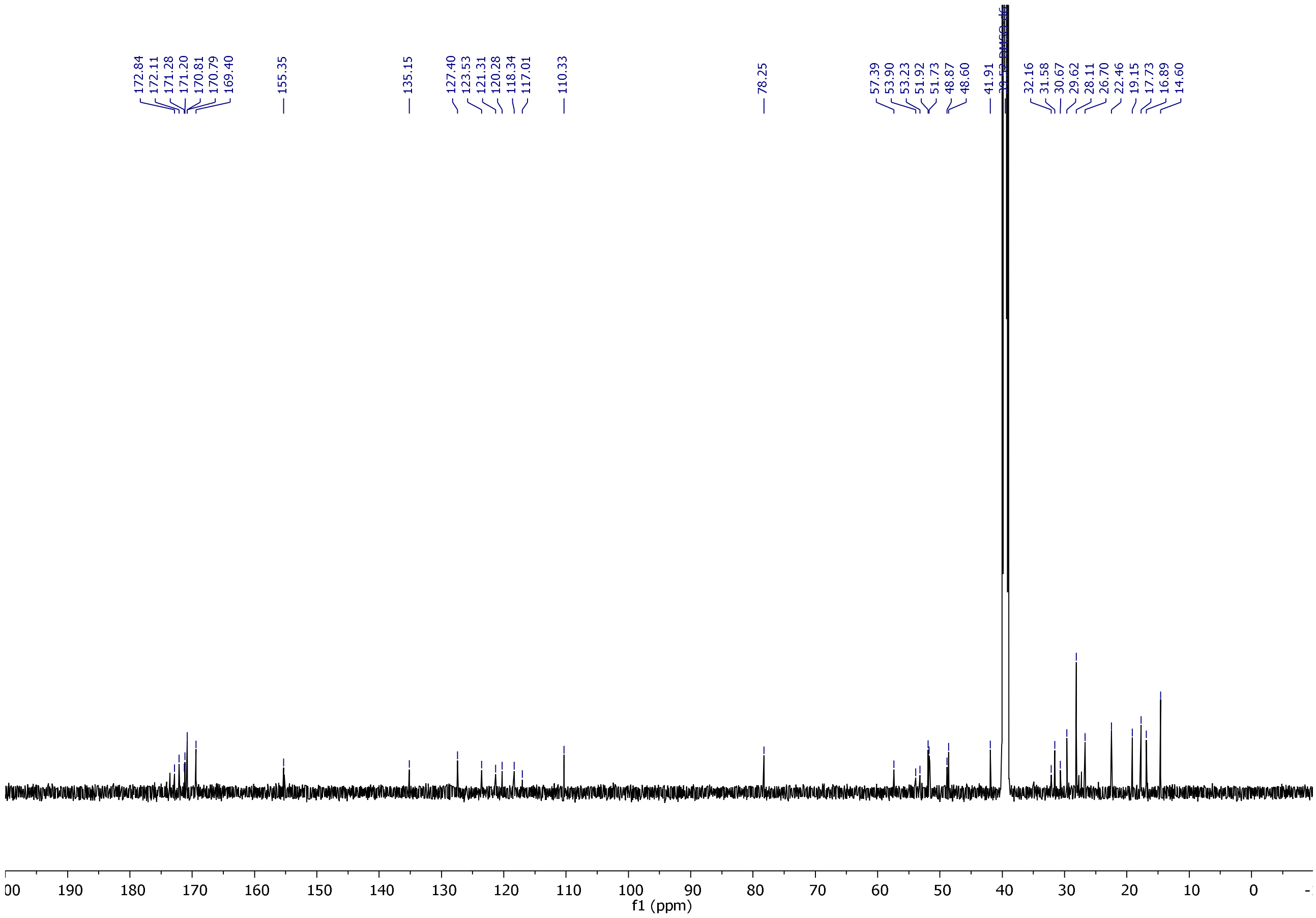

Ac-Leu-Val-Trp(6-butyl)-Ala-Gly-NH$_2$ (10a)

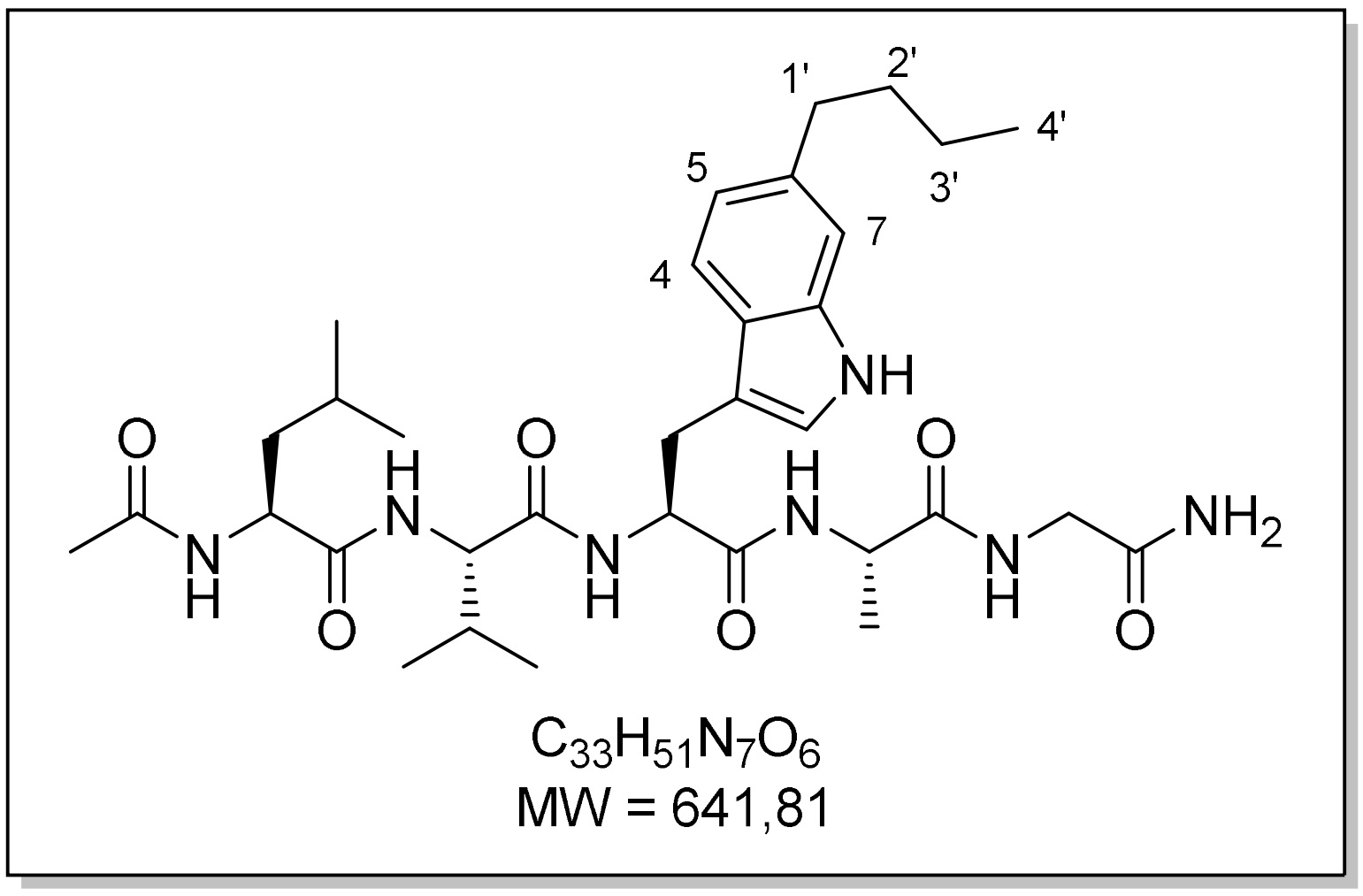

Ac-Leu-Val-Trp(6-butyl)-Ala-Gly-NH$_2$ was synthesized according to GP1. Therefore, a stock solution of Ac-Leu-Val-Trp(6-Br)-Ala-Gly-NH$_2$ (0.063 M) was prepared in a mixture of anhydrous DMF/DMSO (3:1) and degassed under argon atmosphere for 30 min. Separately, activated zinc dust (8.46 mg, 129 µmol, 5.5 eq.) and Pd(amphos)$_2$Cl$_2$ (2.12 mg, 2.99 µmol, 13 mol%) were charged into a glass vial, which was subsequently placed under argon atmosphere. After 10 min 1-iodobutane (13.7 µL, 120 µmol, 5.1 eq.), and the peptide stock solution (0.063 M, 375 µL, 1.0 eq.) were added sequentially to the vial. The reaction mixture was purged with argon for 5 min. The vial was sealed and the suspension was vigorously stirred at 40 °C for 24 h. When the reaction time elapsed, the mixture was allowed to cool to room temperature, and centrifuged. The crude product was purified by preparative RP-HPLC (preparative run protocol 3).
Lyophilization provided the product as a colourless solid (12.2 mg, 19.0 µmol, 75%).

^{1}H NMR (600 MHz, [D$_6$]DMSO): δ [ppm] = 10.60 (d, 1H, $^3J_{H\text{-}H}$ = 1.9 Hz; N**H**-indole), 8.06 (d, $^3J_{H\text{-}H}$ = 6.9 Hz, 1H; N**H**^{Ala}), 8.01 (m, 2H; N**H**^{Trp}, N**H**^{Leu}), 7.83 (t, $^3J_{H\text{-}H}$ = 5.8 Hz, 1H; N**H**^{Gly}), 7.65 (d, $^3J_{H\text{-}H}$ = 8.7 Hz, 1H; N**H**^{Val}), 7.45 (d, $^3J_{H\text{-}H}$ = 8.1 Hz, 1H; C4-**H**^{Trp}), 7.15 (s, 1H; N**H**$_2$), 7.08 (s, 1H; C7-**H**^{Trp}), 7.07 (s, 1H; N**H**$_2$), 7.04 (d, $^3J_{H\text{-}H}$ = 1.9 Hz, 1H; C2-**H**^{Trp}), 6.81 (dd, $^3J_{H\text{-}H}$ = 8.1 Hz, $^3J_{H\text{-}H}$ = 1.1 Hz, 1H; C5-**H**^{Trp}), 4.52 (m, 1H; C$_\alpha$-**H**^{Trp}), 4.29 (m, 1H; C$_\alpha$-**H**^{Leu}), 4.19 (m, 1H; C$_\alpha$-**H**^{Ala}), 4.12 (dd, $^3J_{H\text{-}H}$ = 8.7 Hz, $^3J_{H\text{-}H}$ = 6.9 Hz, 1H; C$_\alpha$-**H**^{Val}), 3.60 (dd, $^2J_{H\text{-}H}$ = 16.8 Hz, $^3J_{H\text{-}H}$ = 5.8 Hz, 1H; C$_\alpha$-**H**^{Gly}), 3.54 (dd, $^2J_{H\text{-}H}$ = 16.8 Hz, $^3J_{H\text{-}H}$ = 5.8 Hz, 1H; C$_\alpha$-**H**^{Gly}), 3.08 (dd, $^2J_{H\text{-}H}$ = 14.8 Hz, $^3J_{H\text{-}H}$ = 5.5 Hz, 1H; C$_\beta$-**H**^{Trp}), 2.91 (dd, $^2J_{H\text{-}H}$ = 14.8 Hz, $^3J_{H\text{-}H}$ = 8.3 Hz, 1H; C$_\beta$-**H**^{Trp}), 2.63 (t, $^3J_{H\text{-}H}$ = 7.6 Hz, 2H; C1'-**H**$_2$), 1.92 (m, 1H; C**H**^{Val}), 1.83 (s, 3H; C**H**$_3$-Ac), 1.57 (m, 3H; C**H**^{Leu}, C2'-**H**$_2$), 1.40 (m, 2H; C-**H**$_2$^{Leu}), 1.31 (m, 2H; C3'-**H**$_2$), 1.16 (d, $^3J_{H\text{-}H}$ = 7.1 Hz, 3H; C**H**$_3$^{Ala}), 0.89 (t, $^3J_{H\text{-}H}$ = 7.4 Hz, 3H; C4'-**H**$_3$), 0.86 (d, $^3J_{H\text{-}H}$ = 6.6 Hz, 3H; C**H**$_3$^{Leu}), 0.81 (d, $^3J_{H\text{-}H}$ = 6.6 Hz, 3H; C**H**$_3$^{Leu}), 0.76 (d, $^3J_{H\text{-}H}$ = 6.7 Hz, 3H; C**H**$_3$^{Val}), 0.75 (d, $^3J_{H\text{-}H}$ = 6.8 Hz, 3H; C**H**$_3$^{Val}).

^{13}C NMR (151 MHz, DMSO): δ [ppm] = 172.1 (**C**=O^{Ala}), 172.0 (**C**=O^{Leu}), 171.3 (**C**=O^{Trp}), 170.8 (**C**=O^{Val}, **C**=O^{Gly}), 169.2 (CH$_3$**C**=O), 136.4 (**C**7a), 135.0 (**C**6), 125.5 (**C**3a), 122.9 (**C**2), 119.3 (**C**5), 118.1 (**C**4), 110.4 (**C**7), 109.6 (**C**3), 57.4 (**C**$_\alpha$^{Val}), 53.2 (**C**$_\alpha$^{Trp}), 51.1 (**C**$_\alpha$^{Leu}), 48.5 (**C**$_\alpha$^{Ala}), 41.9 (**C**$_\alpha$^{Gly}), 40.4 (**C**$_\beta$^{Leu}), 35.2 (**C**1'), 33.9 (**C**2'), 30.7 (**C**$_\beta$^{Val}), 27.4 (**C**$_\beta$^{Trp}), 24.2 (**C**$_\gamma$^{Leu}), 23.0 (**C**H$_3$^{Leu}), 22.5 (**C**H$_3$C=O), 21.8 (**C**3'), 21.6 (**C**H$_3$^{Leu}), 19.1 (**C**H$_3$^{Val}), 18.0 (**C**H$_3$^{Val}), 17.8 (**C**H$_3$^{Ala}), 13.9 (**C**4').

HRMS (ESI): *m/z* calcd: 642.3974 [M+H]$^+$, 659.4239 [M+Na]$^+$, 664.3793 [M+K]$^+$; found: 642.3969 [M+H]$^+$, 659.4233 [M+Na]$^+$, 664.3786 [M+K]$^+$.

UHPLC-MS chromatogram at λ = 220 nm

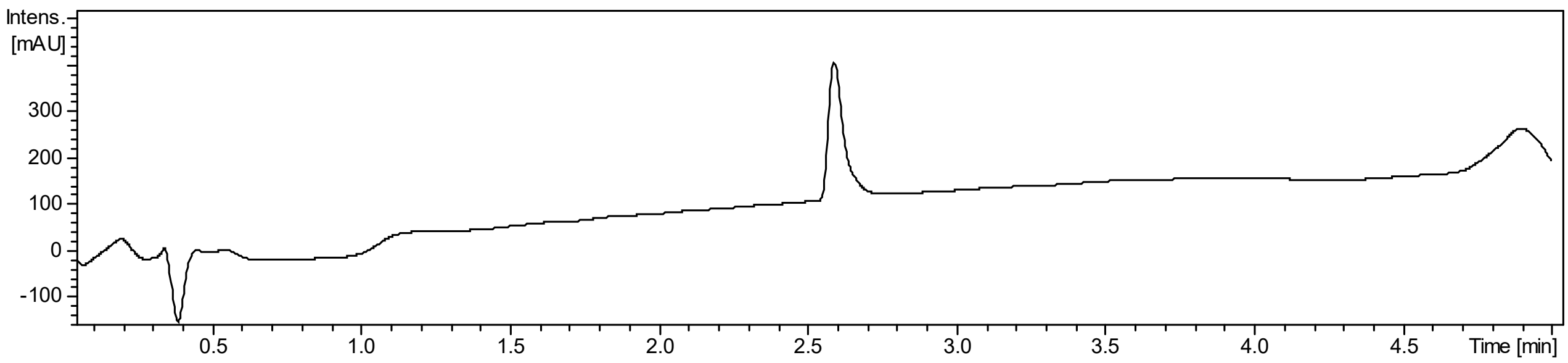

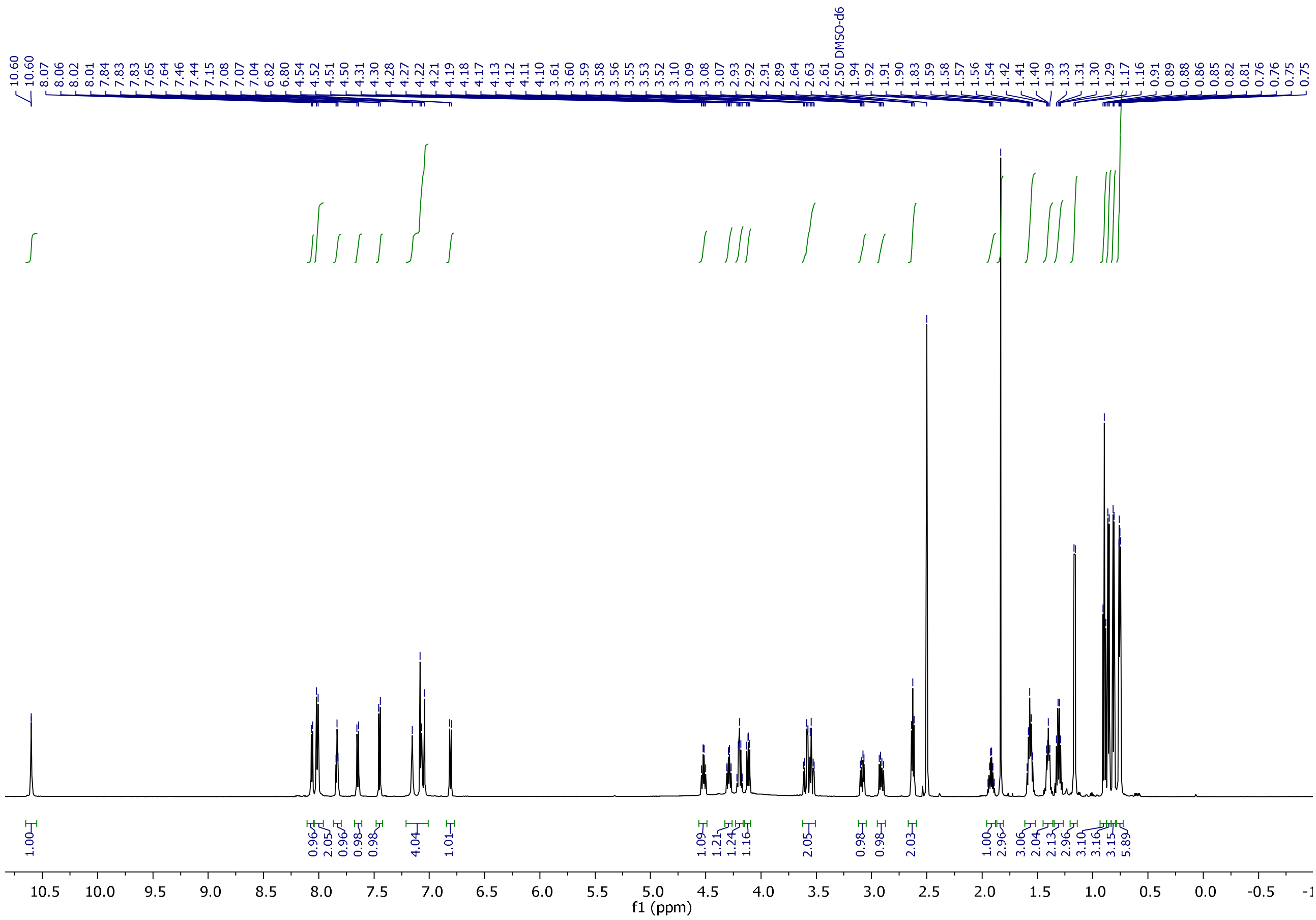
10.60
10.60
8.07
8.06
8.02
8.01
7.84
7.83
7.65
7.64
7.46
7.44
7.15
7.08
7.07
7.04
6.82
6.80
4.54
4.52
4.51
4.50
4.31
4.30
4.28
4.27
4.22
4.21
4.19
4.18
4.17
4.13
4.12
4.11
4.10
3.61
3.60
3.59
3.58
3.56
3.55
3.53
3.52
3.10
3.09
3.08
3.07
2.93
2.92
2.91
2.89
2.64
2.63
2.61
2.50 DMSO-d6
1.94
1.92
1.91
1.90
1.83
1.59
1.58
1.57
1.56
1.54
1.42
1.41
1.40
1.39
1.33
1.31
1.30
1.29
1.17
1.16
0.91
0.89
0.88
0.86
0.85
0.82
0.81
0.76
0.75
0.75
1.00
0.96
2.05
0.96
0.98
0.98
4.04
1.01
1.09
1.21
1.24
1.16
2.05
0.98
0.98
2.03
1.00
2.96
3.06
2.04
2.13
2.96
3.10
3.16
3.15
5.89
f1 (ppm)

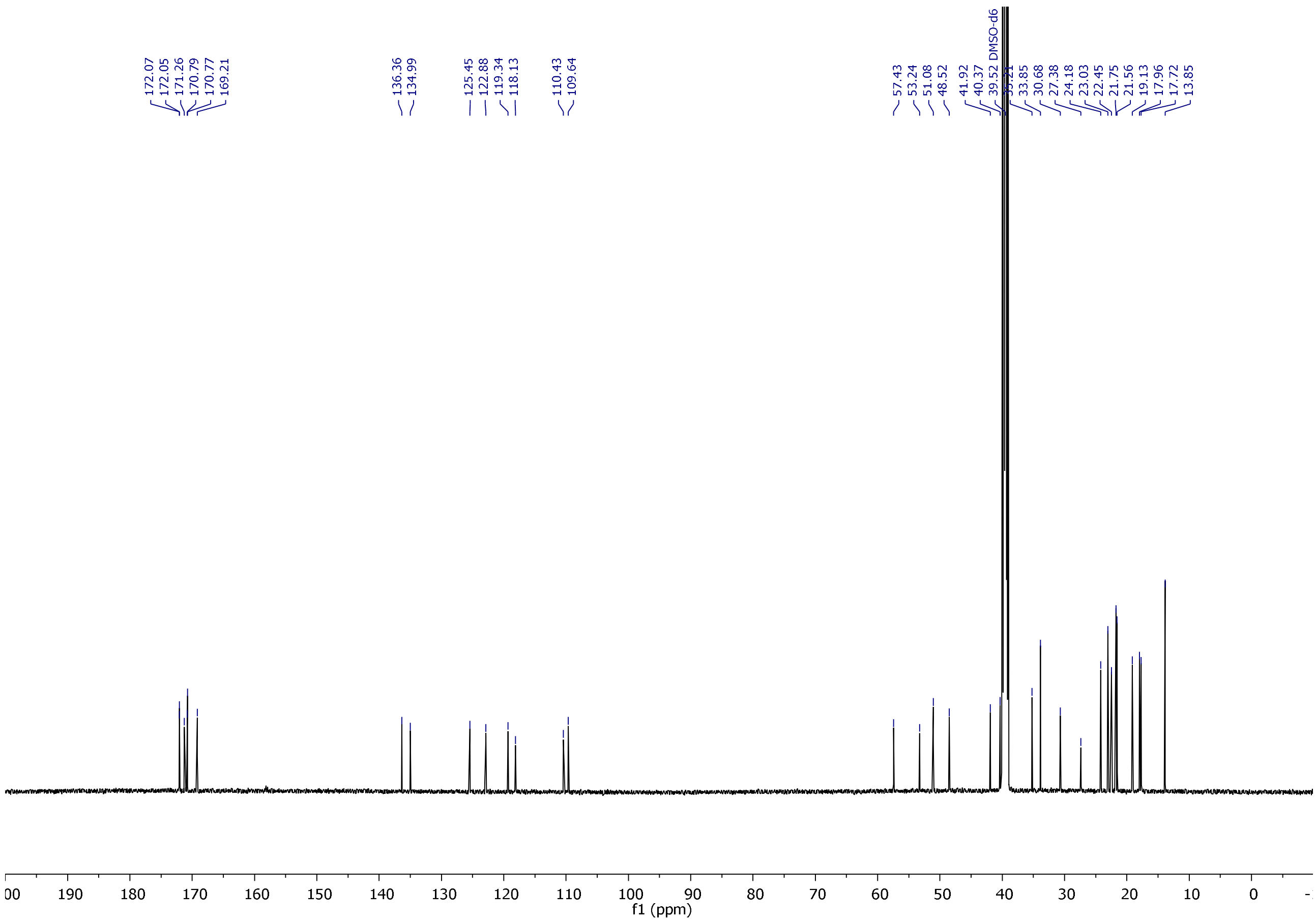
172.07
172.05
171.26
170.79
170.77
169.21
136.36
134.99
125.45
122.88
119.34
118.13
110.43
109.64
57.43
53.24
51.08
48.52
41.92
40.37
39.52 DMSO-d6
35.21
33.85
30.68
27.38
24.18
23.03
22.45
21.75
21.56
19.13
17.96
17.72
13.85
f1 (ppm)

Ac-Leu-Val-Trp(6-cyclohexyl)-Ala-Gly-NH$_2$ (10b)

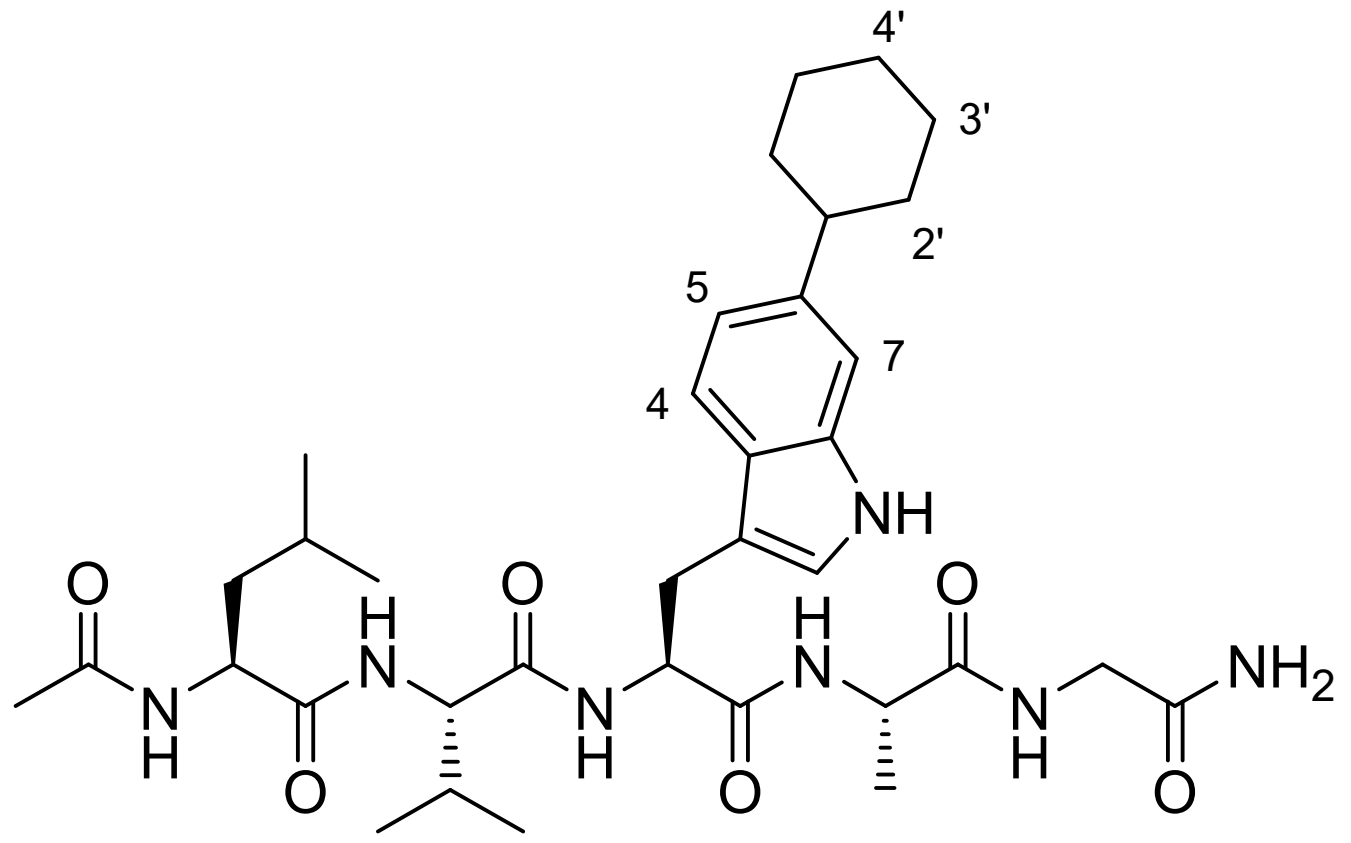

$C_{35}H_{53}N_7O_6$
MW = 667,85

Ac-Leu-Val-Trp(6-cyclohexyl)-Ala-Gly-NH$_2$ was synthesized according to GP1. Therefore, a stock solution of Ac-Leu-Val-Trp(6-Br)-Ala-Gly-NH$_2$ (0.101 M) was prepared in a mixture of anhydrous DMF/DMSO (3:2) and degassed under argon atmosphere for 30 min. Separately, activated zinc dust (8.29 mg, 127 µmol, 4.2 eq.) and Pd(amphos)$_2$Cl$_2$ (2.15 mg, 3.04 µmol, 10 mol%) were charged into a glass vial, which was subsequently placed under argon atmosphere. After 10 min iodocyclohexane (15.5 µL, 120 µmol, 4.0 eq.), and the peptide stock solution (0.101 M, 300 µL, 1.0 eq.) were added sequentially to the vial. The reaction mixture was purged with argon for 5 min. The vial was sealed and the suspension was vigorously stirred at 40 °C for 24 h. When the reaction time elapsed, the mixture was allowed to cool to room temperature, and centrifuged. The crude product was purified by preparative RP-HPLC (preparative run protocol 3). Lyophilization provided the product as a colourless solid (13.8 mg, 20.7 µmol, 68%).

^{1}H NMR (600 MHz, [D$_6$]DMSO): δ [ppm] = 10.60 (d, 1H, $^3J_{H\text{-}H}$ = 2.0 Hz; N**H**-indole), 8.06 (d, $^3J_{H\text{-}H}$ = 6.9 Hz, 1H; N**H**^{Ala}), 8.01 (m, 2H; N**H**^{Trp}, N**H**^{Leu}), 7.82 (t, $^3J_{H\text{-}H}$ = 5.9 Hz, 1H; N**H**^{Gly}), 7.64 (d, $^3J_{H\text{-}H}$ = 8.8 Hz, 1H; N**H**^{Val}), 7.45 (d, $^3J_{H\text{-}H}$ = 8.2 Hz, 1H; C4-**H**^{Trp}), 7.15 (s, 1H; N**H**$_2$), 7.10 (s, 1H; C7-**H**^{Trp}), 7.07 (s, 1H; N**H**$_2$), 7.05 (d, $^3J_{H\text{-}H}$ = 2.0 Hz, 1H; C2-**H**^{Trp}), 6.85 (dd, $^3J_{H\text{-}H}$ = 8.2 Hz, $^3J_{H\text{-}H}$ = 1.2 Hz, 1H; C5-**H**^{Trp}), 4.51 (m, 1H; C$_\alpha$-**H**^{Trp}), 4.29 (ddd, $^3J_{H\text{-}H}$ = 8.6 Hz, $^3J_{H\text{-}H}$ = 8.5 Hz, $^3J_{H\text{-}H}$ = 6.3 Hz, 1H; C$_\alpha$-**H**^{Leu}), 4.19 (m, 1H; C$_\alpha$-**H**^{Ala}), 4.12 (dd, $^3J_{H\text{-}H}$ = 8.8 Hz, $^3J_{H\text{-}H}$ = 6.8 Hz, 1H; C$_\alpha$-**H**^{Val}), 3.59 (dd, $^2J_{H\text{-}H}$ = 16.8 Hz, $^3J_{H\text{-}H}$ = 5.9 Hz, 1H; C$_\alpha$-**H**^{Gly}), 3.53 (dd, $^2J_{H\text{-}H}$ = 16.8 Hz, $^3J_{H\text{-}H}$ = 5.9 Hz, 1H; C$_\alpha$-**H**^{Gly}), 3.08 (dd, $^2J_{H\text{-}H}$ = 14.7 Hz, $^3J_{H\text{-}H}$ = 5.6 Hz, 1H; C$_\beta$-**H**^{Trp}), 2.91 (dd, $^2J_{H\text{-}H}$ = 14.7 Hz, $^3J_{H\text{-}H}$ = 8.1 Hz, 1H; C$_\beta$-**H**^{Trp}), 2.53 (m, 1H; C1'-**H**), 1.92 (m, 1H; C**H**^{Val}), 1.83 (s, 3H; C**H**$_3$-Ac), 1.81 (m, 4H; 2C**H**$_2$-cyclohexy), 1.71 (m, 1H; C4'-**H**), 1.56 (m, 1H; C**H**^{Leu}), 1.47-1.33 (m, 6H, C-**H**$_2^{Leu}$; 2C**H**$_2$-cyclohexy), 1.25 (m, 1H; C4'-**H**), 1.16 (d, $^3J_{H\text{-}H}$ = 7.1 Hz, 3H; C**H**$_3^{Ala}$), 0.86 (d, $^3J_{H\text{-}H}$ = 6.6 Hz, 3H; C**H**$_3^{Leu}$), 0.81 (d, $^3J_{H\text{-}H}$ = 6.6 Hz, 3H; C**H**$_3^{Leu}$), 0.76 (d, $^3J_{H\text{-}H}$ = 6.6 Hz, 3H; C**H**$_3^{Val}$), 0.75 (d, $^3J_{H\text{-}H}$ = 6.6 Hz, 6H; C**H**$_3^{Val}$).

^{13}C NMR (151 MHz, DMSO): δ [ppm] = 172.1 (**C**=O^{Ala}), 172.0 (**C**=O^{Leu}), 171.2 (**C**=O^{Trp}), 170.8 (**C**=O^{Val}, **C**=O^{Gly}), 169.2 (CH$_3$**C**=O), 140.6 (**C**6), 136.3 (**C**7a), 125.6 (**C**3a), 123.0 (**C**2), 118.2 (**C**4), 118.0 (**C**5), 109.6 (**C**3), 108.6 (**C**7), 57.4 (**C**$_\alpha^{Val}$), 53.3 (**C**$_\alpha^{Trp}$), 51.1 (**C**$_\alpha^{Leu}$), 48.5 (**C**$_\alpha^{Ala}$), 44.1 (**C**1'), 41.9

(C_α^{Gly}), 40.4 (C_β^{Leu}), 34.7 (2CH$_2$-cyclohexy), 30.7 (C_β^{Val}), 27.4 (C_β^{Trp}), 26.6 (2CH$_2$-cyclohexy), 25.8 (C4'), 24.2 (C_γ^{Leu}), 23.0 (CH_3^{Leu}), 22.5 (CH$_3$C=O), 21.6 (CH_3^{Leu}), 19.1 (CH_3^{Val}), 18.0 (CH_3^{Val}), 17.7 (CH_3^{Ala}).

HRMS (ESI): *m/z* calcd: 668.4130 $[M+H]^+$, 690.3950 $[M+Na]^+$, 706.3689 $[M+K]^+$; found: 668.4119 $[M+H]^+$, 690.3938 $[M+Na]^+$, 706.3683 $[M+K]^+$.

UHPLC-MS chromatogram at λ = 220 nm

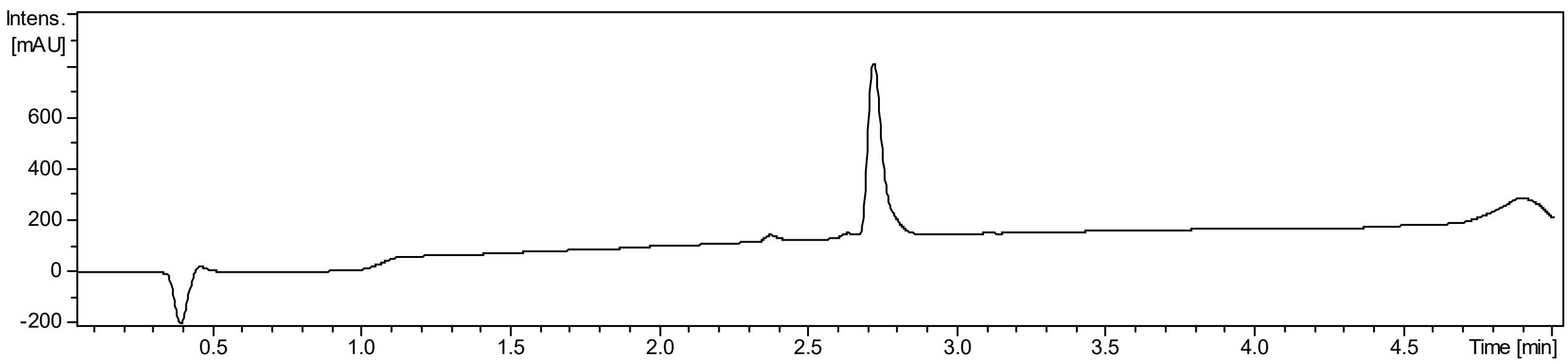

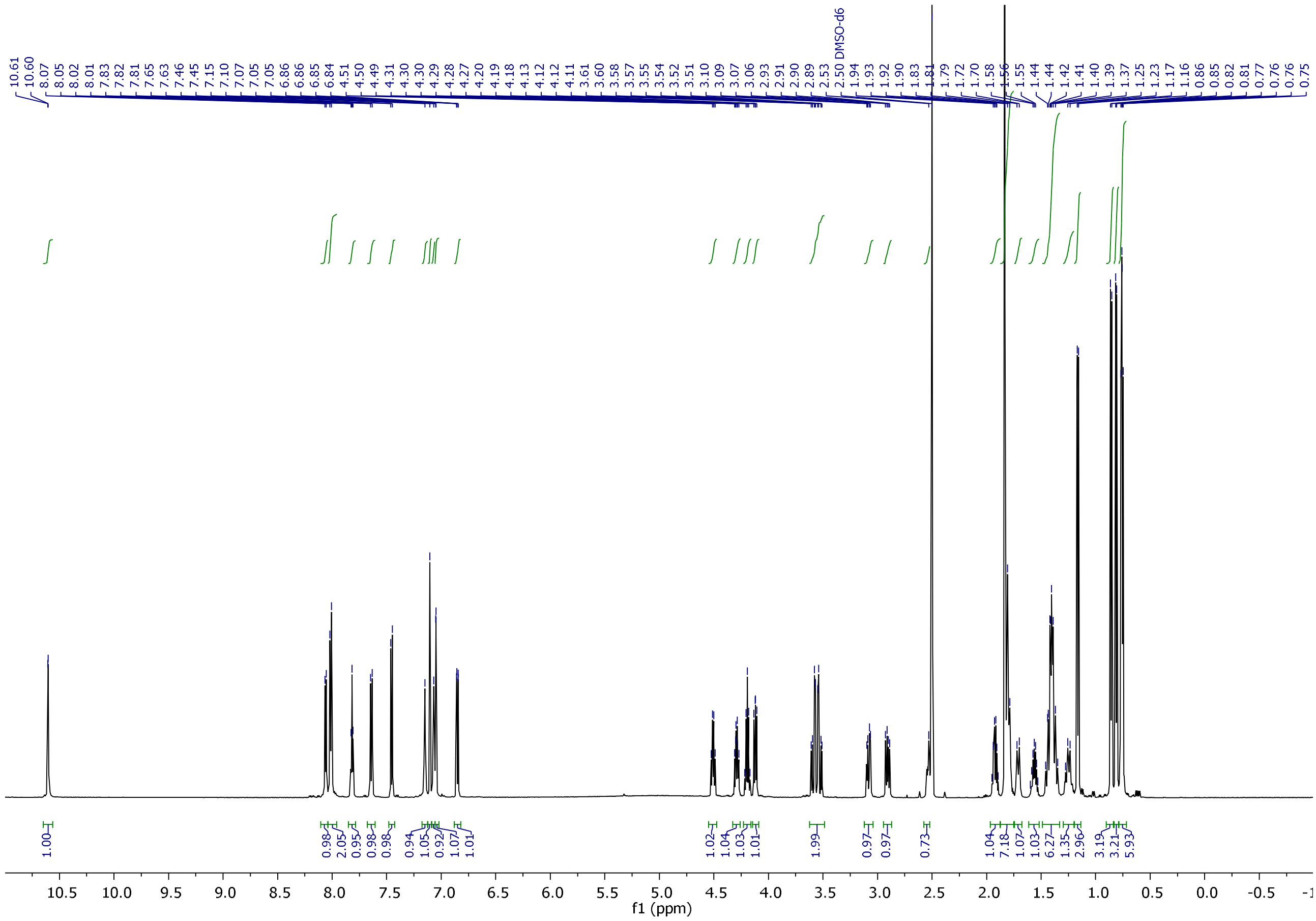
2.50 DMSO-d6
f1 (ppm)

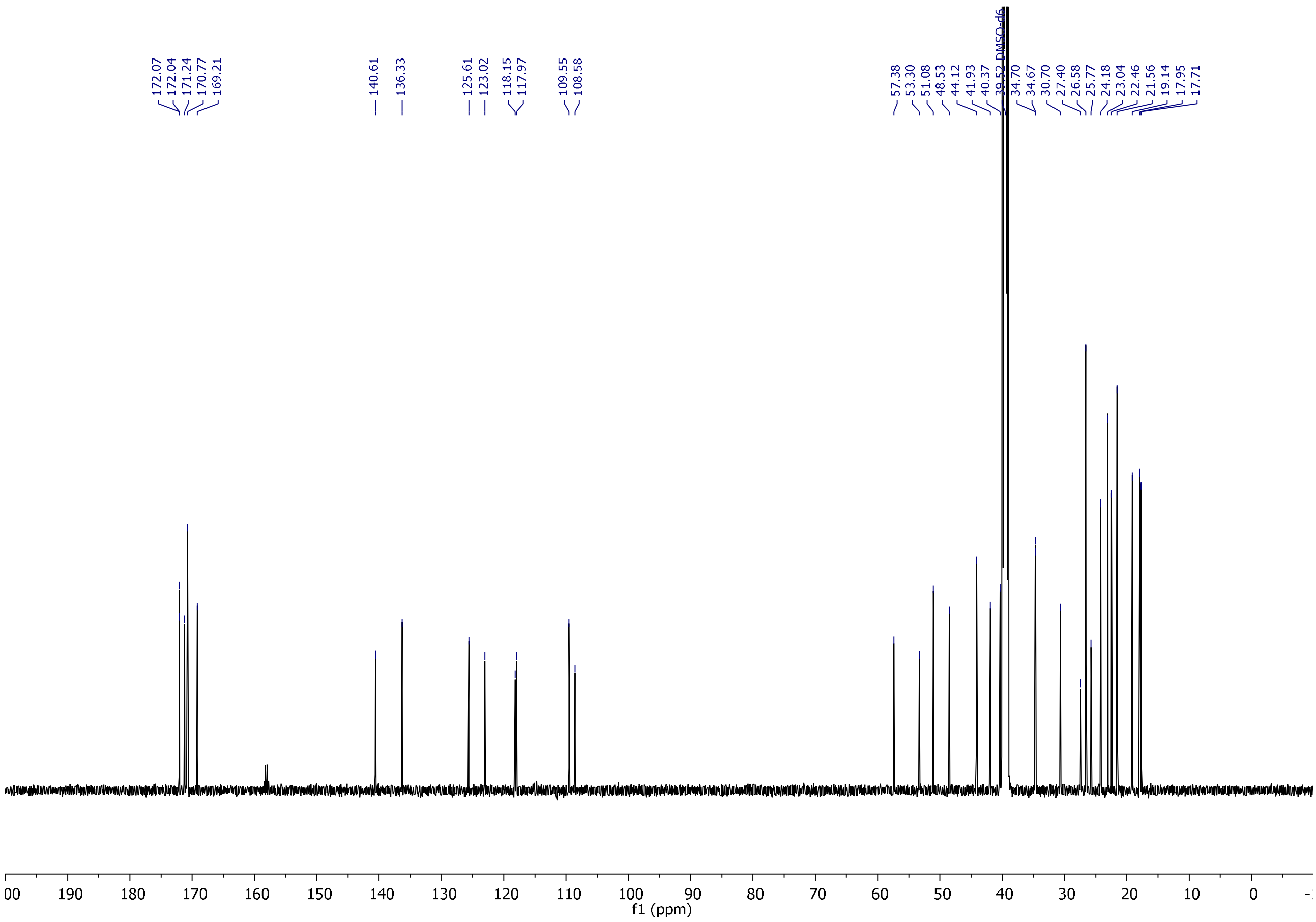
39.52 DMSO-d6
f1 (ppm)

Ac-Leu-Val-Trp(6-benzyl)-Ala-Gly-NH_2 (10c)

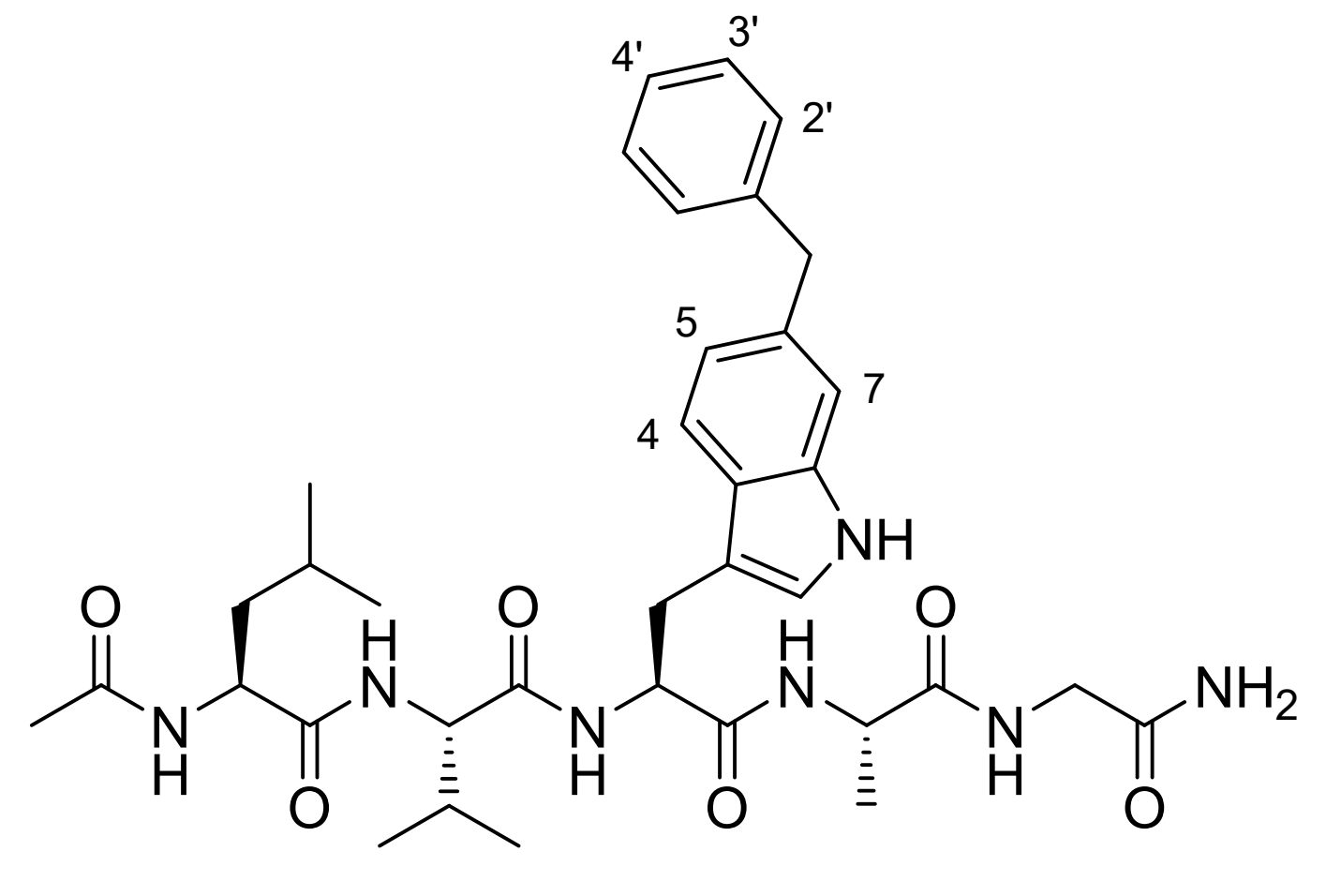

Ac-Leu-Val-Trp(6-benzyl)-Ala-Gly-NH_2 was synthesized according to GP1. Therefore, a stock solution of Ac-Leu-Val-Trp(6-Br)-Ala-Gly-NH_2 (0.101 M) was prepared in a mixture of anhydrous DMF/DMSO (3:2) and degassed under argon atmosphere for 30 min. Separately, activated zinc dust (8.35 mg, 128 µmol, 4.2 eq.), $Pd(amphos)_2Cl_2$ (2.32 mg, 3.28 µmol, 11 mol%) and benzyl iodide (39.01 mg, 179 µmol, 5.9 eq.) were charged into a glass vial, which was subsequently placed under argon atmosphere. After 10 min the peptide stock solution (0.101 M, 300 µL, 1 eq.) was added to the vial. The reaction mixture was purged with argon for 5 min. The vial was sealed and the suspension was vigorously stirred at 40 °C for 24 h. When the reaction time elapsed, the mixture was allowed to cool to room temperature, and centrifuged. The crude product was purified by preparative RP-HPLC (preparative run protocol 3). Lyophilization provided the product as a colourless solid (17.0 mg, 25.2 µmol, 83%).

^{1}H NMR (600 MHz, [D$_6$]DMSO): δ [ppm] = 10.65 (d, 1H, $^3J_{H-H}$ = 2.0 Hz; N**H**-indole), 8.07 (d, $^3J_{H-H}$ = 6.9 Hz, 1H; N$\mathbf{H}^{Ala}$), 8.02 (d, $^3J_{H-H}$ = 7.6 Hz, 1H; N$\mathbf{H}^{Trp}$), 8.01 (d, $^3J_{H-H}$ = 8.2 Hz, 1H; N$\mathbf{H}^{Leu}$), 7.85 (t, $^3J_{H-H}$ = 5.8 Hz, 1H; N$\mathbf{H}^{Gly}$), 7.66 (d, $^3J_{H-H}$ = 8.7 Hz, 1H; N$\mathbf{H}^{Val}$), 7.47 (d, $^3J_{H-H}$ = 8.1 Hz, 1H; C4-$\mathbf{H}^{Trp}$), 7.26 (m, 2H; C2'-**H**/C6'-**H**), 7.22 (m, 2H; C3'-**H**/C5'-**H**), 7.18-7.13 (m, 2H; C4'-**H**, N$\mathbf{H}_2$), 7.10 (s, 1H; C7-$\mathbf{H}^{Trp}$), 7.08 (s, 1H; N$\mathbf{H}_2$), 7.06 (d, $^3J_{H-H}$ = 2.0 Hz, 1H; C2-$\mathbf{H}^{Trp}$), 6.86 (dd, $^3J_{H-H}$ = 8.1 Hz, $^3J_{H-H}$ = 1.1 Hz, 1H; C5-$\mathbf{H}^{Trp}$), 4.51 (m, 1H; C$_\alpha$-$\mathbf{H}^{Trp}$), 4.29 (ddd, $^3J_{H-H}$ = 8.6 Hz, $^3J_{H-H}$ = 8.2 Hz, $^3J_{H-H}$ = 6.4 Hz, 1H; C$_\alpha$-$\mathbf{H}^{Leu}$), 4.19 (m, 1H; C$_\alpha$-$\mathbf{H}^{Ala}$), 4.11 (dd, $^3J_{H-H}$ = 8.7 Hz, $^3J_{H-H}$ = 6.9 Hz, 1H; C$_\alpha$-$\mathbf{H}^{Val}$), 3.99 (s, 2H; C$\mathbf{H}_2$), 3.59 (dd, $^2J_{H-H}$ = 16.8 Hz, $^3J_{H-H}$ = 5.8 Hz, 1H; C$_\alpha$-$\mathbf{H}^{Gly}$), 3.54 (dd, $^2J_{H-H}$ = 16.8 Hz, $^3J_{H-H}$ = 5.8 Hz, 1H; C$_\alpha$-$\mathbf{H}^{Gly}$), 3.07 (dd, $^2J_{H-H}$ = 14.8 Hz, $^3J_{H-H}$ = 5.5 Hz, 1H, C$_\beta$-$\mathbf{H}^{Trp}$), 2.90 (dd, $^2J_{H-H}$ = 14.8 Hz, $^3J_{H-H}$ = 8.3 Hz, 1H; C$_\beta$-$\mathbf{H}^{Trp}$), 1.91 (m, 1H; C$\mathbf{H}^{Val}$), 1.83 (s, 3H; C$\mathbf{H}_3$-Ac), 1.56 (m, 1H; C$\mathbf{H}^{Leu}$), 1.39 (m, 2H; C-$\mathbf{H}_2^{Leu}$), 1.15 (d, $^3J_{H-H}$ = 7.1 Hz, 3H; C$\mathbf{H}_3^{Ala}$), 0.85 (d, $^3J_{H-H}$ = 6.6 Hz, 3H; C$\mathbf{H}_3^{Leu}$), 0.80 (d, $^3J_{H-H}$ = 6.6 Hz, 3H; C$\mathbf{H}_3^{Leu}$), 0.75 (d, $^3J_{H-H}$ = 6.7 Hz, 6H; C$\mathbf{H}_3^{Val}$), 0.75 (d, $^3J_{H-H}$ = 6.7 Hz, 6H; C$\mathbf{H}_3^{Val}$).

^{13}C NMR (151 MHz, DMSO): δ [ppm] = 172.1 (**C**=O^{Ala}, **C**=O^{Leu}), 171.3 (**C**=O^{Trp}), 170.8 (**C**=O^{Val}, **C**=O^{Gly}), 169.2 (CH$_3$**C**=O), 142.20 (**C**1'), 136.4 (**C**7a), 134.0 (**C**6), 128.7 (**C**2'/**C**6'), 128.3 (**C**3'/**C**5'), 125.7 (**C**3a), 125.6 (**C**4'), 123.2 (**C**2), 119.8 (**C**5), 118.4 (**C**4), 111.1 (**C**7), 109.7 (**C**3), 57.4 ($\mathbf{C}_\alpha^{Val}$),

53.2 ($\mathbf{C}_{\alpha}^{\mathrm{Trp}}$), 51.1 ($\mathbf{C}_{\alpha}^{\mathrm{Leu}}$), 48.5 ($\mathbf{C}_{\alpha}^{\mathrm{Ala}}$), 41.9 ($\mathbf{C}_{\alpha}^{\mathrm{Gly}}$), 41.5 ($\mathbf{C}H_2$), 40.4 ($\mathbf{C}_{\beta}^{\mathrm{Leu}}$), 30.7 ($\mathbf{C}_{\beta}^{\mathrm{Val}}$), 27.4 ($\mathbf{C}_{\beta}^{\mathrm{Trp}}$), 24.2 ($\mathbf{C}_{\gamma}^{\mathrm{Leu}}$), 23.1 ($\mathbf{C}H_3^{\mathrm{Leu}}$), 22.5 ($\mathbf{C}H_3C{=}O$), 21.6 ($\mathbf{C}H_3^{\mathrm{Leu}}$), 19.1 ($\mathbf{C}H_3^{\mathrm{Val}}$), 18.0 ($\mathbf{C}H_3^{\mathrm{Val}}$), 17.7 ($\mathbf{C}H_3^{\mathrm{Ala}}$).

<u>**HRMS**</u> (ESI): *m/z* calcd: 676.3816 $[M+H]^+$, 693.4083 $[M+NH_4]^+$, 698.3637 $[M+Na]^+$, 714.3376 $[M+K]^+$; found: 676.3816 $[M+H]^+$, 693.4093 $[M+NH_4]^+$, 698.3637 $[M+Na]^+$, 714.3372 $[M+K]^+$.

UHPLC-MS chromatogram at λ = 220 nm

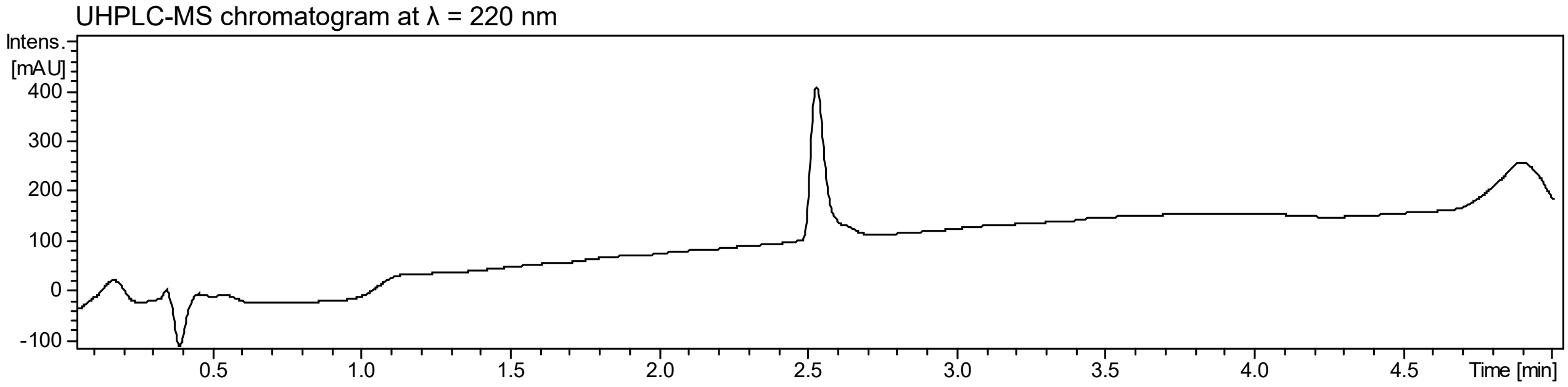

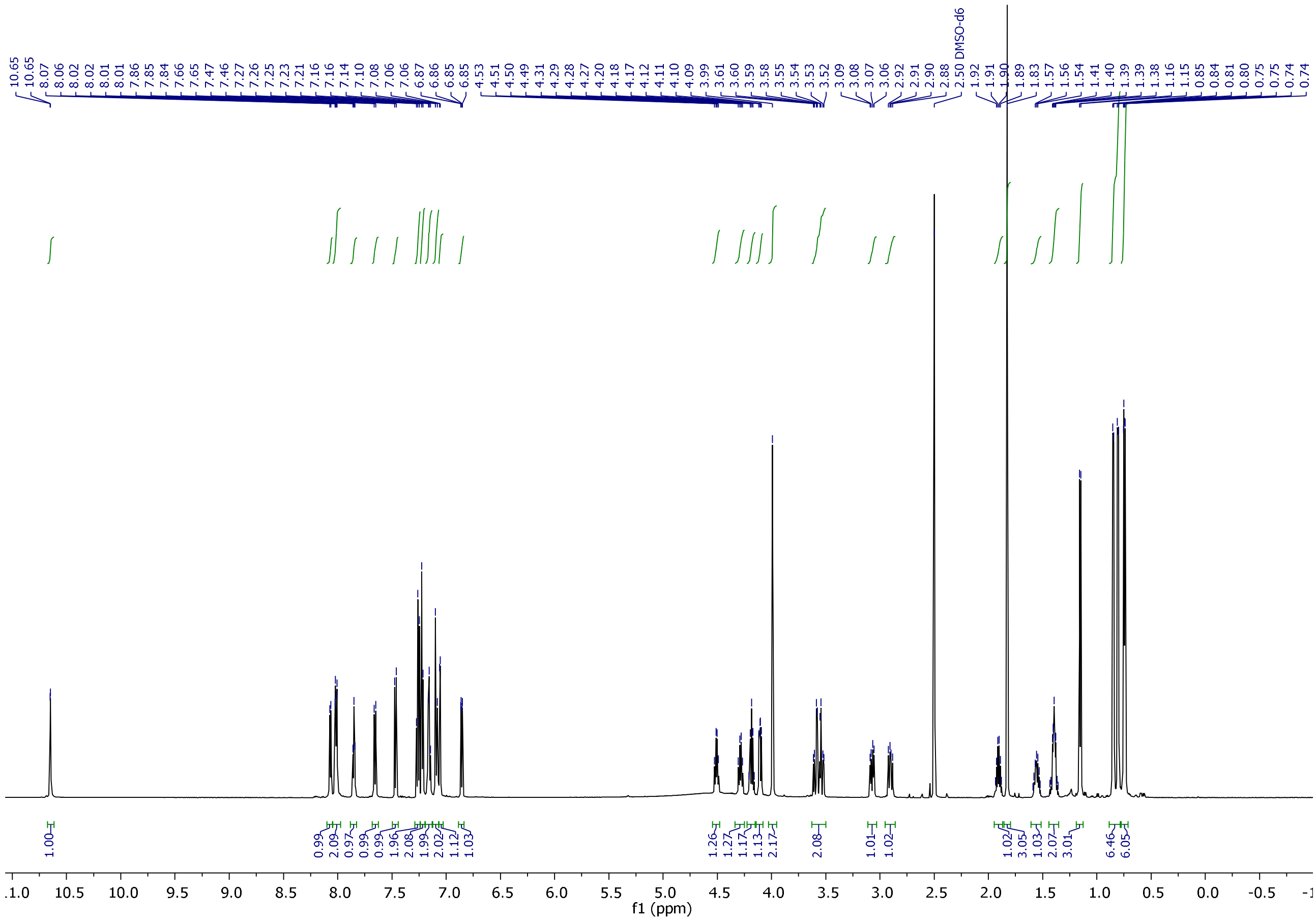

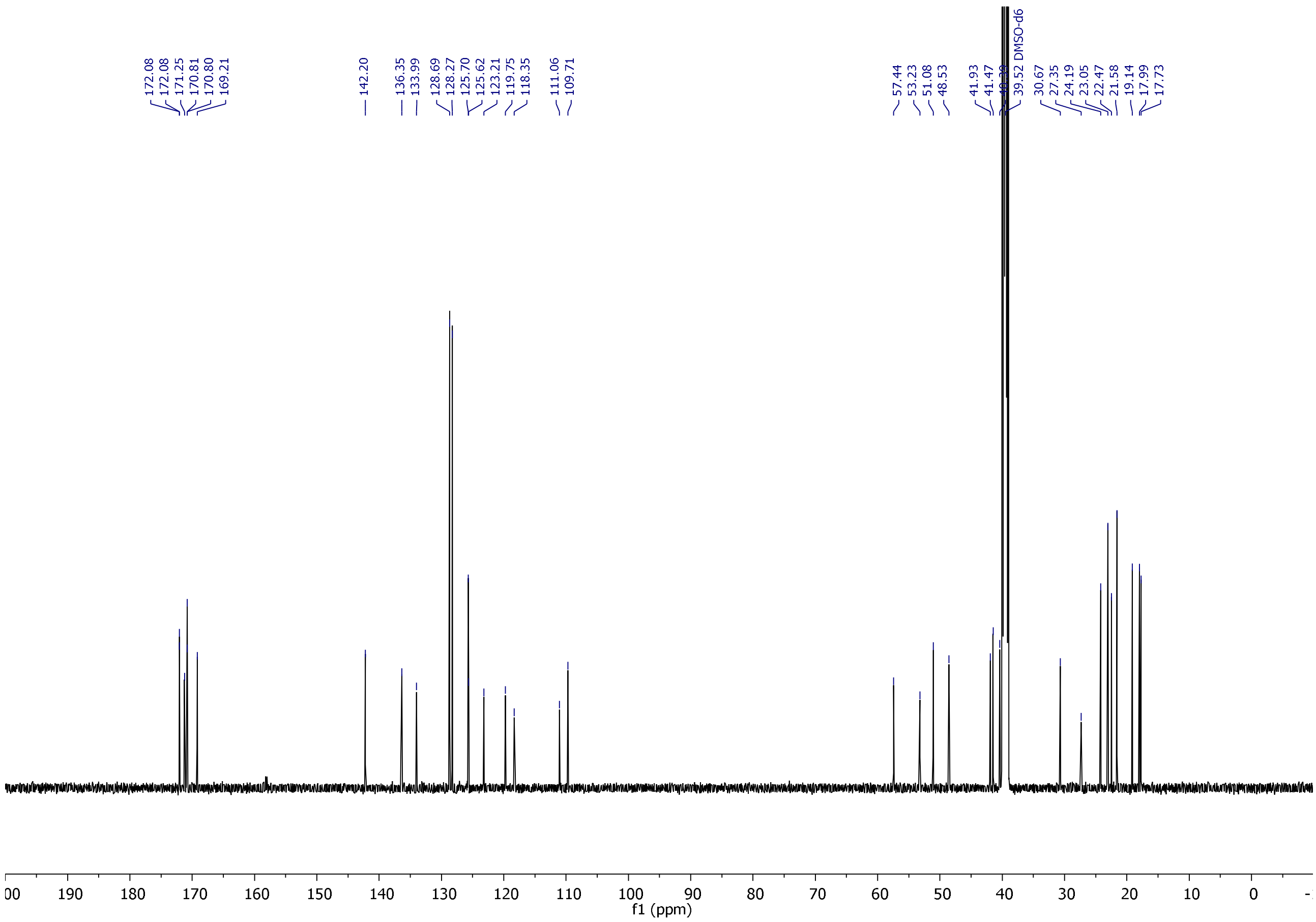

Peptide 10d

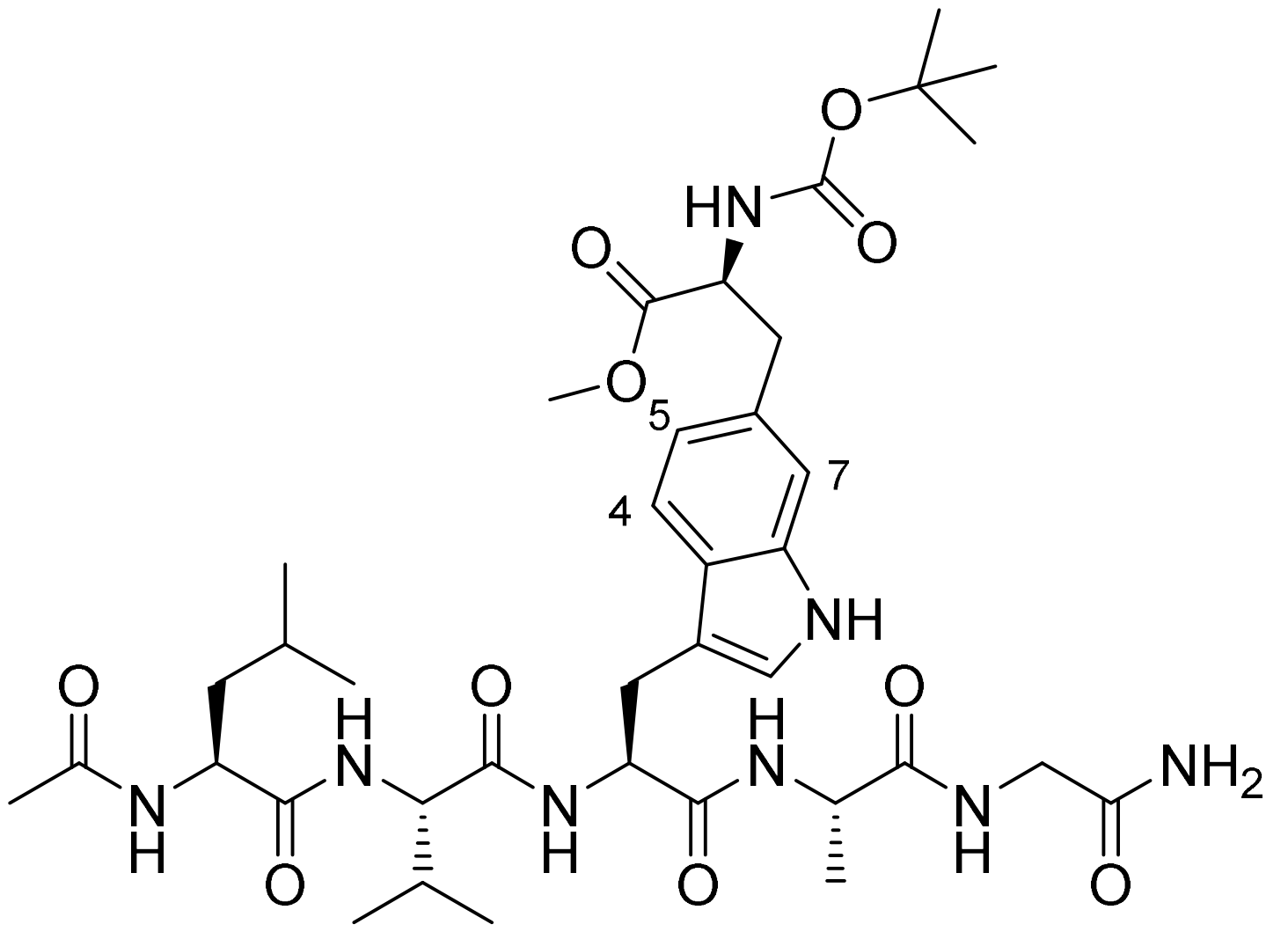

$C_{38}H_{58}N_8O_{10}$
MW = 786,93

Peptide **10d** was synthesized according to GP1. Therefore, a stock solution of Ac-Leu-Val-Trp(6-Br)-Ala-Gly-NH_2 (0.101 M) was prepared in a mixture of anhydrous DMF/DMSO (3:2) and degassed under argon atmosphere for 30 min. Separately, activated zinc dust (5.07 mg, 77.5 µmol, 2.6 eq.), Pd(amphos)$_2$Cl$_2$ (2.22 mg, 3.14 µmol, 10 mol%) and N$^{\alpha}$-Boc-L-3-iodoalanine methyl ester (20.11 mg, 61.1 µmol, 2.0 eq.) were charged into a glass vial, which was subsequently placed under argon atmosphere. After 10 min the peptide stock solution (0.101 M, 300 µL, 1.0 eq.) was added to the vial. The reaction mixture was purged with argon for 5 min. The vial was sealed and the suspension was vigorously stirred at 40 °C for 24 h. When the reaction time elapsed, the mixture was allowed to cool to room temperature, and centrifuged. The crude product was purified by preparative RP-HPLC (preparative run protocol 3). Lyophilization provided the product as a colourless solid (21.5 mg, 27.3 µmol, 90%).

^{1}H NMR (600 MHz, [D$_6$]DMSO): δ [ppm] = 10.72 (d, 1H, $^3J_{H\text{-}H}$ = 1.7 Hz; N**H**-indole), 8.08 (d, $^3J_{H\text{-}H}$ = 6.9 Hz, 1H; N**H**^{Ala}), 8.04-7.95 (m, 2H; N**H**^{Trp}, N**H**^{Leu}), 7.87 (t, $^3J_{H\text{-}H}$ = 5.7 Hz, 1H; N**H**^{Gly}), 7.65 (d, $^3J_{H\text{-}H}$ = 8.7 Hz, 1H; N**H**^{Val}), 7.48 (d, $^3J_{H\text{-}H}$ = 8.0 Hz, 1H; C4-**H**^{Trp}), 7.22 (d, $^3J_{H\text{-}H}$ = 7.9 Hz, 1H; N**H**^{Ala2}), 7.16 (m, 2H, C7-**H**^{Trp}; N**H**$_2$), 7.08 (s, 2H; C2-**H**^{Trp}, N**H**$_2$), 6.84 (d, $^3J_{H\text{-}H}$ = 8.0 Hz, 1H; C5-**H**^{Trp}), 4.52 (m, 1H; C$_\alpha$-**H**^{Trp}), 4.29 (m, 1H; C$_\alpha$-**H**^{Leu}), 4.18 (m, 1H; C$_\alpha$-**H**^{Ala}), 4.14 (m, 1H; C$_\alpha$-**H**^{Ala2}), 4.11 (dd, $^3J_{H\text{-}H}$ = 8.7 Hz, $^3J_{H\text{-}H}$ = 6.9 Hz, 1H; C$_\alpha$-**H**^{Val}), 3.65-3.58 (m, 4H; C$_\alpha$-**H**$_2$^{Gly}, C**H**$_3$-OMe), 3.54 (dd, $^2J_{H\text{-}H}$ = 16.9 Hz, $^3J_{H\text{-}H}$ = 5.7 Hz, 1H; C$_\alpha$-**H**^{Gly}), 3.09 (dd, $^2J_{H\text{-}H}$ = 14.8 Hz, $^3J_{H\text{-}H}$ = 5.5 Hz, 1H; C$_\beta$-**H**^{Trp}), 3.03 (dd, $^2J_{H\text{-}H}$ = 13.8 Hz, $^3J_{H\text{-}H}$ = 5.0 Hz, 1H; C$_\beta$-**H**^{Ala2}), 2.95-2.85 (m, 2H; C$_\beta$-**H**^{Trp}, C$_\beta$-**H**^{Ala2}), 1.92 (m, 1H; C**H**^{Val}), 1.83 (s, 3H; C**H**$_3$-Ac), 1.56 (m, 1H; C**H**^{Leu}), 1.41 (m, 2H; C-**H**$_2$^{Leu}), 1.38-1.28 (m, 9H; 3C**H**$_3$-Boc), 1.16 (d, $^3J_{H\text{-}H}$ = 7.1 Hz, 3H; C**H**$_3$^{Ala}), 0.86 (d, $^3J_{H\text{-}H}$ = 6.6 Hz, 3H; C**H**$_3$^{Leu}), 0.81 (d, $^3J_{H\text{-}H}$ = 6.6 Hz, 3H; C**H**$_3$^{Leu}), 0.75 (d, $^3J_{H\text{-}H}$ = 6.7 Hz, 3H; C**H**$_3$^{Val}), 0.75 (d, $^3J_{H\text{-}H}$ = 6.7 Hz, 3H; C**H**$_3$^{Val}).

^{13}C NMR (151 MHz, DMSO): δ [ppm] = 172.9 (**C**=O^{Ala2}), 172.1 (**C**=O^{Ala}, **C**=O^{Leu}), 171.3 (**C**=O^{Trp}), 170.8 (**C**=O^{Val}, **C**=O^{Gly}), 169.2 (CH$_3$**C**=O), 155.4 (NH**C**=O), 136.1 (**C**7a), 130.1 (**C**6), 126.0 (**C**3a), 123.4 (**C**2), 119.6 (**C**5), 118.2 (**C**4), 111.6 (**C**7), 109.7 (**C**3), 78.2 (**C**-Boc), 57.4 (**C**$_\alpha$^{Val}), 56.0 (**C**$_\alpha$^{Ala2}),

53.2 (C_α^{Trp}), 51.7 (CH_3-OMe), 51.1 (C_α^{Leu}), 48.6 (C_α^{Ala}), 41.9 (C_α^{Gly}), 40.4 (C_β^{Leu}), 36.8 (C_β^{Ala2}), 30.7 (C_β^{Val}), 28.1 (3CH_3-Boc), 27.4 (C_β^{Trp}), 24.2 (C_γ^{Leu}), 23.0 (CH_3^{Leu}), 22.5 (CH_3C=O), 21.6 (CH_3^{Leu}), 19.1 (CH_3^{Val}), 18.0 (CH_3^{Val}), 17.7 (CH_3^{Ala}).

HRMS (ESI): *m/z* calcd: 787.4349 $[M+H]^+$, 804.4614 $[M+NH_4]^+$, 809.4168 $[M+Na]^+$; found: 787.4358 $[M+H]^+$, 804.4622 $[M+NH_4]^+$, 809.4179 $[M+Na]^+$.

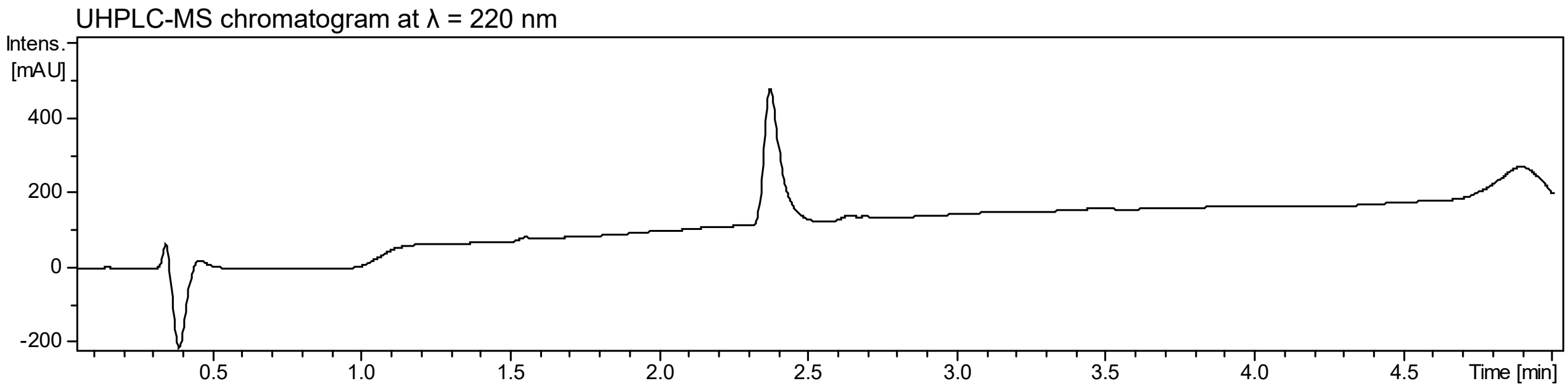

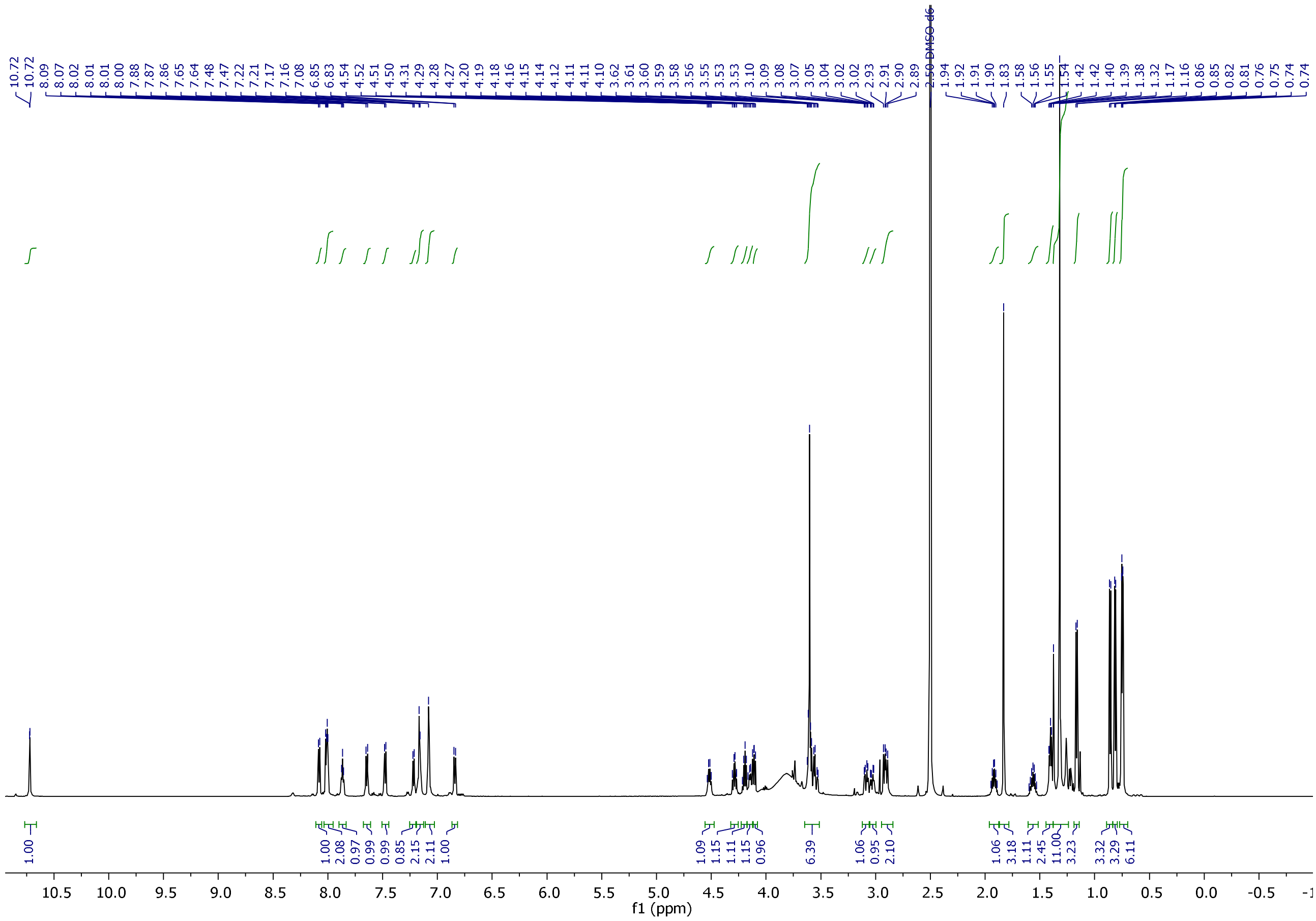
2.50 DMSO-d6
f1 (ppm)

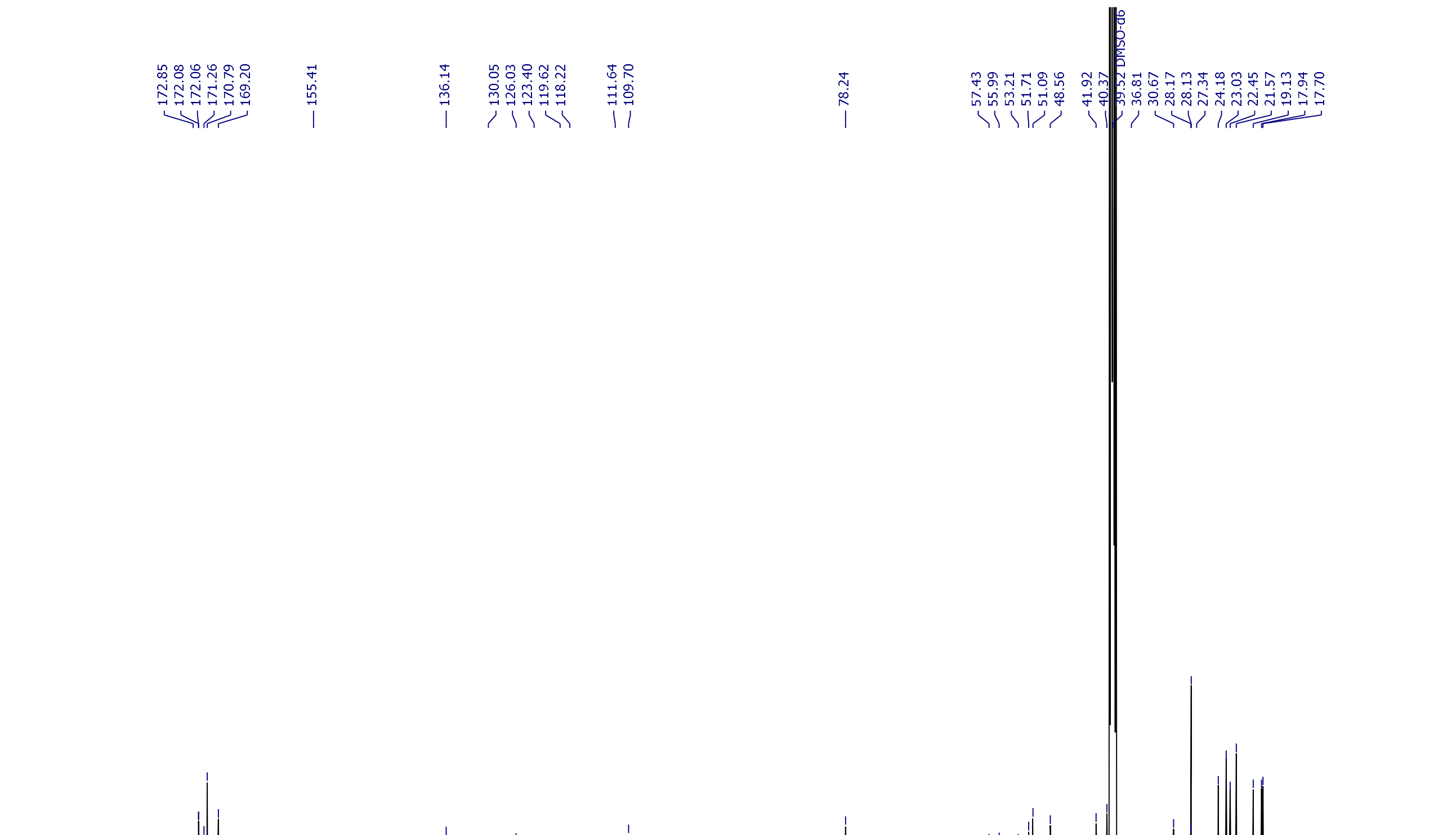
39.52 DMSO-d6
f1 (ppm)

Supplementary Figures to Tables 1-4

UHPLC-MS Chromatograms at 220 nm of the crude-reaction and isolated products (when not previously reported). Peaks marked with "*" correspond to the dehalogenated peptide.

Entry 1

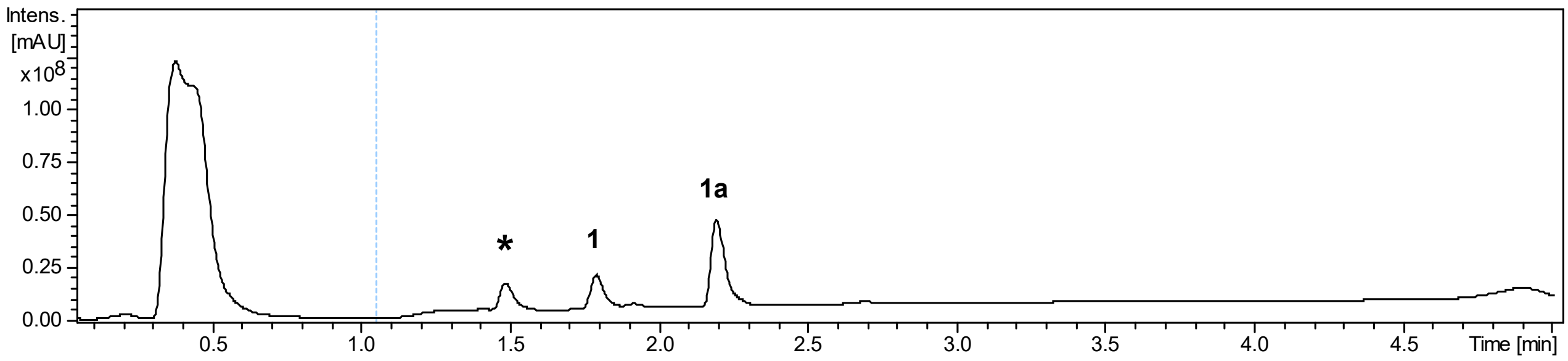

Entry 2

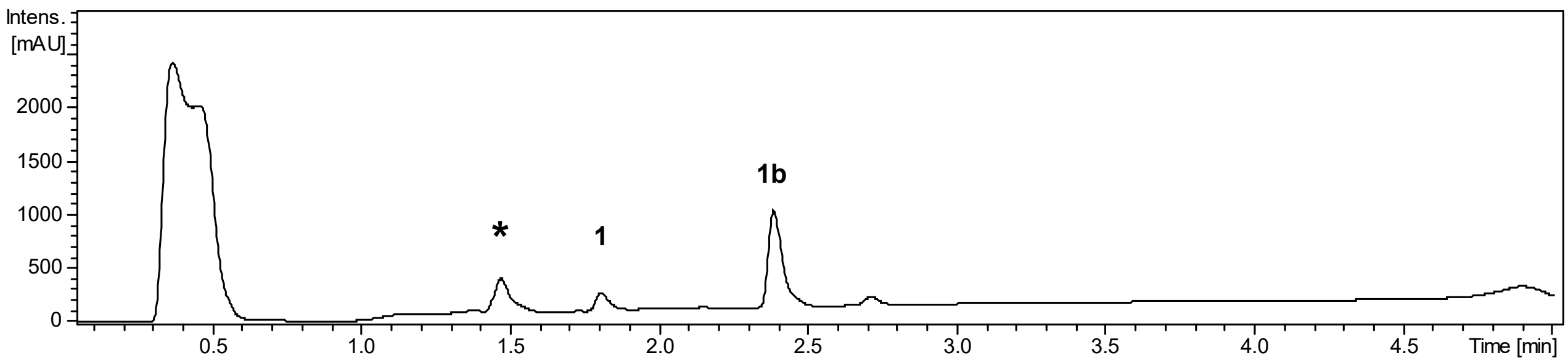

Entry 3

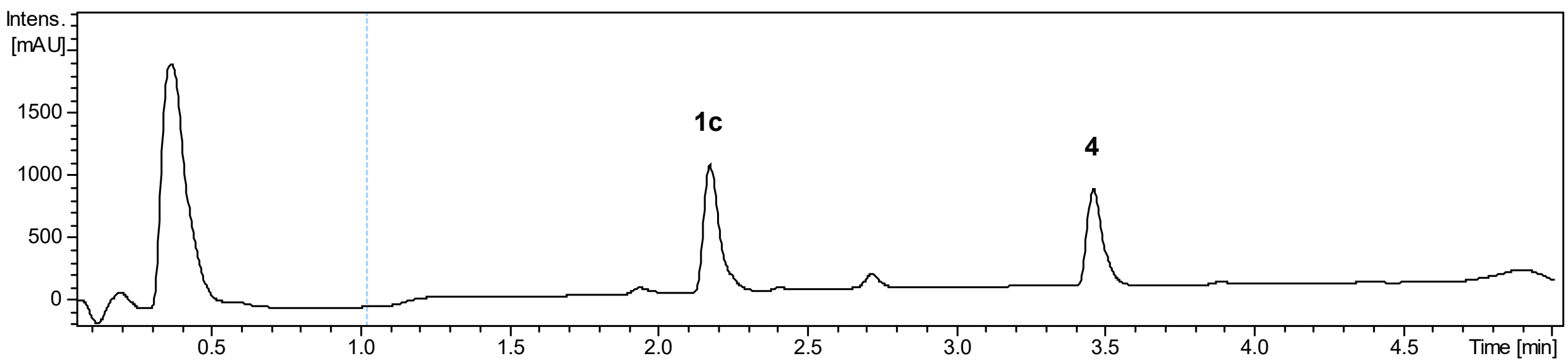

Entry 4

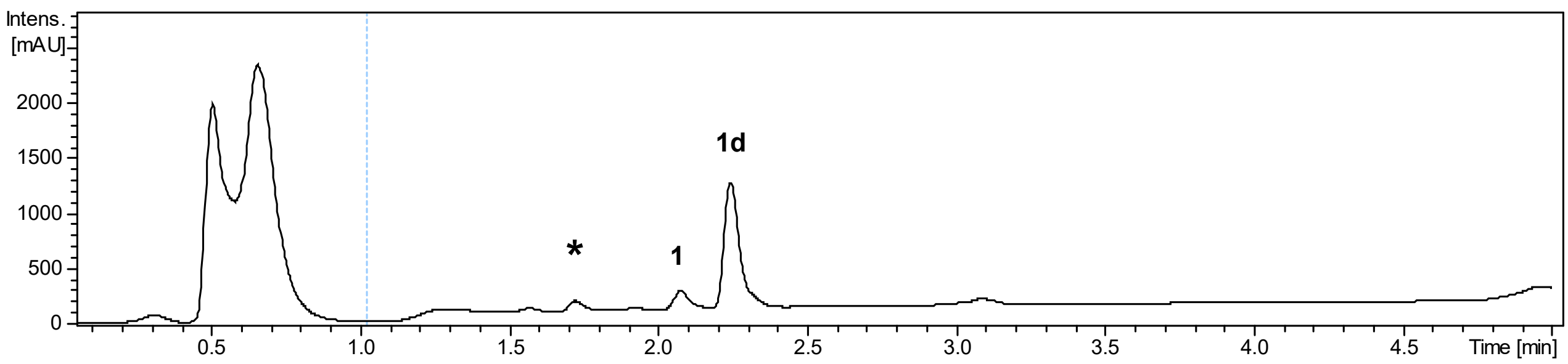

Entry 5

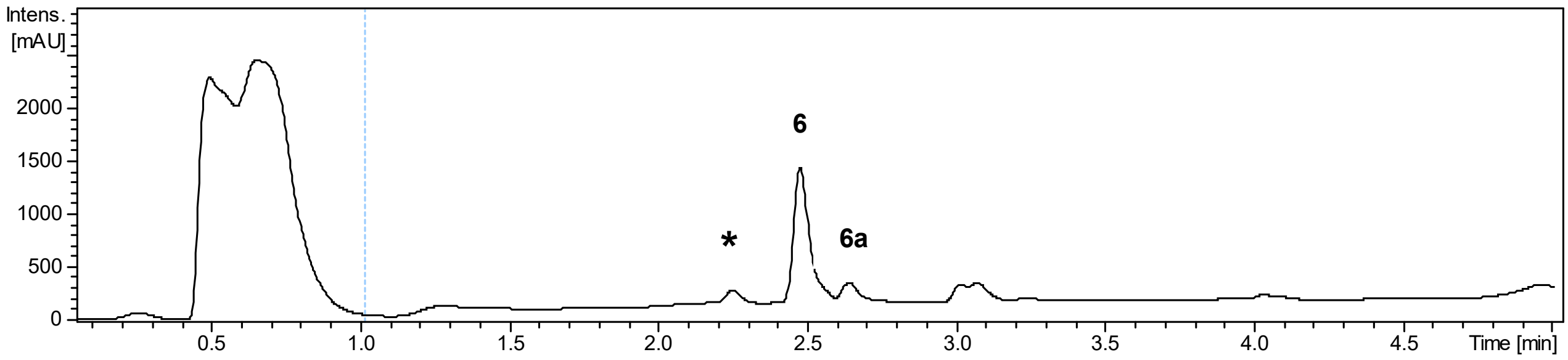

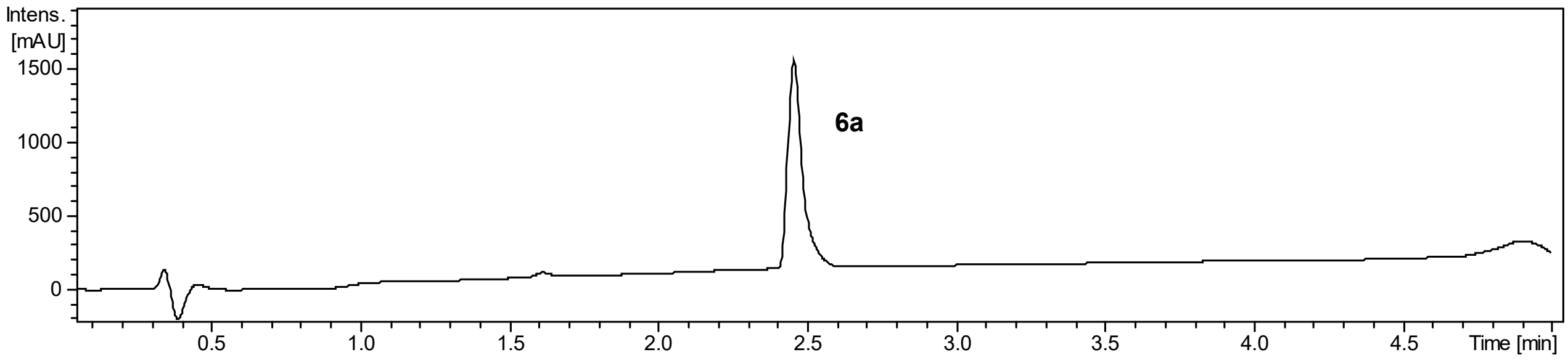

Entry 6

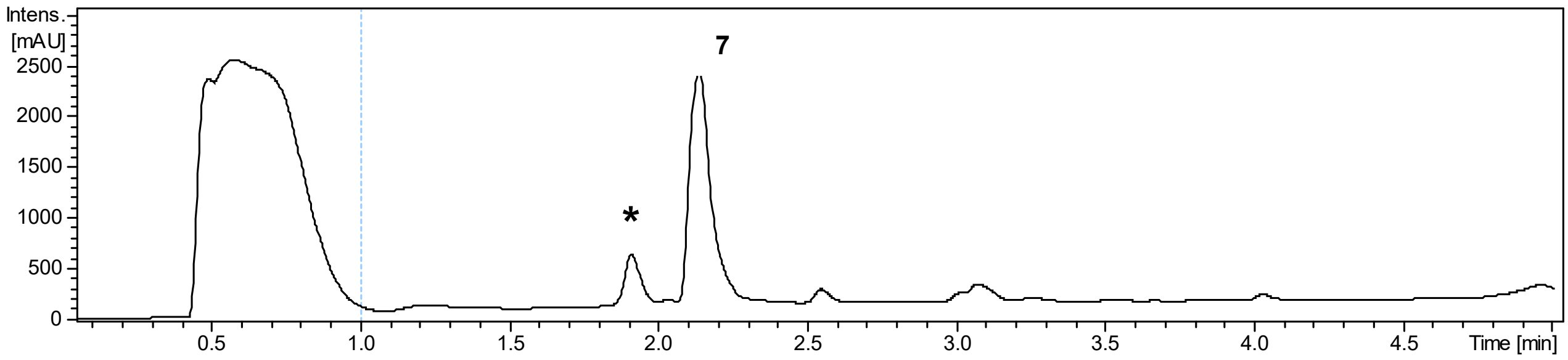

Entry 7

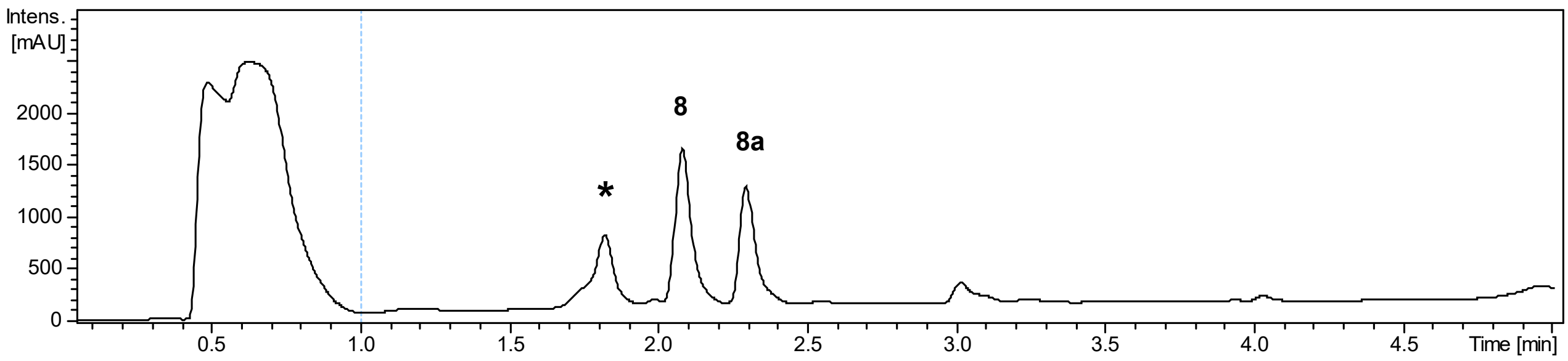

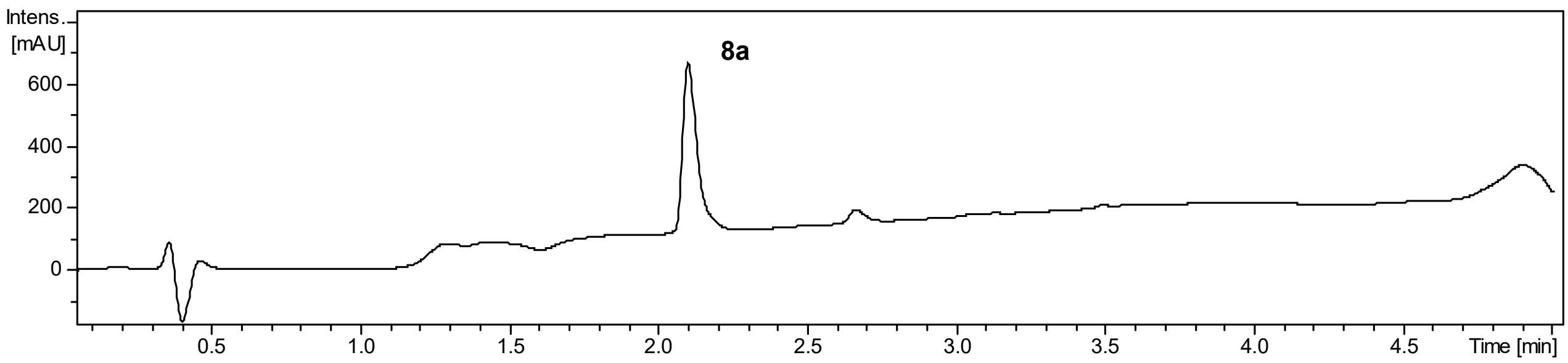

Entry 8

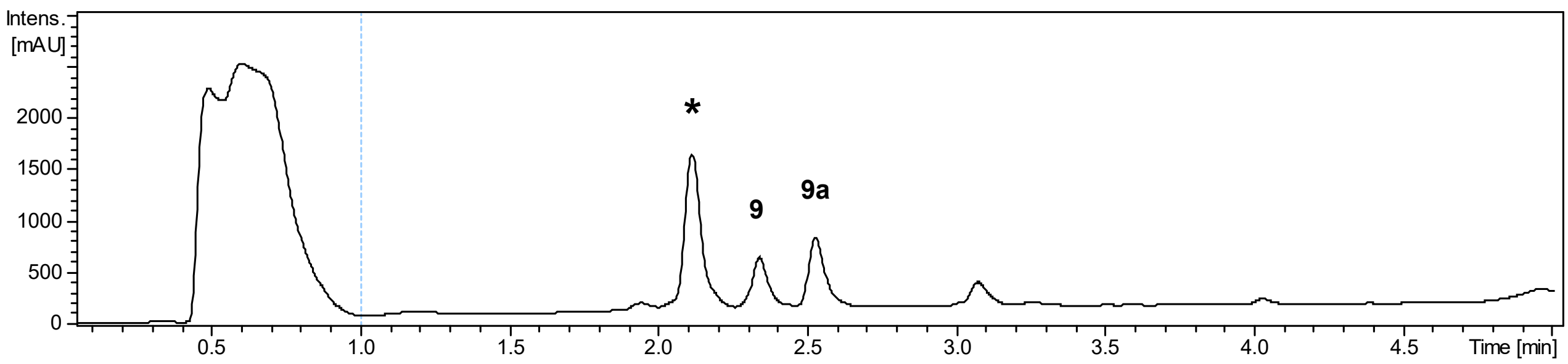

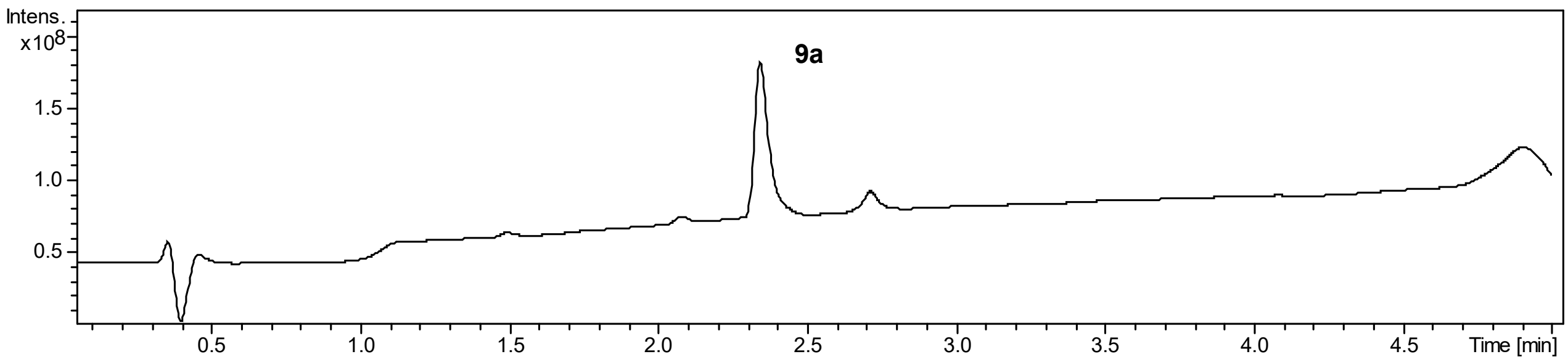

Entry 9

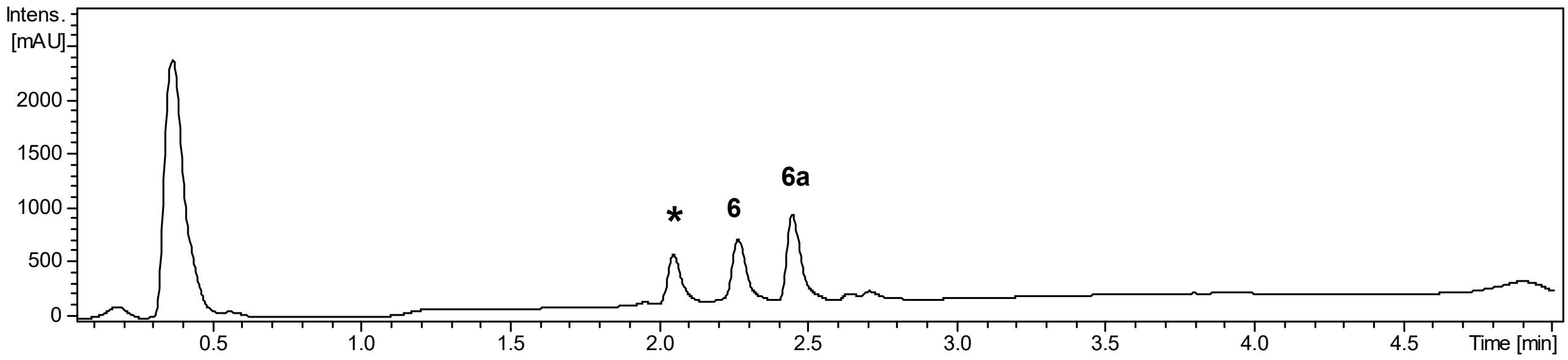

Entry 10

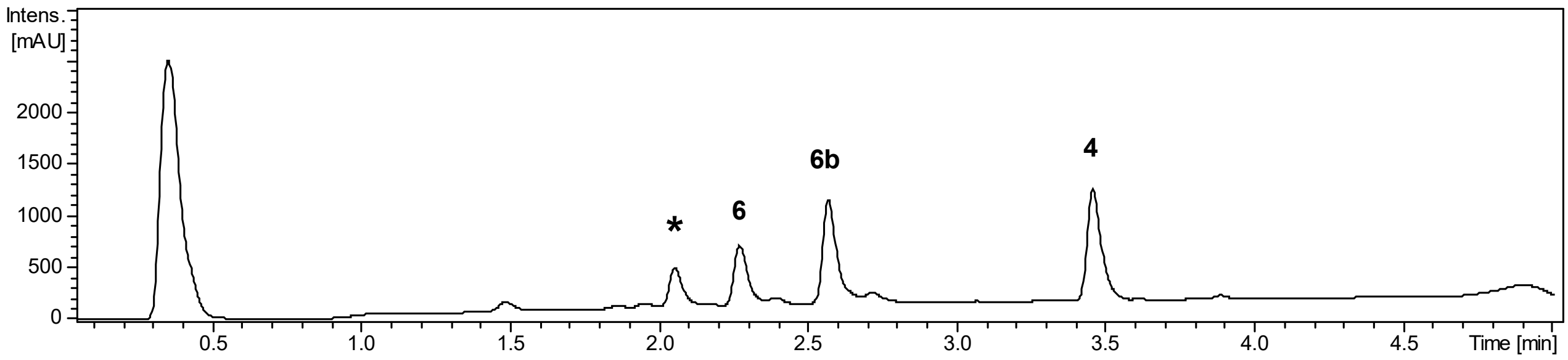

Entry 11

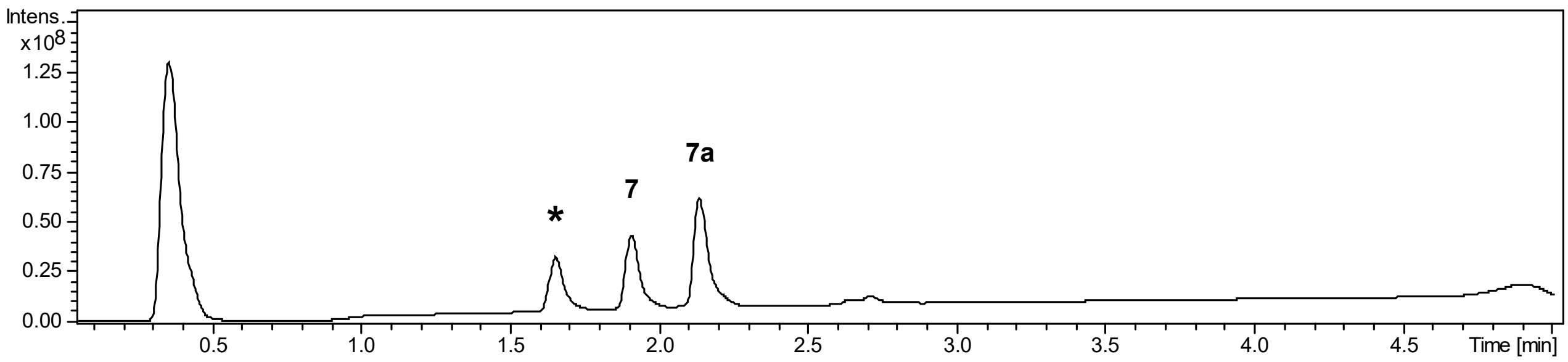

Entry 12

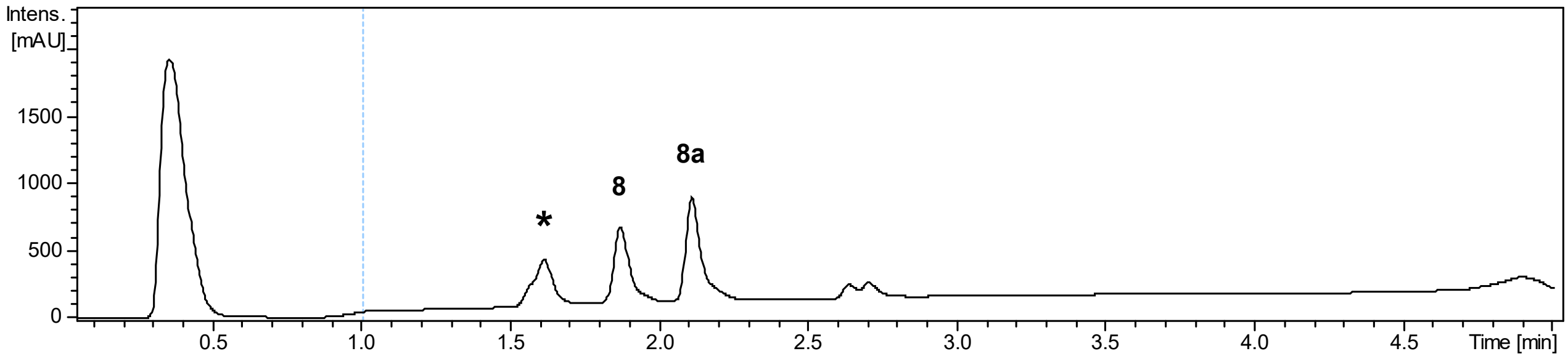

Entry 13

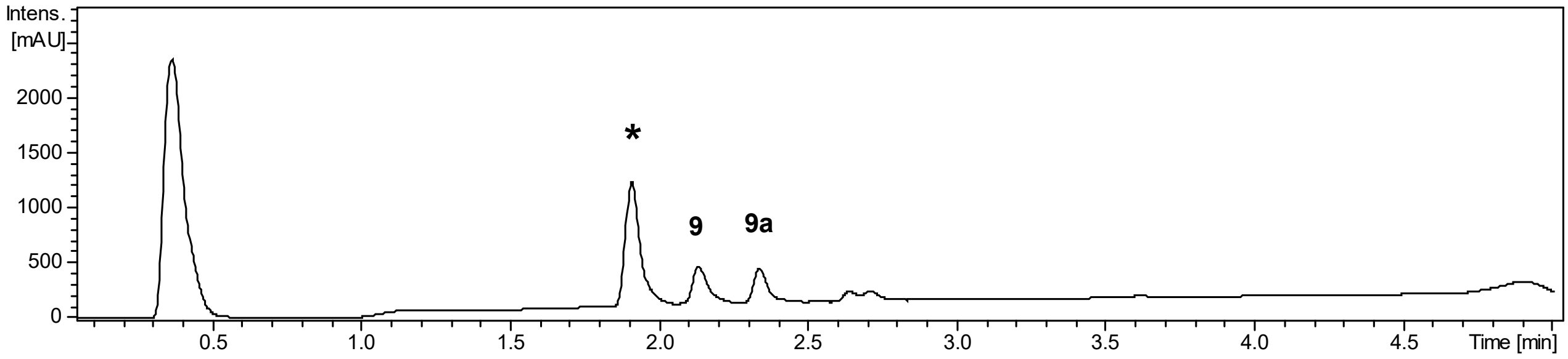

Entry 14

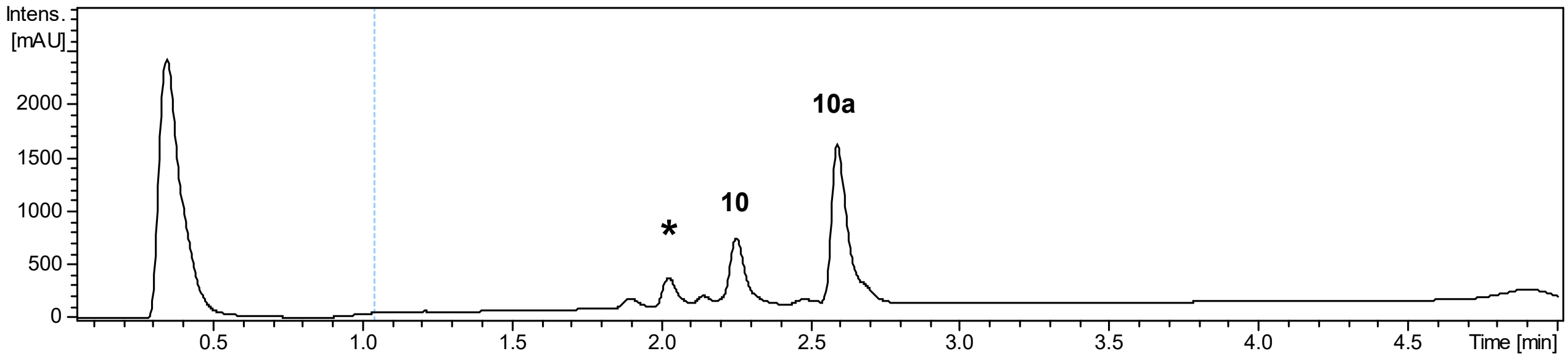

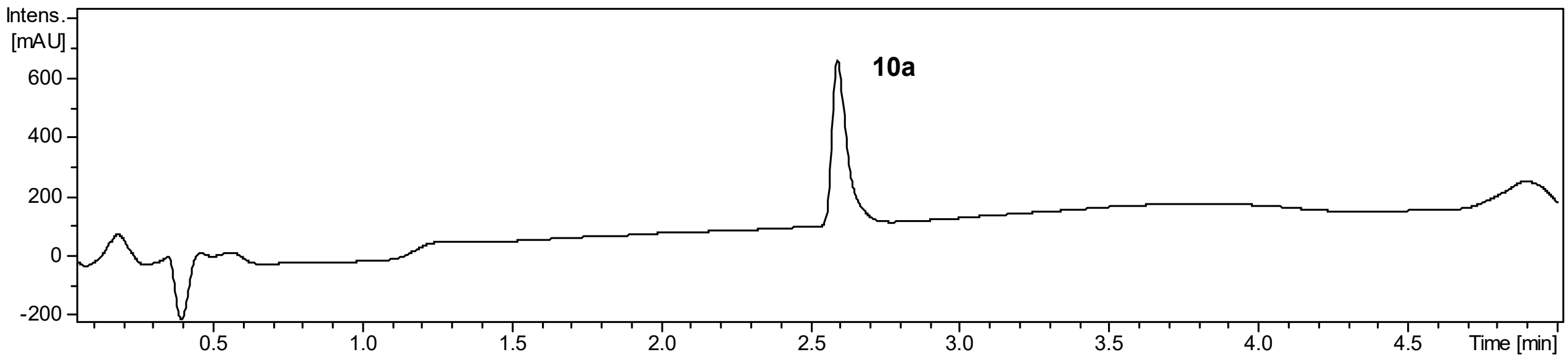

Entry 15

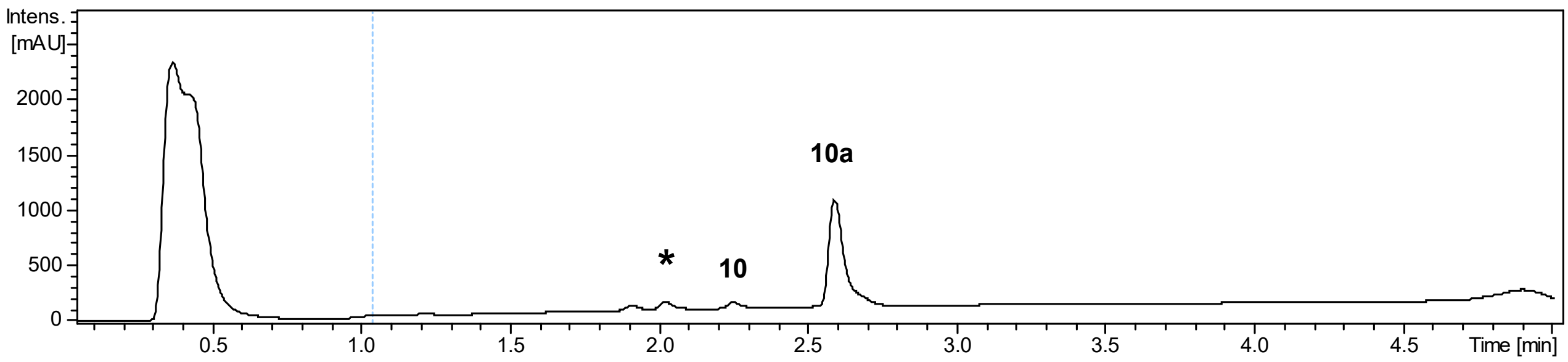

Entry 16

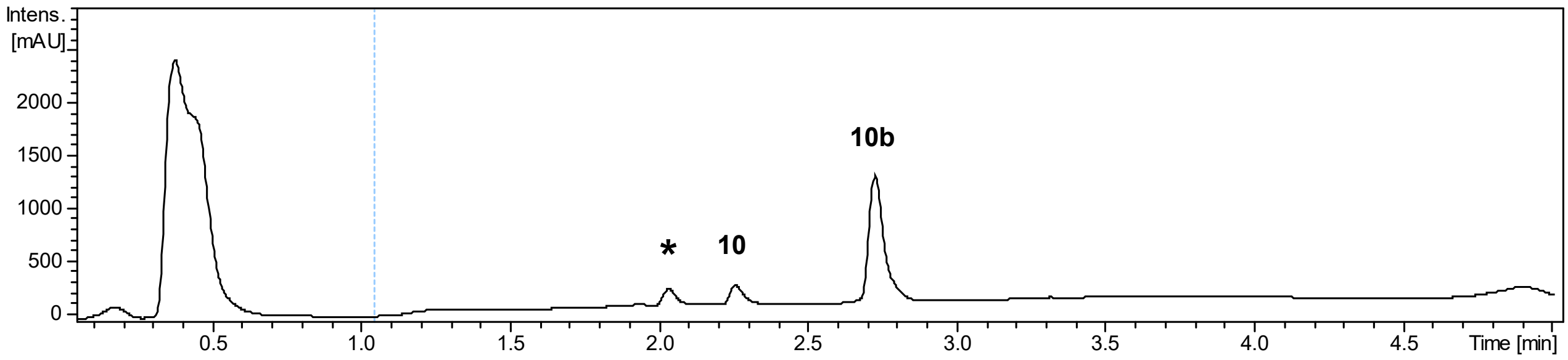

Entry 17

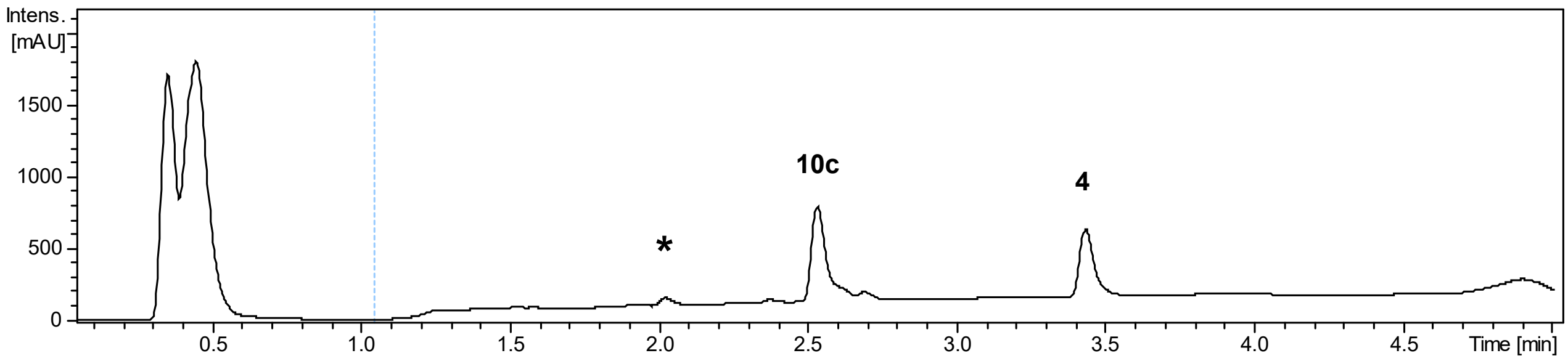

Entry 18

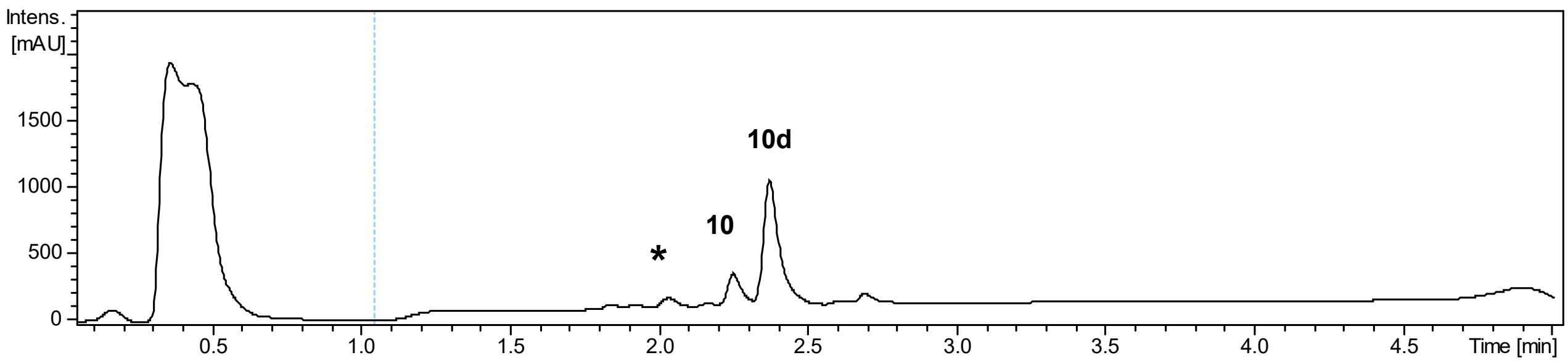

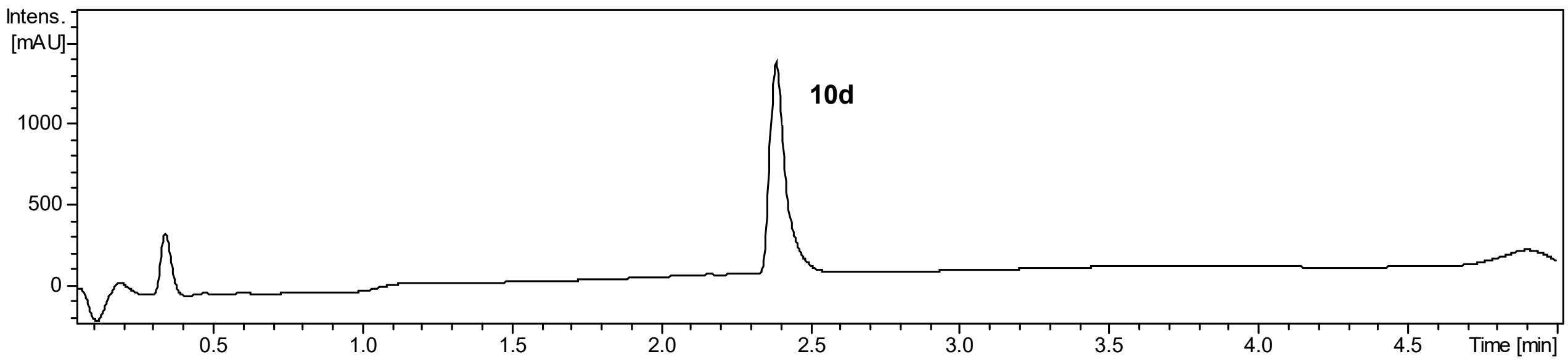

Entry 19

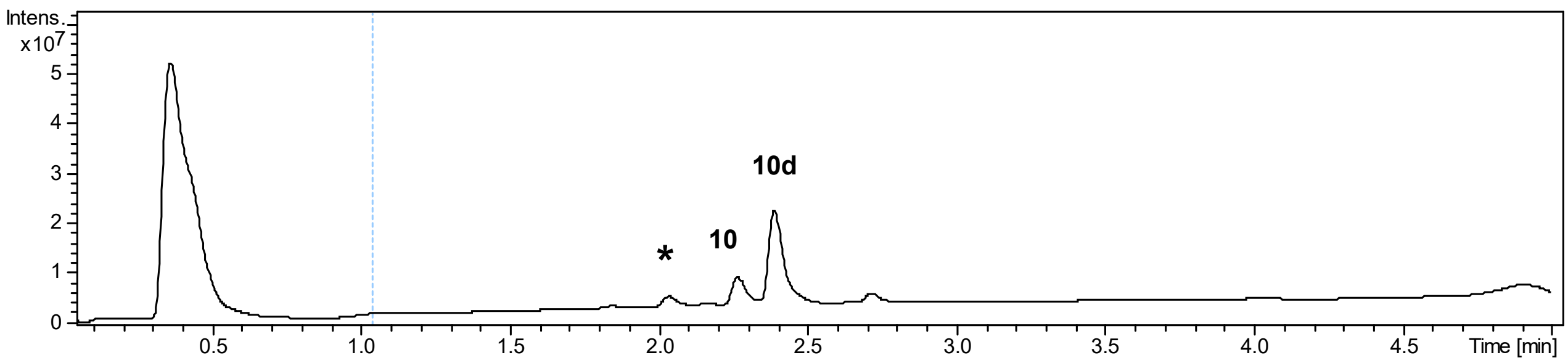

References

[1] M. S. Newman, F. J. Evans Jr., "The Reformatsky Reaction: Effect of Alkyl Group in Alkyl α-Bromopropionates", *Journal of the American Chemical Society* **77**, (1955): 946.

[2] N. Montua, N. Sewald, "Extended Biocatalytic Halogenation Cascades Involving a Single-Polypeptide Regeneration System for Diffusible FADH2", *Chembiochem : a European journal of chemical biology* **24**, (2023): e202300478.

[3] G. H. Daub, R. N. Castle, "The Synthesis of Some Substituted Benzyl Iodides", *Journal of Organic Chemistry* **19**, (1954): 1571.
